# Fluorescence to measure light intensity

**DOI:** 10.1038/s41592-023-02063-y

**Published:** 2023-11-23

**Authors:** Aliénor Lahlou, Hessam Sepasi Tehrani, Ian Coghill, Yuriy Shpinov, Mrinal Mandal, Marie-Aude Plamont, Isabelle Aujard, Yuxi Niu, Ladislav Nedbal, Dusan Lazár, Pierre Mahou, Willy Supatto, Emmanuel Beaurepaire, Isabelle Eisenmann, Nicolas Desprat, Vincent Croquette, Raphaël Jeanneret, Thomas Le Saux, Ludovic Jullien

**Affiliations:** 1 PASTEUR, Department of Chemistry, École Normale Supérieure, PSL University, Sorbonne University, CNRS, Paris, France; 2 Sony Computer Science Laboratories, Paris, France; 3 Institute of Bio- and Geosciences/Plant Sciences, Forschungszentrum Jülich, Jülich, Germany; 4 Department of Biophysics, Faculty of Science, Palacký University, Olomouc, Czech Republic; 5 Laboratory for Optics and Biosciences, Ecole Polytechnique, CNRS, INSERM, IP Paris, Palaiseau, France; 6 Laboratory of Physics of the École Normale Supérieure, University of PSL, CNRS, Sorbonne University, University of Paris City, Paris, France; 7 Institute of Biology of ENS (IBENS), École Normale Supérieure, CNRS, INSERM, University of PSL, Paris, France

**Keywords:** Fluorescence imaging, Chemical tools

## Abstract

Despite the need for quantitative measurements of light intensity across many scientific disciplines, existing technologies for measuring light dose at the sample of a fluorescence microscope cannot simultaneously retrieve light intensity along with spatial distribution over a wide range of wavelengths and intensities. To address this limitation, we developed two rapid and straightforward protocols that use organic dyes and fluorescent proteins as actinometers. The first protocol relies on molecular systems whose fluorescence intensity decays and/or rises in a monoexponential fashion when constant light is applied. The second protocol relies on a broad-absorbing photochemically inert fluorophore to back-calculate the light intensity from one wavelength to another. As a demonstration of their use, the protocols are applied to quantitatively characterize the spatial distribution of light of various fluorescence imaging systems, and to calibrate illumination of commercially available instruments and light sources.

## Abstract (web summary)

Two methods for fluorescence-based actinometry using organic dyes and photoconvertible fluorescent proteins enable rapid and precise measurement of light intensity at the sample in fluorescence microscopes.

## Main

Involved in key mechanisms of living systems (for example, photosynthesis, vision), photochemistry has found multiple applications at micro- and/or macro-scales from producing molecules^1^ to designing medical protocols (for example, photodynamic therapy^2^). In bioimaging, optical microscopists balance light intensity to get optimal signals without phototoxicity. In optogenetics, biologists use photons for triggering physiological processes^3^. In photocatalysis^4^, chemists exploit photons for driving the synergetic action of light-absorbing and metallic catalysts. Nowadays, a vast community of biologists, chemists, engineers and physicists are concerned with delivering precise numbers of photons.

Illumination systems require accurate quantitative characterization to ensure reproducibility as well as to enable a fair comparison of results obtained by various groups^5^, or to rationally choose parameters such as duration of light application for delivering the right number of photons to a sample. Here we address light intensity, more precisely irradiance, which is a surfacic power (W m^−2^), alternatively known as photon flux density in units of mol m^−2^ s^−1^ (or E m^−2^ s^−1^; Supplementary Note 4) that we will use in the following as it is wavelength independent.

Several tools are helpful to measure light intensity^6,7^. Light meters provide a fast response over a wide range of wavelengths and intensities^8,9^. However, their detectors integrate light over their surface and do not yield any information on the spatial distribution of light. A further measurement of the area of the illuminated surface is required to retrieve the light intensity. Fluorescent microscope slides deliver an image read-out of the spatial profile of illumination in imaging systems. Yet, the latter is affected by optical aberrations and the detection efficiency. Therefore, it cannot be relied on for retrieving accurate spatial information. Moreover, it does not give access to absolute light intensity. Eventually, light meters and fluorescent microscope slides sense light at sample surfaces only and experience geometrical constraints from relying on large and rigid sensing elements.

Actinometers provide an alternative approach. Light intensity is directly retrieved from following the time course of their reaction extent on constant illumination^10^. This approach can further yield spatial information when used with an imaging system. Moreover, since they take the form of liquid solutions, actinometers can measure light in samples of various sizes and geometries. However, most established and new actinometers have relied on absorbance to report on reaction extent^10–12^: an observable that is not very sensitive and is not easily accessible in imaging systems. Furthermore, they are generally restricted for the ranges of wavelengths and light intensities.

Fluorescence is a more sensitive observable than absorbance^13^ and is accessible to imaging systems. Harnessing fluorophore photobleaching has been proposed for quantitative measurement of light intensity^14^. However, the photobleaching kinetics can be complex and exhibit environmental dependence, and therefore the use of photobleaching kinetics is limited to situations of high light levels or long time periods since most fluorophores are strongly resistant to photobleaching.

Here, we demonstrate the use of synthetic and genetically encoded fluorescent photoactivatable systems that we previously reported^15–19^ as actinometers, whereby fluorescence is used for reporting the extent of the photoconversion reaction. Such conversions proceed much more rapidly than photobleaching, and thus these systems can be used in weak-light situations (or short time periods). To allow for measurement over a wider range of wavelengths, we complement our proposed method with a broad-absorbing photochemically inert fluorophore that enables light intensity at one wavelength to be used to calculate light intensity at a second wavelength. In this Article, we report on the relevant features of these systems for measuring light intensity and demonstrate their use in characterizing illumination systems at both microscopic and macroscopic scales with samples of various sizes and geometries.

## Results

### The first protocol to measure light intensity

**Fig. 1:**
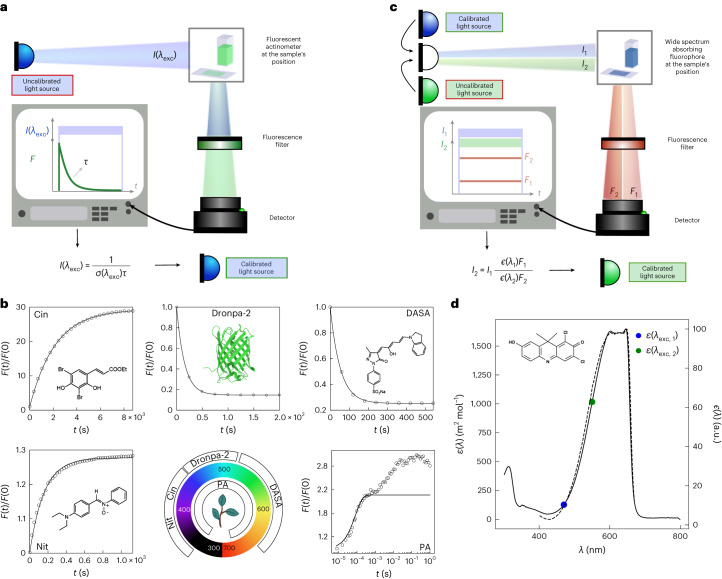
Fluorescence reporting for retrieving light intensity. **a**, First protocol with a fluorescent actinometer. A jump of constant light *I* is applied onto the actinometer. The time evolution of its fluorescence signal *F* is recorded and fit with a monoexponential curve to extract its characteristic time *τ*. *I* is retrieved from *τ* by using the photoconversion cross section *σ* of the actinometer. **b**, Five fluorescent actinometers covering the UV-vis range in action. Monoexponential fit of the time evolution of the normalized fluorescence signal *F*(*t*)/*F*(0) provides *τ* (Supplementary Table 3). **c**, Second protocol with a fluorophore to transfer information on light intensity from one wavelength to another. Lights at wavelengths *λ*_1_ (with intensity *I*_1_, known) and *λ*_2_ (with intensity *I*_2_, to be measured) are successively applied onto the fluorophore and the associated fluorescence signals *F*_1_ and *F*_2_ are recorded at a same emission wavelength. *I*_2_ is extracted from *F*_1_ and *F*_2_ by using *I*_1_ and the tabulated fluorescence excitation spectrum *ϵ*(*λ*) of the fluorophore. **d**, Absorption (*ε*(*λ*); dotted line) and normalized fluorescence excitation (*ϵ*(*λ*); solid line) spectra of DDAO. *ε*(*λ*_1_) and *ε*(*λ*_2_) indicated by blue and green disks, respectively, are used to retrieve *I*(*λ*_2_) in **c** (text and Supplementary Tables 1–3).

The protocol to measure light intensity (Fig. 1a) exploits molecular actinometers, which react on absorbing the excitation light to be characterized (Supplementary Note 5). The absorbance of their solution is adjusted low enough to ensure that light intensity is essentially constant along the optical path (below 0.15, an easily met threshold). The protocol begins with the sudden exposure of the actinometer solution to illumination, set at the level of light intensity *I* to be measured. One subsequently collects the time evolution of the fluorescence signal, which reports on the actinometer photoconversion extent. It is processed by the fitting of a monoexponential curve, to enable the retrieval of the associated characteristic time *τ*, which evaluates the time scale of the actinometer photoconversion. In an appropriate range of light intensity, *I* is equal to the inverse of the product *στ* where *σ* is the photoconversion cross section (a measure of the molecular surface leading to the actinometer photoconversion after light absorption) (Fig. 1a) and it is measured within a 20% achievable uncertainty (Supplementary Note 7). Where photoconversion occurs rapidly, on a time scale where molecular motion is minimal, it is possible to retrieve a map of the spatial distribution of light intensity. However, if the molecules can visit the whole irradiated area at the time scale of the actinometer photoconversion, only mean light intensity values can be obtained^16^.

The choice of the reported actinometers has been guided by several considerations. First, we wanted to illustrate two mechanisms enabling fluorescence to be used as a reporter of their photoconversion. In the first one, the actinometer and/or its photoproducts are intrinsically fluorescent: the fluorescence intensity changes in accordance with the actinometer photoconversion and the actinometer alone is sufficient to retrieve *I*. This mechanism is simple but its implementation relies on specific features, which led us to identify appropriate candidates. Indeed, photoconversion and fluorescence emission are competitive deexcitation processes from an excited state. By contrast, when the actinometer and its photoproducts are nonfluorescent, the actinometer photoconversion only drives a change of absorbance. Here, a photochemically inert fluorophore can be added to report, through fluorescence, on the time evolution of the absorbance at the excitation wavelength by the inner filter effect (Supplementary Information Section 5.1.2). In this last mechanism, the absorbance is advantageously adjusted around 0.15 to increase the amplitude of the time variation of the fluorescence signal, and thus sensitivity. Then we wanted to make fluorescent actinometers accessible to different communities of end users. Hence, we report on easily synthesized chemicals for the chemists, whereas we propose to use proteins and photosynthetic organisms for end users with access to biological techniques. Eventually, the five reported actinometers cover the entire ultraviolet-visible light (UV-vis) wavelength range for measurement of light intensity (Fig. 1c and Supplementary Information Sections 7.4–7.6):

- Two actinometers for the UV-A wavelength range (relevant for decontamination of materials^20^, evaluation of environmental radiation^21^, photoactivation of many caged molecules in optogenetics^22^, photocatalysis with metal complexes^4^ and so on): (1) the dark (*E*)-3-(3,5-dibromo-2,4-dihydroxyphenyl) acrylic acid ethyl ester (Cin)^15,23^, which irreversibly converts under illumination between 350 and 420 nm into the bright 6,8-dibromo-7-hydroxycoumarin fluorescing between 400 and 550 nm in Tris pH 7 buffer and (2) the dark *α*-(4-diethylamino)phenyl)-*N*-phenylnitrone (Nit)^24^, which irreversibly converts under illumination between 320 and 430 nm into the dark *N*-(*p*-dimethylaminophenyl)formanilide in ethanol^19^. The photochemically inert rhodamine B (RhB) emitting fluorescence between 550 and 650 nm is selected here for optimally reporting on Nit photoconversion by inner filter effect.
- One actinometer for the blue wavelength range (important in optogenetics for photoactivating opsins, FAD CRY, FAD BLUF and FMN LOV systems^3^, or driving photosynthesis^25^). A bright reversibly photoswitchable fluorescent protein Dronpa-2 (or M159T)^26^, contained within *Escherichia coli* or eucaryotic cells, or in buffered solution or polyacrylamide gel, emitting fluorescence between 500 and 600 nm, which reversibly converts into a dark photoisomerized state under illumination between 400 and 550 nm.
- One actinometer for the green to red wavelength range (important for photoactivating opsins or bilin PHY^3^ in optogenetics, or driving photosynthesis^26^). In acetonitrile, the donor-acceptor Stenhouse dye DASA (sodium 4-(4-((2*Z*,4*E*)-2-hydroxy-5-(indolin-1-yl)penta-2,4-dien-1-ylidene)-3-methyl-5-oxo-4,5-dihydro-1H-pyra-zol-1-yl)benzenesulfonate)^17^ emitting fluorescence extending up to 675 nm reversibly converts into a dark state under illumination between 530 and 670 nm.
- As the width of the absorption band of the preceding fluorescent actinometers is limited, which necessitates several of them covering the whole range of wavelengths, we eventually report on the last actinometer, the photosynthetic apparatus of algae, which can provide an estimate of light intensity for the entire visible range of wavelengths. In oxygenic photosynthetic organisms, a few percent of collected sunlight energy is released as fluorescence in the 650–800 nm range^27^. When exposed to constant light at sun-like intensity, the fluorescence of dark-adapted photosynthetic organisms rises in less than 1 s from a minimum to a maximum via intermediate steps^28,29^. The rate constant of the fastest step linearly depends on the light intensity^30,31^. Usefully, its value does not considerably depend on the type of photosynthetic organism^32^.

Characterization and validation of the five above-mentioned actinometers as well as specific protocols for their use are reported in the Supplementary Notes 2 and 7, respectively. Table 1 provides information to facilitate the selection of the most optimal actinometer to use for specific scenarios. The tabulated parameters will enable end users to reliably measure light intensity as long as they follow the reported measurement protocols. To further facilitate the use of these actinometers, we provide online access to the actinometer properties (https://chart-studio.plotly.com/~Alienor134/#/) and to codes and user-friendly applications to process the acquired data without specific installation (https://github.com/DreamRepo/light_calibration).

**Table 1:** Key parameters for choosing a fluorescent actinometer

Actinometer (availability)^a^	*λ*_exc_ (nm)	*λ*_em_ (nm)	*σ*(*λ*_exc_) (±10%, m^2^ mol^−1^)	(*i*(*λ*_exc_)) (E m^−2^ s^−1^(W m^−2^))^b^	5*τ*_min_ (s)
**Cin** (S)	350		940	(0–1.6) 10^−5^((0–5.5))	332
	365	400–550	1,200	(0–1.4) 10^−5^((0–4.6))	298
	380		1,000	(0–1.7) 10^−5^((0–5.3))	294
	405		184	(0–5.0) 10^−5^((0–15))	543
	420		49	(0–1.3) 10^−4^((0–37))	785
**Nit** (S)	365		960	(0–11) 10^−4^((0–360))	4.7
	380	550–650	1,200	(0–7.2) 10^−4^((0–230))	5.8
	405		1,100	(0–7.0) 10^−4^((0–200))	6.5
	420		850	(0–12) 10^−4^((0–340))	4.9
**Dronpa-2** (GE)	445		140 (192)^c^	(3 × 10^−4^–18)((80–4.8 × 10^6^))	2.0 × 10^−3^
	480	500–600	198 (251)^c^	(2 × 10^−4^–10)((50–2.5 × 10^6^))	2.5 × 10^−3^
	500		128 (151)^c^	(3 × 10^−4^–13)((72–1.0 × 10^6^))	3.0 × 10^−3^
**DASA** (S)	530		255	(8–290) 10^−5^((18–660))	6.8
	560	530–670	530	(4–150) 10^−5^((9–320))	6.3
	600		885	(2–72) 10^−5^((4–140))	7.8
	632		1,135	(2–60) 10^−5^((4–110))	7.3
	650		575	(3–140) 10^−5^((6–260))	6.2
**PA** (B)	405		2.0 (±0.4) 10^6^	(0–10^−2^) ((0–3,000))	1^d^
	470	650–800	2.0 (±0.4) 10^6^	(0–10^−2^) ((0–2,600))	1^d^
	650		1.1 (±0.4) 10^6^	(0–10^−2^) ((0–1,900))	1^d^

*λ*_exc_, (*λ*_em_), *σ*(*λ*_exc_) and (*I*(*λ*_exc_)), respectively, designate the excitation wavelength, the range of emission wavelengths, the cross section and the range of reliably measurable light intensity associated to the actinometer photoconversion at *λ*_exc_. 5*τ*_min_ is the minimum measurement duration at the highest measurable light intensity. See Supplementary Note 7.

^a^S, easily synthesized; GE, genetically encoded; B, photosynthetic organism available for sale (chlamycollection.org) (Supplementary Note 1).

^b^The conversion between the units mol of photon m^−2^ s^−1^, E m^−2^ s^−1^ and W m^−2^ is given in Supplementary Note 4.

^c^The first and second numbers provide the values to be used in the Dronpa-2 solution and in the Dronpa-2 labeled fixed cells, and in the Dronpa-2 labeled bacteria, respectively.

^d^5*τ*_min_ is the time requested to record the whole photosynthetic apparatus fluorescence rise.

### The second protocol to measure light intensity

The second protocol to measure light intensity (Fig. 1c) is envisioned as a complementary tool to face the limited absorption bandwidth of the fluorescent actinometers; it exploits a photochemically inert fluorophore exhibiting a broad absorption band to transfer information on light intensity from one wavelength, measured with a fluorescent actinometer as reported above, to another (Supplementary Note 5). Below a value of 0.15 for the absorbance of its solution, the fluorophore emits fluorescence at an intensity proportional to light intensity of the illumination system. By recording the fluorescence emitted when the fluorophore solution is exposed to light at a wavelength (*λ*_1_) where the light intensity (*I*_1_) is known, and a wavelength (*λ*_2_) where the light intensity (*I*_2_) is unknown, the unknown light intensity (*I*_2_) can be determined (Fig. 1c).

As demonstrated in Supplementary Information Section 7.6, 7-hydroxy-9H-(1,3-dichloro-9,9-dimethylacridin-2-one) (DDAO) is a suitable light intensity-transferring fluorophore (Fig. 1d). It is commercially available, absorbs light between 450 and 650 nm and emits fluorescence between 640 and 700 nm in neutral aqueous solutions^33,34^, and its quantum yield of fluorescence does not depend on the excitation wavelength as evidenced by the similarity of its absorption and fluorescence excitation spectra. These features are particularly attractive for light calibration in the orange and red wavelength range where actinometers are scarce and often exhibit a poor quantum yield of fluorescence.

### Measurement of light intensity in fluorescence imaging systems

Accurate measurement of light intensity is important in many fluorescence bioimaging studies (for example, to limit phototoxicity on live biological samples^35^, for quantitative analysis in long timelapse^36^ or ratiometric^37^ studies, or optimal conditions in single molecule localization^38^ and dynamic contrast^16,39^). Hence, we first used fluorescent actinometers for measuring light intensity at the focal plane of multiple wide-field and light-scanning fluorescence imaging systems. Dronpa-2 has been used here for this.

**Fig. 2:**
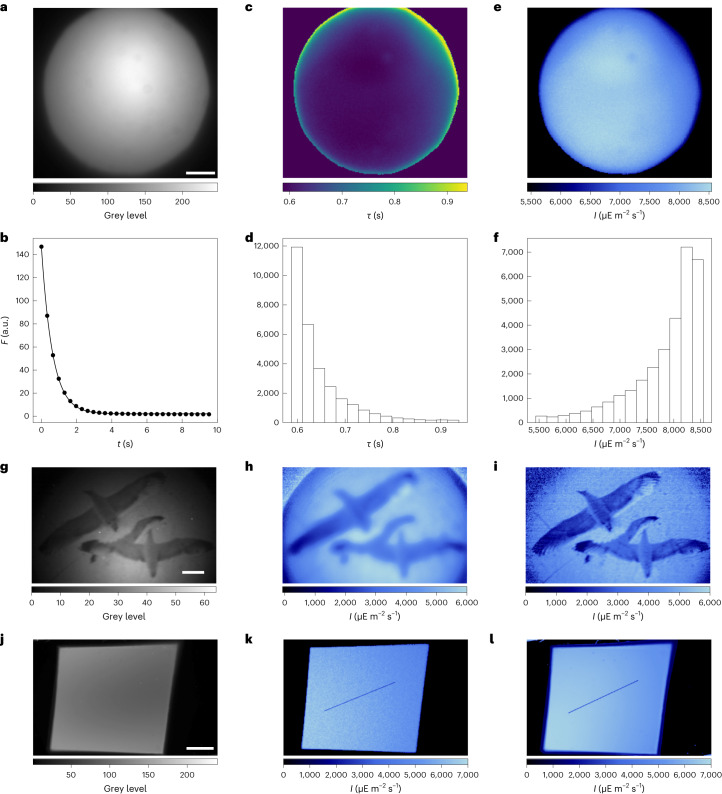
Dronpa-2 for characterization of illumination in wide-field fluorescence imaging. **a**–**i**, Epifluorescence microscopy. **a**, Initial image of the Dronpa-2 solution under homogeneous illumination. **b**, Time fluorescence response (*τ* = 0.63 s), maps (**c**,**e**) and histograms (**d**,**f**) of the characteristic time *τ* (**c**,**d**) and light intensity (**e**,**f**) in the field of view. **g**–**i**, Initial image (**g**) and maps of light intensity (**h**,**i**) of Dronpa-2 in solution (**h**) or in polyacrylamide gel under patterned illumination. **j**–**l**, Macroscopic fluorescence imaging: initial image of the Dronpa-2 solution (**j**) and experimental (**k**) and simulated (**l**) maps of light intensity. The blue line shows the angle of the linear light gradient; the angle between the simulated gradient and the measured one is 3°. **a**–**f**,**h**,**j**–**l**, 10 μM Dronpa-2 solution or 19% polyacrylamide gel in Tris buffer pH 7.4 (50 mM Tris, 150 mM NaCl). Scale bars, 100 μm (**a**,**c**,**e**,**g**,**h**); 3 mm (**j**–**l**). *T* = 293 K. *λ*_exc_ = 470 nm; *λ*_em_ = 550 nm (text and Supplementary Tables 1 and 2). Independent repeats, more than 30 (**a**,**c**,**e**); 3 (**g**–**i**); 15 (**j**,**k**).

**Extended Data Fig. 1:**
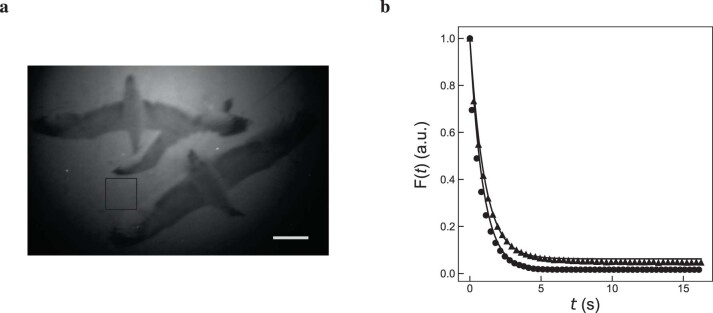
Comparison of the fluorescence decay associated to the photoconversion of Dronpa-2 in solution and in a polyacrylamide gel. **a**: 100×100 pixels region of interest selected for analysis in the image generated in the polyacrylamide gel (scale bar: 100 μm); **b**: Averaged decays of the fluorescence signal over the selected area. Experimental data: disk (solution) triangles (gel); monoexponential fits: solid lines (solution: τ = 0.91 s, polyacrylamide gel: τ = 1.03 s).

We first implemented Dronpa-2 for wide-field epifluorescence microscopy. A Dronpa-2 aqueous solution sandwiched between two glass slides was imaged (Fig. 2a) and subjected to a light jump at 470 nm. Figure 2b shows the relevance of a monoexponential fit of the resulting temporal fluorescence decay over the field of view. We retrieved the map of the characteristic time *τ* at each pixel (Fig. 2c) and built the histogram of the *τ* values (Fig. 2d). The map (Fig. 2e) and the corresponding histogram (Fig. 2f) of light intensity at 470 nm were subsequently computed from using the photoconversion cross section of Dronpa-2 in Table 1. We further exploited a patterned illumination at 470 nm (Fig. 2g) and observed that the map of light intensity was obtained at lower spatial resolution when Dronpa-2 was in solution (Fig. 2h) than when it was embedded in a polyacrylamide gel (Fig. 2i), albeit with identical quantitative information (Extended Data Fig. 1). This result was anticipated from molecular diffusion occurring during Dronpa-2 photoconversion, which generates blurring (Supplementary Information Section 2.1.4).

For quantitative validation, Dronpa-2 was then implemented in fluorescence macroimaging on an original optical setup (Supplementary Information Section 7.3.2), whose illumination is not homogeneous but instead contains a gradient of light intensity across the field of view (Fig. 2j). The sandwiched Dronpa-2 solution was submitted to a light jump at 470 nm to extract the map of the characteristic time *τ*, which was converted into the map of light intensity. This latter map was validated by favorable comparison of the direction of the linear gradient of light intensity, either experimentally observed (Fig. 2k) or computed from an optical simulation (Fig. 2l and Supplementary Information Section 8.1). The lines along the gradient direction differ from each other only by an angle of 3°.

**Fig. 3:**
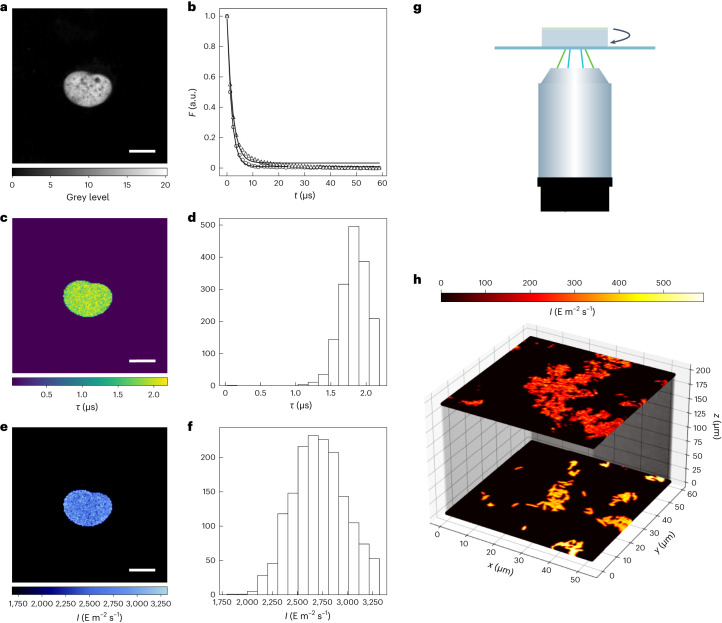
Dronpa-2 for characterization of illumination in confocal microscopy equipped with a pulsed laser in the raster scanning mode. **a**–**f**, Dronpa-2-labeled nucleus of a fixed U-2 OS cell. **a**, Initial image. **b**, Time evolution of the averaged fluorescence over the whole nucleus (circles, experimental data; solid line, monoexponential fit, *τ* = 1.9 µs). The corresponding evolution from a central portion of the overall image of a 10 μM Dronpa-2 solution sandwiched between two glass slides is shown with triangles (*τ* = 2.1 µs). **c**–**f**, Maps (**c**,**e**) and histograms (**d**,**f**) of the characteristic time *τ* (**c**,**d**) and light intensity (**e**,**f**) (Supplementary Table 3). **g**,**h**, Setup (**g**) and map of light intensity retrieved from Dronpa-2-labeled *E. coli* bacteria imaged at the surface or through a 2% agarose pad by changing the sample orientation (**h**). Solvent was Tris buffer pH 7.4 (50 mM Tris, 150 mM NaCl); *T* = 293 K. *λ*_exc_ = 488 nm; 500 nm < *λ*_em_ < 550 nm. Scale bar, 12 μm (**a**,**c**,**e**) (text and Supplementary Tables 1–3). Independent repeats, 4 (**a**,**c**,**e**); 3 (**h**).

**Extended Data Fig. 2:**
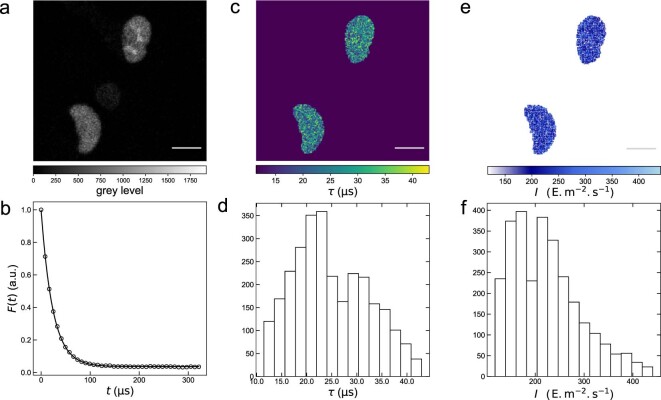
Dronpa-2 for characterization of the spatial distribution of the light intensity of a confocal microscope equipped with a continuous laser. **a, b**: Dronpa-2-labeled nucleus of a fixed U-2 OS cell imaged with a confocal microscope in the raster scanning mode (λ_exc_ = 488 nm; 500 nm ≤ λ_em_ ≤ 600 nm). Initial image (**a**); Time evolution of the averaged fluorescence over the whole nucleus (**b**; circles: experimental data; solid line: monoexponential fit with Eq.(S1). The corresponding evolution from a central portion of the overall image of a 10 μM Dronpa-2 solution sandwiched between two glass-slides is shown with circles); **c–f**: Maps of the characteristic time τ (**c**) and light intensity (**d**), and corresponding histograms (**e,f**; a 3×3 binning is applied to the initial video sequence to improve fitting accuracy). Solvent: Tris buffer pH 7.4 (50 mM Tris, 150 mM NaCl); Pixel size: 0.33 μm; Laser power 10%; *T* = 293 K ; Scale bar: 29 μm; Independent repeats: 4.

A similar protocol was applied in confocal microscopy. A series of images of the Dronpa-2-labeled nucleus of a fixed U-2 OS cell were acquired in raster scanning mode with a pulsed laser at 488 nm (Fig. 3a). The dwell time was used to convert the observed fluorescence decay on the number of frames into a fluorescence drop over time. Figure 3b displays the average drop over a nucleus. It also shows that a Dronpa-2 aqueous solution sandwiched between two glass slides yields a similar kinetic signature on properly restricting analysis to a central portion of the overall image to limit the interference of molecular diffusion on the results (Supplementary Information Section 2.3.3). Maps of the characteristic time *τ* (Fig. 3c) and light intensity (Fig. 3e), and the corresponding histograms (Fig. 3d,f, respectively), were obtained. The mean light intensity retrieved was shown to be consistent with that calculated using the photon flux measured with a power meter, combined with area measurements of the waist of the laser beam, evaluated by raster image correlation spectroscopy^40^ or imaging a fluorescent bead (Supplementary Information Section 8.2). The same series of experiments and validations were performed using a confocal microscope equipped with a continuous, rather than pulsed, laser (Extended Data Fig. 2 and Supplementary Information Sections 2.3.3 and 8.2). The results of which confirmed the ability of this protocol to retrieve light intensity with both modes of laser scanning.

We eventually performed measurements with Dronpa-2-labeled *E. coli* bacteria cells with and without a layer of 2% agarose gel between them and the imaging system (Fig. 3g). Light was applied with the deposited layer facing the objective, and then again after the sample was flipped, such that the light had to cross the gel before reaching the cells. The maps of light intensity for both orientations can be measured in bacteria cells even when buried behind the agarose gel (Fig. 3h), showing that this protocol can measure light intensity, not just at the surface, but in situ, deep within samples.

#### Calibration of setting scales of light intensity

Many optical instruments do not provide information on the absolute light intensity, but rather just the percentage of the maximum possible light that may change with time as the light source ages. Thus, as a second application, we applied the Dronpa-2 and photosynthetic apparatus actinometers for the calibration of the percentage scales of a confocal microscope and a fluorometer, respectively.

**Fig. 4:**
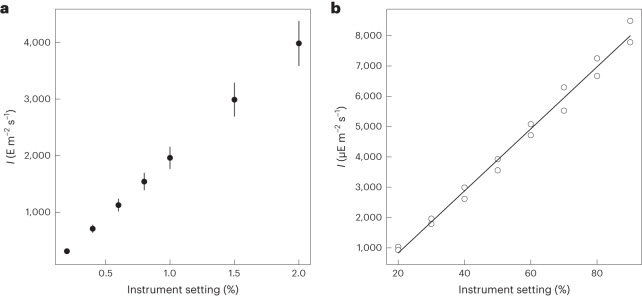
Calibration of optical instruments. **a**,**b**, The dependence of the light intensity at 488 nm (**a**) and 625 nm (**b**) on the percentage scale available on the instruments (confocal microscope with pulsed laser in **a** and fluorometer in **b**) has been established with the Dronpa-2-labeled nucleus of a fixed U-2 OS cell (mean value ± s.d., s.d. are propagated from the error on *σ*(488) (20 repeats)) and photosynthetic apparatus actinometers, respectively. *T* = 293 K (text and Supplementary Tables 1 and 2).

Using a Dronpa-2 aqueous solution sandwiched between glass slides, we measured the light intensity of a confocal microscope equipped with a pulsed laser at 488 nm at different percentages of the maximal laser power, using the protocol detailed above for confocal systems (Fig. 4a). Then we generated the same type of calibration graph (Fig. 4b) for a fluorometer by analyzing the photosynthetic apparatus fluorescence kinetics of a dark-acclimated suspension of microalgae exposed to light at 625 nm (Supplementary Information Sections 2.4.3 and 5.2). These experiments demonstrated that this protocol can allow end users to convert the percentage value indicated on the instrument into absolute light intensity, and to verify linearity in the range of settings investigated.

#### Spectral measurement of light intensity

Photons at different wavelengths drive photoconversion of actinometers to differing degrees. Hence, the spectral characteristics must be considered to obtain accurate measurements of light intensity for light sources, which do not emit at a single wavelength but rather over a spectrum of wavelengths. Accordingly, we extended our protocol to deliver spectral light intensity of nonmonochromatic light sources.

This extended protocol begins with matching the excitation spectrum of the actinometer with the emission spectrum of the light source made available by the manufacturer or measured with a spectrophotometer (Supplementary Information Section 7.2.2). After obtaining the time course of the fluorescence intensity as before, the retrieved characteristic time is used in conjunction with the integral of the action spectrum, the convolution of the normalized emission spectrum of the light source with the excitation spectrum of the actinometer, to quantitatively extract the spectral light intensity (Supplementary Information Section 2.5).

**Fig. 5:**
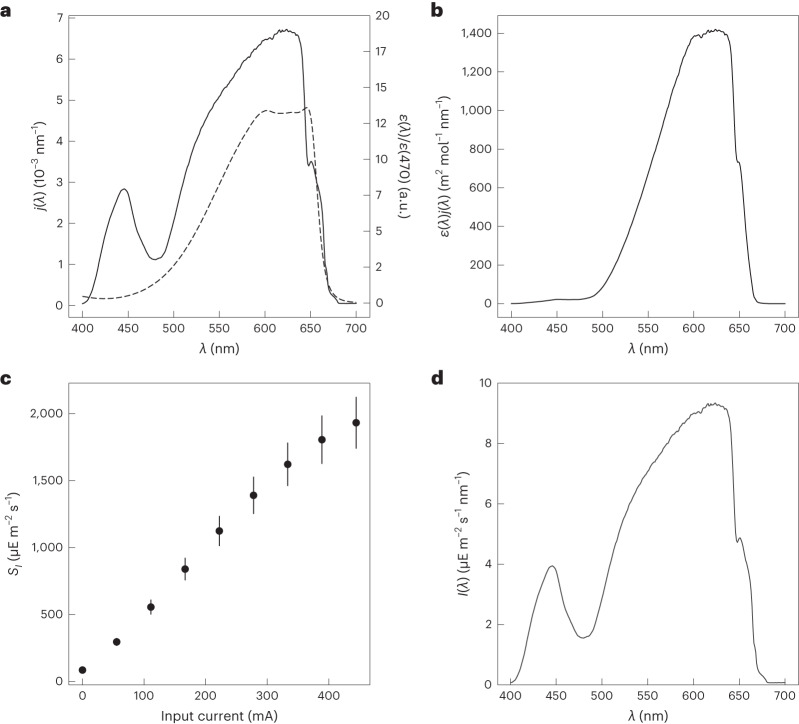
Characterization of the spectral light intensity of a white LED by DDAO-mediated measurement. **a**, Emission spectrum of the white LED normalized by its integral (solid line), absorbance spectrum of DDAO normalized at 470 nm (dashed line). **b**, Action spectrum of the white LED on DDAO. **c**, Dependence of the integral light intensity *S*_*I*_ emitted by the white LED on the input currents as measured with spectral transfer of DDAO fluorescence intensity (mean value ± s.d., s.d. are propagated from the error on *σ*(470) (20 repeats)). **d**, Incident spectral light intensity *I(λ)* of the white LED fed with 277 mA current as retrieved with DDAO (*S*_*I*_ = 1.4 mE m^−2^ s^−1^) (text and Supplementary Table 1).

**Extended Data Fig. 3:**
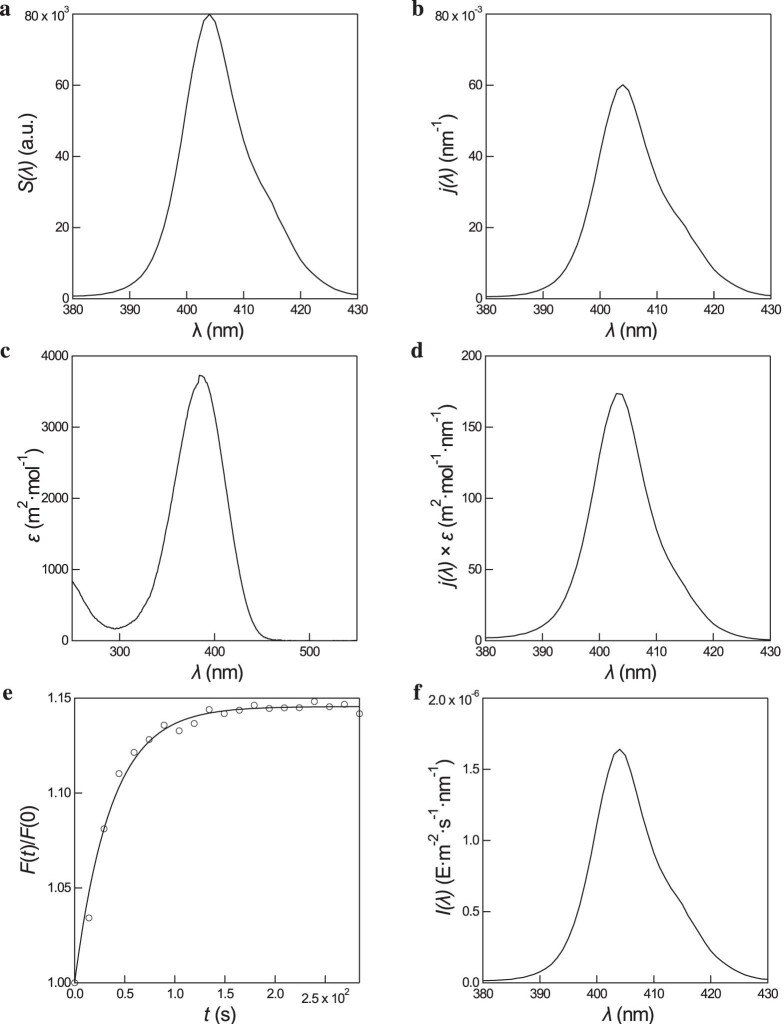
Quantitation of a purple LED emitting at 405 nm. Non-normalized (S(λ); **a**) and normalized (j(λ) = S(λ)/S; **b**) emission spectrum of the LED at 405 nm; **c**: Absorption spectrum of **Nit** ε(λ); **d**: Action spectrum of the LED on **Nit** in ethanol; **e**: Rise of the fluorescence emission of a 12 µM **Nit** and 1 µM **RhB** solution in ethanol at 574 nm as a function of time. Markers: experimental data; solid line: monoexponential fit. From the fit, we retrieved τ = 38.3 s for the relaxation time. T = 293 K; **f**: Scaled emission spectrum of the LED.

**Extended Data Fig. 4:**
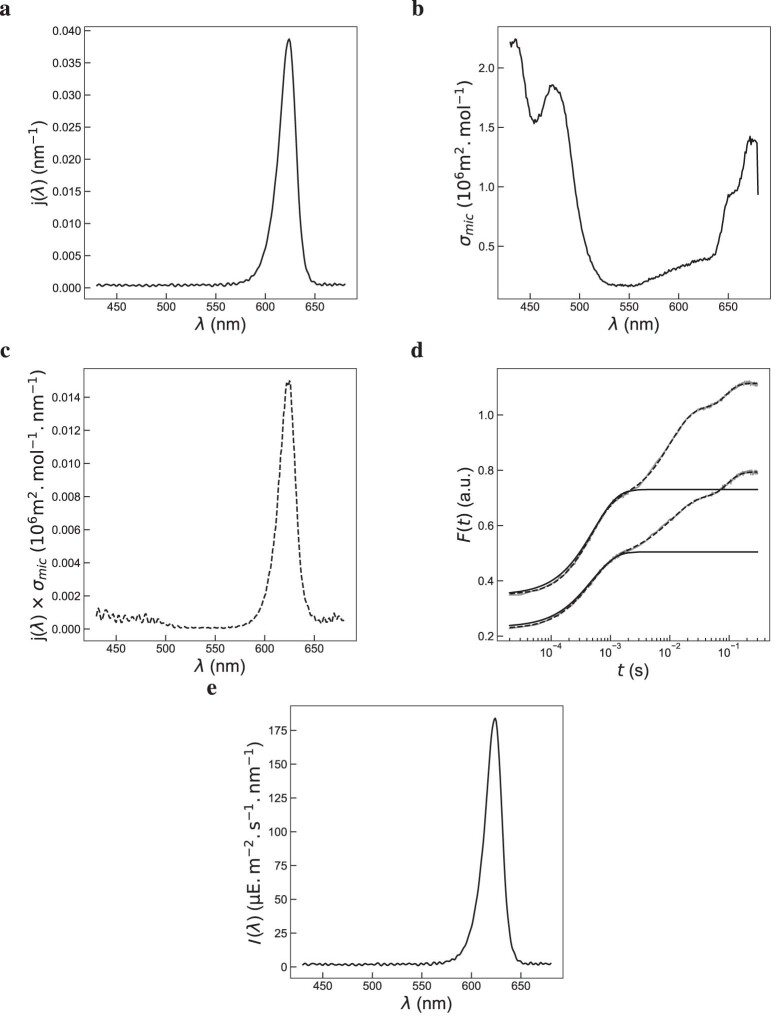
Quantitation of a red-orange LED emitting at 625 nm. **a**: Normalized emission spectrum (j(λ) = S(λ)/S) of the LED at 625 nm; **b**: Scaled fluorescence excitation spectrum σ_mic_(λ) of PA in *Chlamydomonas reinhardtii* (CC 124) in exponential phase in minimal media (λ_em_ = 470 nm); **c**: Action spectrum of the LED at 625 nm on PA in *Chlamydomonas reinhardtii* (CC 124); **d**: Time evolution of the PA fluorescence emission under constant illumination at 625 nm for the power setting 60% on the PSI instrument (2 repeats). The experimental data (grey markers) have been fitted with Eqs.(S3) (dashed lines) and (S4) (solid lines) to retrieve τ = 427 µs and τ = 460 µs for the characteristic time τ associated to the initial step of PA fluorescence rise ; **e**: Scaled emission spectrum of the LED at 625 nm. *T* = 293 K.

**Extended Data Fig. 5:**
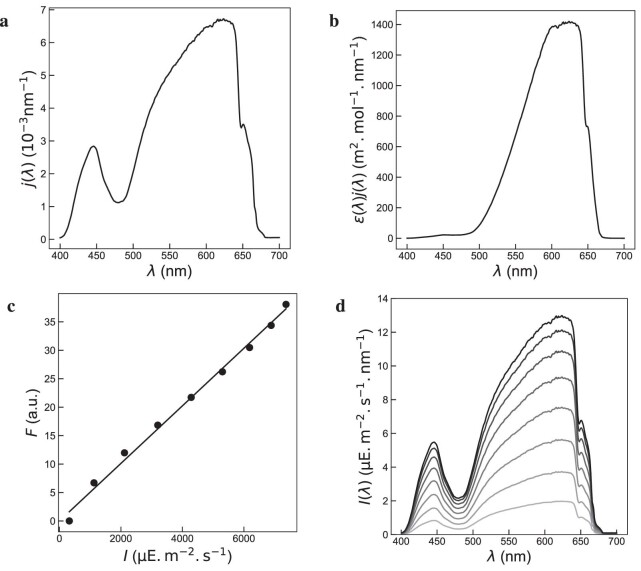
Quantitation of a white LED with DDAO. **a**: Emission spectrum of the white LED normalized by its integral j(λ) = S(λ)/S; **b**: Action spectrum of the white LED on DDAO ϵ(λ)j(λ); **c**: Dependence of the fluorescence level of 10 μM DDAO in aqueous HEPES pH 7.9 buffer (100 mM NaCl, 5 mM NaOH, 10 mM HEPES) sandwiched between two microscope slides F (λ_exc,1_) on the light intensity I (λ_exc,1_) at 470 nm. Squares: experimental data from averaging the fluorescence collected by the camera, solid line: linear fit; **d**: Scaled spectral photon flux of the white LED for current levels feeding the white LED ranging from 55, 111, 166, 222, 277, 333, 388, 444 mA (light to dark). *T* = 293 K.

As preliminary application, we used Nit and photosynthetic apparatus to characterize the illumination from purple and red-orange light emitting diodes (LEDs), respectively (Extended Data Figs. 3 and 4). Then we turned to the more challenging task of measuring the spectral light intensity of a white LED by using DDAO to implement the protocol for transferring information on light intensity (Fig. 5a, Extended Data Fig. 5 and Supplementary Information Sections 2.2, 2.5 and 5.3). We measured the DDAO fluorescence intensity when illuminated by a blue LED previously calibrated with the Dronpa-2 actinometer, and by the white LED. The ratio of the fluorescence signals obtained was used, in combination with the known light intensity of the blue LED, to infer the light intensity of the white LED. The light intensity was then spectrally corrected by using the integral of the normalized action spectrum between the white LED and DDAO (Fig. 5b). This experiment was repeated to deliver the integrated light intensities for a range of LED current settings (Fig. 5c) and the scaled spectral light intensity of the white LED (Fig. 5d).

## Discussion

The measurement of the absolute flux of light at the sample (Working Group 1) and the assessment of the uniformity of illumination (Working Group 3) are key issues of the QUAREP-LiMi community (Quality Assessment and Reproducibility for Instruments and Images in Light Microscopy; https://quarep.org/) made accessible by fluorescent actinometers^6^. After a selection relying on the wavelength to investigate and access to chemical or biological facilities, end users should implement the protocol illustrated in Fig. 1a and detailed in Supplementary Note 2 on using https://github.com/DreamRepo/light_calibration/releases/. Alternatively, they should use the commercially available DDAO to retrieve the light intensity of their desired light source after calibrating another light source with the actinometer they can access. Beyond wide-field fluorescence micro- and/or macroimaging and confocal microscopy explored here, our previous use of quantitative photoconversions suggests the fluorescent actinometers to be relevant on other optical imaging systems (for example, single plane illumination microscopes^41^, fluorescence endoscopes^42^).

Compared to alternative methods implemented here for validation purposes, the fluorescent actinometers benefit from (1) direct access to information sought for, independently on their concentration and without any further measurement (for example, the illuminated surface); (2) measuring light intensities at the surface of samples as well as in their depth, which is difficult to obtain by any other method. Hence irradiance could be calibrated with depth in thick samples by using gels with similar refractive index as biological tissues; (3) a high signal-to-noise ratio from using fluorescence; (4) online access to actinometer properties, (https://chart-studio.plotly.com/~Alienor134/#/) and codes and user-friendly applications for data processing (https://github.com/DreamRepo/light_calibration) and (5) easy and fast (less than 1 hour, from sample preparation to data processing) transfer of know-how to end users. Their kinetics-based protocol is notably robust with respect to parameters that may affect fluorescence from the sample^43^ and the instrument^44^ side^13^. In particular, the quantum yield of fluorescence of the actinometer does not enter into the expression of the characteristic time *τ*. Hence, its temperature and possible wavelength dependence are not detrimental as long as the temperature and wavelength of light excitation remain constant over the measurement. With 20% measurement uncertainty, even a spatial gradient of 20 °C over the distance overcome in time *τ* by the reporting fluorophore, for example rhodamine B^45^, would not affect the result.

However, the time resolution of the fluorescent actinometers is fixed by the kinetics of their photoconversion, which imposes a lower limit on both the measurement duration (still short, 300 ms–30 min) and the ability to analyze time evolving profiles of light intensity. Moreover, the mechanisms underlying their photoconversion often involve multiple steps. Thus, the mechanistic reduction making relevant monoexponentially fitting the time evolution of their fluorescence response to illumination^16^ is reliable only in ranges of light intensity in which the light-driven photoconversion step is constant and rate limiting.

**Extended Data Fig. 6:**
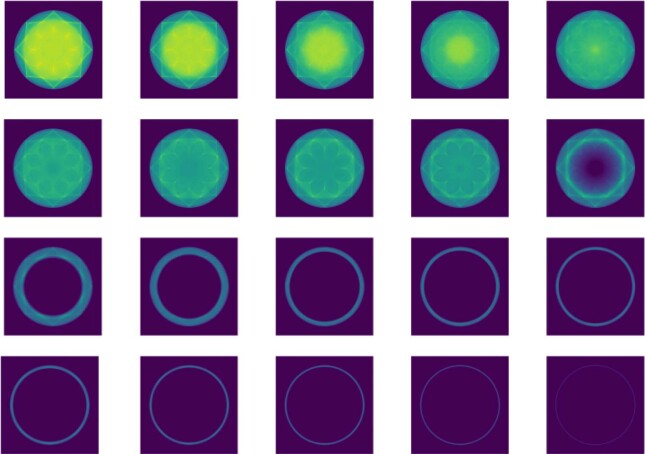
2D images of the absorbed light flux at each of the 20 z-axis locations within the detection volume. The first image corresponds to the **Dronpa-2** solution space adjacent to the bottom of the Petri dish, and the following images, going left to right, and down, in a raster fashion, correspond to image slices progressively closer to the fluid surface. Since this work did not consider the absolute light intensity, but rather the distribution, a color scale is omitted.

**Extended Data Fig. 7:**
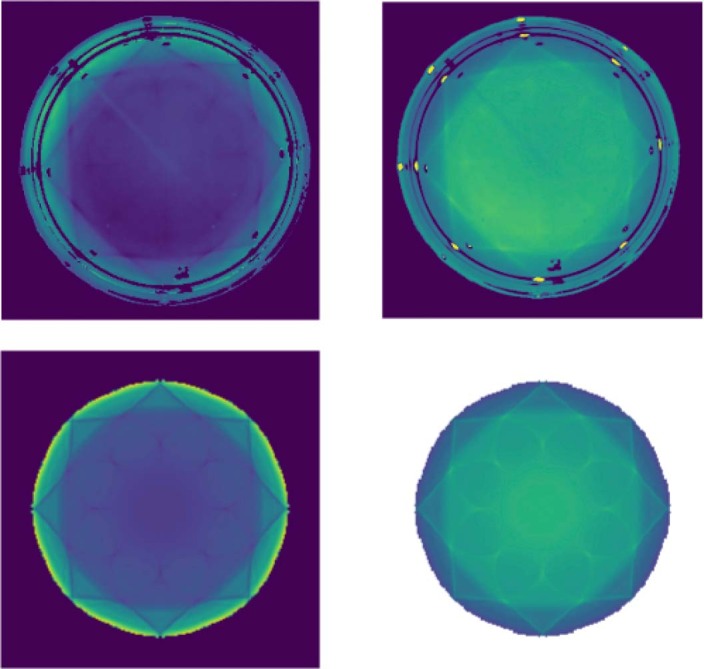
Experimental and simulated intensity maps in a 3D sample. τ (left) and light intensity (right) maps of the experimental (top) and simulation (bottom) situation. Since this work did not consider the absolute light intensity, but rather the distribution, a color scale is omitted.

Eventually, two issues arise for characterizing spatially inhomogeneous light profiles: (1) as illustrated by imaging a Petri dish containing a Dronpa-2 solution illuminated by a surrounding radial array of LEDs (Supplementary Note 9), a 3D-inhomogeneous light profile cannot be retrieved from the 2D-map of the characteristic time *τ*. Yet, one can still retrieve a useful information on the light intensity averaged over the sample thickness (Extended Data Figs 6 and 7); (2) as shown in Fig. 2g–i, molecular motion introduces blurring in the retrieved 2D light profile. However, this phenomenon is marginal as long as molecular diffusion is spatially limited at the *τ* time scale, which can be reached by using a medium in which molecular diffusion is reduced. A polyacrylamide gel is relevant for the water-soluble fluorescent actinometers. Immobilization of the synthetic fluorescent actinometers would require developments, which have not been covered during the present study.

We envision that such organic dye- and fluorescent protein-based actinometry will improve our understanding of how light dose effects the health and viability of biological specimens, and think that measuring and reporting light dose experienced by the sample should become commonplace for improving reporting and reproducibility in microscopy.

## Methods

The Supplementary Information starts with a list, which indicates the (sub)sections associated with the supplementary elements of the main text, as well as details on statistical parameters and image processing. It is then divided in two parts: (1) sections 1–3 are dedicated to end users, who want to directly implement the reported fluorescent actinometers; and (2) sections 4–9 contain more advanced information as well as the complete validation of the reported fluorescent actinometers. Codes and metadata for the figures in the main text can be found online, as well as user-friendly tools and how to use them: https://github.com/DreamRepo/light_calibration and in a mirror Zenodo repository (10.5281/zenodo.7966573).

### Syntheses

#### Cin

Bromine (2.32 g, 0.74 ml, 14.5 mmol; 2 eq.) was added dropwise to a solution of 2,4-dihydroxybenzaldehyde (1.00 g, 7.25 mmol) in acetic acid (10 ml) over 30 min at room temperature. The resulting mixture was vigorously stirred for 2 h at room temperature. After addition of water (20 ml), the precipitate was filtered, washed with water and dried. 3,5-dibromo-2,4-dihydroxybenzaldehyde was obtained as pale orange crystals after recrystallization of the crude residue in ethanol (1.20 g, 55% yield). m.p: 200 °C; ^1^H-nuclear magnetic resonance (NMR) (ppm, 250 MHz, CDCl_3_, 298 K) δ 9.68 (s, 1 H), 7.70 (s, 1 H), 6.60 (s, 1 H); ^13^C-NMR (ppm, 62.8 MHz, CDCl_3_, 298 K) δ 193.5, 158.6, 157.1, 135.6, 114.9, 99.9 and 97.8.

Ethyl bromoacetate (5.75 g, 34.5 mmol) was added to a solution of triphenylphosphine (10.0 g, 38 mmol; 1.15 eq) in toluene (40 ml). The mixture was vigorously refluxed for 10 h under stirring. The white precipitate was filtered off, washed with toluene and dried. The addition at 5 °C of 1 M NaOH (50 ml) to a solution of white solid (10 g) in water (200 ml) gave a white and gummy solid that was filtered, washed with water and dried to yield 1-carboxymethylidene triphenyl phosphorane as a white solid (8.0 g, 60% yield).

A mixture of 3,5-dibromo-2,4-dihydroxybenzaldehyde and 1-carboethoxymethylidene triphenyl phosphorane (1.5 eq) in toluene (10 ml for 1 mmol of aldehyde) was heated at 60 °C under argon on protecting from light. The course of the reaction was followed by cyclohexane and AcOEt. After 2 to 4 h, the reaction was completed. After cooling to room temperature, toluene was removed in a vacuum. The crude residue was purified by flash chromatography on silica gel (mixtures of ethyl acetate and cyclohexane as eluent) to give Cin in 40 to 60% yield^15^. m.p. 118–118.5 °C; ^1^H-NMR (ppm, 250 MHz, CDCl_3_, 298 K) δ 7.81 (d, 1 H, *J* = 16.1 Hz), 7.60 (s, 1 H), 6.47 (d, 1 H, *J* = 16.1 Hz), 6.07 (bs, 2 H), 4.22 (q, 2 H, *J* = 7.0 Hz), 1.33 (t, 3 H, *J* = 7.0 Hz); ^13^C-NMR (ppm, 62.8 MHz, CD_3_COCD_3_, 298 K) δ 167.3, 154.2, 153.7, 138.7, 131.6, 118.8, 117.8, 102.1, 101.8, 60.7, 14.6. Elemental analysis: (%) for C_11_H_10_O_4_Br_2_(365.9): C 36.10, H 2.75; found: C 36.06, H 2.63; mass spectrometry: MS (CI, CH_4_): *m/z* 367 [M + 1]; MS (CI, CH_4_, HR): *m/z* 364.9024, 366.9006 and 368.8992 (calculated mass for C_11_H_10_O_4_Br_2_: 364.9024, 366.9004 and 368.8985).

#### Nit

4-(Diethylamino)benzaldehyde (1.5 g, 8.5 mmol) and phenylhydroxylamine (0.925 g, 8.5 mmol) were stirred in glacial acetic acid (8 ml) at room temperature for 2 h. The reaction mixture was then poured into water and extracted with ether. The ether extracts washed with saturated aqueous sodium bicarbonate and with brine, dried on sodium sulfate and concentrated under a vacuum. After recrystallization in cyclohexane and toluene, Nit^24^ was obtained as orange needles (1.70 g, 6.4 mmol, 75%). ^1^H-NMR (CDCl_3_, 300 MHz) δ 8.30 (d, 2H, *J* = 9 Hz); 7.79–7.76 (m, 3H), 7.48–7.37 (m, 3H), 6.71 (d, 2H, *J* = 9 Hz), 3.43 (q, 4H, *J* = 7 Hz), 1.22 (t, 6H, *J* = 7 Hz).

#### DASA

3-Methyl-1-(4-sulfophenyl)-5-pyrazolone (1.97 mmol, 500 mg) was suspended in water (5 ml) and carefully neutralized to pH 7 with 1 M sodium bicarbonate. To this, furfural (1.97 mmol, 189 mg) was added and the reaction mixture stirred at 20 °C for 16 h. The solvent was then evaporated under reduced pressure to give the sodium 4-(4-(furan-2-ylmethylene)-3-methyl-5-oxo-4,5-dihydro-1H-pyrazol-1-yl)benzenesulfonate intermediate (I) as a red solid (494 mg, 76% yield). The product is a mixture of *Z* and *E* isomers (2:1). ^1^H-NMR (300 MHz, deuterium oxide) δ 8.09 (d, *J* = 3.8 Hz, 1H), 7.83 (d, *J* = 1.6 Hz, 1H), 7.80–7.61 (m, 7H), 7.33 (s, 1H), 7.18 (d, *J* = 3.5 Hz, 0.5H), 7.16 (s, 0.5H), 6.68 (dd, *J* = 3.7, 1.6 Hz, 1H), 6.61 (dd, *J* = 3.6, 1.7 Hz, 0.5H), 2.32 (s, 1.5H), 2.11 (s, 3H). ^13^C-NMR (75 MHz, DMSO) δ 164.32, 161.54, 150.90, 150.76, 150.26, 150.11, 149.51, 148.12, 144.35, 138.07, 130.29, 127.36, 127.22, 126.24, 126.18, 124.65, 121.29, 120.10, 116.91, 116.62, 114.84, 114.45, 17.39, 12.64. High-resolution mass spectrometry (electrospray ionization+) *m/z* calculated. For [C_15_H_11_N_2_O_5_S] [M–H+]: 331.039; found, 331.040.

Compound I (0.565 mmol, 200 mg) and indoline (0.565 mmol, 67 mg) were dissolved in methanol (2 ml) and stirred at 20 °C for 1 h. Then this solution was diluted in 10 ml of ethyl ether, the precipitate filtered and washed with 3 × 5 ml of ethyl ether to yield DASA^17^ as a dark blue powder (165 mg, 62% yield). ^1^H-NMR (300 MHz, deuterium oxide) δ 8.01 (dd, *J* = 6.0, 1.9 Hz, 1H), 7.95 (d, *J* = 8.4 Hz, 2H), 7.76 (d, *J* = 8.6 Hz, 2H), 7.27 (d, *J* = 7.6 Hz, 1H), 7.05 (t, *J* = 7.6 Hz, 1H), 6.86 (t, *J* = 7.3 Hz, 1H), 6.67–6.56 (m, 2H), 5.37 (d, *J* = 1.5 Hz, 1H), 3.76 (d, *J* = 3.2 Hz, 1H), 3.48–3.37 (m, 2H), 3.15–2.97 (m, 2H), 1.80 (s, 3H). ^13^C-NMR (75 MHz, DMSO) δ 202.51, 163.24, 162.70, 150.52, 149.47, 144.27, 137.06, 134.63, 129.83, 126.99, 126.28, 124.53, 117.63, 116.97, 107.38, 103.62, 61.04, 47.83, 43.50, 27.78 and 10.58. Additional small peaks due to the presence of the keto isomer on the pyrazole. High-resolution mass spectrometry (electrospray ionization−) *m/z* calculated. For [C_23_H_20_N_3_O_5_S] [M−]: 450.11; found, 450.11.

### Production of Dronpa-2-containing samples

#### Plasmids

The plasmids for bacterial expression of Dronpa-2 carrying an N-terminal hexahistidine tag and for mammalian expression of Dronpa-2 fused at the C terminal of the histone H2B (H2B-Dronpa-2) have been previously described in refs. ^39,41^.

#### Production of Dronpa-2-labeled *E. coli*

*E. coli* cells from the TOP10 strain were transformed with the Dronpa-2 plasmid by electroporation. The transformed *E. coli* cells were grown at 37 °C in Luria Bertani medium. When the optical density at 600 nm reached 0.2, expression was induced by addition of isopropyl β-d-1-thio-galactopyranoside (IPTG) to a final concentration of 1 mM. After 4 h of expression at 30 °C, 1 ml aliquots were taken and cells were centrifuged at 8,000 rpm for 5 min. After centrifugation, the supernatant was removed and the *E. coli* cells were washed once with 1 ml of PBS (pH 7.4, 50 mM sodium phosphate, 150 mM NaCl) and then resuspended in 250 μl of PBS buffer.

This suspension of *E. coli* was used to prepare a monolayer of bacterial cells deposited on an agarose pad as follows: 125 μl of a 2% pad of low-melting agarose in PBS was sandwiched between two circular glass slides separated by 250 μm by spacers (Gene Frames AB0578; Thermo Scientific). After the agarose became solid, the top cover slide was removed and 2 μl of the bacterial suspension was deposited on the surface of the agar pad. After 15 min, the top cover slide was replaced to seal the sample.

#### Production and purification of Dronpa-2

The Dronpa-2 plasmid with an N-terminal hexahistidine tag was transformed in *E. coli* BL21 strain. Cells were grown in Terrific Broth at 37 °C. The expression was induced at 30 °C or 16 °C by addition of IPTG to a final concentration of 1 mM at optical density at 600 nm of 0.6. The cells were collected after 16 h of expression and lysed by sonication in lysis buffer (50 mM PBS with 150 mM NaCl at pH 7.4, 1 mg ml^−1^ DNAse, 5 mM MgCl_2_ and 1 mM phenylmethylsulfonyl fluoride, and a cocktail of protease inhibitors (Sigma Aldrich; catalog no. S8830)). After lysis, the mixture was incubated on ice for 2 h for DNA digestion. The insoluble material was removed by centrifugation and the supernatant was incubated overnight with Ni-NTA agarose beads (Thermo Fisher) at 4 °C in a rotator-mixer. The protein loaded Ni-NTA column was washed with 20 column volumes of N1 buffer (50 mM PBS, 150 mM NaCl, 20 mM imidazole, pH 7.4) and 5 column volumes of N2 buffer (50 mM PBS, 150 mM NaCl, 40 mM imidazole, pH 7.4). The bound protein was eluted with N3 buffer (50 mM PBS, 150 mM NaCl, 0,5 M imidazole, pH 7.4). The protein fractions were eventually dialyzed with cassette Slide-A-Lyzer Dialysis Cassettes (Thermo Fisher) against 50 mM PBS, 150 mM NaCl pH 7.4.

#### Production of Dronpa-2-labeled mammalian cells

U-2 OS cells were grown at 37 °C in 5% CO_2_ in air atmosphere in McCoy’s 5A Medium complemented with 10% fetal bovine serum. Cells were transiently transfected with Genejuice (Merck) according to the manufacturer’s protocol then washed with Dulbecco’s phosphate buffered saline (2.7 mM KCl, 138 mM NaCl, 1.5 mM KH_2_PO_4_, 8.1 mM Na_2_HPO_4_, Thermo Fisher) and fixed with 2% paraformaldehyde solution in Dulbecco’s phosphate buffered saline.

### Production of photosynthetic apparatus-containing samples

The algae strain used were wild type CC124 and WT4 of *Chlamydomonas reinhardtii* provided by the Institut de Biologie Physico-Chimique (http://www.ibpc.fr/UMR7141/en/home/). The algae were grown in heterotrophic media TAP (https://www.chlamycollection.org/methods/media-recipes/tap-and-tris-minimal/) under constant 5–10 μE m^−2^ s^−1^ white LED and agitation at 25 °C. The population was diluted to one-tenth the day before the experiment to ensure that the culture observed is in exponential phase.

### Reporting summary

Further information on research design is available in the Nature Portfolio Reporting Summary linked to this article.

## Online content

Any methods, additional references, Nature Portfolio reporting summaries, source data, extended data, supplementary information, acknowledgements, peer review information; details of author contributions and competing interests; and statements of data and code availability are available at 10.1038/s41592-023-02063-y.

### Supplementary information

Supplementary Information Supplementary Notes 1–9 (Materials, Protocols of implementation of the fluorescent systems for measuring light intensity, Glossary, Conversion of energy units, Theoretical derivation of the expressions for retrieving light intensity, Assumption of monochromatic versus polychromatic light for retrieving cross sections of light absorption, Characterization of the fluorescent systems for measuring light intensity, Validation of the extraction of the light intensity in fluorescence imaging, Measurement of light intensity from a LED array), Figs. 1–58 and Tables 1–7.

Reporting Summary

Peer Review File

## Data Availability

The online repository contains representative raw data files corresponding to the methods described. The datasets generated and/or analyzed during the current study and that are not in the online repository due to their profuse nature are available from the corresponding author on request. Absorption and emission spectra of the actinometers: https://github.com/DreamRepo/light_calibration/tree/main/spectra_plotly and https://chart-studio.plotly.com/~Alienor134/#/. Metadata for the video acquisitions used to produce the main text figures: https://github.com/DreamRepo/light_calibration/tree/main/imaging_metadata. Simulations of the illumination used in Fig. 2k,l: https://github.com/DreamRepo/light_calibration/tree/main/Macroscope. Simulation of 3D illumination pattern and comparison with 2D imaging: https://github.com/DreamRepo/light_calibration/tree/main/LED%20Array. Representative data: https://github.com/DreamRepo/light_calibration/blob/main/data.

## Code availability

The codes that require several steps to reproduce the data analysis have been made available online: https://github.com/DreamRepo/light_calibration and in a mirror Zenodo repository (https://doi.org/10.281/zenodo.7665939). Application to process a fluorescence evolution curve: https://github.com/DreamRepo/light_calibration/releases/tag/review. Wavelength transfer using DDAO: https://github.com/DreamRepo/light_calibration/tree/main/DDAO. Light intensity in the field of view (Fig. 2): jupyter notebook: https://github.com/DreamRepo/light_calibration/blob/main/notebooks/Dronpa2_video.ipynb, instructions: https://github.com/DreamRepo/light_calibration/tree/main. Implementation of the fitting algorithm for the fluorescence induction of microalgae: https://github.com/DreamRepo/light_calibration/blob/main/notebooks/PA_OJIP_rise_fit.ipynb, data: https://github.com/DreamRepo/light_calibration/blob/main/data/2022-01-17_13_33_ojip_curve_363_blue.csv. The python codes can be executed without preliminary installation with Binder: https://mybinder.org/v2/gh/DreamRepo/light_calibration/HEAD.
