# Supplementary material for: Fluorescence to measure light intensity
Source: Nat Methods. 2023 Nov 23;20(12):1930–8. doi: 10.1038/s41592-023-02063-y (PMC10703675; doi:10.1038/s41592-023-02063-y)
Supplement: Supplementary file 1 — Supplementary Notes 1–9 (Materials, Protocols of implementation of the fluorescent systems for measuring light intensity, Glossary, Conversion of energy units, Theoretical derivation of the expressions for retrieving light intensity, Assumption of monochromatic versus polychromatic light for retrieving cross sections of light absorption, Characterization of the fluorescent systems for measuring light intensity, Validation of the extraction of the light intensity in fluorescence imaging, Measurement of light intensity from a LED array), Figs. 1–58 and Tables 1–7. [file 41592_2023_2063_MOESM1_ESM.pdf]

nature methods

Article https://doi.org/10.1038/s41592-023-02063-y

Fluorescence to measure light intensity

In the format provided by the authors and unedited

The Supporting Information is divided in two parts:

- The three sections 1–3 are dedicated to end-users, who are willing to implement the reported fluorescent actinometers;
- The six sections 4–9 contain more advanced information as well as the complete validation of the reported fluorescent actinometers.

The following list indicates the references of the sections and subsections associated with calls in the Main Text:

- Conversion of units of light intensity: Section 4;
- Theoretical derivation of the time evolution of the fluorescence signal associated with the photoconversion of the fluorescent actinometers: Section 5, subsections 5.1 and 5.2;
- Data processing for retrieving the characteristic time τ from the time evolution of the fluorescence signal associated with the photoconversion of the fluorescent actinometers: Theoretical rational in Section 5, subsections 5.1 and 5.2, and Fitting protocol in subsection 2.1;
- Range of light intensity I in which its derivation obeys the simple equation given in Figure 1a of the Main Text: Section 5, subsections 5.1.1 and 5.2.1, and Eq.S62;
- General estimate of the achievable measurement uncertainty on light intensity I: Section 7
- Information on the fluorescent actinometers:
  - **Cin**: Protocol for implementation: Subsection 2.1.3, Validation: Subsection 7.4.1;
  - **Nit**: Protocol for implementation: Subsection 2.1.3, Validation: Subsection 7.5.1;
  - **DASA**: Protocol for implementation: Subsection 2.1.3, Validation: Subsection 7.4.3;
  - **Dronpa-2**: Protocol for implementation: Subsection 2.1.3, Validation: Subsection 7.4.2;
  - **PA**: Protocol for implementation: Subsection 2.1.3, Validation: Subsection 7.4.4;
- Reporting with fluorescence on the time evolution of the absorbance (which follows reaction extent) at the excitation wavelength by the inner filter effect: Subsection 5.1.2;
- A second protocol to measure light intensity : Subsection 5.3.1;
- **DDAO** as a suitable photochemically inert light intensity-transferring fluorophore: Subsection 7.6.3;
- Molecular diffusion generates blurring when it occurs at the time scale of **Dronpa-2** photoconversion: Subsection 2.1.4;

- Original optical setup for fluorescence macroimaging: Subsection 7.3.2;
- Optical simulation of illumination in the original optical setup for fluorescence macroimaging: Subsection 8.1;
- Interference of molecular diffusion of the fluorescent actinometer in confocal microscopy: Subsection 2.3.3;
- Consistency of the light intensities extracted in confocal microscopy with a fluorescent actinometer and with a power meter: Subsection 8.2;
- Experiments and validations performed using a confocal microscope equipped with a continuous laser: Subsections 2.3.3 and 8.2;
- Analysis of the **PA** fluorescence kinetics of a dark-acclimated suspension of microalgae exposed to its illumination light at 625 nm: Subsections 5.2 and 2.4.3;
- Measurement of the emission spectrum of a light source with a spectrophotometer: Subsection 7.2.2;
- Retrieval of information on a light source in the form of spectral light intensity: Subsections: 2.5;
- Characterization of the illumination from purple and red-orange Light Emitting Diodes (LEDs): Subsections: 2.5;
- Retrieval of information on a white LED in the form of spectral light intensity by using **DDAO**: Subsections: 2.2, 2.5, and 5.3;
- Imaging of a Petri dish containing a solution of **Dronpa-2** being illuminated by a surrounding radial array of LEDs: Section: 9.

Table S1 indicates the subsections providing the theoretical background (Theory), the sample preparation (Sample), the instrumental description (Instrument), the implementation protocol (Protocol), and the protocol validation (Validation) associated to the Figures of the Main Text.

Table S2 complement the metadata, statistical parameter and image processing of the figures of the Main Text. The metadata can be found in the "imaging_metadata" folder of the online repository (doi.org/10.5281/zenodo.7966573). Precision on image analysis: since the exponential fitting parameters are not restricted by boundaries, the fits can output outliers. Effects of misfits can be seen on the borders of Figure 2i. To limit the outlier influence on the display of the content of interest, a uniform mask computed with the library *skimage.filters.thresholding*[1] from the grey-level image is used on the tau maps and intensity maps to avoid fitting noise on the dark areas and reduce computation costs. The unmasked values are clipped between specified histogram quantiles of tau maps and intensity maps to remove misfit-related outliers. Note that a Gaussian smoothing (*skimage.filter.gaussan*[1]($\sigma = 1$)) was used after processing of the intensity map to build Figure 3h.

Table S1: Supporting Information related to the Figures of the Main Text.

	**Theory**	**Sample**	**Instrument**	**Protocol**	**Validation**
Figure 1a,b	5.1–5.2	1	7.2–7.3	2.1	7.4
Figure 1c,d	5.3	2.2	7.2–7.3	2.2	7.6.3
Figure 2a–i	5.2	5.2.1	7.3.1	2.3.3	7.4.2
Figure 2j–l	5.2	5.2.1	S25	8.1	8.1
Figure 3	S90–5.2	5.2.1	7.3.3	MT–2.3.3	8.2.3
Figure 4a	S90	5.2.1	7.3.3	2.3.3	8.2.2
Figure 4b	7.4.4	–[a]	7.3.1–7.3.4	2.4.3	7.4.4
figure 5a–c	5.3	2.2	7.3.1	2.5	7.6.3

[a] See Methods in the Main Text.

Table S2: Complementary data for the Figures in the Main Text

Figure	Metadata	τ	$CI_{95\%}$	threshold	quantile clip
1b	refer to table S1	**Nit**: 157 s<br>**Cin**: 2160 s<br>**Dronpa-2**: 15 s<br>**DASA**: 51 s<br>**PA**: 71 µs	± 4.2%[a]<br>± 1.5%[a]<br>± 1.5%[a]<br>± 4.1%[a]<br>± 9.0%[b]	N/A	N/A
2a-i	metadata_epifluo	0.63 s	± 0.9%	minimum	[0.01, 0.995]
2j-l	metadata_macroscope	N/A	N/A	triangle	[0.01, 0.995]
3a-f	metadata_pulse_confocal<br>metadata_confocal_cell	nucleus: 1.87 µs<br>liquid: 2.08 µs	± 0.5 %[b]<br>± 6.1%[b]	li	[0.01, 0.95]
3h	metadata_pulse_confocal<br>metadata_confocal_bacteria	N/A	N/A	li	[0.01, 0.95]
4a	metadata_pulse_confocal<br>metadata_confocal_calib	[1.26, 16.29] µs	max: ± 0.6%[b]	li	N/A
4b	metadata_OJIP_PSI<br>FL6000 fluorometer PSI	[0.26,2.3] ms	max: ± 11%[b]	N/A	N/A

[a] The confidence interval was computed with Igor Pro.
[b] The confidence interval was computed with the python library lmfit.[2]

Table S3: Complements to the legends of the Figures in the Main Text.

Figure	Complements
Figure 1b	*Five fluorescent actinometers covering the UV-Vis range in action.* Monoexponential fit of the time evolution of the normalized fluorescence signal $F(t)/F(0)$ provides τ (in s: 157, 2160, 15, 51, 71.10^{-6}), which yields I in μE.m^{-2}.s^{-1} (W.m^{-2}): 5.3 (1.7) at 380 nm,2.5 (0.7) at 405 nm, 270 (69) at 480 nm, 15 (2.9) at 632 nm, 7800 (2000) at 470 nm with **Nit**, **Cin**, **Dronpa-2**, **DASA**, and **PA** respectively.
Figure 3c–f	Maps of the characteristic time τ (**c**) and light intensity (**e**), and corresponding histograms (**d**,**f**; a 3×3 binning is applied to the initial video sequence to improve fitting accuracy).

Contents

1 Supplementary Note 1: Materials **10**

1.1 Characterization of the commercially available fluorophores . . . 10

1.2 Syntheses . . . 11

1.2.1 *(E)*-3-(3,5-Dibromo-2,4-dihydroxyphenyl) acrylic acid ethyl ester (**Cin**)[3] . . . 11

1.2.2 α-(4- Diethylamino)phenyl)-N-phenylnitrone (**Nit**)[4] . . . 11

1.2.3 Sodium 4-(4-((2Z,4E)-2-hydroxy-5-(indolin-1-yl)penta-2,4-dien-1-ylidene)-3-methyl-5-oxo-4,5-dihydro-1H-pyra-zol-1-yl)benzenesulfonate (**DASA**)[5] . . . 12

2 Supplementary Note 2: Protocols of implementation of the fluorescent systems for measuring light intensity **12**

2.1 Measurements of light intensity with the fluorescent actinometers . . . 12

2.1.1 Reagents . . . 12

2.1.2 Equipments . . . 12

2.1.3 Procedure . . . 13

Generic protocol (Figure S6) . . . 13

Description of five fluorescent actinometers covering the entire UV-Vis wavelength range . . . 14

Specific protocols . . . 15

Cin to measure light intensity in the 350–420 nm range . . . 15

Nit to measure light intensity in the 320–430 nm range . . . 17

Dronpa-2 to measure light intensity in the 440–500 nm range . . . 18

DASA to measure light intensity in the 530–670 nm range . . . 19

PA to measure light intensity in the 400–650 nm range . . . 21

2.1.4 Troubleshooting . . . 22

General . . . 22

Specific . . . 23

DASA . . . 24

PA . . . 24

2.1.5 Time taken . . . 24

2.1.6 Anticipated results . . . 24

2.1.7 References . . . 24

2.2 A reporting fluorophore to transfer information on light intensity from one wavelength to another . . . 25

2.2.1 Reagents . . . 26

2.2.2 Equipments . . . 26

2.2.3 Procedure . . . 27
2.2.4 Troubleshooting . . . 28
2.2.5 Time taken . . . 28
2.2.6 Anticipated results . . . 28
2.2.7 References . . . 28
2.3 Measurement of the light intensity at 488 nm at the focal plane of fluorescence microscopes . . . 28
2.3.1 Reagents . . . 28
2.3.2 Equipments . . . 28
2.3.3 Procedure . . . 29
Epifluorescence microscope . . . 29
Confocal microscope equipped with a continuous 488 nm laser . . . 29
Confocal microscope equipped with a pulsed 488 nm laser . . . 30
2.3.4 Troubleshooting . . . 31
General . . . 31
Epifluorescence microscope . . . 31
Confocal microscopy . . . 31
2.3.5 Time taken . . . 32
2.3.6 Anticipated results . . . 33
2.3.7 References . . . 33
2.4 Quantitation of non-monochromatic light sources with a fluorescent actinometer . . . 33
2.4.1 Reagents . . . 33
2.4.2 Equipments . . . 34
2.4.3 Procedure . . . 34
Quantitation of a purple LED emitting at 405 nm . . . 34
Quantitation of a red-orange LED emitting at 625 nm . . . 35
2.4.4 Troubleshooting . . . 36
2.4.5 Time taken . . . 36
2.4.6 Anticipated results . . . 36
2.4.7 References . . . 37
2.5 Quantitation of non-monochromatic light sources with a photochemically inert fluorophore . . . 38
2.5.1 Reagents . . . 38
2.5.2 Equipments . . . 38
2.5.3 Procedure . . . 39
2.5.4 Troubleshooting . . . 40

2.5.5 Time taken . . . 40
2.5.6 Anticipated results . . . 41
2.5.7 References . . . 41

3 Supplementary Note 3: Glossary 41

4 Supplementary Note 4: Conversion of energy units 43

5 Supplementary Note 5: Theoretical derivation of the expressions for retrieving light intensity 43

5.1 First protocol: Kinetic analysis of an actinometer engaged in an irreversible photochemical reaction . . . 43
5.1.1 The model . . . 43
5.1.2 Light-jump experiments with monochromatic illumination . . . 44
Expression of the concentrations . . . 44
Expression of the absorbance . . . 46
Expression of the fluorescence . . . 46
Intrinsic fluorescence reporting . . . 47
Extrinsic fluorescence reporting . . . 47
5.1.3 Light-jump experiments with non-monochromatic illumination . . . 48
5.2 First protocol: Kinetic analysis of an actinometer engaged in a reversible photochemical reaction . . . 49
5.2.1 The model . . . 49
5.2.2 Light-jump experiments . . . 50
Expression of the concentrations . . . 50
Expression of the absorbance . . . 51
Expression of the fluorescence . . . 51
Intrinsic fluorescence reporting . . . 52
Extrinsic fluorescence reporting . . . 52
5.3 Second protocol: Transfer of information on light intensity from a wavelength to another with a photochemically inert fluorophore . . . 52
5.3.1 Illumination with monochromatic illumination . . . 52
5.3.2 Illumination with non-monochromatic illumination . . . 53

6 Supplementary Note 6: Assumption of monochromatic vs polychromatic light for retrieving cross sections of light absorption 54

7 Supplementary Note 7: Characterization of the fluorescent systems for measuring light intensity 55

7.1 Materials . . . 56

7.2 UV/Vis absorption and fluorescence spectrometers 56
7.2.1 Instruments 56
7.2.2 Light sources 57
7.2.3 Measurement of the light intensity with a powermeter 57
7.3 Optical setups 57
7.3.1 Epifluorescence microscope 57
Description 57
Measurement of the light intensity at the focal plane with a powermeter 58
7.3.2 Fluorescence macroimager 59
7.3.3 Confocal microscopy 60
7.3.4 Fluorometer for acquisition of the fast OJIP transient fluorescence response to **PA** illumination . . 60
7.3.5 Fluorescence Correlation Spectroscopy 61
7.4 Intrinsically fluorescent actinometers 61
7.4.1 **Cin** 61
Preparation of the **Cin** solutions 61
Absorption spectrum of **Cin** and emission spectrum of its coumarin photoproduct 61
Determination of the quantum yield of **Cin** photoconversion in pH 7 Tris buffer at 405 nm 62
Dependence of the quantum yield of the **Cin** photoconversion on the excitation wavelength 63
Estimate of the achievable measurement uncertainty on light intensity I 63
7.4.2 **Dronpa-2** 64
Preparation of the **Dronpa-2** samples 65
Absorption and emission spectra of **Dronpa-2** 65
Determination of the cross sections of photoconversion of **Dronpa-2** in bacteria at 480 and 405 nm 65
Comparison of the cross sections of photoconversion of **Dronpa-2** at 480 nm in solution and in a polyacrylamide gel 66
Dependence of the cross sections of the **Dronpa-2** photoconversion on the excitation wavelength . 68
Estimate of the achievable measurement uncertainty on light intensity I 68
7.4.3 **DASA** 69
Preparation of the **DASA** solution 70
Absorption and emission spectra of **DASA** 70
Determination of the quantum yield of **DASA** photoconversion in acetonitrile at 632 nm 70
Dependence of the quantum yield of the **DASA** photoconversion on the excitation wavelength . . 71
Decay of the **DASA** absorbance under illumination 71
Decay of the **DASA** fluorescence under illumination 72

Estimate of the achievable measurement uncertainty on light intensity I 73
7.4.4 **Photosynthetic apparatus (PA)** 73
Conditioning of the **PA**-containing samples 74
Fluorescence excitation and emission spectra of **PA** 74
Determination of the cross section of **PA** photoconversion at 470 nm 75
Dependence of the cross section of the **PA** photoconversion on the excitation wavelength 76
Estimate of the achievable measurement uncertainty on light intensity I 77
7.5 Combination of a non-fluorescent actinometer with a fluorescent reporter 78
7.5.1 α-(4-Diethylamino)phenyl)-N-phenylnitrone (**Nit**) 78
Preparation of the **Nit** solutions 78
Absorption and emission spectra of **Nit** 78
Determination of the quantum yield of **Nit** photoconversion in ethanol at 405 nm 78
Dependence of the quantum yield of the **Nit** photoconversion on the excitation wavelength 79
Decay of the **Nit** absorbance under illumination 79
Rise of the **RhB** fluorescence under illumination 80
Estimate of the achievable measurement uncertainty on light intensity I 81
7.6 **DDAO** for transferring information on light intensity from a wavelength to another 82
7.6.1 Preparation of the **DDAO** solutions 82
7.6.2 Absorption and fluorescence emission spectra of **DDAO** 82
7.6.3 Validation of **DDAO** as light intensity-transferring fluorophore 82
Regime of low light intensity 83
Regime of high light intensity 84
Estimate of the achievable measurement uncertainty on light intensity I 85

8 Supplementary Note 8: Validation of the extraction of the light intensity in fluorescence imaging 86
8.1 Fluorescence macroimager 86
8.2 Confocal microscopes 88
8.2.1 Theory 88
Photoconversion along a line 89
Photoconversion of a square 90
8.2.2 Confocal microscopy equipped with a continuous laser 91
Measurement of the radial waist of the laser beam 91
Measurement of the light intensity with the fluorescent actinometer 92
Measurement of the light intensity with the powermeter 93

Conclusion . . . 94
8.2.3 Confocal microscopy equipped with a pulsed laser . . . 94
Measurement of the radial waist of the laser beam . . . 94
Measurement from Raster image correlation spectroscopy (RICS) . . . 95
Measurement from collecting the point spread function . . . 96
Measurement of the light intensity with the fluorescent actinometer . . . 96
Measurement of the light intensity with the powermeter . . . 96
Conclusion . . . 97

9 Supplementary Note 9: Measurement of light intensity from a LED array 97
9.1 Experimental Setup . . . 98
9.2 Simulation Setup and Protocol . . . 99
9.3 Results and Discussion . . . 99
9.3.1 Simulated 3D-distribution of absorbed light flux . . . 99
9.3.2 Simulated 2D-maps of the characteristic times of **Dronpa-2** photoconversion and light intensity . 99
Theoretical derivation . . . 99
Results and discussion . . . 101

1 Supplementary Note 1: Materials

The following subsections report on (i) the characterization of the commercially available fluorophores and (ii) the synthetic schemes associated with the production of the synthetic fluorescent actinometers, which have been used to implement and validate the protocols for measuring light reported in this manuscript.

1.1 Characterization of the commercially available fluorophores

Two commercially available fluorophores have been used in the following: Rhodamine B (**RhB**) and 7-hydroxy-9H-(1,3-dichloro-9,9-dimethylacridin-2-one) (**DDAO**). To enable the end-users to control the quality of their sample of those fluorophores, we provide below two series of analytical characterizations:

- Their absorption, fluorescence excitation, and fluorescence emission spectra (Figure S1a,b);

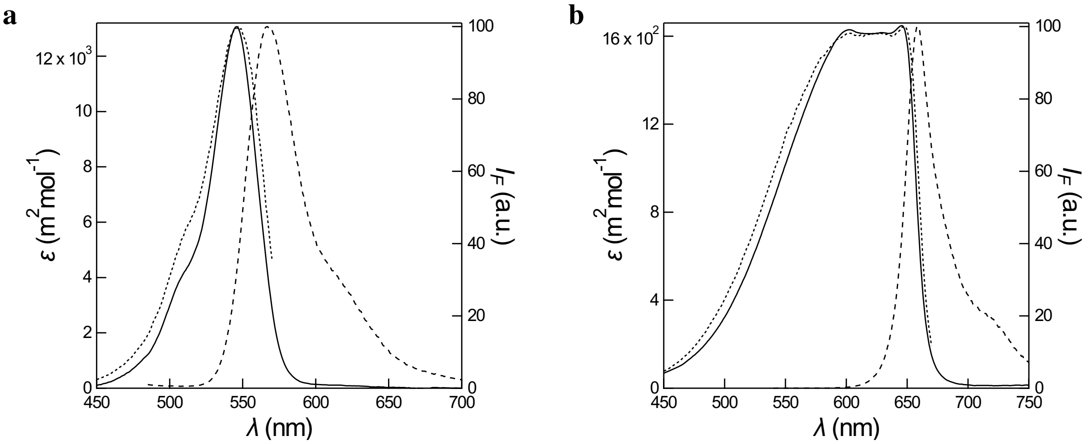

Figure S1: Absorption (solid line), fluorescence excitation (dotted line), and fluorescence emission (dashed line) spectra of 0.8 μM Rhodamine B (**a**; **RhB**; Solvent: Ethanol; $\lambda_{\mathrm{em}} = 574$ nm and $\lambda_{\mathrm{exc}} = 480$ nm for the excitation and emission spectra respectively) and 7.4 μM 7-hydroxy-9H-(1,3-dichloro-9,9-dimethylacridin-2-one) (**b**; **DDAO**; Solvent: pH = 7.9 HEPES buffer (100 mM NaCl, 5 mM NaOH, 10 mM HEPES); $\lambda_{\mathrm{em}} = 675$ nm and $\lambda_{\mathrm{exc}} = 520$ nm for the excitation and emission spectra respectively). $T = 298$ K.

- Their chromatography in HPLC. The experiments were carried out using 2.6 μM Rhodamine B (**RhB**) and 10 μM **DDAO** solutions in H_2O. They have been analyzed by high-pressure liquid chromatography using a C18 column (4.6×50 mm, particle size 2.7 μm; Poroshell 120) operating at 1.0 mL/min and thermostated at 25 °C. The detection of **RhB** and **DDAO** was performed at 565 nm and 450 nm respectively. 15 μL of **RhB** or **DDAO** solutions were injected in the chromatographic system. **RhB** was eluted with a mobile phase composed of two solvents A (methanol) and B (water) whereas **DDAO** was eluted with two solvents A (methanol) and C (0.1% formic acid in H_2O). A gradient was used to optimize the separation of the analytes:
  - For **RhB** (Figure S2a), the gradient used was as follow: Initially, the column was equilibrated with a mobile phase consisting of 50% A and 50% B. Six minutes after the injection, the proportion of A was linearly

increased to 80% and continued at 80% A for another 2 minutes. After this step, the composition of the mobile phase was set to initial condition within 1 min and the column was equilibrated for 6 min prior to next injection. The retention time for **RhB** was $t_R = 6.1$ min in these experimental conditions.

- For **DDAO** (Figure S2b), the gradient used was as follow: Initially, the column was equilibrated with a mobile phase consisting of 50% A and 50% C. Seven minutes after the injection, the proportion of A was linearly increased to 90% and continued at 90% A for another 1 min. After this step, composition of the mobile phase was set to initial condition within 1 min and the column was equilibrated for 6 min prior to next injection. The retention time for **DDAO** was $t_R = 5.3$ min in these experimental conditions.

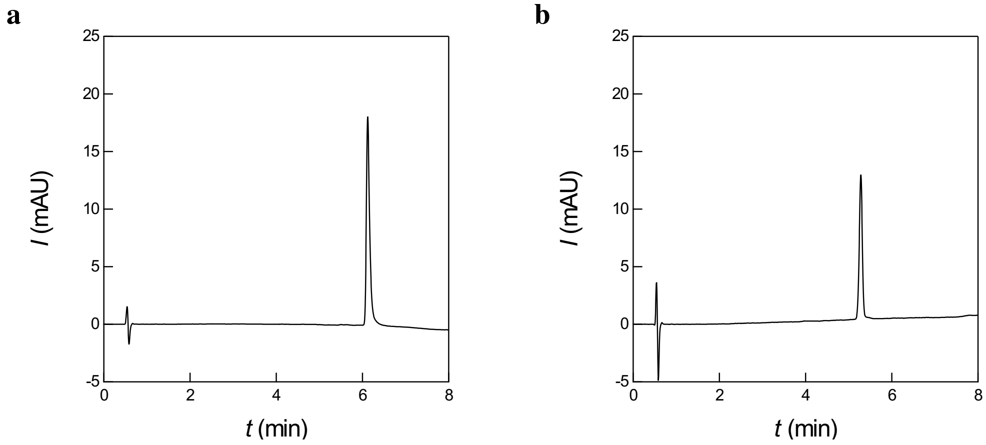

Figure S2: HPLC Chromatography of 2.6 μM Rhodamine B (**a**; **RhB**) and 10 μM 7-hydroxy-9H-(1,3-dichloro-9,9-dimethylacridin-2-one) (**b**; **DDAO**). See Text. $T = 298$ K.

1.2 Syntheses

1.2.1 *(E)*-3-(3,5-Dibromo-2,4-dihydroxyphenyl) acrylic acid ethyl ester (Cin)[3]

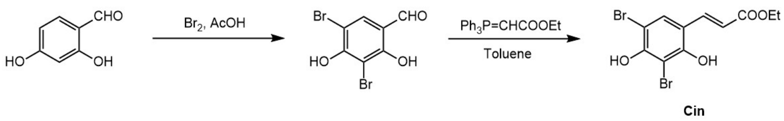

Figure S3: Synthetic pathway of **Cin**.

1.2.2 α-(4- Diethylamino)phenyl)-N-phenylnitrone (Nit)[4]

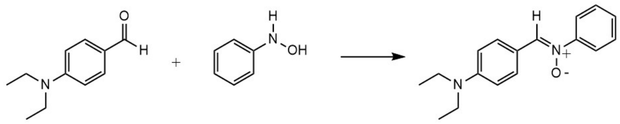

Figure S4: Synthetic pathway of **Nit**.

1.2.3 Sodium 4-(4-((2Z,4E)-2-hydroxy-5-(indolin-1-yl)penta-2,4-dien-1-ylidene)-3-methyl-5-oxo-4,5-dihydro-1H-pyra-zol-1-yl)benzenesulfonate (DASA)[5]

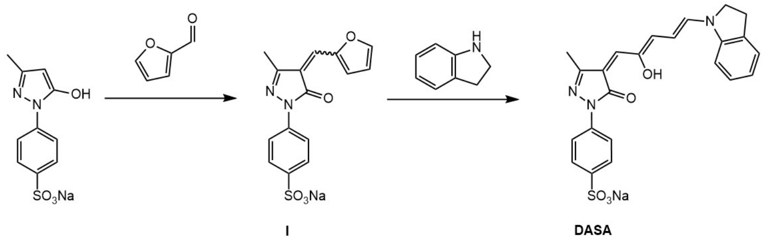

Figure S5: Synthetic pathway of **DASA**.

2 Supplementary Note 2: Protocols of implementation of the fluorescent systems for measuring light intensity

2.1 Measurements of light intensity with the fluorescent actinometers

2.1.1 Reagents

- Fluorescent actinometer among **Cin**, **Nit** and Rhodamine B **RhB**, **Dronpa-2**, **DASA**, or **PA**
- Solvent to produce the solution of the fluorescent actinometer:
  - 20 mM Tris pH 7 100 mM NaCl buffer for **Cin**
  - Spectrograde ethanol for **Nit**
  - DPBS pH 7.4 buffer (2.7 mM KCl, 138 mM NaCl, 1.5 mM KH_2PO_4, 8.1 mM Na_2HPO_4) or Tris buffer pH 7.4 (50 mM Tris, 150 mM NaCl) for **Dronpa-2**
  - Spectrograde DMSO and spectrograde acetonitrile for **DASA**
  - Heterotrophic media TAP [1] for **PA**

2.1.2 Equipments

- Balance to prepare the solutions
- Brown glassware or Aluminium foil to keep the solutions in the darkness
- Spectral data available online $\mathrm{https://chart-studio.plotly.com/\sim Alienor134/\#/}$
- Fluorimeter or any optical instrument, which can measure and record the time evolution of the fluorescence signal from the fluorescent actinometer
- Quartz cuvette or glass microscope slides with a 100 μm spacer to build a chamber

[1] https://www.chlamycollection.org/methods/media-recipes/tap-and-tris-minimal/

- Software for fitting the time evolution of the fluorescence response to illumination

2.1.3 Procedure

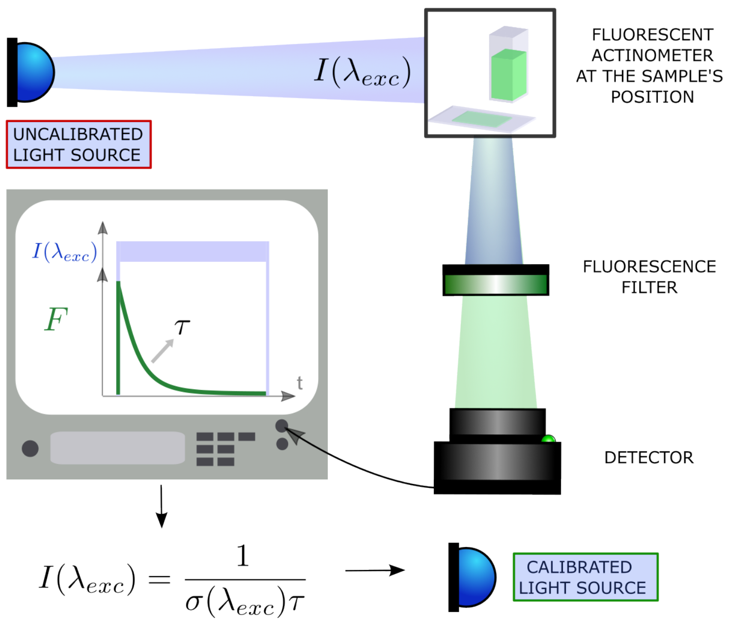

Figure S6: *Fluorescence reporting for retrieving light intensity with a fluorescent actinometer*. A jump of constant light I is applied onto the actinometer. The time evolution of its fluorescence signal F is recorded and fit with a mono-exponential curve to extract its characteristic time τ. I is retrieved from τ by using the photoconversion cross section σ of the actinometer.

Generic protocol (Figure S6)

1. Have a solution of the fluorescent actinometer (liquid or solid), which exhibits an absorbance lower than 0.15 along the optical path;

2. Suddenly expose the solution of the fluorescent actinometer to illumination, set at the level of light intensity I to be measured;

3. Collect the time evolution of the fluorescence emission signal;

4. Except for the **PA** actinometer (see below), process the time evolution of the fluorescence emission signal $F\left(\lambda_{\text{exc}}, \lambda_{\text{em}}, t\right)$ (or $F\left(\lambda_{\text{exc}}, \lambda_{\text{em}}, \vec{r}, t\right)$ in fluorescence imaging) by the fitting of a mono-exponential curve given in Eq.(S1), to enable the retrieval of the associated characteristic time τ (or $\tau\left(\vec{r}\right)$ in fluorescence imaging).

$$F\left(\lambda_{\text{exc}}, \lambda_{\text{em}}, t\right) = F\left(\lambda_{\text{exc}}, \lambda_{\text{em}}, \infty\right) + \left[F\left(\lambda_{\text{exc}}, \lambda_{\text{em}}, 0\right) - F\left(\lambda_{\text{exc}}, \lambda_{\text{em}}, \infty\right)\right] \exp\left(-\frac{t}{\tau}\right) \quad \text{(S1)}$$

   First apply the unsupervised monoexponential fitting function given in Eq.(S1) over the whole acquisition window to extract a first guess of the characteristic time τ_1. Then restrict the time window to $[0, 5\tau_1]$ and extract a second value τ_2 using the same monoexponential fitting function. This last τ value is used for the next step.

5. Compute the level of light intensity I as $I = \frac{1}{\sigma\tau}$ where σ is the photoconversion cross section tabulated below.

6. Check that the computed level of light intensity I is within the appropriate range of light intensity I tabulated below.

7. The preceding computation yields the level of light intensity I in $\mathrm{E.m^{-2}.s^{-1}}$. Denoting its values in $\mathrm{E.m^{-2}.s^{-1}}$ and $\mathrm{W.m^{-2}}$ as $I(\lambda_{exc}, \mathrm{E.m^{-2}.s^{-1}})$ and $I(\lambda_{exc}, \mathrm{W.m^{-2}})$ respectively, the relation between $I(\lambda_{exc}, \mathrm{E.m^{-2}.s^{-1}})$ and $I(\lambda_{exc}, \mathrm{W.m^{-2}})$ is given in Eq.(S12)

$$I(\lambda_{exc}, \mathrm{W.m^{-2}}) = \frac{hcN_A}{\lambda_{exc}} \times I(\lambda_{exc}, \mathrm{E.m^{-2}.s^{-1}}) \approx 0.12 \times \frac{\mathrm{I}(\lambda_{exc}, \mathrm{E.m^{-2}.s^{-1}})}{\lambda_{exc}\,(\mathrm{m})} \quad \text{(S2)}$$

with the Planck constant $h = 6.63\ 10^{-34}\ \mathrm{m^2.kg.s^{-1}}$, speed of light in a vacuum $c = 3.00\ 10^8\ \mathrm{m.s^{-1}}$, the Avogadro number $N_A = 6.02\ 10^{23}\ \mathrm{mol^{-1}}$, and where λ_{exc} is in m.

Description of five fluorescent actinometers covering the entire UV-Vis wavelength range The following specific protocols exploit five fluorescent actinometers covering the entire UV-Vis wavelength range for measurement of light intensity (Figure S7):

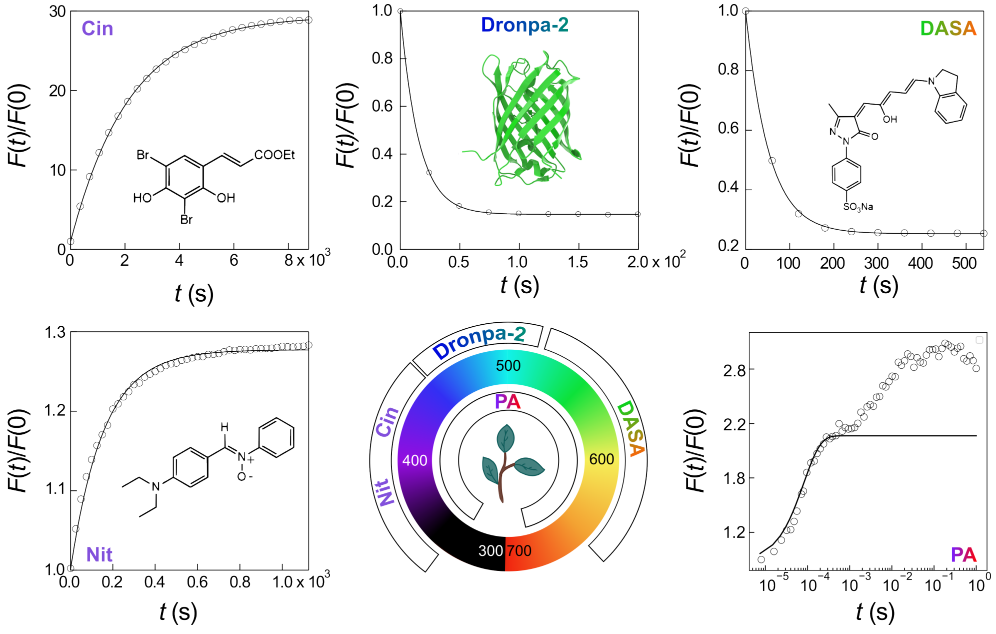

Figure S7: *Five fluorescent actinometers covering the UV-Vis range in action*. Monoexponential fit of the time evolution of the normalized fluorescence signal $F(t)/F(0)$ provides τ.

- Two actinometers for the UV A wavelength range (relevant for decontamination of materials, evaluation of environmental radiation, photoactivation of many caged molecules in optogenetics, photocatalysis with metal complexes, etc): (i) The dark (E)-3-(3,5-dibromo-2,4-dihydroxyphenyl) acrylic acid ethyl ester (**Cin**), which irreversibly converts under illumination between 350 and 420 nm into the bright 6,8-dibromo-7-hydroxycoumarin

fluorescing between 400 and 550 nm in Tris pH 7 buffer (Figure S8a); (ii) The dark α-(4-diethylamino)phenyl)-N-phenylnitrone (**Nit**), which irreversibly converts under illumination between 320 and 430 nm into the dark N-(p-dimethylaminophenyl)formanilide in ethanol (Figure S8b). The photochemically inert rhodamine B (**RhB**) emitting fluorescence between 550 and 650 nm is used here for reporting on **Nit** photoconversion with fluorescence;

- One actinometer for the blue wavelength range (important in optogenetics for photoactivating opsins, FAD CRY, FAD BLUF, and FMN LOV systems, or driving photosynthesis): A bright reversibly photoswitchable fluorescent protein **Dronpa-2** (or M159T), contained within Escherichia coli or eucaryotic cells, or in buffered solution or polyacrylamide gel, emitting fluorescence between 500 and 600 nm, which reversibly converts into a dark photoisomerized state under illumination between 400 and 550 nm (Figure S8c);

- One actinometer for the green to red wavelength range (important for photoactivating opsins or bilin PHY3 in optogenetics, or driving photosynthesis): In acetonitrile, the donor-acceptor Stenhouse dye **DASA** emitting fluorescence extending up to 675 nm reversibly converts into a dark state under illumination between 530 and 670 nm (Figure S8d);

- Since the width of the absorption band of the preceding fluorescent actinometers is limited which necessitates to have several of them to cover the whole range of wavelengths, we eventually report on the last fluorescent actinometer, the photosynthetic apparatus of algae (denoted **PA**), which can provide an estimate of light intensity for the entire visible range of wavelengths. In oxygenic photosynthetic organisms, a few percent of collected sunlight energy is released as fluorescence in the 650-800 nm range. When exposed to constant light at sun-like light intensity, the fluorescence of dark-adapted photosynthetic organisms rises in less than 1 s from a minimum to a maximum via intermediate steps (Figure S8e). The rate constant of the fastest step linearly depends on the light intensity. Usefully, its value does not significantly depend on the type of photosynthetic organism.

These fluorescent actinometers are easily accessible to different communities of end users either via syntheses (for **Cin**, **Nit**, **DASA**) or as protein (**Dronpa-2**) and microorganisms (**Dronpa-2**, **PA**) for end users with access to biological techniques.

Specific protocols

Cin to measure light intensity in the 350–420 nm range

1. Prepare a 10 mM stock solution of **Cin** (MW: 365.9 g.mol^{-1}) by dissolving 3.8 mg of **Cin** powder in 1 mL of spectrograde DMSO.

2. Dilute the 10 mM stock solution in 20 mM Tris pH 7 100 mM NaCl buffer to yield a final solution with absorbance equal to 0.15 along the optical path ℓ of the investigated sample. For instance, it yields [**Cin**] $\simeq$ 6 μM for the $\ell = 1$

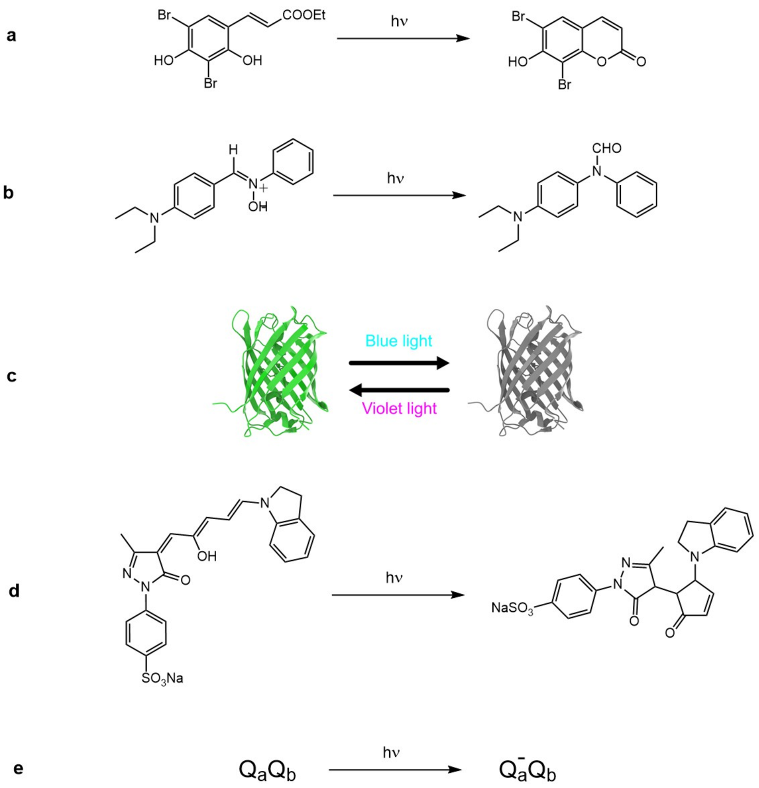

Figure S8: *Photoconversion of the fluorescent actinometers:* **Cin** (**a**), **Nit** (**b**), **Dronpa-2** (**c**), **DASA** (**d**), **PA** (**e**).

cm representatives containers used in photocatalysis or [**Cin**] = 0.6 mM for a solution sandwiched between two glass slides with a 100 μm-thick spacer for measuring light intensity in microscopy applications of optogenetics. This solution can be kept at 4 °C for a week under the protection from ambient light (e.g. by using a brown glassware enveloped in an Aluminium foil). The reference absorption and fluorescence emission spectra of **Cin**, as well as the list of its molar absorption coefficient at various wavelengths is provided online (see also Figure S26 and Table S4).

3. Fill the container with the final solution.

4. Expose the container to time constant monochromatic light at wavelength $\lambda_{\rm exc}$ in the [350 nm;420 nm] wavelength range.

5. Record the fluorescence intensity at 456 nm,[2] $F(\lambda_{\rm exc}$,456 nm,$t)$, as a function of time (Figure S9). If illumination of interest and fluorescence measurements are performed on a same instrument, $F(\lambda_{\rm exc}$,456 nm,$t)$ can be recorded continuously upon illumination at the targeted excitation wavelength. Otherwise, the excitation can be performed in one instrument, and the fluorescence read in another. In this case the excitation has to be performed stepwise by recording each exposure duration to account for the time scale. The fluorescence should be measured between

[2]This wavelength corresponds to the maximum of fluorescence emission and it is given here as an example. Any wavelength between 400 and 550 nm can be used with fluorescence reporting, albeit with a lower fluorescence signal.

each step, and will be optimal when exciting at 368 nm, which is the maximum of absorption wavelength of the photogenerated 6,8-dibromo-7-hydroxycoumarin.

6. Process the time evolution of the gathered fluorescence signal with the monoexponential fitting equation given in Eq.(S1) to retrieve the relaxation time τ (Figure S9).

7. Extract the light intensity I as $I = \frac{1}{\sigma\tau}$ by using the relevant value of the cross section of **Cin** photoconversion $\sigma(\lambda_{\mathrm{exc}})$ given in Table S4.

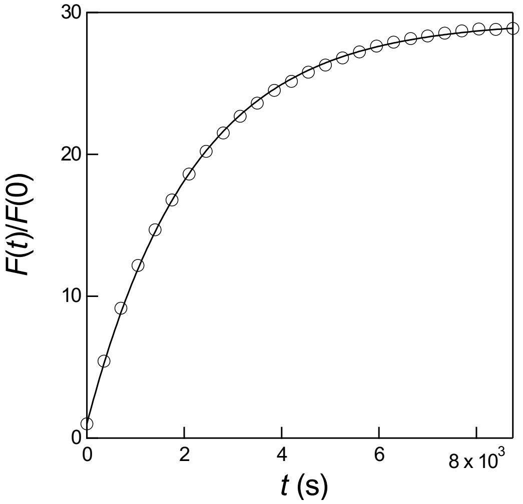

Figure S9: *Representative time evolution of the fluorescence signal with the **Cin** actinometer.* Time dependence of the normalized fluorescence emission at 456 nm of a 5.6 μM **Cin** solution in 20 mM Tris pH 7 100 mM NaCl buffer contained in a 1×1 cm^2 quartz cuvette upon illumination at constant light intensity at $\lambda_{\mathrm{exc}} = 405$ nm. Markers: Experimental data; solid lines: Monoexponential fit with Eq.(S1). Using data provided in Table S4, the characteristic time $\tau = 2160$ s retrieved from the fit yields $I(\lambda_{\mathrm{exc}}) = 2.5$ μE.m^{-2}.s^{-1} (0.7 W.m^{-2}). $T = 293$ K.

Nit to measure light intensity in the 320–430 nm range

1. Prepare stock solutions of **Nit** (MW: 240 g.mol^{-1}; 3.0 mg/ml; 12.5 mM) and **RhB** (MW: 479 g.mol^{-1}; 2.9 mg/mL; 6 mM) in spectrograde ethanol.

2. Dilute the **Nit** stock solution in spectrograde ethanol to produce the final solutions upon taking care to exhibit an absorbance lower or equal to 0.15 at the absorption maximum along the optical path ℓ of the investigated sample. For instance, it yields [**Nit**] = 5.8 μM for the $\ell = 1$ cm representatives containers used in photocatalysis or [**Nit**] = 0.58 mM for a solution sandwiched between two glass slides with a 100 μm-thick spacer for measuring light intensity in microscopy applications of optogenetics.

3. Dilute the **RhB** stock solution in the preceding **Nit** solution in ethanol to produce a solution further containing Rhodamine B (**RhB**) at [**RhB**] = 1 μM. This solution can be kept at 4 °C for a week under the protection from

ambient light (e.g. by using a brown glassware enveloped in an Aluminium foil). The reference absorption and fluorescence emission spectra of **Nit**, as well as the list of its molar absorption coefficient at various wavelengths is provided online (see also Figure S40 and Table S8).

4. Fill the container with the final solution.

5. Expose the container to time constant monochromatic light at wavelength $\lambda_{\rm exc}$ in the [320 nm;430 nm] wavelength range.

6. Record the fluorescence intensity at 574 nm,[3] $F(\lambda_{\rm exc}$,574 nm,$t)$, as a function of time (Figure S10). If illumination of interest and fluorescence measurements are performed on a same instrument, $F(\lambda_{\rm exc}$,574 nm,$t)$ can be recorded continuously upon illumination at the targeted excitation wavelength. Otherwise, the excitation can be performed in one instrument, and the fluorescence read in another. In this case the excitation has to be performed stepwise by recording each exposure duration to account for the time scale. The fluorescence should be measured between each step, and will be optimal when exciting at 555 nm, which is the maximum of absorption wavelength of **RhB**.

7. Process the time evolution of the gathered fluorescence signal with the monoexponential fitting equation given in Eq.(S1) to retrieve the relaxation time τ (Figure S10).

8. Extract the light intensity I as $I = \frac{1}{\sigma\tau}$ by using the relevant value of the cross section of **Nit** photoconversion $\sigma(\lambda_{\rm exc})$ given in Table S8.

Dronpa-2 to measure light intensity in the 440–500 nm range

1. Dilute the **Dronpa-2**-labeled *Escherichia coli* cells and **Dronpa-2** solution in DPBS pH 7.4 buffer (2.7 mM KCl, 138 mM NaCl, 1.5 mM KH_2PO_4, 8.1 mM Na_2HPO_4, Thermofischer) or in Tris buffer pH 7.4 (50 mM Tris, 150 mM NaCl) to get final solutions, which exhibit an absorbance lower than 0.15 at 488 nm along the optical path ℓ. These solutions can be kept at 4 ^{o}C for weeks under the protection from ambient light (e.g. by using a brown glassware enveloped in an Aluminium foil). The reference absorption and fluorescence emission spectra of **Dronpa-2**, as well as the list of its molar absorption coefficient at various wavelengths is provided online (see also Figure S29 and Table S5). The **Dronpa-2**-labeled eucaryotic cells are directly used after fixation (see Methods in the Main Text). They can be kept for at least a week at 4 ^{o}C in the dark.

2. Fill the container with the final **Dronpa-2**-labeled *Escherichia coli* cells and **Dronpa-2** solution.

3. Expose the container (in the case of the **Dronpa-2**-labeled *Escherichia coli* cells and **Dronpa-2** solution) or the **Dronpa-2**-labeled eucaryotic cells to time constant monochromatic light at wavelength $\lambda_{\rm exc}$ in the [445 nm;500 nm] wavelength range.

[3] This wavelength corresponds to the maximum of fluorescence emission of **RhB** and it is given here as an example. Any wavelength between 550 and 650 nm can be used with fluorescence reporting, albeit with a lower fluorescence signal.

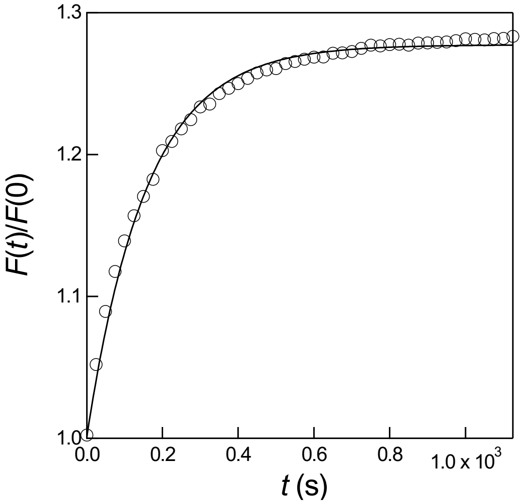

Figure S10: *Representative time evolution of the fluorescence signal with the **Nit** actinometer.* Time dependence of the normalized fluorescence emission at 574 nm of a 5.8 μM **Nit** and 1μM **RhB** solution in spectroscopy grade ethanol contained in a 1×1 cm^2 quartz cuvette upon illumination at constant light intensity at λ_{exc} = 380 nm. Markers: Experimental data; solid lines: Monoexponential fit with Eq.(S1). Using data provided in Table S8, the characteristic time τ = 157 s retrieved from the fit yields $I(\lambda_{\text{exc}}) = 5.3$ μE.m^{-2}.s^{-1} (1.7 W.m^{-2}). T = 293 K.

4. Record the fluorescence intensity at 515 nm,[4] $F(\lambda_{\text{exc}}$,515 nm,t), as a function of time (Figure S11). If illumination of interest and fluorescence measurements are performed on a same instrument, $F(\lambda_{\text{exc}}$,515 nm,t) can be recorded continuously upon illumination at the targeted excitation wavelength. Otherwise, the excitation can be performed in one instrument, and the fluorescence read in another. In this case the excitation has to be performed stepwise by recording each exposure duration to account for the time scale. The fluorescence should be measured between each step, and will be optimal when exciting at 488 nm, which is the maximum of absorption wavelength of **Dronpa-2**.

5. Process the time evolution of the gathered fluorescence signal with the monoexponential fitting equation given in Eq.(S1) to retrieve the relaxation time τ (Figure S11).[5]

6. Extract the light intensity I as $I = \frac{1}{\sigma\tau}$ by using the relevant value of the cross section of **Dronpa-2** photoconversion $\sigma(\lambda_{\text{exc}})$ given in Table S5.

DASA to measure light intensity in the 530–670 nm range

1. Prepare a stock solution of **DASA** (MW: 450 g.mol^{-1}) in spectrograde DMSO in order to yield a stock solution at about 1 mg.mL^{-1}.

2. Dilute the **DASA** stock solution in spectrograde acetonitrile to generate a final solution with absorbance equal to 0.15 along the optical path ℓ of the investigated sample. As a representative example for applications in photochem-

[4]This wavelength corresponds to the maximum of fluorescence emission of **Dronpa-2** and it is given here as an example. Any wavelength between 500 and 600 nm can be used with fluorescence reporting, albeit with a lower fluorescence signal.

[5]A Jupyter notebook Notebooks/Dronpa2_video.ipynb is available to perform the corresponding computations online with Binder.

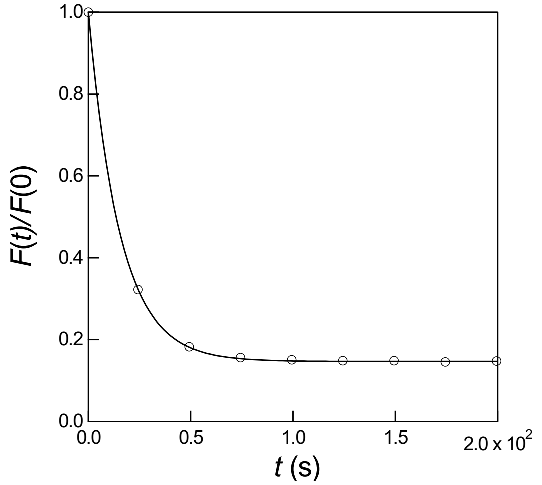

Figure S11: *Representative time evolution of the fluorescence signal with the* ***Dronpa-2*** *actinometer.* Time dependence of the normalized fluorescence emission at 515 nm from **Dronpa-2**-labeled bacteria in DPBS $1\times$ pH 7.4 buffer in 3 mm optical path cuvette upon illumination at constant light intensity at $\lambda_{\mathrm{exc}} = 480$ nm. Markers: Experimental data; solid lines: Monoexponential fit with Eq.(S1). Using data provided in Table S5, the characteristic time $\tau = 15$ s retrieved from the fit yields $I(\lambda_{\mathrm{exc}}) = 270$ μE.m^{-2}.s^{-1} (69 W.m^{-2}). $T = 293$ K.

istry, we adopt $\ell = 0.3$ cm and [**DASA**] = 40 μM. As a representative example for applications in optogenetics and fluorescence microscopy, [**DASA**] = 100 μM for a **DASA** solution sandwiched between to glass slides with a $\ell = 200$ μm-thick spacer. The **DASA** solutions have to be equilibrated at room temperature under the protection from ambient light (e.g. by using a brown glassware enveloped in an Aluminium foil) for 2 h after preparation, and used no later than a day after their production. The reference absorption and fluorescence emission spectra of **DASA**, as well as the list of its molar absorption coefficient at various wavelengths is provided online (see also Figure S33 and Table S6).

3. Fill the container with the final solution.

4. Expose the container to time constant monochromatic light at wavelength λ_{exc} in the [530 nm; 670 nm] wavelength range.

5. Record the fluorescence intensity in the [650 nm; 675 nm] wavelength range $F(\lambda_{\mathrm{exc}},\lambda_{\mathrm{em}},t)$, as a function of time (Figure S12). If illumination of interest and fluorescence measurements are performed on a same instrument, $F(\lambda_{\mathrm{exc}},\lambda_{\mathrm{em}},t)$ can be recorded continuously upon illumination at the targeted excitation wavelength. Otherwise, the excitation can be performed in one instrument, and the fluorescence read in another. In this case the excitation has to be performed stepwise by recording each exposure duration to account for the time scale and the fluorescence should be measured between each step.

6. Process the time evolution of the gathered fluorescence signal with the monoexponential fitting equation given in Eq.(S1) to retrieve the relaxation time τ (Figure S12).

7. Extract the light intensity I as $I = \frac{1}{\sigma\tau}$ by using the relevant value of the cross section of **DASA** photoconversion $\sigma(\lambda_{\mathrm{exc}})$ given in Table S6).

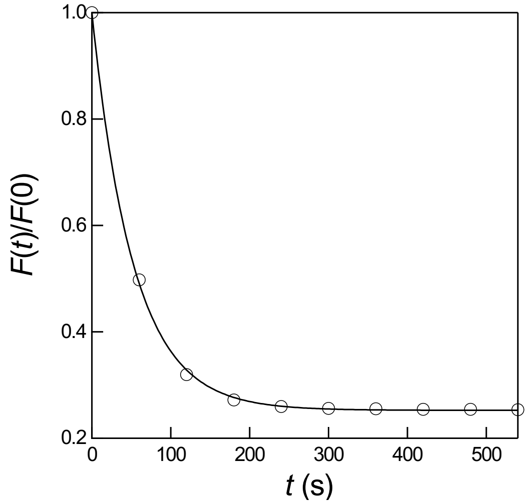

Figure S12: *Representative time evolution of the fluorescence signal with the **DASA** actinometer.* Time dependence of the normalized fluorescence emission of a 20 μM **DASA** solution in a 1:100 (v/v) mixture of spectroscopy grade DMSO and acetonitrile in a 3 mm light path quartz cuvette upon illumination at constant light intensity at $\lambda_{\mathrm{exc}} = 632$ nm. Markers: Experimental data; solid lines: Monoexponential fit with Eq.(S1). Using data provided in Table S6), the characteristic time $\tau = 52$ s retrieved from the fit yields $I(\lambda_{\mathrm{exc}}) = 15$ μE.m^{-2}.s^{-1} (2.9 W.m^{-2}). $T = 293$ K.

PA to measure light intensity in the 400–650 nm range

1. Prepare the microalgae solution according to the Methods in the Main Text.

2. Dilute the microalgae solution to generate a final solution with absorbance equal to 0.15 along the optical path ℓ of the investigated sample.

3. Fill the container with the final solution.

4. Expose the container to time constant monochromatic light at wavelength λ_{exc} in the [400 nm; 650 nm] wavelength range.

5. Record the time evolution of the fluorescence intensity at 690 nm,[6] $F(t)$, over 1 s at 3 MHz sampling frequency by using a fast photodetector (e.g. photodiode, MPPC, oscilloscope; see subsection 7.4.4) (Figure S13).

6. The time evolution of the fluorescence signal retrieved from applying constant illumination on the **PA** actinometer is more complex than the one of the other fluorescent actinometers[7] and it deserves a specific fitting protocol. Process

[6]This wavelength corresponds to the maximum of fluorescence emission of **PA** and it is given here as an example. Any wavelength between 650 and 750 nm can be used with fluorescence reporting, albeit with a lower fluorescence signal.

[7]For more details on the origin of the light-limited step of **PA**, refer to section 7.4.4.

the time evolution of the fluorescence signal with the following iterative fitting method to retrieve the relaxation time τ:[8]

- In a first step, apply an unsupervised fit with Eq.(S3)

$$F(t) = F(0) + A_{OJ}\left(1 - e^{-t/\tau_{OJ}}\right)^{s_{OJ}} + A_{JI}\left(1 - e^{-t/\tau_{JI}}\right)^{s_{JI}} + A_{IP}\left(1 - e^{-t/\tau_{IP}}\right)^{s_{IP}} \quad \text{(S3)}$$

  in order to retrieve a first estimate of the value of the characteristic time τ_{OJ} associated to the of the initial step of **PA** fluorescence rise ;

- In a second step, restrict the time window to [0;3τ_{OJ}] and apply the fit given in Eq.(S4) to the time evolution of the fluorescence emission

$$F(t) = F(0) + A\left(1 - e^{-t/\tau}\right)^{s} \quad \text{(S4)}$$

  upon fixing $s = 1.24$[6] in order to retrieve a second estimate of the value of the characteristic time τ associated to the initial step of **PA** fluorescence rise ;

- In the last step, restrict the time window to [0;5τ], apply the fit given in Eq.(S4) to the time evolution of the fluorescence emission upon adopting the values of parameters extracted during the second step as starting values, and retrieve the final value of the characteristic time τ associated to the initial step of **PA** fluorescence rise .

7. Extract the light intensity I as $I = \frac{1}{\sigma\tau}$ by using the relevant value of the cross section of **PA** photoconversion $\sigma(\lambda_{\text{exc}})$ given in Table S7.

2.1.4 Troubleshooting

General

- No time evolution of the fluorescence signal from the final solution of fluorescent actinometer under constant illumination: Check that the final solution has been kept at 4 °C under the protection from ambient light.

- If the cross section σ at an excitation wavelength belonging to the wavelength range of relevance for a given fluorescent actinometer is not tabulated in the reported Table, a good estimate can be retrieved from linear interpolation by exploiting the cross sections at the two closest tabulated values.

- With **Dronpa-2** and **DASA** which are reversibly photoswitchable fluorescent actinometers, the light intensity I obeys Eq.(S8)

$$I\left(\lambda_{\text{exc}}\right) = \frac{1 - k^{\Delta}\tau}{\sigma\tau} \quad \text{(S5)}$$

[8] A Jupyter notebook Notebooks/PA_OJIP_rise_fit.ipynb is available to perform the corresponding computations online with Binder.

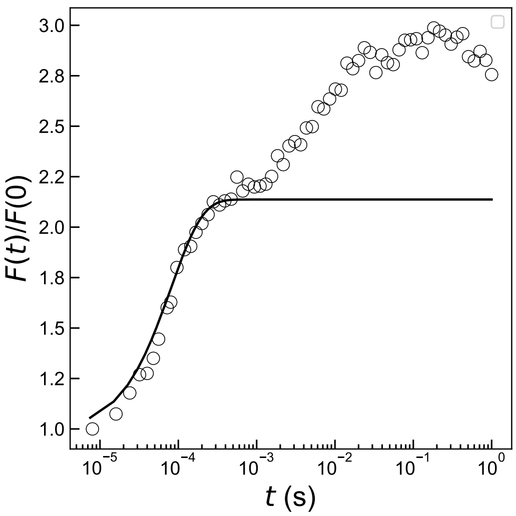

Figure S13: *Representative time evolution of the fluorescence signal with the* ***PA*** *actinometer.* Time dependence of the normalized fluorescence emission of *Chlamydomonas reinhardtii* (CC 124) in exponential growth phase upon illumination at constant light intensity at $\lambda_{\text{exc}} = 470$ nm. Markers: Experimental data; solid lines: Monoexponential fit with Eq.(S1). Using data provided in Table S7, the characteristic time $\tau = 71\ \mu$s retrieved from the fit yields $I(\lambda_{\text{exc}}) = 7.8\ m$E.m^{-2}.s^{-1} (2.0 kW.m^{-2}). $T = 293$ K.

where k^{Δ} is the rate constant associated to thermal relaxation after photoconversion (expressed in s^{-1}). Hence, using Eq.(S6)

$$I = \frac{1}{\sigma\tau} \tag{S6}$$

for retrieving I implies that $k^{\Delta}\tau \ll 1$, which has to be checked. k^{Δ} is respectively equal to 0.02 and 0.002 s^{-1} for **Dronpa-2**[7] and **DASA**[5] around room temperature.

- For calibration of light intensity in fluorescence imaging, Eq.(S1) becomes Eq.(S7)

$$F\left(\lambda_{\text{exc}}, \lambda_{\text{em}}, \overrightarrow{r}, t\right) = F\left(\lambda_{\text{exc}}, \lambda_{\text{em}}, \overrightarrow{r}, \infty\right) + \left[F\left(\lambda_{\text{exc}}, \lambda_{\text{em}}, \overrightarrow{r}, 0\right) - F\left(\lambda_{\text{exc}}, \lambda_{\text{em}}, \overrightarrow{r}, \infty\right)\right] \exp\left(-\frac{t}{\tau\left(\overrightarrow{r}\right)}\right) \tag{S7}$$

which yields the map $I\left(\overrightarrow{r}\right)$ sought for from Eq.(S8)

$$I\left(\overrightarrow{r}\right) = \frac{1}{\sigma\tau\left(\overrightarrow{r}\right)} \tag{S8}$$

Eqs.(S7,S8) are strictly valid when the actinometer molecules do not move. However, when they can diffuse (diffusion coefficient D), Eqs.(S1,S8) can still be used but the spatial resolution of the $I\left(\overrightarrow{r}\right)$ is then limited to $\sqrt{D\tau\left(\overrightarrow{r}\right)}$:[7] The faster the photoconversion, the higher the spatial resolution. At the limit of homogenization (e.g. upon stirring), the spatial information of the illumination profile is lost and one can retrieve the overall amount of light intensity I received by the actinometer solution from the measured overall relaxation time τ with Eq.(S6).

Specific

DASA

- The time evolution of the fluorescence signal from the DASA fluorescent actinometer does not conform to the observation in Figure S12: Check that the **DASA** solutions have been equilibrated at room temperature under the protection from ambient light for 2 h after preparation, and used no later than a day after their production.

PA

- The fluorescence rise starts decaying after reaching the maximum. The tri-exponential fit must be stopped at the maximum for better accuracy.
- When calibrating a red light source, make sure to exclude the excitation light from the detected light by using a narrow fluorescence filter (for example, a 700 nm filter to calibrate a 650 nm LED).
- Make sure that the observed rise time of the **PA** fluorescence signal is not driven by the rise time of your light source (e.g. due to heating in LEDs; generally in the μs range[7]) or of your photodetector by preliminarily analyzing the rise time of the fluorescence signal from a photochemically inert fluorophore (**DDAO** for instance) upon turning on light.[7]

2.1.5 Time taken

The duration of the overall measurement is evaluated to 2 h:

1. Preparation of the final solution of a fluorescent actinometer: 75 min
2. Collection of the time evolution of the fluorescence emission under constant illumination: 30 min as an order of magnitude (depending on the actinometer and light intensity)
3. Data processing: 15 min

2.1.6 Anticipated results

A general estimate of the achievable measurement uncertainty on I is 20%. Where photoconversion occurs rapidly, on a timescale where molecular motion is minimal, it is possible to retrieve a map of the spatial distribution of light intensity. However, if the molecules can visit the whole irradiated area at the timescale of the actinometer photoconversion, only mean light intensity values can be obtained.

2.1.7 References

- About actinometry: H. J. Kuhn, S. E. Braslavsky, and R. Schmidt, Chemical Actinometry (IUPAC Technical Report), *Pure Appl. Chem.*, **2004**, *76*, 2105–2146.

- About fluorescence: B. Valeur, M.-N. Berberan-Santos, Molecular Fluorescence: Principles and Applications 2nd Edition, Wiley, **2012**.

- About **Cin**: **1**. N. Gagey, P. Neveu, and L. Jullien. Reporting two-photon uncaging with the efficient 3,5-dibromo-2,4-dihydroxycinnamic caging group, *Angew. Chem. Intl. Ed.*, **2007**, *46*, 2467–2469.; **2**. N. Gagey, P. Neveu, C. Benbrahim, B. Goetz, I. Aujard, J. - B. Baudin, L. Jullien, Two-photon uncaging with fluorescence reporting: Evaluation of the o-hydroxycinnamic platform, *J. Am. Chem. Soc.*, **2007**, *129*, 9986–9998.

- About **Nit**: **1**. P. F. Wang, L. Jullien, B. Valeur, J.-S. Filhol, J. Canceill, J.-M. Lehn, Multichromophoric Cyclodextrins. 5. Antenna-induced Unimolecular Photoreactions. Photoisomerization of a Nitrone, *New J. Chem.*, **1996**, *20*, 895–907; **2**. M. Emond, T. Le Saux, S. Maurin, J.-B. Baudin, R. Plasson, L. Jullien, 2-Hydroxy-Azobenzenes to Tailor pH Pulses and Oscillations with Light, *Chem. Eur. J.*, **2010**, *16*, 8822–8831.

- About **Dronpa-2**: **1**. R. Chouket, A. Pellissier-Tanon, A. Lahlou, R. Zhang, D. Kim, M.-A. Plamont, M. Zhang, X. Zhang, P. Xu, N. Desprat, D. Bourgeois, A. Espagne, A. Lemarchand, T. Le Saux, L. Jullien, Extra kinetic dimensions for label discrimination, *Nat. Commun.*, **2022**, *13*, 1482; **2**. J. Quérard, R. Zhang, Z. Kelemen, M.-A. Plamont, X. Xie, R. Chouket, I. Roemgens, Y. Korepina, S. Albright, E. Ipendey, M. Volovitch, H. L. Sladitschek, P. Neveu, L. Gissot, A. Gautier, J.-D. Faure, V. Croquette, T. Le Saux, L. Jullien, Resonant out-of-phase fluorescence microscopy and remote imaging overcome spectral limitations, *Nat. Commun.*, **2017**, *8*, 969.

- About **DASA**: Y Shpinov, A Schlichter, P Pelupessy, T Le Saux, L Jullien, B Adelizzi, Unexpected acid-triggered formation of reversibly photoswitchable Stenhouse salts from Donor-acceptor Stenhouse adducts, *Chem. Eur. J.*, **2022**, *28*, e202200497.

- About **PA**: **1**. Maxwell, K., and Johnson, G. N., Chlorophyll fluorescence – a practical guide, J. Exp. Bot, **2000**, *51*, 659–668; **2**. D. Lazar, The polyphasic chlorophyll a fluorescence rise measured under high intensity of exciting light, *Funct. Plant Biol.*, **2006**, *33*, 9 – 30.

- About data processing: **1**. Mechanistic reduction leading to monoexponential fit of the time evolution of the fluorescence signal: R. Chouket, A. Pellissier-Tanon, A. Lahlou, R. Zhang, D. Kim, M.-A. Plamont, M. Zhang, X. Zhang, P. Xu, N. Desprat, D. Bourgeois, A. Espagne, A. Lemarchand, T. Le Saux, L. Jullien, Extra kinetic dimensions for label discrimination, *Nat. Commun.*, **2022**, *13*, 1482; **2**. About **PA** data processing: D. Joly, R. Carpentier, Sigmoidal reduction kinetics of the photosystem II acceptor side in intact photosynthetic materials during fluorescence induction, *Photochem. Photobiol. Sci.*, **2009**, *8*, 167–173.

2.2 A reporting fluorophore to transfer information on light intensity from one wavelength to another

7-Hydroxy-9H-(1,3-dichloro-9,9-dimethylacridin-2-one) (**DDAO**) is a suitable photochemically inert light intensity-transferring fluorophore (Figure S15). It is commercially available. It absorbs light between 450 and 650 nm and emits fluores-

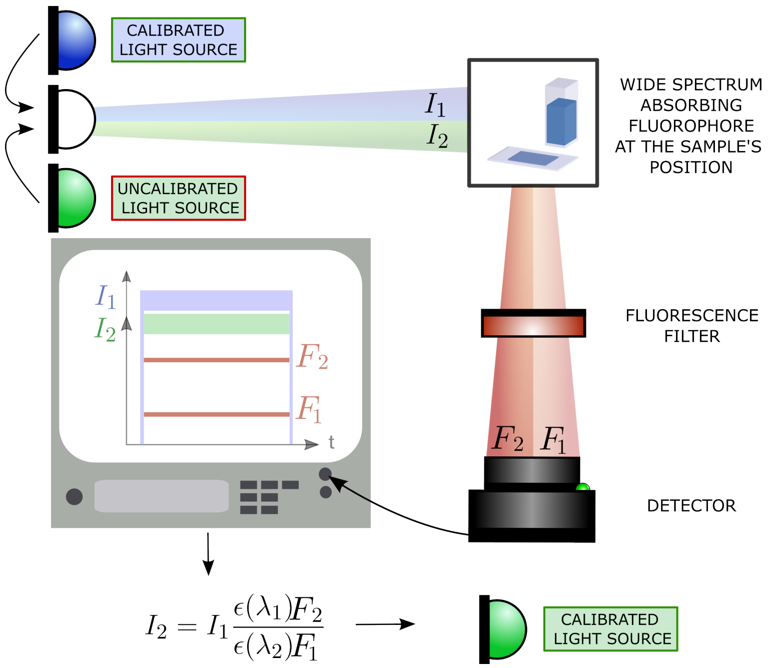

Figure S14: *Fluorescence reporting for retrieving light intensity by using a fluorophore to transfer information on light intensity from one wavelength to another.* Lights at wavelengths λ_1 (with intensity I_1 – known) and λ_2 (with intensity I_2 – to be measured) are successively applied onto the fluorophore and the associated fluorescence signals F_1 and F_2 are recorded at a same emission wavelength. I_2 is extracted from F_1 and F_2 by using I_1 and the tabulated fluorescence excitation spectrum $\epsilon(\lambda)$ of the fluorophore.

cence between 640 and 700 nm in neutral aqueous solutions, which is particularly attractive for light calibration in the orange and red wavelength range where actinometers are scarce and often exhibit a poor quantum yield of fluorescence. Eventually, its quantum yield of fluorescence does not depend on the excitation wavelength as evidenced by the similarity of its absorption and normalized fluorescence excitation spectrum.

2.2.1 Reagents

- 7-Hydroxy-9H-(1,3-dichloro-9,9-dimethylacridin-2-one) (**DDAO**)
- Solvent to produce the **DDAO** solution:
  - Spectrograde DMSO
  - Aqueous HEPES pH 7.9 buffer (100 mM NaCl, 5 mM NaOH, 10 mM HEPES)

2.2.2 Equipments

- Balance to prepare the **DDAO** solution
- Brown glassware or Aluminium foil to keep the solutions in the darkness
- Fluorimeter or any optical instrument, which can measure and record the fluorescence signal

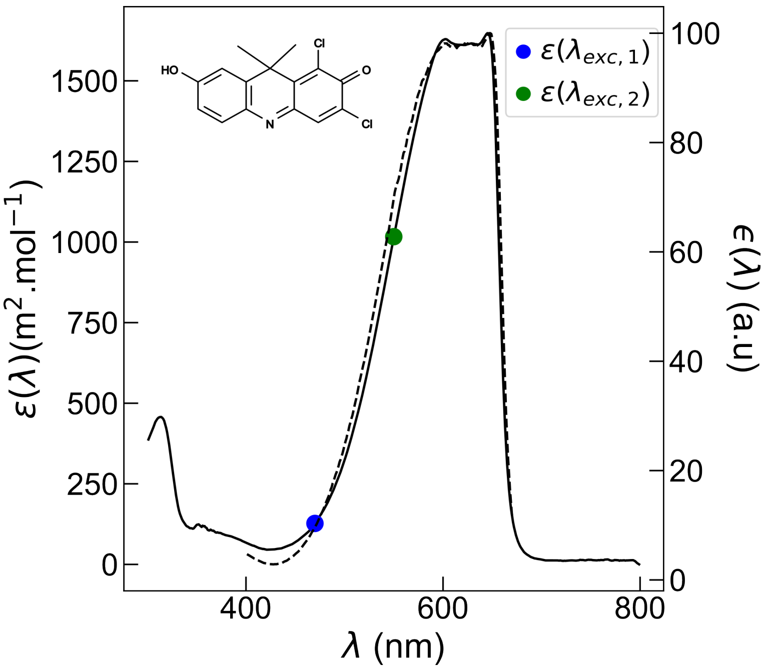

Figure S15: *Absorption ($\varepsilon(\lambda)$; dotted line) and normalized fluorescence excitation ($\epsilon(\lambda)$; solid line) spectra of DDAO.* $\epsilon(\lambda_1)$ and $\epsilon(\lambda_2)$ indicated by blue and green disks respectively are used to retrieve $I(\lambda_2)$ in Figure S14.

- Quartz cuvette or glass microscope slides with a 100 μm spacer to build a chamber

2.2.3 Procedure

1. Dissolve **DDAO** (MW: 308 g.mol^{-1}) in spectrograde DMSO in order to yield a stock solution at about 50 mM concentration.

2. Dilute the preceding stock solution in an aqueous HEPES pH 7.9 buffer (100 mM NaCl, 5 mM NaOH, 10 mM HEPES) to produce a final solution at **DDAO** concentration in the few μM range. The reference absorption and fluorescence emission spectra of **DDAO**, as well as the list of its molar absorption coefficient[9] at various wavelengths is provided online (see also Figure S44 and Table S9).

3. Submit the final solution of **DDAO** to illumination at two wavelengths $\lambda_{\text{exc},1}$ (with known intensity $I\left(\lambda_{\text{exc},1}\right)$ measured with one actinometer reported above) and $\lambda_{\text{exc},2}$ (with intensity $I(\lambda_{\text{exc},2})$ to be measured).

4. Collect the corresponding intensities of its fluorescence emission at a same emission wavelength between 640 and 700 nm.

5. Use Eq.(S9)

$$I(\lambda_{\text{exc},2}) = \frac{\varepsilon\left(\lambda_{\text{exc},1}\right) F\left(\lambda_{\text{exc},2}, \lambda_{\text{em}}\right)}{\varepsilon\left(\lambda_{\text{exc},2}\right) F\left(\lambda_{\text{exc},1}, \lambda_{\text{em}}\right)} I\left(\lambda_{\text{exc},1}\right). \tag{S9}$$

[9]In **DDAO**, the molar absorption coefficient $\varepsilon\left(\lambda_{\text{exc}}\right)$ and the scaled excitation coefficient $\epsilon\left(\lambda_{\text{exc}}\right)$ are identical.

to extract the light intensity sought for by using the list of its molar absorption coefficient at various wavelengths provided online and in Table S9.

2.2.4 Troubleshooting

- If the absorption coefficient ε at an excitation wavelength belonging to the wavelength range of light absorption by **DDAO** is not tabulated in Table S9, a good estimate can be retrieved from linear interpolation by exploiting the absorption coefficients at the two closest tabulated values.

2.2.5 Time taken

The duration of the overall measurement is evaluated to 2 h:

1. Preparation of the final solution of **DDAO**: 75 min
2. Collection of the **DDAO** fluorescence emission at two wavelengths: 30 min
3. Data processing with Eq.(S9): 15 min

2.2.6 Anticipated results

A general estimate of the achievable measurement uncertainty on I is 20%.

2.2.7 References

About **DDAO** as a dye:

- **1**. D. Warther, F. Bolze, J. Lonéard, S. Gug, A. Specht, D. Puliti, X.- H. Sun, P. Kessler, Y. Lutz, J.-L. Vonesch, B. Winsor, J.-F. Nicoud, M. Goeldner, Live-Cell One- and Two-Photon Uncaging of a Far-Red Emitting Acridinone Fluorophore, *J. Am. Chem. Soc.*, **2010**, *132*, 2585–2590; **2**. R. Labruère, A. Alouane, T. Le Saux, I. Aujard, P. Pelupessy, A. Gautier, S. Dubruille, F. Schmidt, L. Jullien, Self-immolation for uncaging with fluorescence reporting, *Angew. Chem. Int. Ed.*, **2012**, *51*, 9344–9347.

2.3 Measurement of the light intensity at 488 nm at the focal plane of fluorescence microscopes

2.3.1 Reagents

- Either fixed **Dronpa-2**-labeled *Escherichia coli* or eucaryotic cells at the nucleus, or 5–10 μM **Dronpa-2** solutions either in Tris buffer pH 7.4 (50 mM Tris, 150 mM NaCl) or in DPBS 1$\times$ pH 7.4 buffer (2.7 mM KCl, 138 mM NaCl, 1.5 mM KH_2PO_4, 8.1 mM Na_2HPO_4).

2.3.2 Equipments

- Fluorescence microscope
- Software for fitting the time evolution of the fluorescence response to illumination

2.3.3 Procedure

Epifluorescence microscope

1. Sandwich a **Dronpa-2** aqueous solution between two glass-slides with a 100 μm-thick spacer. Alternatively, you can use **Dronpa-2**-labeled *Escherichia coli* or eucaryotic cells.

2. Expose the sample to a pulse of constant 470 nm light. Alternatively, expose the sample to a train of four 30 s-long 470 nm light pulses separated by 2 min of darkness in order to benefit from additional data.[10]

3. Collect the fluorescence images at 550 nm.[11]

4. Apply monoexponential fits to the time evolution of the fluorescence signal at each pixel with Eq.(S1) to extract the map of characteristic time $\tau\,(\overrightarrow{r})$ (averaged over the successive light ON-light OFF cycle when using a train of light pulses).

5. Compute the light intensity at each pixel $I(470\ \text{nm},\vec{r})$ by using Eq.(S8) and the cross section σ at 488 nm provided in Table S5. A simplified version of the code is given in an online Jupyter notebook in Notebooks/Dronpa2_video.ipynb.

Confocal microscope equipped with a continuous 488 nm laser

1. Acquire 100 square images of the **Dronpa-2** containing sample (**Dronpa-2**-labeled *Escherichia coli* or eucaryotic cells, or a **Dronpa-2** solution sandwiched between two glass-slides with a 80-100 μm-thick spacer) in the raster scanning mode (128 $\times$ 128 pixel^2; pixel size: 0.4 μm; dwell time τ_{dt}: 3.4 μs; λ_{exc} = 488 nm; 500 nm $\leq \lambda_{\text{em}} \leq$ 600 nm) with the focused 488 nm laser of the confocal microscope. The image of fixed **Dronpa-2**-labeled cells at the nucleus displayed in Figure S16a has been obtained with an objective Plan-NeoFluar 20$\times$, NA 0.5; over a 53 $\times$ 53 μm^2 square at 5% light power associated to 1.76 μW measured in the focal plane of the objective with the powermeter.

2. Average the fluorescence signal over square zone of interest located in the center of the collected images (typically a 3×3 μm^2 zone when using the **Dronpa-2** solution or the cell nucleus for the **Dronpa-2**-labeled cells at the nucleus displayed in Figure S16a respectively).

3. Plot the dependence of the averaged fluorescence signal as a function of time, which was extracted from the image rank n as $n\tau_{dt}$. Figure S16b displays the average drop over the nucleus. It also shows that the **Dronpa-2** aqueous solution sandwiched between two glass-slides yields a similar kinetic signature upon properly restricting analysis to a central portion of the overall image, which avoids molecular diffusion to interfere with the **Dronpa-2** photoconversion.

[10] It has been demonstrated that the kinetics of the time evolution of the fluorescence decay is similar in the successive pulses.[7]

[11] We typically used a camera set to 3 Hz over 10 min. Before further processing, the acquired video images can be downsized by a 3×3 averaging kernel to improve the signal-to-noise ratio. A mask of the illuminated area was evaluated by exploiting a threshold of the intensity from the average fluorescence image and all the following operations were performed on the unmasked pixels only.

4. Extract the maps of the characteristic time τ (Figure S16c) and light intensity at 488 nm delivered by the focused laser beam at the focal plane of the confocal microscope (Figure S16d) upon exploiting Eq.(S8) and the relevant parameters for **Dronpa-2** in Table S5. Representative histograms in the field of view are displayed in Figures S16e and f respectively.

5. To calibrate the overall scale of light intensities, reproduce the protocol described above at different settings of the instrument to be calibrated.

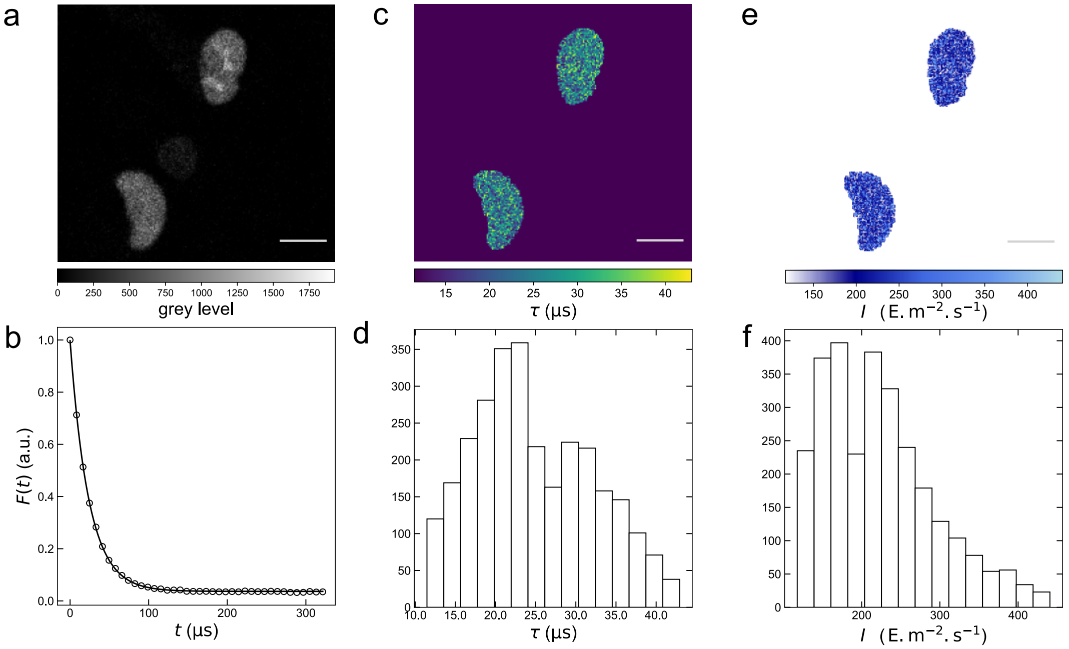

Figure S16: ***Dronpa-2*** *for characterization of the spatial distribution of the light intensity of a confocal microscope equipped with a continuous laser.* **a**,**b**: **Dronpa-2**-labeled nucleus of a fixed U-2 OS cell imaged with a confocal microscope in the raster scanning mode (λ_{exc} = 488 nm; 500 nm $\leq \lambda_{\mathrm{em}} \leq$ 600 nm). Initial image (**a**); Time evolution of the averaged fluorescence over the whole nucleus (**b**; triangles: experimental data; solid line: monoexponential fit with Eq.(S1). The corresponding evolution from a central portion of the overall image of a 10 μM **Dronpa-2** solution sandwiched between two glass-slides is shown with circles); **c**–**f**: Maps of the characteristic time τ (**c**) and light intensity (**d**), and corresponding histograms (**e**,**f**; a 3×3 binning is applied to the initial video sequence to improve fitting accuracy). Solvent: Tris buffer pH 7.4 (50 mM Tris, 150 mM NaCl); Pixel size: 0.33 μm; Laser power 10%; T = 293 K; Scale bar: 29 μm; Independent repeats: 4

.

Confocal microscope equipped with a pulsed 488 nm laser Same as paragraph 2.3.3 upon acquiring 50–100 square images of the **Dronpa-2** containing sample (**Dronpa-2**-labeled *Escherichia coli* or eucaryotic cells, or a **Dronpa-2** solution sandwiched between two glass-slides with a 80-100 μm-thick spacer) in the raster scanning mode (512 × 512 pixel^2; pixel size: from 0.057 to 0.569 μm; dwell time τ_{dt}: 1.2 μs; λ_{exc} = 488 nm; 500 nm $\leq \lambda_{\mathrm{em}} \leq$ 600 nm) with the focused 488 nm laser of the confocal microscope. The image of fixed **Dronpa-2**-labeled cells at the nucleus displayed in Figure S16a has been obtained with an objective HC PL APO CS2; Leica 40×, NA 1.1; over a 29.12 × 29.12 μm^2 to

291.19×291.19 μm^2 square at 2% light power associated to 18.5 μW measured in the focal plane of the objective with the powermeter.

2.3.4 Troubleshooting

General

- No fluorescence decay is observed: Light intensity at 488 nm is too low or the **Dronpa-2** sample is already photoswitched. In the first case, increase light intensity at 488 nm. In the second case, refresh the **Dronpa-2** sample in darkness for half an hour.

- For calibration of light intensity in fluorescence imaging, Eq.(S1) becomes Eq.(S10)

$$F\left(\lambda_{\text{exc}}, \lambda_{\text{em}}, \overrightarrow{r}, t\right) = F\left(\lambda_{\text{exc}}, \lambda_{\text{em}}, \overrightarrow{r}, \infty\right) + \left[F\left(\lambda_{\text{exc}}, \lambda_{\text{em}}, \overrightarrow{r}, 0\right) - F\left(\lambda_{\text{exc}}, \lambda_{\text{em}}, \overrightarrow{r}, \infty\right)\right] \exp\left(-\frac{t}{\tau\left(\overrightarrow{r}\right)}\right) \quad \text{(S10)}$$

  which yields the map $I\left(\overrightarrow{r}\right)$ sought for from Eq.(S11)

$$I\left(\overrightarrow{r}\right) = \frac{1}{\sigma\tau\left(\overrightarrow{r}\right)} \quad \text{(S11)}$$

  Eqs.(S10,S11) are strictly valid when the actinometer molecules do not move. However, when they can diffuse (diffusion coefficient D), Eqs.(S10,S11) can still be used but the spatial resolution of the $I\left(\overrightarrow{r}\right)$ is then limited to $\sqrt{D\tau\left(\overrightarrow{r}\right)}$:[7] The faster the photoconversion, the higher the spatial resolution. At the limit of homogenization (e.g. upon stirring), the spatial information of the illumination profile is lost and one can retrieve the overall amount of light intensity I received by the actinometer solution from the measured overall relaxation time τ with Eq.(S6).

Epifluorescence microscope

- The fluorescence decays are not similar when using a train of light pulses to benefit from additional data: Check that the delay between two light pulses is long enough to yield significant recovery of the bright state of **Dronpa-2** after light-driven photoswitching.[12]

Confocal microscopy

- Figure S16b evidences the similarity of the characteristic times, which have been retrieved by using both **Dronpa-2** containing samples. This observation was anticipated from the previous observations pointing on similar cross sections for photoisomerization of **Dronpa-2** at 488 nm in solution and in fixed cells.[7] However, it also validated the choice of the acquisition parameters, which limited the interference of molecular diffusion for retrieving the characteristic time of **Dronpa-2** photoconversion. Indeed, whereas the **Dronpa-2** molecules do not move in the fixed cell, they can freely diffuse in the **Dronpa-2** solution. Hence, in the latter case, the square imaged surface

[12] The relaxation time for thermal recovery of the bright state of **Dronpa-2** in the dark is 50 s at 25 °C.

has to be chosen large enough in order to keep a central zone where the interference of molecular diffusion can be neglected for retrieving the characteristic time of **Dronpa-2** photoconversion from the light-induced decay of fluorescence signal upon illumination.[7] For building Figure S16b, we recorded 100 scans with a delay of 56 ms between two scans at a same pixel, which yielded 5.6 s for the total duration of the acquisition. During that time, the typical distance randomly visited by the **Dronpa-2** molecule was about 20 μm,[13] which is to be compared with the 53 μm-long side of the imaged square surface so as to reduce the analyzed surface not experiencing any interference from diffusion in the (x,y) plane to a 3 $\times$ 3 μm^2 square zone of interest located in the center of the collected images.[14] As shown in Figure S17, an analysis performed over the whole acquired image evidences the interference of **Dronpa-2** diffusion, which can be observed by the departure from a monoexponential decay at the longest times.[7] Nevertheless, the monoexponential fit still provides a satisfactory order of magnitude of the light intensity. Hence 39 μs was extracted for the characteristic time τ from the latter analysis whereas 34 μs was retrieved from the analysis on a 3 $\times$ 3 μm^2 square zone of interest located in the center of the collected images.

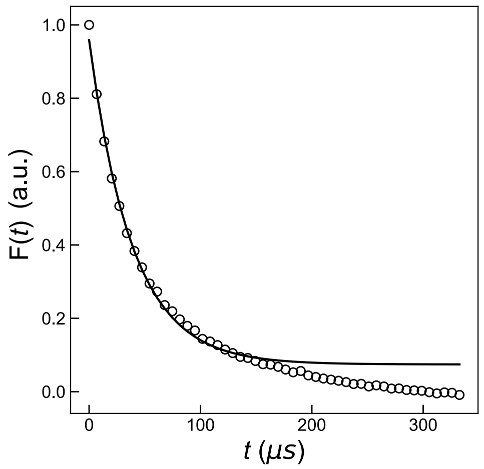

Figure S17: *Evidence for the interference of molecular diffusion in the decay of fluorescence emission from a 5 μM **Dronpa-2** solution in DPBS $1\times$ pH 7.4 buffer submitted to 488 nm illumination.* The series of 100 images has been acquired over a 53 $\times$ 53 μm^2 square in 6 s in the raster scanning mode (Objective 20$\times$, NA 0.5; 128 $\times$ 128 pixel^2; pixel size: 0.4 μm; dwell time τ_{dt}: 3.4 μs) with the focused 488 nm laser of the confocal microscope (5% light power associated to 1.76 μW measured in the focal plane of the objective with the powermeter). The dependence of the fluorescence signal averaged over the whole 53 $\times$ 53 μm^2 square field of view is plotted as a function of time, which was extracted from the image rank n as $n\tau_{dt}$. Markers: Experimental data; solid line: Monoexponential fit with Eq.(S1) of the Main Text. $T = 293$ K.

2.3.5 Time taken

Once the **Dronpa-2** sample is available, the duration of the overall measurement is evaluated to less than 1 h:

[13] We here used the 9 10^{-11} m^2.s^{-1} value of the diffusion coefficient of EGFP to derive an estimate (available on the Bionumber web site).

[14] At the corresponding timescale, we did not notice any interference that could originate from diffusion along the z axis in the inhomogeneous light profile generated with the microscope objective.

1. Collection of the time evolution of the fluorescence emission: 30 min as an order of magnitude (depending on the light intensity)

2. Data processing: 15 min

2.3.6 Anticipated results

The output image of the light of intensity $I(470 \text{ nm}, \vec{r})$ in the field of view of the microscope from which one can subsequently retrieve the histogram of the spatial distribution of the light at 470 nm in the field of view of the microscope. The estimate of the achievable measurement uncertainty on I is 20%.

2.3.7 References

- About actinometry: H. J. Kuhn, S. E. Braslavsky, and R. Schmidt, Chemical Actinometry (IUPAC Technical Report), *Pure Appl. Chem.*, **2004**, *76*, 2105–2146.

- About fluorescence: B. Valeur, M.-N. Berberan-Santos, Molecular Fluorescence: Principles and Applications 2nd Edition, Wiley, **2012**.

- About **Dronpa-2**: **1**. R. Chouket, A. Pellissier-Tanon, A. Lahlou, R. Zhang, D. Kim, M.-A. Plamont, M. Zhang, X. Zhang, P. Xu, N. Desprat, D. Bourgeois, A. Espagne, A. Lemarchand, T. Le Saux, L. Jullien, Extra kinetic dimensions for label discrimination, *Nat. Commun.*, **2022**, *13*, 1482; **2**. J. Quérard, R. Zhang, Z. Kelemen, M.-A. Plamont, X. Xie, R. Chouket, I. Roemgens, Y. Korepina, S. Albright, E. Ipendey, M. Volovitch, H. L. Sladitschek, P. Neveu, L. Gissot, A. Gautier, J.-D. Faure, V. Croquette, T. Le Saux, L. Jullien, Resonant out-of-phase fluorescence microscopy and remote imaging overcome spectral limitations, *Nat. Commun.*, **2017**, *8*, 969.

- About data processing: Mechanistic reduction leading to monoexponential fit of the time evolution of the fluorescence signal: R. Chouket, A. Pellissier-Tanon, A. Lahlou, R. Zhang, D. Kim, M.-A. Plamont, M. Zhang, X. Zhang, P. Xu, N. Desprat, D. Bourgeois, A. Espagne, A. Lemarchand, T. Le Saux, L. Jullien, Extra kinetic dimensions for label discrimination, *Nat. Commun.*, **2022**, *13*, 1482.

2.4 Quantitation of non-monochromatic light sources with a fluorescent actinometer

2.4.1 Reagents

- Fluorescent actinometer. In the reported examples, we adopted **Nit** and **PA**

- Solvent to produce the solution of the fluorescent actinometer:

  - Spectrograde ethanol for **Nit**

  - Heterotrophic media TAP [15] for **PA**

[15] https://www.chlamycollection.org/methods/media-recipes/tap-and-tris-minimal/

2.4.2 Equipments

- Balance to prepare the solutions
- Brown glassware or Aluminium foil to keep the solutions in the darkness
- Spectral data available online $\mathrm{https://chart-studio.plotly.com/\sim Alienor134/\#/}$
- Fluorimeter or any optical instrument, which can measure and record the time evolution of the fluorescence signal from the fluorescent actinometer
- Quartz cuvette or glass microscope slides with a 100 μm spacer to build a chamber
- Software for fitting the time evolution of the fluorescence response to illumination

2.4.3 Procedure

Quantitation of a purple LED emitting at 405 nm We first report an example of such a measurement by using the combination of α-(p-dimethylaminophenyl)-N-phenylnitrone (**Nit**) and Rhodamine B (**RhB**) in ethanol as a fluorescent actinometer to characterize the emission spectrum of a Light Emitting Diode (LED) at 405 nm.

1. Acquire the LED emission spectrum, either as a reference from the purchaser or after a measurement with a spectrophotometer in which the the light emitted from the LED is sent into the emission pathway of a fluorometer with reduced slits opening. Figure S18a displays the unscaled emission spectrum $S(\lambda)$ of a Light Emitting Diode (LHUV-0405, Lumileds, NL), which emits at 405$\pm$15 nm.
2. Divide the curve $S(\lambda)$ by its integral S calculated over the wavelength range of LED emission to yield the normalized emission spectrum $j(\lambda) = S(\lambda)/S$ (Figure S18b).
3. The flux of photons arriving at the sample at each wavelength λ which is sought for, $I(\lambda)$, is proportional to $j(\lambda)$ but the proportionality factor S_I is unknown (see Eq.S38). To measure S_I, first compute the integral of the action spectrum AS of the LED on **Nit** in ethanol (see Eq.S40) by multiplying the absorption spectrum of the nitrone **Nit**, $\varepsilon(\lambda)$ (Figure S18c) by the normalized emission spectrum $j(\lambda)$ (Figure S18d) upon further using $\varphi = 0.17$ as an average over the concerned wavelength range (see Table S8).
4. On the other hand, submit a 12 μM **Nit** and 1 μM **RhB** solution in ethanol to illumination in a 3 mm optical path cuvette.
5. Record the rise of the fluorescence emission at 574 nm as a function of time.
6. Retrieve the relaxation time τ of the photoreaction from monoexponential fitting of the data (see Figure S18e).
7. Exploit the values of τ and AS to retrieve the scaling parameter $S_I = 1/(\tau AS)$.

8. Retrieve the scaled emission spectrum of the LED from $I(\lambda) = S_I \times j(\lambda)$ (see Figure S18f).

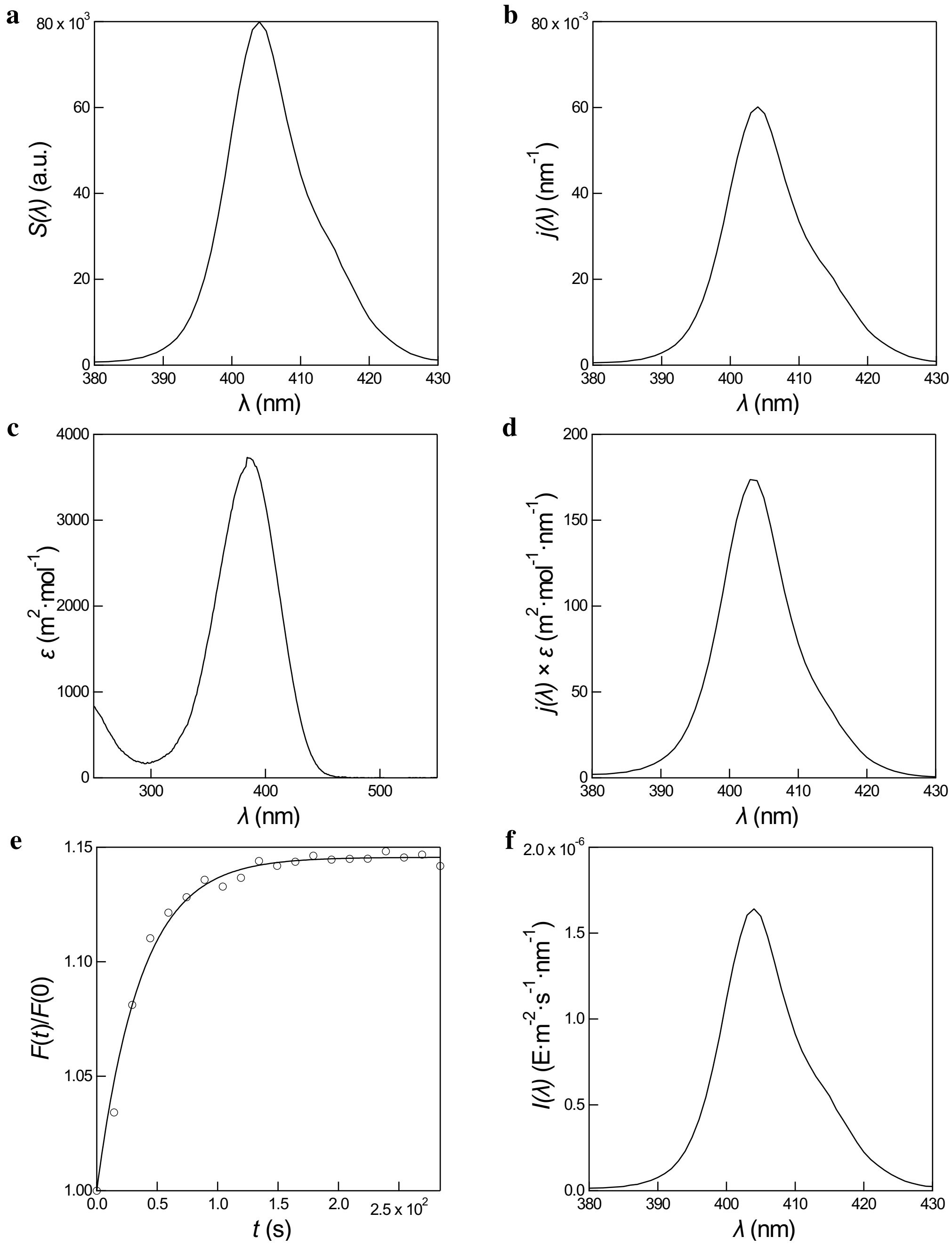

Figure S18: *Quantitation of a purple LED emitting at 405 nm.* Non-normalized ($S(\lambda)$; **a**) and normalized ($S(\lambda)/S$; **b**) emission spectrum of the LED at 405 nm; **c**: Absorption spectrum of **Nit** $\varepsilon(\lambda)$; **d**: Action spectrum of the LED on **Nit** in ethanol; **e**: Rise of the fluorescence emission of a 12 μM **Nit** and 1 μM **RhB** solution in ethanol at 574 nm as a function of time. Markers: experimental data; solid line: monoexponential fit. From the fit, we retrieved $\tau = 38.3$ s for the relaxation time. $T = 293$ K; **f**: Scaled emission spectrum of the LED.

Quantitation of a red-orange LED emitting at 625 nm We then report an example of measurement by using the **PA** fluorescent actinometer to calibrate the emission spectrum of a Light Emitting Diode (LED) at 625 nm mounted on

the FL 6000 fluorometer from Photon Systems Instruments (PSI; Drasov, Czech Republic) (LXML-PH01 LED, which belongs to the LUXEON Rebel Color Line from LUMILEDS). The following protocol has been reproduced for various percentages of light power of the PSI instrument, which led us to build the calibration curve displayed in Figure 4 of the Main Text.

1. Acquire the LED emission spectrum.

2. Divide the curve $S(\lambda)$ by its integral S calculated over the wavelength range of LED emission to yield the normalized emission spectrum $j(\lambda) = S(\lambda)/S$ (Figure S19a).

3. Multiply the scaled excitation spectrum of **PA**, $\sigma_{\text{mic}}(\lambda)$ (Figure S19b), by the normalized emission spectrum $j(\lambda)$ to generate the action spectrum of the LED on **PA**, $\sigma_{\text{mic}}(\lambda) \times j(\lambda)$ (Figure S19c).

4. Compute the integral AS of the action spectrum of the LED on **PA**.

5. On the other hand, submit a sample of *Chlamydomonas reinhardtii* to LED illumination in the fluorometer.

6. Record the rise of the fluorescence emission at 625 nm as a function of time (see Figure S19d).

7. Retrieve the relaxation time τ of the **PA** photoactivation from the fitting protocol reported in paragraph 2.1.3.

8. Exploit the values of τ and AS to retrieve the scaling parameter $S_I = 1/(\tau AS)$.

9. Retrieve the scaled emission spectrum of the LED from $I(\lambda) = S_I \times j(\lambda)$ (see Figure S19e).

2.4.4 Troubleshooting

Make sure that the suspension of algae is not sedimented.

2.4.5 Time taken

The duration of the overall measurement is evaluated to 2 h:

1. Collection of the unscaled LED spectrum: 30 min

2. Collection of the time evolution of the fluorescence emission under constant illumination: 30 min as an order of magnitude (depending on the light intensity)

3. Data processing: 60 min

2.4.6 Anticipated results

A quantitative spectral measurement of light intensity with achievable measurement uncertainty equal to 20% with **Nit** and 70% with **PA**.

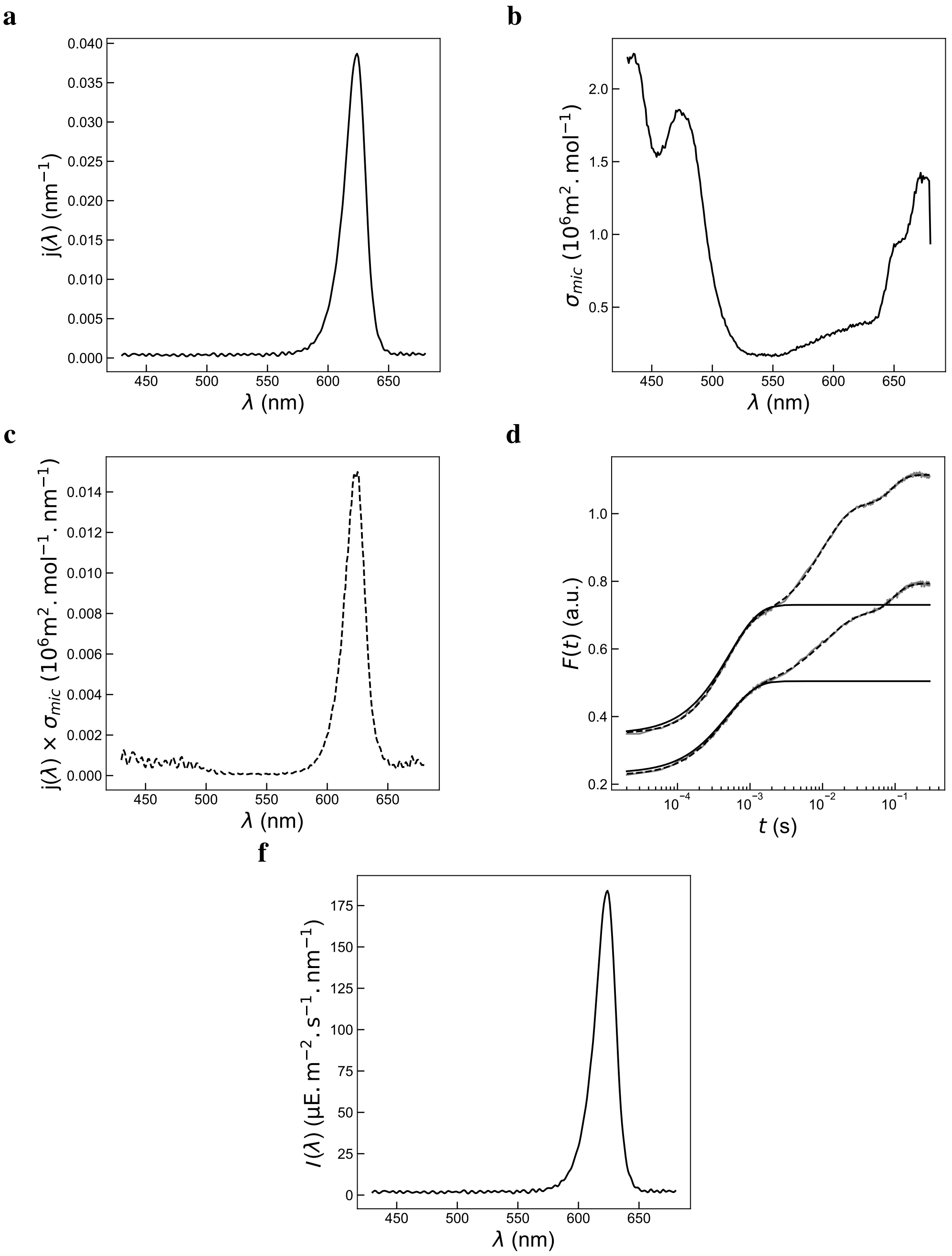

Figure S19: *Quantitation of a red-orange LED emitting at 625 nm.* **a**: Normalized emission spectrum ($j(\lambda) = S(\lambda)/S$) of the LED at 625 nm; **b**: Scaled fluorescence excitation spectrum $\sigma_{\mathrm{mic}}(\lambda)$ of **PA** in *Chlamydomonas reinhardtii (CC_124)* in exponential phase in minimal media ($\lambda_{\mathrm{em}} = 470$ nm); **c**: Action spectrum of the LED at 625 nm on **PA** in *Chlamydomonas reinhardtii (CC_124)*; **d**: Time evolution of the **PA** fluorescence emission under constant illumination at 625 nm for the power setting 60% on the PSI instrument (2 repeats). The experimental data (grey markers) have been fitted with Eqs.(S3) (dashed lines) and (S4) (solid lines) to retrieve $\tau = 427$ μs and $\tau = 460$ μs for the characteristic time τ associated to the initial step of **PA** fluorescence rise ; **e**: Scaled emission spectrum of the LED at 625 nm. $T = 293$ K.

2.4.7 References

- About actinometry: H. J. Kuhn, S. E. Braslavsky, and R. Schmidt, Chemical Actinometry (IUPAC Technical Report), *Pure Appl. Chem.*, **2004**, *76*, 2105–2146.

- About fluorescence: B. Valeur, M.-N. Berberan-Santos, Molecular Fluorescence: Principles and Applications 2nd Edition, Wiley, **2012**.

- About **Nit**: **1**. P. F. Wang, L. Jullien, B. Valeur, J.-S. Filhol, J. Canceill, J.-M. Lehn, Multichromophoric Cyclodextrins. 5. Antenna-induced Unimolecular Photoreactions. Photoisomerization of a Nitrone, *New J. Chem.*, **1996**, *20*, 895–907; **2**. M. Emond, T. Le Saux, S. Maurin, J.-B. Baudin, R. Plasson, L. Jullien, 2-Hydroxy-Azobenzenes to Tailor pH Pulses and Oscillations with Light, *Chem. Eur. J.*, **2010**, *16*, 8822–8831.

- About **PA**: **1**. Maxwell, K., and Johnson, G. N., Chlorophyll fluorescence – a practical guide, J. Exp. Bot, **2000**, *51*, 659–668; **2**. D. Lazar, The polyphasic chlorophyll a fluorescence rise measured under high intensity of exciting light, *Funct. Plant Biol.*, **2006**, *33*, 9 – 30.

- About data processing: **1**. Mechanistic reduction leading to monoexponential fit of the time evolution of the fluorescence signal: R. Chouket, A. Pellissier-Tanon, A. Lahlou, R. Zhang, D. Kim, M.-A. Plamont, M. Zhang, X. Zhang, P. Xu, N. Desprat, D. Bourgeois, A. Espagne, A. Lemarchand, T. Le Saux, L. Jullien, Extra kinetic dimensions for label discrimination, *Nat. Commun.*, **2022**, *13*, 1482; **2**. About **PA** data processing: D. Joly, R. Carpentier, Sigmoidal reduction kinetics of the photosystem II acceptor side in intact photosynthetic materials during fluorescence induction, *Photochem. Photobiol. Sci.*, **2009**, *8*, 167–173.

2.5 Quantitation of non-monochromatic light sources with a photochemically inert fluorophore

2.5.1 Reagents

- 7-Hydroxy-9H-(1,3-dichloro-9,9-dimethylacridin-2-one) (**DDAO**)

- Solvent to produce the **DDAO** solution:

  - Spectrograde DMSO

  - Aqueous HEPES pH 7.9 buffer (100 mM NaCl, 5 mM NaOH, 10 mM HEPES)

2.5.2 Equipments

- Balance to prepare the **DDAO** solution

- Brown glassware or Aluminium foil to keep the solutions in the darkness

- Spectral data available online $\mathrm{https://chart-studio.plotly.com/\sim Alienor134/\#/}$

- Fluorimeter or any optical instrument, which can measure and record the fluorescence signal

- Quartz cuvette or glass microscope slides with a 100 μm spacer to build a chamber

2.5.3 Procedure

- Record the emission spectrum $S(\lambda)$ of the white LED by following the protocol reported in the paragraph 7.2 upon using appropriate filters to avoid spectral overlaps with the fluorescence emission of **DDAO**. To perform this calibration with the epifluorescence microscope, we filtered the LED light before the sample with the same filters as for the spectrum measurement: a short-pass 694 filter (694/SP, AHF, FR) and a fluorescence dichroic 665 nm beamsplitter (FF665-Di02-25x36, Semrock, NY) to eliminate any contribution from the white LED signal that could overlap with **DDAO** fluorescence.

- Normalize the emission spectrum $S(\lambda)$ by its integral to yield the normalized emission spectrum $j(\lambda) = S(\lambda)/S$ (Figure S20a).

- Build the action spectrum of the white LED on **DDAO** from exploiting the excitation spectrum of the latter identical to its absorption spectrum (Figure S20b).

- Use Eq.(S79) to compute its integral AS_{abs}.

- On the other hand, collect the fluorescence signal from the same **DDAO** solution by using a band-pass 775/140 filter (775/140 single band-pass, Semrock, US) under two illuminations:

  - 470±10 nm LED of the epifluorescence microscope calibrated in intensity with **Dronpa-2** at various light intensities to investigate the linear dependence of the fluorescence level of **DDAO** $F\left(\lambda_{exc,1}\right)$ on the light intensity $I\left(\lambda_{\mathrm{exc},1}\right)$ at 470 nm (Figure S20c).
  - White LED at various feeding currents to record the dependence of its fluorescence level $F\left(\lambda_{\mathrm{min}};\lambda_{\mathrm{max}}\right)$ on the LED-feeding current.

Equipped with the molar absorption coefficient of **DDAO** at $\lambda_{\mathrm{exc},1}$, use Eq. (S77) to compute the $S_{I,0}$ value at various currents feeding the white LED. The resulting dependence of the scaled spectral light intensity of the white LED is displayed in Figure S20d.

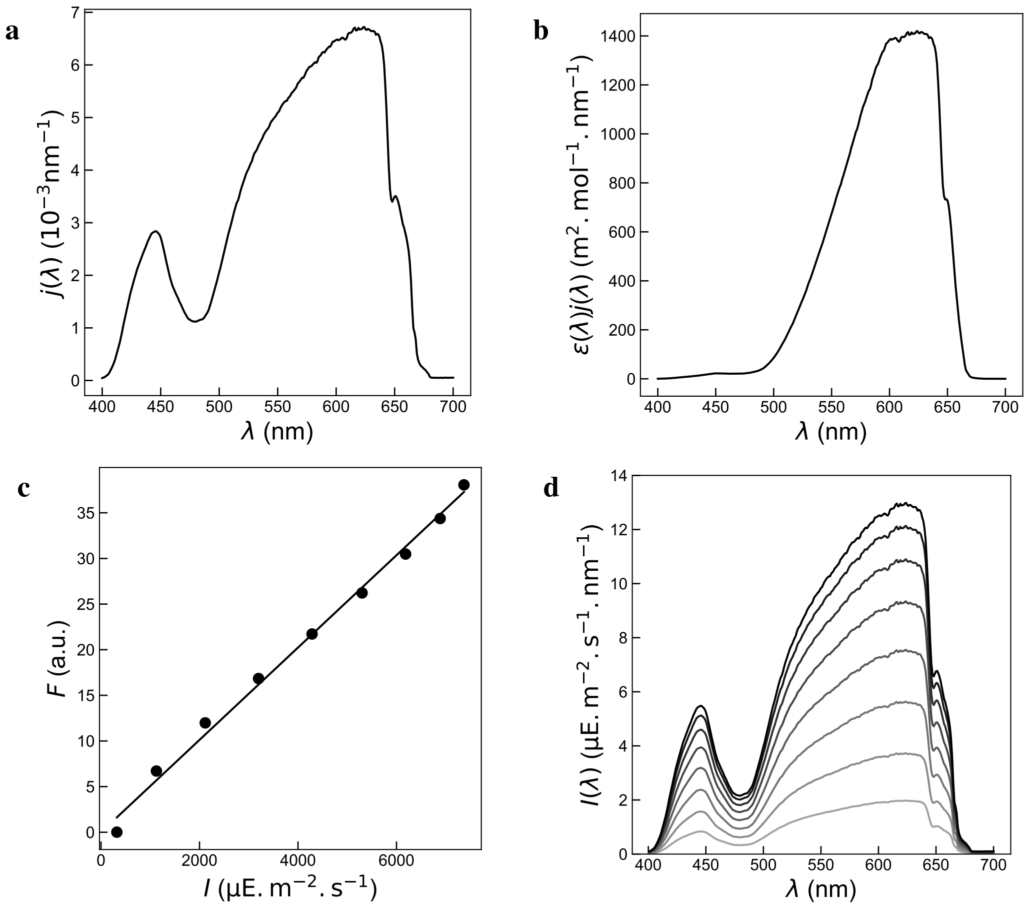

Figure S20: *Quantitation of a white LED with* ***DDAO***. **a**: Emission spectrum of the white LED normalized by its integral $j(\lambda) = S(\lambda)/S$; **b**: Action spectrum of the white LED on **DDAO** $\epsilon(\lambda)j(\lambda)$; **c**: Dependence of the fluorescence level of 10 μM **DDAO** in aqueous HEPES pH 7.9 buffer (100 mM NaCl, 5 mM NaOH, 10 mM HEPES) sandwiched between two microscope slides $F\left(\lambda_{\mathrm{exc},1}\right)$ on the light intensity $I\left(\lambda_{\mathrm{exc},1}\right)$ at 470 nm. Squares: experimental data from averaging the fluorescence collected by the camera, solid line: linear fit; **d**: Scaled spectral photon flux of the white LED for current levels feeding the white LED ranging from 55, 111, 166, 222, 277, 333, 388, 444 mA (light to dark). $T = 293$ K.

2.5.4 Troubleshooting

- Make sure that the same filter is used for the spectrum measurement of the light source and the set-up;
- Make sure the excitation light is properly filtered and not received by the detector for the two light sources used.

2.5.5 Time taken

The duration of the overall measurement is evaluated to 2 h:

1. Collection of the unscaled LED spectrum: 30 min
2. Acquisition of the DDAO fluorescence signals: 30 min
3. Data processing: 60 min

2.5.6 Anticipated results

A quantitative spectral measurement of light intensity with 20% achievable measurement uncertainty.

2.5.7 References

About **DDAO** as a dye:

- **1**. D. Warther, F. Bolze, J. Lonéard, S. Gug, A. Specht, D. Puliti, X.- H. Sun, P. Kessler, Y. Lutz, J.-L. Vonesch, B. Winsor, J.-F. Nicoud, M. Goeldner, Live-Cell One- and Two-Photon Uncaging of a Far-Red Emitting Acridinone Fluorophore, *J. Am. Chem. Soc.*, **2010**, *132*, 2585–2590; **2**. R. Labruère, A. Alouane, T. Le Saux, I. Aujard, P. Pelupessy, A. Gautier, S. Dubruille, F. Schmidt, L. Jullien, Self-immolation for uncaging with fluorescence reporting, *Angew. Chem. Int. Ed.*, **2012**, *51*, 9344–9347.

3 Supplementary Note 3: Glossary

- Actinometer: An actinometer is a system which can be used to directly measure light intensity by knowing the quantum yield of its photo-conversion, and following the time-course of the reaction extent on the application of light.

- Characteristic time (or relaxation time): Time retrieved by the fitting of a mono-exponential curve from the time evolution of the fluorescence emission signal, which reports on the actinometer photoconversion extent.

- Cross section of photoconversion: Measure of the molecular surface leading to the actinometer photoconversion after light absorption (often expressed in $m^2.mol^{-1}$).

- Fluorescence emission spectrum: A fluorescence emission spectrum is recorded by fixing the excitation wavelength and scanning the emission wavelength. Hence, it results in a plot of fluorescence intensity vs. emission wavelength.

- Fluorescence excitation spectrum: A fluorescence excitation spectrum is recorded by fixing the emission wavelength and scanning the excitation monochromator wavelength. Hence, the fluorescence excitation spectrum gives information about the wavelengths at which a sample absorbs light so as to emit at the single emission wavelength chosen for observation.

- Inner filter effect: The inner filter effect manifests itself in samples exhibiting an absorbance along the optical path typically exceeding 0.15. It results in a decay of the intensity of the excitation light in the sample. As a consequence, the fluorescence signal is attenuated and dominated by the sample surface. In addition, if the excitation and emission fluorescence spectra significantly overlap, the fluorescence emission can be reabsorbed by the sample itself, which results in spectral distortion and even in some cases complete loss of the fluorescence signal.

- Irradiance: Irradiance is the light flux received by a surface per unit area. The SI unit of irradiance is the watt per square metre. Alternatively, it is denominated photon flux density with mole of photon per unit of time per square metre (See Eq.(S12) for the energy conversion) units, which is wavelength independent. Irradiance is often called light intensity. Spectral irradiance is the irradiance of a surface per unit wavelength, which is commonly measured in watts per square metre per nanometre or in mole of photon per unit of time per square metre per nanometre as done in the Main Text.

- Quantum yield of fluorescence: The fluorescence quantum yield gives the probability of the excited state being deactivated by fluorescence rather than by another, non-radiative mechanism.

- Quantum yield of photoconversion: The quantum yield of photoconversion gives the probability of the excited state being deactivated by photoconversion rather than by another mechanism.

4 Supplementary Note 4: Conversion of energy units

In this manuscript, we provide the values of the light intensities in E.m^{-2}.s^{-1} (or mol. of photons.m^{-2}.s^{-1}). This unit is currently used in actinometry. However, it is not often used in other fields such as optical microscopy, in which the researchers prefer to adopt W.m^{-2}. We provide below the conversion between both units.

We consider a monochromatic light of wavelength λ_{exc}. Its values in E.m^{-2}.s^{-1} and W.m^{-2} are respectively denoted as $I(\lambda_{\text{exc}}, \text{E.m}^{-2}.\text{s}^{-1})$ and $I(\lambda_{\text{exc}}, \text{W.m}^{-2})$. The relation between $I(\lambda_{\text{exc}}, \text{E.m}^{-2}.\text{s}^{-1})$ and $I(\lambda_{\text{exc}}, \text{W.m}^{-2})$ is given in Eq.(S12)

$$I(\lambda_{\text{exc}}, \text{W.m}^{-2}) = \frac{hcN_A}{\lambda_{\text{exc}}} \times I(\lambda_{\text{exc}}, \text{E.m}^{-2}.\text{s}^{-1}) \approx 0.12 \times \frac{\text{I}(\lambda_{\text{exc}}, \text{E.m}^{-2}.\text{s}^{-1})}{\lambda_{\text{exc}}\,(\text{m})} \quad \text{(S12)}$$

with the Planck constant $h = 6.63\ 10^{-34}$ m^2.kg.s^{-1}, speed of light in a vacuum $c = 3.00\ 10^8$ m.s^{-1}, the Avogadro number $N_A = 6.02\ 10^{23}$ mol^{-1}, and where λ_{exc} is in m.

5 Supplementary Note 5: Theoretical derivation of the expressions for retrieving light intensity

5.1 First protocol: Kinetic analysis of an actinometer engaged in an irreversible photochemical reaction

We first perform the kinetic analysis of an actinometer engaged in an irreversible photochemical reaction, which is relevant of the **Cin**, **Nit**, and **PA**[16] actinometers.

5.1.1 The model

We consider mechanisms of light-driven conversions, which can be reduced to the irreversible photochemical reaction displayed in Figure S21.

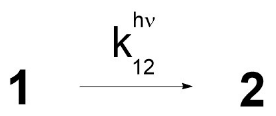

Figure S21: Reduced mechanism accounting for the photoactivation of an actinometer **1** leading to the irreversible formation of the product **2**.

Since most such light-driven conversions involve multiple steps, this assumption implies that the light intensity is low enough to make the photochemical activation step rate-limiting.

We here consider that the irreversible photochemical reaction is performed in a closed system (overall volume V).

Two asymptotic cases are considered:

[16] In fact, the kinetic model of the **PA** actinometer is more complex than the one developed below. However, we used Eq.(S24) to retrieve the cross section associated to the **PA** photoconversion.

- *Case 1*. The actinometer molecules do not diffuse and one can access the spatial profile of photon flux $\mathcal{I}(x, y, z)$ at coordinates (x, y, z) (given in mole of photons – or E – per unit of time) assumed to be constant in time;
- *Case 2*. The system can be considered homogeneous at any time of its evolution. This situation can result from either (i) homogeneously illuminating the whole system, (ii) stirring the sample content, or (iii) applying a non-homogeneous illumination profile on a small enough system such that molecular motion leading to homogeneous concentrations (by diffusion, convection,...) can be considered realized at any time of the photochemically-driven evolution of the whole system.[17] The whole system can then be considered to be submitted to an integrated photon flux (given in mole of photons – or E – per unit of time) constant in time and equal to $\mathcal{I} = \int_V \mathcal{I}(x, y, z)$.

Analyses of the temporal evolution of the concentration profiles in **1** and **2** with other boundary conditions can be found elsewhere.[7–9]

In the following, in a purpose of simplification, we adopt a unique notation, which has to be interpreted as accounting for the local (Case 1) or global (Case 2) time evolution of the concentrations and observables. Hence we rely on the mechanism displayed in Figure S21 to write Eq.(S13) describing the evolution of the concentrations :

$$\frac{d1}{dt} = -\frac{d2}{dt} = -k_{12}\,1 \tag{S13}$$

where we can make explicit the photochemical contribution to the rate constant by writing

$$k_{12} = k_{12}^{h\nu}. \tag{S14}$$

We first derive the theoretical expressions of the concentrations in **1** and **2** (denoted 1 and 2 respectively) upon applying a light jump and establish the relation existing between the illumination time associated to the photochemical reaction and the photophysical and photochemical parameters associated to the photo-activation step. Then we analyze the time evolution of the absorbance and intrinsic/extrinsic fluorescence emission of the actinometer under illumination.

5.1.2 Light-jump experiments with monochromatic illumination

Expression of the concentrations We consider that the system initially contains the actinometer **1** at concentration S_{tot}. The system is "suddenly"[18] illuminated, so that the incident photon flux varies from 0 to $\mathcal{I}(x, y, z)$ (Case 1), or from 0 to $\mathcal{I}$ (Case 2). The rate constant $k_{12} = k_{12}^{h\nu}$ can be considered constant. Upon writing $S_{\text{tot}} = 1 + 2$, Eq.(S13) yields:

$$1 = S_{\text{tot}} \exp\left(-\frac{t}{\tau}\right) \tag{S15}$$

$$2 = S_{\text{tot}} \left[1 - \exp\left(-\frac{t}{\tau}\right)\right] \tag{S16}$$

[17]This condition is fulfilled when $\tau_V \ll \tau$ where τ_V and τ respectively designate the time associated to molecular motion and the photochemical reaction over the whole system volume V.

[18]Note that "suddenly" here refers to a time interval such that Eq.(S13) can be considered valid.

where

$$\tau = \frac{1}{k_{12}} \tag{S17}$$

designates the local (Case 1) or global (Case 2) relaxation time associated to the photochemical reaction in the presence of light at constant photon flux $\mathcal{I}(x, y, z)$ (Case 1) or $\mathcal{I}$ (Case 2).

The reaction rate for the photo-consumption of the actinometer **1** is proportional to the local (Case 1) or global (Case 2) photon flux of the monochromatic excitation light absorbed by **1** at the excitation wavelength – written in both cases as, $\mathcal{I}_{\mathrm{abs}}(\lambda_{\mathrm{exc}})$, and to the unitless photo-consumption cross section, φ_{12}, which measures the probability of **1** photoactivation leading to **2** production after **1** light absorption:

$$-\frac{d1}{dt} = \frac{\mathcal{I}_{\mathrm{abs}}(\lambda_{\mathrm{exc}})\varphi_{12}}{V} \tag{S18}$$

where V is the irradiated volume (expressed in L or dm^3) and $\mathcal{I}_{\mathrm{abs}}(\lambda_{\mathrm{exc}})$ is expressed in E per unit of time.

$\mathcal{I}_{\mathrm{abs}}(\lambda_{\mathrm{exc}})$ is given by Eq.(S19)

$$\mathcal{I}_{\mathrm{abs}}(\lambda_{\mathrm{exc}}) = \frac{A_1(\lambda_{\mathrm{exc}})}{A_{\mathrm{tot}}(\lambda_{\mathrm{exc}})}\mathcal{I}(\lambda_{\mathrm{exc}}) \tag{S19}$$

where $A_1(\lambda_{\mathrm{exc}})$ is the absorbance of **1**, $A_{\mathrm{tot}}(\lambda_{\mathrm{exc}})$ is the total absorbance, and $\mathcal{I}(\lambda_{\mathrm{exc}})$ is the photon flux absorbed by the solution at the excitation wavelength. According to the Beer-Lambert law, the latter can be written

$$\mathcal{I}(\lambda_{\mathrm{exc}}) = \mathcal{I}_0(\lambda_{\mathrm{exc}})\left[1 - \exp\left(-2.3A_{\mathrm{tot}}(\lambda_{\mathrm{exc}})\right)\right] \tag{S20}$$

where $\mathcal{I}_0(\lambda_{\mathrm{exc}})$ is the photon flux of the incident beam at the excitation wavelength.

We further consider that an actinometer is illuminated with a light source that perpendicularly illuminates a cuvette filled with its solution. Upon introducing the length of the optical pathlength ℓ and the scaled molar excitation (leading to light absorption by the actinometer[19]) coefficient $\epsilon_1(\lambda_{\mathrm{exc}})$ (expressed in m^2.mol^{-1}) at the excitation wavelength,[20] Eq.(S18) yields

$$-\frac{d1}{dt} = \epsilon_1(\lambda_{\mathrm{exc}})\mathcal{I}_0(\lambda_{\mathrm{exc}})\varphi_{12}\ell\frac{1 - exp\left(-2.3A_{\mathrm{tot}}(\lambda_{\mathrm{exc}})\right)}{A_{\mathrm{tot}}(\lambda_{\mathrm{exc}})V}1. \tag{S21}$$

If the total absorbance $A_{\mathrm{tot}}(\lambda_{\mathrm{exc}})$ is lower than 0.15, the kinetics is first order (at first order[10]) with a rate constant k_{12} given in Eq.(S22)

$$k_{12} = 2.3\epsilon_1(\lambda_{\mathrm{exc}})\varphi_{12}\frac{\mathcal{I}_0(\lambda_{\mathrm{exc}})\ell}{V} = 2.3\epsilon_1(\lambda_{\mathrm{exc}})\varphi_{12}I_0(\lambda_{\mathrm{exc}}) \tag{S22}$$

upon introducing the incident light intensity $I_0(\lambda_{\mathrm{exc}})$ (expressed in E.m^{-2}.s^{-1}).

[19]When energy transfer can occur from a light-collecting antenna towards the actinometer chromophore (like in the actinometer **PA**), the molar absorption coefficient $\varepsilon_1(\lambda_{\mathrm{exc}})$ has to be replaced by the excitation coefficient $\epsilon_1(\lambda_{\mathrm{exc}})$, which take into account the contribution of the antenna towards light absorption. For a sake of simplicity, we have only retained the notation $\epsilon_1(\lambda_{\mathrm{exc}})$ in the derivation.

[20]Note that the unit of $\varepsilon(\lambda)$ is in m^2.mol^{-1}. One has: $\varepsilon(\lambda)$(m^2.mol^{-1})=0.1$\times\varepsilon(\lambda)$(mol.L^{-1}.cm^{-1}).

Eq. (S22) can be alternatively written by introducing the cross section for the photochemical reaction $\sigma_{12}(\lambda_{\text{exc}})$

$$\sigma_{12}(\lambda_{\text{exc}}) = 2.3\epsilon_1(\lambda_{\text{exc}})\varphi_{12} \tag{S23}$$

which yields

$$k_{12} = \frac{1}{\tau} = \sigma_{12}(\lambda_{\text{exc}})I_0(\lambda_{\text{exc}}). \tag{S24}$$

Eq.(S22) shows that the rate constant k_{12} can be evaluated from the knowledge of the photophysical and photochemical parameters, $\epsilon_1(\lambda_{\text{exc}})$ and φ_{12} or $\sigma_{12}(\lambda_{\text{exc}})$, as soon as $I_0(\lambda_{\text{exc}})$ is known. Conversely, $I_0(\lambda_{\text{exc}})$ can be retrieved from Eq.(S25) from the knowledge of $\epsilon_1(\lambda_{\text{exc}})$ and φ_{12} or $\sigma_{12}(\lambda_{\text{exc}})$, and the relaxation time of the photochemical reaction τ.

$$I_0(\lambda_{\text{exc}}) = \frac{k_{12}}{2.3\epsilon_1(\lambda_{\text{exc}})\varphi_{12}} = \frac{1}{2.3\epsilon_1(\lambda_{\text{exc}})\varphi_{12}\tau} = \frac{1}{\sigma_{12}(\lambda_{\text{exc}})\tau} \tag{S25}$$

In order to simplify the notations, we identified $I_0(\lambda_{\text{exc}})$ to $I(\lambda_{\text{exc}})$ and $\sigma_{12}(\lambda_{\text{exc}})$ to $\sigma(\lambda_{\text{exc}})$ for the actinometers engaged in an irreversible photoconversion in the Main Text.

When the monochromatic illumination is focussed such that photo-activation occurs only within a sub-volume V_{exc} of the total volume V and provided that molecular motion over the whole system volume is fast at the time scale of its photoconversion, one has:

$$k_{12} = \frac{V_{\text{exc}}}{V}k_{\text{V}_{\text{exc}}} \tag{S26}$$

where $k_{\text{V}_{\text{exc}}}$ designates the rate constant associated to photoactivation within the volume V_{exc}.

Expression of the absorbance The time evolution of the absorbance $A(\lambda_{\text{exc}})$ at the excitation wavelength λ_{exc} is expressed in Eq.(S27)

$$A(\lambda_{\text{exc}}) = [\varepsilon_1(\lambda_{\text{exc}})1 + \varepsilon_2(\lambda_{\text{exc}})2]\,\ell = \left\{[\varepsilon_1(\lambda_{\text{exc}}) - \varepsilon_2(\lambda_{\text{exc}})]\exp\left(-\frac{t}{\tau}\right) + \varepsilon_2(\lambda_{\text{exc}})\right\} S_{\text{tot}}\ell \tag{S27}$$

where $\varepsilon_1(\lambda_{\text{exc}})$ and $\varepsilon_2(\lambda_{\text{exc}})$ designate the molar absorption coefficients of **1** and **2** respectively and ℓ is the optical pathlength.

In particular, when $\varepsilon_2(\lambda_{\text{exc}})$ is vanishing (as with the nitrone actinometer), Eq.(S27) yields

$$A(\lambda_{\text{exc}}) = \varepsilon_1(\lambda_{\text{exc}})S_{\text{tot}}\ell\exp\left(-\frac{t}{\tau}\right). \tag{S28}$$

As shown in Eqs.(S27,S28), the relaxation time τ can be simply extracted from a robust monoexponential fit of the temporal evolution of the absorbance signal.

Expression of the fluorescence We consider two cases where fluorescence can be used to report on the time evolution of the illuminated actinometer:

- We first consider the case of bright states **1** and **2**;
- We then consider the case of adding a fluorophore, which reports on the time evolution of the illuminated actinometer by the inner filter effect (see below).

Intrinsic fluorescence reporting When **1** and **2** are intrinsically bright, the temporal dependence of the fluorescence emission $F(t)$ originates from summing the individual contributions of **1** and **2**. Denoting $Q_i = \epsilon_{\mathrm{i}}(\lambda_{\mathrm{exc}})\varphi_{\mathrm{F,i}}$ for the molecular brightness ($\varphi_{\mathrm{F,i}}$ designate the quantum yield of fluorescence of the species **i**), one has

$$F(t) \quad = \quad (Q_1 1 + Q_2 2)\, I = \left[(Q_1 - Q_2)\, S_{\mathrm{tot}} \exp\left(-\frac{t}{\tau}\right) + Q_2 S_{\mathrm{tot}}\right] I. \qquad \text{(S29)}$$

Then, as shown in Eq.(S29), the relaxation time τ can be simply extracted from a robust monoexponential fit of the temporal evolution of the fluorescence signal.

Extrinsic fluorescence reporting When **1** and **2** are not fluorescent, one can add a fluorophore to report with fluorescence on the time evolution of the absorbance driven by the time evolution of the illuminated actinometer. To illustrate the corresponding principle of inner filter effect, we analyze the following model.

We consider that a light beam of wavelength λ_{exc} and intensity $I(\lambda_{\mathrm{exc}})$ perpendicularly illuminates a pathlength ℓ. At the abscissa x, the excitation light intensity is equal to $I(\lambda_{\mathrm{exc}}) \exp\left[-2.3 A_{\mathrm{tot}}(\lambda_{\mathrm{exc}}) x/\ell\right]$ where $A_{\mathrm{tot}}(\lambda_{\mathrm{exc}})$ is the total absorbance of the sample along the pathlength ℓ. The light absorbed by the reporting fluorophore **F** in a thin slice of thickness dx is $1 - \exp\left[-2.3 A_{\mathbf{F}}(\lambda_{\mathrm{exc}}) dx/\ell\right] \approx 2.3 A_{\mathbf{F}}(\lambda_{\mathrm{exc}}) dx/\ell$ with $A_{\mathbf{F}}$ the fluorophore absorbance of the cuvette which is assumed to be small. Then the total fluorescence emission from the reporting fluorophore collected at the wavelength λ_F at the detecting element can be written

$$F(\lambda_{\mathrm{exc}}, \lambda_F) = \int_0^{\ell} \alpha(x) F(\lambda_F) \times I(\lambda_{\mathrm{exc}}) \exp\left[-2.3 A_{\mathrm{tot}}(\lambda_{\mathrm{exc}}) x/\ell\right] \times 2.3 A_{\mathbf{F}}(\lambda_{\mathrm{exc}}) dx/\ell \qquad \text{(S30)}$$

where $\alpha(x)$ is a collecting factor and $F(\lambda_F)$ the fluorescence intensity per absorbed photon at the wavelength λ_F. Assuming that $\alpha(x)$ only weakly depends on x in the considered geometry, we finally derive

$$F(\lambda_{\mathrm{exc}}, \lambda_F) = C \frac{1 - \exp\left(-2.3 A_{\mathrm{tot}}(\lambda_{\mathrm{exc}})\right)}{2.3 A_{\mathrm{tot}}(\lambda_{\mathrm{exc}})} \qquad \text{(S31)}$$

where

$$C = 2.3 \alpha F(\lambda_F) I(\lambda_{\mathrm{exc}}) A_{\mathbf{F}}(\lambda_{\mathrm{exc}}) \qquad \text{(S32)}$$

which is a constant during a given experiment.

When $A_{\mathrm{tot}}(\lambda_{\mathrm{exc}}) < 0.15$, we develop the exponential term in Eq.(S31) at the second order around zero and obtain Eq.(S33)

$$F(\lambda_{\mathrm{exc}}, \lambda_F) = C\left(1 - \frac{2.3 A_{\mathrm{tot}}(\lambda_{\mathrm{exc}})}{2}\right) \qquad \text{(S33)}$$

When $A_{\mathbf{F}} \ll A_{\mathrm{tot}}(\lambda_{\mathrm{exc}})$, we use Eq.(S27) and derive Eq.(S34)

$$F(\lambda_{\mathrm{exc}}, \lambda_F) = C\left[1 - \frac{2.3}{2}\left(\left[\varepsilon_1(\lambda_{\mathrm{exc}}) - \varepsilon_2(\lambda_{\mathrm{exc}})\right] S_{\mathrm{tot}} \exp\left(-\frac{t}{\tau}\right) + \varepsilon_2(\lambda_{\mathrm{exc}}) S_{\mathrm{tot}}\right) \ell\right] \qquad \text{(S34)}$$

reducing to Eq.(S35)

$$F(\lambda_{\mathrm{exc}}, \lambda_F) = C\left[1 - \frac{2.3}{2}\left(\varepsilon_1(\lambda_{\mathrm{exc}}) S_{\mathrm{tot}} \exp\left(-\frac{t}{\tau}\right)\right) \ell\right] \qquad \text{(S35)}$$

when $\varepsilon_2(\lambda_{\text{exc}})$ is vanishing (as with the nitrone actinometer).

Eqs.(S34,S35) demonstrates that τ can then be retrieved from analyzing the time evolution fluorescence of the reporting fluorophore **F**.

5.1.3 Light-jump experiments with non-monochromatic illumination

We now consider a light source associated with a spectral light intensity $I(\lambda)$ (expressed in E.m^{-2}.s^{-1}.nm^{-1}) spread over [λ_{min};λ_{max}] (with the wavelength expressed in nm). It perpendicularly illuminates a cuvette filled with an actinometer associated to a scaled excitation spectrum leading to its light absorption $\epsilon_1(\lambda)$ (expressed in m^2.mol^{-1}). Following from Eqs.(S22–S24), the rate constant k_{12} for the actinometer photoconversion as well as the associated characteristic time τ obey Eq.(S36)

$$k_{12} = \frac{1}{\tau} = 2.3 \int_{\lambda_{\text{min}}}^{\lambda_{\text{max}}} \epsilon_1(\lambda)\phi_{12}(\lambda)I(\lambda)d(\lambda) = \int_{\lambda_{\text{min}}}^{\lambda_{\text{max}}} \sigma_{12}(\lambda)I(\lambda)d(\lambda) \quad \text{(S36)}$$

where $\phi_{12}(\lambda)$ and $\sigma_{12}(\lambda)$ designate the quantum yield and the cross section associated with the photoconversion.

We introduce the normalized emission spectrum $j(\lambda) = I(\lambda)/S_I$, where S_I designates the integral of $I(\lambda)$ over [λ_{min};λ_{max}] (Eq.(S38))

$$\int_{\lambda_{\text{min}}}^{\lambda_{\text{max}}} I(\lambda)d(\lambda) = S_I \quad \text{(S37)}$$

and the integral of $j(\lambda)$ over the same wavelength range is equal to one (Eq.(S38))

$$\int_{\lambda_{\text{min}}}^{\lambda_{\text{max}}} j(\lambda)d(\lambda) = 1 \quad \text{(S38)}$$

Eq.(S36) yields Eq.(S39)

$$k_{12} = \frac{1}{\tau} = 2.3 S_I \int_{\lambda_{\text{min}}}^{\lambda_{\text{max}}} \epsilon_1(\lambda)\phi(\lambda)j(\lambda)d(\lambda) = S_I \int_{\lambda_{\text{min}}}^{\lambda_{\text{max}}} \sigma_{12}(\lambda)j(\lambda)d(\lambda) \quad \text{(S39)}$$

From computing the integral of the action spectrum AS of the light source given in Eq. (S40)

$$AS = \int_{\lambda_{\text{min}}}^{\lambda_{\text{max}}} \sigma_{12}(\lambda)j(\lambda)d(\lambda) \quad \text{(S40)}$$

and measuring the characteristic time τ, one can extract the integral $S_I = 1/(\tau AS)$ and retrieve the scaled spectral light intensity $I(\lambda) = S_I \times j(\lambda)$ sought for given in Eq.(S71)

$$I(\lambda) = \frac{1}{\left(\int_{\lambda_{\text{min}}}^{\lambda_{\text{max}}} \sigma_{12}(\lambda)j(\lambda)d(\lambda)\right) \times \tau} j(\lambda) \quad \text{(S41)}$$

Eq.(S71) bears much similarity to Eq.(S25) except for a convolution of the cross section with the spectral light intensity.

In practice, one has the dependence of $\epsilon_1(\lambda)\phi(\lambda)$ or $\sigma_{12}(\lambda)$ on the wavelength and an unscaled emission spectrum of the light source $S(\lambda)$ (expressed in arbitrary unit) and the goal is to scale it to retrieve the spectral light intensity $I(\lambda)$

(expressed in E.m^{-2}.s^{-1}.nm^{-1}) at the sample. The scaling step first involves the normalization of the unscaled emission spectrum $S(\lambda)$ by its integral S (Eq.(S42))

$$\int_{\lambda_{\min}}^{\lambda_{\max}} S(\lambda)d(\lambda) = S \tag{S42}$$

to yield the normalized emission spectrum $j(\lambda) = S(\lambda)/S$. Then one proceeds as reported above to first compute the integral of the action spectrum AS of the light source given in Eq.(S40), and then retrieve the scaled spectral light intensity $I(\lambda) = S_I \times j(\lambda)$ sought for from the measured characteristic time τ and the integral $S_I = 1/(\tau AS)$.

5.2 First protocol: Kinetic analysis of an actinometer engaged in a reversible photochemical reaction

The actinometers engaged in an irreversible photochemical reaction benefit from simple kinetic laws. However, they can be used only once. Hence, we additionally performed the kinetic analysis of an actinometer engaged in a reversible photochemical reaction, which is relevant of the **Dronpa-2** and **DASA** actinometers.

5.2.1 The model

We now consider relaxation mechanisms, which can be reduced to the reversible photochemical reaction displayed in Figure S22. In particular this model is often relevant to account for the behavior of reversibly photoswitchable actinometers such as the reversibly photoswitchable fluorescent proteins or synthetic photochromes.

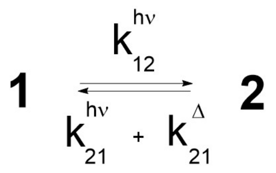

Figure S22: Reduced mechanism accounting for the photochemical behavior of a reversibly photoswitchable actinometer. The most stable state of the actinometer is assumed to be **1**, which can switch to the less stable state **2** by photoisomerization. Back reaction from **2** to **1** can occur either by photoisomerization or thermally-driven exchange.

The frame of assumptions is the same as the one exposed in the preceding section. Relying on the mechanism displayed in Figure S22, we write Eqs.(S43–S44) describing the concentration evolutions:

$$\frac{d1}{dt} = -k_{12}\,1 + k_{21}\,2 \tag{S43}$$

$$\frac{d2}{dt} = k_{12}\,1 - k_{21}\,2 \tag{S44}$$

where we make explicit the photochemical and thermal contributions to the rate constants by writing

$$k_{12} = k_{12}^{h\nu} \tag{S45}$$

$$k_{21} = k_{21}^{h\nu} + k_{21}^{\Delta} \tag{S46}$$

where the exponent indicates the nature of the contribution.

In the following, we derive the theoretical expressions of the concentrations in **1** and **2** and the intrinsic/extrinsic fluorescence intensity upon applying a light jump.

5.2.2 Light-jump experiments

Expression of the concentrations We consider that the system initially contains the actinometer **1** at concentration S_{tot}. The system is "suddenly"[21] illuminated so that the incident light intensity varies from 0 to $I(x, y, z)$ (Case 1), or from 0 to I (Case 2). The rate constant $k_{12} = k_{12}^{h\nu}$ and $k_{21} = k_{21}^{h\nu} + k_{21}^{\Delta}$ can be considered constant. Upon introducing the total concentration in actinometer $S_{\text{tot}} = 1 + 2$, Eqs.(S43,S44) yield:

$$-\frac{d\left(2 - 2^{\infty}\right)}{dt} = \left(k_{12} + k_{21}\right)\left(2 - 2^{\infty}\right) \tag{S47}$$

from which we derive

$$2 - 2^{\infty} = 1^{\infty} - 1 = -2^{\infty} \exp\left(-\frac{t}{\tau}\right) \tag{S48}$$

where

$$2^{\infty} = \frac{K_{12}}{1 + K_{12}} S_{\text{tot}} \tag{S49}$$

$$1^{\infty} = \frac{1}{1 + K_{12}} S_{\text{tot}} \tag{S50}$$

$$\tau = \frac{1}{k_{12} + k_{21}} \tag{S51}$$

$$K_{12} = \frac{k_{12}}{k_{21}} \tag{S52}$$

In Eqs.(S48–S52), 2^{∞}, 1^{∞}, τ, and K_{12} respectively denote the steady-state value of the concentrations in **2** and **1**, the relaxation time associated to the reversible exchange between the states **1** and **2**,[22] and the photoisomerization constant of the actinometer in the presence of light constant in time, which has to be interpreted either locally (Case 1) or globally (Case 2) in relation to the presence of light at constant light intensity $I(x, y, z)$ (Case 1) or I (Case 2).

Upon keeping the notations and the assumptions introduced above in the model of irreversible two-state exchange, we derive at first order

$$k_{12} = 2.3\epsilon_1(\lambda_{\text{exc}})\varphi_{12} I_0(\lambda_{\text{exc}}) \tag{S53}$$

$$k_{21} = 2.3\epsilon_2(\lambda_{\text{exc}})\varphi_{21} I_0(\lambda_{\text{exc}}) + k_{21}^{\Delta} \tag{S54}$$

where $\epsilon_1(\lambda_{\text{exc}})$ and $\epsilon_2(\lambda_{\text{exc}})$ are the scaled molar excitation coefficients for photoisomerization of **1** and **2** at the excitation wavelength, and φ_{12} and φ_{21} are the quantum yields associated to photoisomerization of **1** into **2** and of **2** into **1** respectively. Eqs. (S53,S54) can be alternatively written by introducing the cross sections for the photochemical reaction $\sigma_{12}(\lambda_{\text{exc}})$ and $\sigma_{21}(\lambda_{\text{exc}})$

$$\sigma_{12}(\lambda_{\text{exc}}) = 2.3\epsilon_1(\lambda_{\text{exc}})\varphi_{12} \tag{S55}$$

$$\sigma_{21}(\lambda_{\text{exc}}) = 2.3\epsilon_2(\lambda_{\text{exc}})\varphi_{21} \tag{S56}$$

[21] Note that "suddenly" here refers to a time interval such that Eqs.(S43,S44) can be considered valid.

[22] Note that the expression given in Eq.(S51) is independent on the initial composition of the system.

which yields

$$k_{12} = \sigma_{12}(\lambda_{\rm exc}) I_0(\lambda_{\rm exc}) \quad \text{(S57)}$$

$$k_{21} = \sigma_{21}(\lambda_{\rm exc}) I_0(\lambda_{\rm exc}) + k_{21}^{\Delta}. \quad \text{(S58)}$$

Eqs.(S53,S54) show that the rate constants k_{12} and k_{21} can be evaluated from the knowledge of the photophysical and photochemical parameters, $\epsilon_1(\lambda_{\rm exc})$, $\epsilon_2(\lambda_{\rm exc})$, φ_{12} and φ_{21}, as soon as $I_0(\lambda_{\rm exc})$ is known. Conversely, $I_0(\lambda_{\rm exc})$ can be retrieved with Eq.(S59) retrieved from Eq.(S53,S54,S57,S58) from the knowledge of $\epsilon_1(\lambda_{\rm exc})$, $\epsilon_2(\lambda_{\rm exc})$, φ_{12} or $\sigma_{12}(\lambda_{\rm exc})$, φ_{21} or $\sigma_{21}(\lambda_{\rm exc})$, and the relaxation time of the photochemical reaction τ.

$$I_0(\lambda_{\rm exc}) = \frac{k_{12} + k_{21} - k_{21}^{\Delta}}{2.3\left(\epsilon_1(\lambda_{\rm exc})\varphi_{12} + \epsilon_2(\lambda_{\rm exc})\varphi_{21}\right)} = \frac{\left(1 - k_{21}^{\Delta}\tau\right)}{\left(\sigma_{12}(\lambda_{\rm exc}) + \sigma_{21}(\lambda_{\rm exc})\right)\tau} \quad \text{(S59)}$$

When

$$I(\lambda_{\rm exc}) \geq 10 \times \frac{k_{21}^{\Delta}}{\sigma(\lambda_{\rm exc})}, \quad \text{(S60)}$$

$$k_{21}^{\Delta}\tau \ll 1, \quad \text{(S61)}$$

and Eq.(S59) can be simplified to yield Eq.(S62)

$$I = \frac{1}{\sigma(\lambda_{\rm exc})\tau} \quad \text{(S62)}$$

where we simplified the notations by identifying $I_0(\lambda_{\rm exc})$ to $I(\lambda_{\rm exc})$ and $[\sigma_{12}(\lambda_{\rm exc}) + \sigma_{21}(\lambda_{\rm exc})]$ to $\sigma(\lambda_{\rm exc})$ as done in the Main Text.

When the illumination is focussed, Eq.(S26) is valid with a similar analysis to the one exposed above.

Expression of the absorbance The time evolution of the absorbance $A(\lambda_{\rm exc})$ at the excitation wavelength $\lambda_{\rm exc}$ is expressed in Eq.(S63)

$$A(\lambda_{\rm exc}) = \left[\varepsilon_1(\lambda_{\rm exc})1 + \varepsilon_2(\lambda_{\rm exc})2\right]\ell = A(\lambda_{\rm exc})^{\infty} + \left\{\left[\varepsilon_1(\lambda_{\rm exc}) - \varepsilon_2(\lambda_{\rm exc})\right]\exp\left(-\frac{t}{\tau}\right)\right\}2^{\infty}\ell \quad \text{(S63)}$$

where $\varepsilon_1(\lambda_{\rm exc})$ and $\varepsilon_2(\lambda_{\rm exc})$ designate the molar absorption coefficients of **1** and **2** respectively, $A(\lambda_{\rm exc})^{\infty} = \varepsilon_1(\lambda_{\rm exc})1^{\infty} + \varepsilon_2(\lambda_{\rm exc})2^{\infty}$, and ℓ is the optical pathlength. As shown in Eq.(S63), the relaxation time τ can be simply extracted from a robust monoexponential fit of the temporal evolution of the absorbance signal.

Expression of the fluorescence We consider two cases where fluorescence can be used to report on the time evolution of the illuminated actinometer:

- We first consider the case of bright states **1** and **2**;
- We then consider the case of adding a fluorophore, which reports on the time evolution of the illuminated actinometer by the inner filter effect.

Intrinsic fluorescence reporting When **1** and **2** are intrinsically bright, the temporal dependence of the fluorescence emission $F(t)$ originates from summing the individual contributions of **1** and **2**. Denoting $Q_i = \epsilon_{\rm i}(\lambda_{\rm exc})\varphi_{\rm F,i}$ for the molecular brightness ($\varphi_{\rm F,i}$ designate the quantum yield of fluorescence of the species **i**), one has

$$F(t) \quad = \quad (Q_1 1 + Q_2 2)\, I = F^{\infty} + (Q_1 - Q_2)\, 2^{\infty} I \exp\left(-\frac{t}{\tau}\right) \tag{S64}$$

where $F^{\infty} = Q_1 1^{\infty} + Q_2 2^{\infty}$. Then, as shown in Eq.(S64), the relaxation time τ can be simply extracted from a robust monoexponential fit of the temporal evolution of the fluorescence signal.

Extrinsic fluorescence reporting When **1** and **2** are not fluorescent, one can add a fluorophore to report on the time evolution of the absorbance driven by the the time evolution of the illuminated actinometer. The equations (S31,S32) derived above are still valid and when $A_{\rm tot}(\lambda_{\rm exc}) < 0.15$, we develop the exponential term in Eq.(S31) at the second order around zero and obtain Eq.(S33), which demonstrates that τ can be retrieved from analyzing the time evolution fluorescence of the reporting fluorophore **F**.

5.3 Second protocol: Transfer of information on light intensity from a wavelength to another with a photochemically inert fluorophore

5.3.1 Illumination with monochromatic illumination

We consider a fluorophore illuminated with a light source (incident photon flux $\mathcal{I}_0(\lambda_{\rm exc})$) that perpendicularly illuminates a cuvette filled with its solution. The reaction rate for its conversion to its excited state **E** is proportional to the photon flux absorbed by its ground state **G** at the monochromatic excitation wavelength $\mathcal{I}_G(\lambda_{\rm exc})$ (expressed in E.s^{-1}). Then the time evolution of the concentration G in **G** obeys Eq.(S65)

$$-\frac{dG}{dt} \quad = \quad \frac{\mathcal{I}_G(\lambda_{\rm exc})}{V} \tag{S65}$$

where V designates the illuminated volume of the fluorophore solution.

$\mathcal{I}_G(\lambda_{\rm exc})$ is given by Eq.(S66)

$$\mathcal{I}_G(\lambda_{\rm exc}) \quad = \quad \frac{A_G(\lambda_{\rm exc})}{A_{\rm tot}(\lambda_{\rm exc})}\mathcal{I}(\lambda_{\rm exc}) \tag{S66}$$

where $A_G(\lambda_{\rm exc})$ is the absorbance of **G**, $A_{\rm tot}(\lambda_{\rm exc})$ is the total absorbance, and $\mathcal{I}(\lambda_{\rm exc})$ is the photon flux absorbed by the solution at the excitation wavelength. The latter is given in Eq.(S20).

Upon introducing the length of the optical pathlength ℓ and the known molar absorption coefficient $\varepsilon_G(\lambda_{\rm exc})$ (expressed in m^2.mol^{-1}) of the fluorophore at the excitation wavelength, Eq.(S65) yields

$$-\frac{dG}{dt} \quad = \quad \varepsilon_G(\lambda_{\rm exc})\mathcal{I}_0(\lambda_{\rm exc})\ell\frac{1 - exp\,(-2.3A_{\rm tot}(\lambda_{\rm exc}))}{A_{\rm tot}(\lambda_{\rm exc})V}G. \tag{S67}$$

If the total absorbance $A_{\rm tot}(\lambda_{\rm exc})$ is lower than 0.15, the kinetics of light absorption is first order (at first order) with a rate constant $k_{\rm abs}$ given in Eq.(S68)

$$k_{\rm abs} \quad = \quad 2.3\varepsilon_G(\lambda_{\rm exc})\frac{\mathcal{I}_0(\lambda_{\rm exc})\ell}{V} = 2.3\varepsilon_G(\lambda_{\rm exc})I_0(\lambda_{\rm exc}) \tag{S68}$$

upon introducing the incident light intensity $I_0(\lambda_{\rm exc})$ (expressed in E.m^{-2}.s^{-1}).

We introduce the total concentration of the fluorophore as $F_{\rm tot}$. Far from the saturation of the excited state of the fluorophore, the fluorophore population in its excited state is given by $E = k_{\rm abs}\tau_E F_{\rm tot}$ where τ_E designates the lifetime of the excited state. Then the fluorescence signal $F\left(\lambda_{\rm exc}, \lambda_{\rm em}\right)$ collected from the fluorophore at a wavelength $\lambda_{\rm em}$ upon exciting at $\lambda_{\rm exc}$ is given in Eq.(S69)

$$F\left(\lambda_{\rm exc}, \lambda_{\rm em}\right) = k_F E = k_F k_{\rm abs}\tau_E F_{\rm tot} = 2.3 k_F \tau_E F_{\rm tot}\varepsilon_G(\lambda_{\rm exc}) I_0(\lambda_{\rm exc}) \tag{S69}$$

where k_F designates the rate constant associated to the radiative deexcitation of the excited state by fluorescence emission.

Most of the fluorophores obey the Kasha's rule, which states that photon emission (termed fluorescence in the case of a singlet S state) is expected in appreciable yield only from the lowest excited state, S_1. Since only one state is expected to yield emission, an equivalent statement of the rule is that the wavelength and the quantum yield of fluorescence emission are independent of the excitation wavelength. Then the ratio $F\left(\lambda_{\rm exc}, \lambda_{\rm em}\right)/\varepsilon_G(\lambda_{\rm exc}) I_0(\lambda_{\rm exc})$ is a constant and the fluorescence signals $F\left(\lambda_{\rm exc,1}, \lambda_{\rm em}\right)$ and $F\left(\lambda_{\rm exc,2}, \lambda_{\rm em}\right)$ recorded at a same emission wavelength $\lambda_{\rm em}$ from a same fluorophore solution upon exciting at the wavelengths $\lambda_{\rm exc,1}$ (with a known light intensity $I_0(\lambda_{\rm exc,1})$) and $\lambda_{\rm exc,2}$ (with light intensity $I_0(\lambda_{\rm exc,2})$ to be measured) respectively are linked with Eq.(S70), which is given in Figure 1b of the Main Text upon identifying $I_0(\lambda_{\rm exc})$ to $I(\lambda_{\rm exc})$ in order to simplify the notations

$$I_0(\lambda_{\rm exc,2}) = \frac{\varepsilon_G\left(\lambda_{\rm exc,1}\right) F\left(\lambda_{\rm exc,2}, \lambda_{\rm em}\right)}{\varepsilon_G\left(\lambda_{\rm exc,2}\right) F\left(\lambda_{\rm exc,1}, \lambda_{\rm em}\right)} I_0\left(\lambda_{\rm exc,1}\right). \tag{S70}$$

5.3.2 Illumination with non-monochromatic illumination

We now consider a light source associated to a spectral light intensity $I_0(\lambda)$ (expressed in E.m^{-2}.s^{-1}.nm^{-1}) spread over [$\lambda_{\rm min}$;$\lambda_{\rm max}$] (with the wavelength expressed in nm). Then the rate constant $k_{\rm abs}$ for light absorption by the fluorophore obeys Eq.(S71)

$$k_{\rm abs} = 2.3 \int_{\lambda_{\rm min}}^{\lambda_{\rm max}} \varepsilon_G(\lambda) I_0(\lambda) d(\lambda) \tag{S71}$$

and

$$F\left([\lambda_{\rm min};\lambda_{\rm max}], \lambda_{\rm em}\right) = k_F E = k_F k_{\rm abs}\tau_E F_{\rm tot} = 2.3 k_F \tau_E F_{\rm tot} \int_{\lambda_{\rm min}}^{\lambda_{\rm max}} \varepsilon_G(\lambda) I_0(\lambda) d(\lambda). \tag{S72}$$

The application of the Kasha's rule now yields the ratio $F\left([\lambda_{\rm min};\lambda_{\rm max}], \lambda_{\rm em}\right) / \int_{\lambda_{\rm min}}^{\lambda_{\rm max}} \varepsilon_G(\lambda) I_0(\lambda) d(\lambda)$ to be a constant and the relation Eq.(S72) yields Eq.(S73)

$$\int_{\lambda_{\rm min}}^{\lambda_{\rm max}} \varepsilon_G(\lambda) I_0(\lambda) d(\lambda) = \frac{\varepsilon_G\left(\lambda_{\rm exc,1}\right) F\left([\lambda_{\rm min};\lambda_{\rm max}], \lambda_{\rm em}\right)}{F\left(\lambda_{\rm exc,1}, \lambda_{\rm em}\right)} I_0\left(\lambda_{\rm exc,1}\right) \tag{S73}$$

by assuming that the light source delivering emission at $\lambda_{\rm exc,1}$ is monochromatic (see section 6).

We introduce the normalized emission spectrum $j(\lambda) = I_0(\lambda)/S_{I,0}$, where $S_{I,0}$ designates the integral of $I(\lambda)$ over [$\lambda_{\rm min}$;$\lambda_{\rm max}$] (Eq.(S74))

$$\int_{\lambda_{\rm min}}^{\lambda_{\rm max}} I_0(\lambda) d(\lambda) \quad = \quad S_{I,0} \tag{S74}$$

and the integral of $j(\lambda)$ over the same wavelength range is equal to one (see Eq.(S38)). Then Eq.(S73) yields Eq.(S75)

$$S_{I,0}\int_{\lambda_{\min}}^{\lambda_{\max}}\varepsilon_G(\lambda)j(\lambda)d(\lambda)=\frac{\varepsilon_G\left(\lambda_{\text{exc},1}\right)F\left([\lambda_{\min};\lambda_{\max}],\lambda_{\text{em}}\right)}{F\left(\lambda_{\text{exc},1}\right)}I_0\left(\lambda_{\text{exc},1}\right)\tag{S75}$$

Hence, the computation of the integral of the action spectrum AS_{abs} of the light source given in Eq.(S79)

$$AS_{\text{abs}}=\int_{\lambda_{\min}}^{\lambda_{\max}}\varepsilon_G(\lambda)j(\lambda)d(\lambda)\tag{S76}$$

together with the acquisition of $F\left([\lambda_{\min};\lambda_{\max}],\lambda_{\text{em}}\right)$ and $F\left(\lambda_{\text{exc},1}\right)$ measured with a light source emitting at $\lambda_{\text{exc},1}$ with intensity $I_0\left(\lambda_{\text{exc},1}\right)$ yields the integral

$$S_{I,0}=\frac{\varepsilon_G\left(\lambda_{\text{exc},1}\right)F\left([\lambda_{\min};\lambda_{\max}],\lambda_{\text{em}}\right)}{AS_{\text{abs}}F\left(\lambda_{\text{exc},1}\right)}I_0\left(\lambda_{\text{exc},1}\right)\tag{S77}$$

which can be alternatively written

$$S_{I,0}=\frac{F\left([\lambda_{\min};\lambda_{\max}],\lambda_{\text{em}}\right)}{AS_{\text{abs,norm}}F\left(\lambda_{\text{exc},1}\right)}I_0\left(\lambda_{\text{exc},1}\right)\tag{S78}$$

by introducing the integral of the normalized action spectrum $AS_{\text{abs,norm}}$ of the light source given in Eq.(S79)

$$AS_{\text{abs,norm}}=\int_{\lambda_{\min}}^{\lambda_{\max}}\frac{\varepsilon_G(\lambda)}{\varepsilon_G\left(\lambda_{\text{exc},1}\right)}j(\lambda)d(\lambda)\tag{S79}$$

It becomes then possible to retrieve the scaled spectral light intensity $I_0(\lambda)=S_{I,0}\times j(\lambda)$ sought for given in Eq.(S80)

$$I_0(\lambda)=\frac{\varepsilon_G\left(\lambda_{\text{exc},1}\right)F\left([\lambda_{\min};\lambda_{\max}],\lambda_{\text{em}}\right)}{\left(\int_{\lambda_{\min}}^{\lambda_{\max}}\varepsilon_G(\lambda)j(\lambda)d(\lambda)\right)F\left(\lambda_{\text{exc},1}\right)}I_0\left(\lambda_{\text{exc},1}\right)j(\lambda)=\frac{F\left([\lambda_{\min};\lambda_{\max}],\lambda_{\text{em}}\right)}{AS_{\text{abs,norm}}F\left(\lambda_{\text{exc},1}\right)}I_0\left(\lambda_{\text{exc},1}\right)j(\lambda)\tag{S80}$$

Eq.(S80) bears much similarity to Eq.(S70) except for a convolution of the molar absorption coefficient of the fluorophore with the spectral light intensity.

In practice, one has the dependence of the absorption molar coefficient $\varepsilon_G(\lambda)$ on the wavelength and an unscaled emission spectrum of the light source $S(\lambda)$ (expressed in arbitrary unit) and the goal is to scale it to retrieve the spectral light intensity $I_0(\lambda)$ (expressed in E.m^{-2}.s^{-1}.nm^{-1}) at the sample. The scaling step first involves the normalization of the unscaled emission spectrum $S(\lambda)$ by its integral S (see Eq.(S42)) to yield the normalized emission spectrum $j(\lambda)=S(\lambda)/S$. Then one proceeds as reported above to first compute the integral of the action spectrum AS_{abs} of the light source given in Eq.(S79), and then retrieve the scaled spectral light intensity $I_0(\lambda)=S_{I,0}\times j(\lambda)$ sought for after determining $S_{I,0}$ with Eq.(S77) from the acquisition of $F\left([\lambda_{\min};\lambda_{\max}],\lambda_{\text{em}}\right)$ and $F\left(\lambda_{\text{exc},1}\right)$ measured with a light source emitting at $\lambda_{\text{exc},1}$ with intensity $I_0\left(\lambda_{\text{exc},1}\right)$.

In order to simplify the notations, we identified $I_0(\lambda_{\text{exc}})$ to $I(\lambda_{\text{exc}})$ and $S_{I,0}$ to S_I in the Main Text.

6 Supplementary Note 6: Assumption of monochromatic vs polychromatic light for retrieving cross sections of light absorption

In this account, we used filtered LEDs (for the experiments in cuvettes and in epifluorescence microscopy) and a Xe lamp (for the experiments in cuvettes) as light sources. Although they exhibit a limited bandwidth – less than 2-3 and 10

nm with the filtered Xe lamp and LEDs respectively, we have been concerned with the level of error, which result from considering them as strictly monochromatic for retrieving both the cross sections associated to the photoconversion (for the actinometers) and the fluorescence intensity (for **DDAO**) at a given wavelength λ_{exc}.

We consider a light source associated to a spectral light intensity $I(\lambda)$ (expressed in E.m^{-2}.s^{-1}.nm^{-1}) spread over [λ_{min};λ_{max}] (with the wavelength expressed in nm). It perpendicularly illuminates a cuvette filled with an actinometer or a photochemically inert fluorophore associated to a scaled excitation spectrum leading to its light absorption $\epsilon(\lambda)$ (expressed in m^2.mol^{-1}). Then the rate constant for light absorption by the actinometer or the fluorophore obeys Eq.(S81)

$$k_{\text{abs,poly}} = 2.3 \int_{\lambda_{\text{min}}}^{\lambda_{\text{max}}} \epsilon(\lambda) I(\lambda) d(\lambda). \tag{S81}$$

$k_{\text{abs,poly}}$ determines the value of the fluorescence intensity of the fluorophore but it also determines the rate constant of the photoconversion of the actinometer k_{12} when its associated quantum yield φ_{12} does not depend on the excitation wavelength (see Eqs.(S23,S24)).

In the polychromatic approximation, we consider $I(\lambda)$ to be a Gaussian distribution (bandwidth 2σ) with its integral $I_{\lambda_{\text{exc}}}$ peaking at λ_{exc} and extending over the wavelength range [$\lambda_{\text{exc}} - 3\sigma$; $\lambda_{\text{exc}} + 3\sigma$]. Then Eq.(S81) yields Eq.(S82)

$$k_{\text{abs,poly}} = 2.3 \int_{\lambda_{\text{exc}}-3\sigma}^{\lambda_{\text{exc}}+3\sigma} \epsilon(\lambda) \frac{I_{\lambda_{\text{exc}}}}{\sigma\sqrt{2\pi}} e^{\frac{-(\lambda-\lambda_{\text{exc}})^2}{2\sigma^2}} d\lambda. \tag{S82}$$

In the monochromatic approximation, $I(\lambda) = I_{\lambda_{\text{exc}}} \delta(\lambda - \lambda_{\text{exc}})$ and Eq.(S81) reduces to Eq.(S83)

$$k_{\text{abs,mono}} = 2.3 \times \epsilon(\lambda_{\text{exc}}) \times I_{\lambda_{\text{exc}}}. \tag{S83}$$

To assess the relevance of the monochromatic approximation for retrieving both the cross sections associated to the photoconversion (for the actinometers) and the fluorescence intensity (for **DDAO**) at a given wavelength λ_{exc}, we generated a simulated arbitrary spectrum $\epsilon(\lambda)$ (Figure S23a) and computed the deviation ($\frac{|k_{\text{abs,poly}} - k_{\text{abs,mono}}|}{max(k_{\text{abs,poly}}, k_{\text{abs,mono}})}$) for various values of λ_{exc} (position in the spectrum) and σ (half bandwidth of the light source) (Figure S23b). For $\sigma = 20$ nm (larger than the half bandwidth of our LEDs) and whatever λ_{exc}, the error remains below 20%, which is the error estimated for our evaluations of light intensity. In fact, the bandwidths of all the light-sources we used for calibration were below $\sigma = 10$ nm so that the error is closer to 5%, which validates our exploitation of the monochromatic approximation for retrieving both the cross sections associated to the photoconversion (for the actinometers) and the fluorescence intensity (for **DDAO**) at a given wavelength λ_{exc}.

7 Supplementary Note 7: Characterization of the fluorescent systems for measuring light intensity

This section details the Materials and Methods, and the thorough characterization of the fluorescent systems, which we report to measure light intensity. It also details the experiments, which have been performed for their validation.

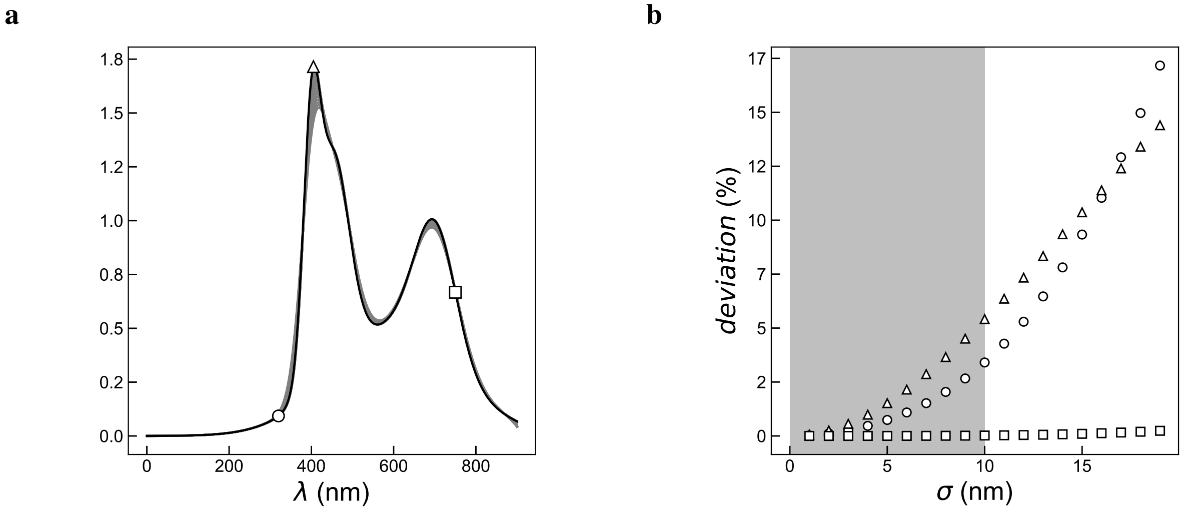

Figure S23: *Evaluation of the assumption of monochromatic vs polychromatic light for retrieving cross sections of light absorption.* **a** Arbitrary excitation spectrum $\epsilon(\lambda)$ (solid black line). This spectrum has been blurred by its convolution with a gaussian kernel with increasing standard deviation (from 1 to 20 nm) representing the emission spectra of light sources with various bandwidths (grey lines) in order to give an insight of how the bandwidth of the light source affects the spectrum values; **b** Evolution of the deviation between $k_{\text{abs,poly}}$ and $k_{\text{abs,mono}}$ computed for various values of λ_{exc} (circles: 320 nm, triangles: 405 nm, squares: 750 nm) and σ for the spectrum $\epsilon(\lambda)$ given in **a**. The grey area represents the range of bandwidths of the various light sources used in this manuscript.

7.1 Materials

The materials were produced as reported in the Methods in the Main Text. For the syntheses, the reagents were obtained from Sigma-Aldrich or TCI and used without purification. Solvents were obtained from VWR. Flash chromatography silica (high purity, 6 nm pore size, 40-63 μm particle size) was obtained from Sigma-Aldrich and was used to perform purifications in manual column chromatography. NMR measurements were recorded using a Bruker Ultrashield 300 MHz NMR. ^{1}H-NMR chemical shifts are reported using the residual protons of the deuterated solvents as references. ^{13}C chemical shifts are reported using the signal from the solvents as references. The NMR spectra were processed using the Mnova Mestrelab software. Mass spectra were recorded using a Bruker UHR-Q-TOF MaXis system with ESI ionization method at the High-resolution Mass Spectroscopy Platform at Orleans University.

7.2 UV/Vis absorption and fluorescence spectrometers

7.2.1 Instruments

UV/Vis absorption spectra were recorded on a UV/Vis spectrophotometer (Cary 300 UV-Vis, Agilent Technologies, Santa Clara, CA – software: Cary Win UV, version 4.10(464)) at 20 °C equipped with a Peltier 1×1 thermostatic cell holder (Agilent Technologies). Samples were contained either in 1 cm × 1 cm (3 mL; the cuvette content was stirred) or in 0.3 cm × 0.3 cm (45 μL; the cuvette content was not stirred) quartz cuvettes (Hellma Optics, Jena, Germany). Fluorescence measurements were acquired on a LPS 220 spectrofluorometer (PTI, Monmouth Junction, NJ), equipped with a TLC50 cuvette holder (Quantum Northwest, Liberty Lake, WA) thermoregulated at 25 °C.

7.2.2 Light sources

The constant illumination applied to generate the time evolution of the fluorescence signal from the chemical actinometers has been obtained by using:

- LEDs for the photoconversion experiments performed at various light intensities. The lights from high power LEDs (NCSU033B, Nichia Corp, JP; LHUV-405, LXZ1-PB01, Lumileds, NL, LXML-PWN1-0080, Lumileds, NL) driven by a 4-channel current driver (DC4104, Thorlabs, NJ) were collimated by high-NA condenser (ACL25416UA, $f = 16$ mm, Thorlabs, NJ) and filtered (BP ZET 365/20x, Chroma Technology, BP ET405/20x; Chroma Technology, VT, BP FF01-479/40-25, Semrock, NY and SP 694/SP, AHF, FR combined with a 665 dichroïc filter FF665-Di02-25x36, Semrock, NY). The quasi-parallel beams were combined with dichroic mirrors (ZT375rdc, T425LPXR, Chroma Technology, VT) and sent on the 3×3 mm^2 aperture of the sample cuvette;
- the Xenon lamp of the LPS 220 spectrofluorometer (PTI, Monmouth Junction, NJ) for the photoconversion experiments performed at various excitation wavelengths.

The emission spectra of the LEDs have been recorded by sending the light emitted from the LEDs into the emission pathway of the fluorometer with reduced slits opening with the software PTI Felix (4.1.0.3096).

7.2.3 Measurement of the light intensity with a powermeter

A powermeter (PM100A, Thorlabs, NJ; *S130C* probe, Thorlabs, NJ) has been used to report on the light intensity applied on the cuvettes. In a first experiment, we analyzed the time recovery of the fluorescence signal from 488 nm-photoswitched **Dronpa-2** upon illumination at 405 nm[7,11] in order to calibrate at 405 nm the geometrical factor linking the indication provided by the powermeter with the light intensity applied on the cuvette. Subsequently keeping constant the position of the powermeter probe in the following experiments, we neglected the dispersion of the refractive index over the investigated wavelength range and retained the value of this geometrical factor to convert the indication of the powermeter (in W) into the light intensity (in E.s^{-1}.m^{-2}; see Section 4) at the excitation wavelength.[23]

7.3 Optical setups

7.3.1 Epifluorescence microscope

Description Several photoconversion experiments (on photosynthetic organisms, **Dronpa-2**, **DASA**, **DDAO**) have been performed on a home-built inverted epifluorescence microscope (Figure S24). To illuminate the samples, the lights from high power LEDs (LXZ1-PB01, LHUV-405, LXZ1-PX01, LXM3-PD01; Lumileds, NL) are collimated by high-NA condensers (ACL25416U-A, $f = 16$ mm, Thorlabs, NJ) and filtered by band pass filters (ET405/20x, ET550/15x; Chroma

[23] In our setup, the LEDs deliver homogeneous illumination on the 0.3 cm $\times$ 0.3 cm cuvette and the light intensity retrieved from the measurement with the powermeter corresponds to the applied illumination in this case. In contrast, the measurement with the powermeter only provides an equivalent light intensity assuming homogeneous illumination of the whole cuvette content when illumination is performed with the Xe lamp – delivering heterogeneous illumination – or when using a partially illuminated a 1 cm $\times$ 1 cm cuvette in which only part of the stirred content is illuminated.

Technology, VT and FF01-479/40-25, FF01-650/13, Semrock, US) to avoid spectral overlaps. The quasi-parallel beams are combined with dichroic mirrors (T425LPXR, T505LPXR Chroma Technology, VT and FF01 560 Di01, Semrock, NY) and injected in a 400 μm-core optical fiber using a 20× objective (Nikon, NA = 0.75) to further homogenize the light beams. Light at the output of the fiber is collimated with a 10× objective (Olympus, NA = 0.5) and focused with $f = 150$ mm lens (AC254-150-A Thorlabs, NJ) at the back focal plane of a 10× imaging objective (Zeiss, NA = 0.5) after being reflected by a dichroic mirror (Di-FF506, Semrock, US). The LEDs are powered by an LED driver (DC4104, Thorlabs, NJ) controlled by an Arduino Uno card or a DAQ card (SCB68A with PCI 6374, National Instruments, US) depending on the applications. The DAQ card also collects the output signals from detectors at 3 MHz maximum. The fluorescence signal can either be collected by a photodetector (MPPC C13366 Hamamatsu, JP) or a camera (UEye 3060CT-M - IDS, DE). Another photodetector (MPPC C13366 Hamamatsu, JP) is used to collect the light excitation before it reaches the sample. The photodetector was used in subsections 2.1.3, 2.4.3, 7.4.4 dealing with experiments on **PA**, **DASA**, and **DDAO** (Fluorescence images at wavelength 690/8 nm – FF01 690/8 Semrock), whereas the camera was used in subsections 2.5, 7.4.3, 7.6 for the **Dronpa-2** experiments (Fluorescence images at 525/30 nm – FF525-30, Semrock, US). The videos were acquired at 3 or 5 Hz.

In order to evidence the effect of diffusion on the determination of the light intensity in the sample (see Figure 2g–i of the Main Text), we patterned the light in the focal plane by inserting a silver film for camera with a black and white imprinted picture on the conjugated plan of the objective.

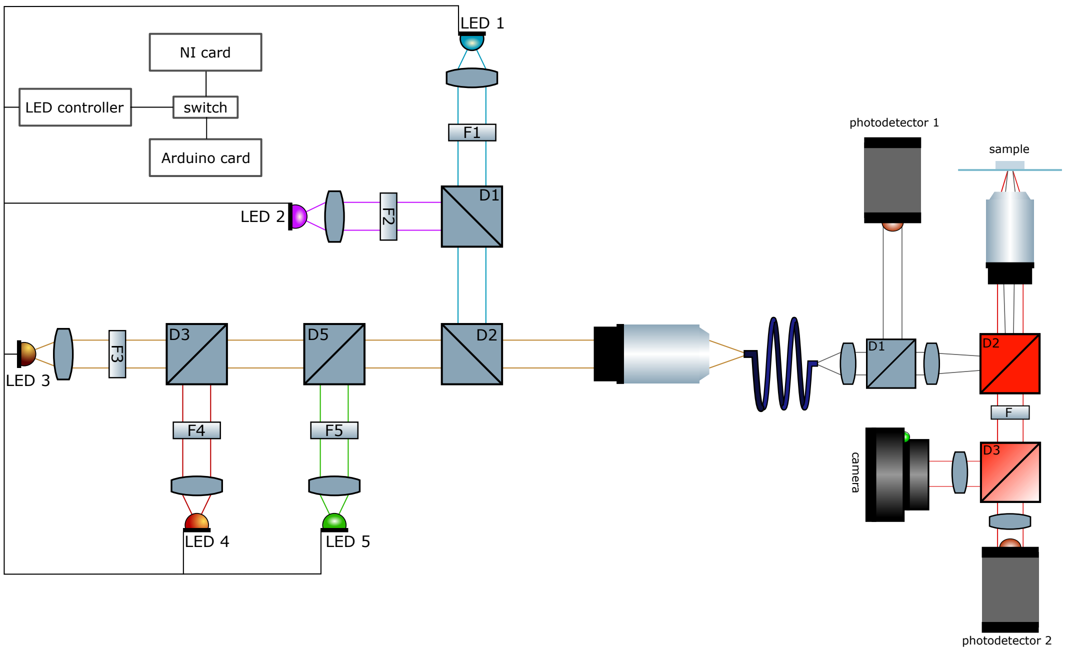

Figure S24: Schematic representation of the epifluorescence microscope.

Measurement of the light intensity at the focal plane with a powermeter The light intensity at the focal plane of this microscope has been estimated with a powermeter (PM100A, Thorlabs, NJ). Its probe (*S170C* Thorlabs, NJ) was

placed at the position of the sample and the excitation wavelength was selected. The collected output is given in Watt. To estimate the surfacic power (W/m^2), we further recorded a scaled image of the illumination spot at the sample position to measure the spot surface (0.25 mm^2 in our configuration where the illumination was narrowed by a diaphragm; see Figure 2a of the Main Text).

7.3.2 Fluorescence macroimager

Images of a CAD model representation of the physical setup, created in Rhinoceros 3D (Robert McNeel & Associates, Seattle, WA, US), are shown in Figure S25. The setup consists of separated imaging and illumination paths. The two views provided of the model show how the angle of the optical axes of both, which are shown in red, differ. Looking at the macroscope from the front, the illumination axis is offset from vertical by 28.6^o, and from the side, 11.2^o. The imaging path consists of a macroscope objective (1X/WF, Nikon, Tokyo, Japan), to collimate the light originating from the sample plane; an emission filter (550 nm CWL, 100 nm FWHM; 33-331, Edmund Optics Inc., Barrington, NJ, US), to pass as much of only the fluoresced light as possible; and a camera objective (AF Nikkor 50 mm f/1.8D, Nikon, Tokyo, Japan), to focus the collimated light onto the image sensor of a greyscale global-shutter camera (UI-3060CP-M-GL, IDS Imaging Development Systems GmbH, Obersulm, Germany). The illumination path consists of a condenser lens (ACL25416U, Thorlabs Inc., Newton, NJ, US) to collect the light from a blue LED (L1RX-BLU1000000000, Lumileds, San Jose, CA, USA) and collimate it for passing through an excitation filter (ET470/40×, Chroma Technology Corp., Bellows Falls, VT, US). Following the filter, the collimated light is injected into the entrance of a lightpipe (65-840, Edmund Optics Inc., Barrington, NJ, US) using a plano convex lens (LA1422-A, Thorlabs Inc., Newton, NJ, US). The lightpipe is used to homogenize the input light, providing homogeneous light at the pipe's exit. The exit end of the lightpipe is conjugated to the sample plane through the use of a matched achromatic doublet pair (MAP1040100-A, Thorlabs Inc., Newton, NJ, US). Images, or a description, of the optomechanical components used to hold the optical components in place are not described here. The distance between the components in the imaging path is such that the macroscope objective forms collimated light at its output, and the camera objective takes the collimated light and focuses it onto the sensor. The distance between the two objectives was approximately 65 mm, with the emission filter being placed about 20 mm above the macroscope objective. The distances between the components in the illumination path are introduced in the following section on the setup of the simulation.

In relation to the electronics, the LED was connected to an LED driver (DC4104, Thorlabs Inc., Newton, NJ, US) to power it, and the camera to a custom-built PC running Windows 10 (Microsoft Corp., Redmond, WA, US) to accept images from it. The cameras frames were triggered using an Elegoo Uno R3 board (Elegoo, Shenzhen, China).

The experiment was carried out using a 4 μM **Dronpa-2** solution sandwiched between two round 40 mm microscope cover slips (Thermo Fisher Scientific, Waltham, MA, US), using a 17 × 28 mm Gene Frame (Thermo Fisher Scientific, Waltham, MA, US) to maintain the separation between the slides at approximately 0.25 mm and hold the solution in place. The sandwich was placed flat, relative to the imaging optical axis, on the sample plane, and at the

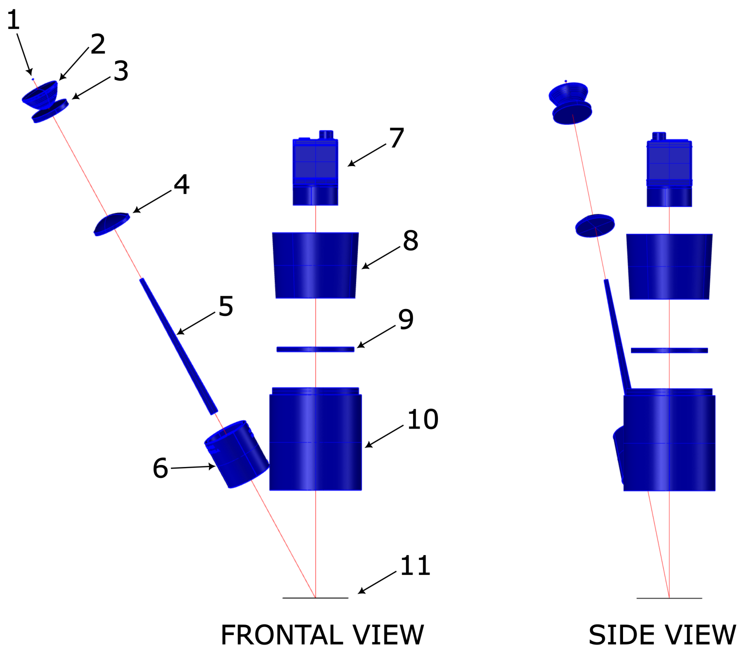

Figure S25: *Fluorescence macroimager*. CAD rendered diagrams of the optical elements composing the macroscope; LEFT – Frontal view, RIGHT –Side view. 1 – LED, 2 – Condenser, 3 – Excitation Filter, 4 – Condenser, 5 – Lightpipe, 6 – Matched Achromatic Doublets, 7 – Camera, 8 – Camera Objective, 9 – Emission Filter, 10 – Macroscope Objective, 11 – Sample.

focal distance of the macroscope objective. The experiment involved acquiring images, at a frequency of 6 Hz, for 30 seconds, while, at a time point shortly after the camera was started, the LED was driven with a current of 1000 mA. The video and data corresponding to the final intensity image output is provided (Macroscope/MACRO_video.tiff and Macroscope/MACRO_ExperimentalData.npy, respectively).

7.3.3 Confocal microscopy

Several photoconversion experiments on **Dronpa-2** have been performed on confocal microscopes. The confocal micrographs were acquired on a Zeiss LSM 710 Laser Scanning Microscope equipped with continuous laser lines at 405 and 488 nm and a Plan NeoFluar 20×/0.5 objective, or on a Leica TCS SP8 confocal microscope equipped with a HC PL APO CS2 40x/1.10 water immersion objective, a continuous laser line delivering light at 405 nm and a white light laser (WLL) source delivering 200-300 ps light pulses at 488 nm every 13.6 ns. ZEN software from Zeiss or LAS X software from Leica were used to collect the data. The images were analyzed with Fiji and Python.

7.3.4 Fluorometer for acquisition of the fast OJIP transient fluorescence response to PA illumination

Besides the corresponding measurements which have been performed with the epifluorescence microscope equipped with the MPPC photodetector, we used the FL 6000 fluorometer from Photon Systems Instruments (PSI; Drásov, Czech Republic) to record the fast OJIP transient fluorescence response to illumination of microalgae.

The LED mounted on this PSI instrument is the LXML-PH01 LED, which belongs to the LUXEON Rebel Color Line

from LUMILEDS. Its unscaled emission spectrum $S(\lambda)$ was measured with a portable spectrophotometer (SpectraPen Mini - PSI, CZ).

7.3.5 Fluorescence Correlation Spectroscopy

The one-photon excitation FCS setup consisted of a laser source, a home built microscope and two avalanche photodiodes (APD). The laser source was a 488 nm laser (LBX-488, Oxxius, Lannion, FR). The laser beam was expanded 3 times at the entrance of the microscope using pairs of converging lenses (AC254-030-A and AC254-075-A, Thorlabs, NJ) to reach a diameter of 8 mm and sent with a dichroic mirror (FF506-Di03, Semrock, NJ) at the back aperture of a water-immersion objective (60×/1.2, UPlanApo, Olympus, JP). The fluorescence was collected through the same objective and filtered with a bandpass filter (ET525/30m, Chroma VT; FF01-650/13 Semrock, NY) before being focused at the entrance of a 50 µm core optical fiber (M14L02, Thorlabs, NJ) with a $f = 200$ mm lens (AC254-200-A, Thorlabs, NJ). Light at the exit the fiber was re-collimated with a 50 mm lens and separated by a cube beam splitter with a 1:1 ratio and then focused on the 200 μm^2 working surfaces of the two APDs (SPCM-AQR-14, Excelitas Technologies, MA). The signal outputs of the APD modules (TTL pulses) were acquired by a digital autocorrelator module (ALV-6000, ALV-Gmbh, DE) which computed online the cross-correlation function of the fluorescence fluctuations. Ten runs of 60 s were averaged to yield the final data including the associated standard deviations at the different lag times.

7.4 Intrinsically fluorescent actinometers

7.4.1 Cin

This actinometer has been thoroughly investigated by Jullien et al.[3,12] It has been shown to irreversibly convert into 6,8-dibromo-7-hydroxycoumarin in water[12] and in water–acetonitrile mixtures[3] under illumination in the UV A wavelength range.

Preparation of the Cin solutions The synthesis of the *(E)*-3-(3,5-dibromo-2,4-dihydroxyphenyl) acrylic acid ethyl ester (**Cin**) actinometer is reported in the Methods in the Main Text. The **Cin** solution was produced in two steps. A first 10 mM stock solution was made by dissolving 3.8 mg **Cin** powder in 1 mL of spectrograde DMSO. This stock solution was subsequently diluted in 20 mM Tris pH 7 100 mM NaCl buffer to get the final solutions used for the fluorometric investigations at 5.6 or 16.8 μM concentration. In these solutions, the volumetric DMSO/Buffer ratio is 0.05 and 0.16 % respectively. These solutions can be kept under the protection of ambient light (container covered with an Aluminium foil) at 20 ^{o}C for up to a day.

Absorption spectrum of Cin and emission spectrum of its coumarin photoproduct **Cin** exhibits a significant light absorption between 300 and 430 nm solution in 20 mM Tris pH 7 100 mM NaCl buffer (see Figure S26). Its photoproduct, 6,8-dibromo-7-hydroxycoumarin, emits fluorescence between 400 and 600 nm in the same solvent (see Figure S26).

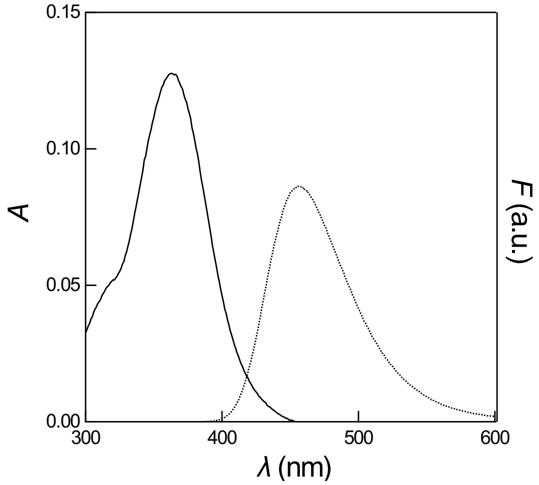

Figure S26: Absorption spectrum of 5.6 μM **Cin** solution (solid line) and fluorescence emission spectrum of its photoproduct, 6,8-dibromo-7-hydroxycoumarin (dotted line; $\lambda_{\mathrm{exc}} = 368$ nm) in 20 mM Tris pH 7 100 mM NaCl buffer recorded in a 1×1 cm^2 quartz cuvette. $T = 293$ K.

Determination of the quantum yield of Cin photoconversion in pH 7 Tris buffer at 405 nm The quantum yield of the **Cin** photoconversion is 0.05 at 350 nm in acetonitrile/20 mM Tris pH 7 100 mM NaCl buffer 1/1 (v/v).[3] In this manuscript, we evaluated its value at 405 nm in pure 20 mM Tris pH 7 100 mM NaCl buffer. We also examined any possible upper range of light intensity beyond which the photochemical step is not anymore rate-limiting in the **Cin** light-driven conversion in pH 7 Tris buffer.

Figure S27a displays the time dependence of the fluorescence emission of 16.8 μM **Cin** in 20 mM Tris pH 7 100 mM NaCl buffer at 456 nm upon irradiation at various light intensities at 405±20 nm, which have been measured by using the **Nit** actinometer (see below). Then we applied the monoexponential fitting function given in Eq.(S1) to the temporal rises of the normalized fluorescence in order to retrieve the characteristic times of the photoconversion τ. As displayed in Figure S27b, the inverse of the characteristic times of the photoconversion linearly depends on the light intensity. This behavior demonstrates that the rate limiting step of **Cin** photoconversion in 20 mM Tris pH 7 100 mM NaCl buffer is photochemical at least up to 6 10^{-4} E.m^{-2}.s^{-1} light intensity. However, despite the observed linearity of the inverse of the characteristic time of the **Cin** photoconversion on the light intensity, one notices that the photoproduced 6,8-dibromo-7-hydroxycoumarin experiences significant photobleaching (see Figure S27a), which may reduce the amplitude of the observable fluorescence rise. Hence it is suggested to restrict the use of the fluorescent actinometer to light measurements below 5 10^{-5} E.m^{-2}.s^{-1} light intensity at 405 nm.

Eq.(S24) was used to retrieve $\sigma = 184 \pm 2$ m^2.mol^{-1} from the slope of the dependence of the inverse of the characteristic time of the photoconversion on the light intensity. Equipped with the latter value of the cross section for the photoconversion and the value of the molar absorption coefficient of **Cin** at 405 nm (0.7 10^4 M^{-1}.cm^{-1} = 0.7 10^3 m^2.mol^{-1}), we used Eq.(S23) to extract $\varphi_F = 0.13 \pm 0.02$ as the value of the quantum yield of the **Cin** photoconversion at 405 nm in 20 mM Tris pH 7 100 mM NaCl buffer.

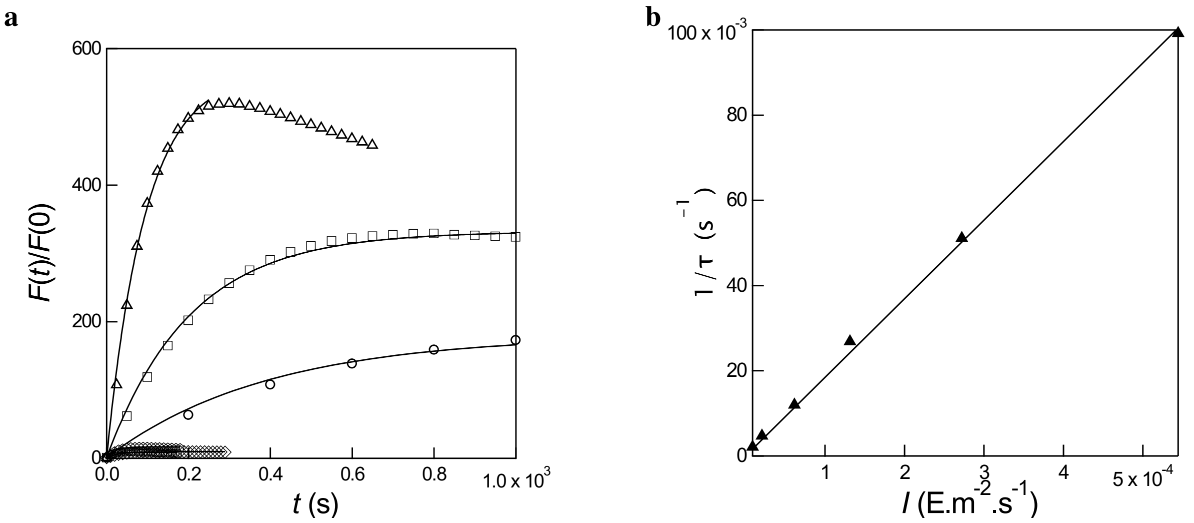

Figure S27: *Determination of the cross section for the photoconversion of (E)-3-(3,5-dibromo-2,4-dihydroxyphenyl) acrylic acid ethyl ester (**Cin**) in pH 7 Tris buffer at 405 nm.* **a**: Time evolution of the normalized fluorescence emission at 456 nm from a 16.8 μM **Cin** in 20 mM Tris pH 7 100 mM NaCl buffer contained in a 3 mm optical path quartz cuvette upon irradiation at various constant light intensity at 405 nm (in 10^{-5} E.m^{-2}.s^{-1}): 0.88 (circles), 2.0 (squares), 6.1 (triangles), 13 (diamonds), 27 (disks), and 54 (hexagons). Markers: Experimental data; Solid lines: Monoexponential fit with Eq.(S1); **b**: Dependence of the inverse of the characteristic time τ retrieved in **a** from the monoexponential fit on the light intensity. Markers: Experimental data; Solid line: Linear fit. The extracted slope is 184 ± 2 m^2.mol^{-1}. $T = 293$ K.

Dependence of the quantum yield of the Cin photoconversion on the excitation wavelength Then we studied the dependence of the quantum yield of the **Cin** photoconversion on the excitation wavelength. Figure S28 displays the time dependence of the fluorescence emission of a 5.6 μM **Cin** in 20 mM Tris pH 7 100 mM NaCl buffer upon irradiation at constant light intensity at 350, 365, 380, 405, and 420 nm, which have been measured from recording the light power transmitted through the 1 cm light path quartz cuvette. Then we applied the monoexponential fitting function given in Eq.(S1) to the temporal rises of the normalized fluorescence at 456 nm in order to retrieve the rate constants of the photoconversion upon exciting at various wavelengths (Figure S28). Equipped with the rate constants of the photoconversion and the light intensities (retrieved from the light powers) measured at all the excitation wavelengths, and the dependence of the molar absorption coefficient of **Cin** on the wavelength, we used Eq.(S23) to extract the dependence of the quantum yield of the **Cin** photoconversion on the excitation wavelength upon fixing the value of the quantum yield of the **Cin** photoconversion to 0.13 at 405 nm. The results are displayed in Table S4. The quantum yield of the **Cin** photoconversion is essentially constant and equal to 0.22 ± 0.02 from 350 to 380 nm but seems to drop on the red edge of its absorption band.

Estimate of the achievable measurement uncertainty on light intensity I Considering the uncertainty in the parameters (σ and τ), we estimate that the achievable measurement uncertainty on light intensity I is 20% by using **Cin**.

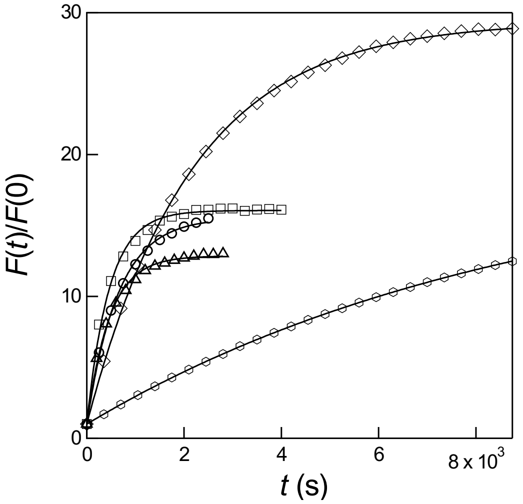

Figure S28: *Dependence of the quantum yield of the* ***Cin*** *photoconversion on the excitation wavelength.* Time dependence of the normalized fluorescence emission at 456 nm of a 5.6 μM **Cin** in 20 mM Tris pH 7 100 mM NaCl buffer contained in a 1×1 cm^2 quartz cuvette upon illumination at constant light intensity at 350 (circles), 365 (squares), 380 nm (triangles), 405 (diamonds), and 420 (hexagons) nm. Markers: Experimental data; solid lines: Monoexponential fit with Eq.(S86). $T = 293$ K.

Table S4: Photoconversion parameters of *(E)*-3-(3,5-dibromo-2,4-dihydroxyphenyl) acrylic acid ethyl ester (**Cin**) in 20 mM Tris pH 7 100 mM NaCl buffer. The quantum yield of photoconversion φ_F has been extracted from analyzing the rise of the fluorescence emission at 456 nm of a 5.6 μM **Cin** in 20 mM Tris pH 7 100 mM NaCl buffer under monochromatic illumination at 350, 365, 380, 405, and 420 nm as representatives of 350 nm – 420 nm range. The molar absorption coefficients $\varepsilon(\lambda_{\rm exc})$ were extracted from the **Cin** absorption spectrum by using the Beer-Lambert law. The cross section $\sigma(\lambda_{\rm exc})$ was evaluated with Eq.(S23). $I_{\rm sup}(\lambda_{\rm exc})$ indicates the upper light intensity tested and relevant for a reliable measurement.

$\lambda_{\rm exc}$ (nm)	$10^3 \times \varepsilon(\lambda_{\rm exc})$ ($\pm$10%; M^{-1}cm^{-1})	$\sigma(\lambda_{\rm exc})$ ($\pm$10%; m^{2}mol^{-1})	φ_F ($\pm$0.02)	$I_{\rm sup}(\lambda_{\rm exc})$ (E.m^{-2}.$^{-1}$ (W.m^2))
350	20	940	0.20	1.6 10^{-5}(5.5)^a
365	23	1200	0.23	1.4 10^{-5}(4.6)^a
380	19	1000	0.24	1.7 10^{-5}(5.3)^a
405	6.4	184	0.13	5.0 10^{-5}(15)^a
420	2.5	49	0.09	1.3 10^{-4}(37)^a

^{a} Extracted from the value of $I_{\rm sup}(\lambda_{\rm exc})$ by using $I_{\rm sup}(\lambda_{\rm exc}) = I_{\rm sup}(\lambda_{\rm exc,ref}) \times \frac{\varepsilon(\lambda_{\rm exc,ref})}{\varepsilon(\lambda_{\rm exc})}$ where $\lambda_{\rm exc,ref}$ designates the wavelength at which the extraction of the cross section of photoconversion has been performed ($\lambda_{\rm exc,ref} = 405$ nm for **Cin**).

7.4.2 Dronpa-2

The reversibly photoswitchable fluorescent proteins (RSFPs)[13] are favorable endogenously fluorescent actinometers, when access to biological techniques is available. Indeed, they are genetically-encoded and can be easily introduced in various live and fixed biological samples (e.g. bacteria, eucaryotic cells from animals and plants, leaves and roots of live plants) or produced as solutes in a buffer solution. Moreover, their cross sections for photoisomerization span a wide range (over two orders of magnitude[7]) in the 450-500 nm range, which opens a road to measure light intensity over a wide range of values. In this manuscript, we illustrate this series of fluorescent actinometers with **Dronpa-2** (or

M159T).[14] This variant of the reversibly photoswitchable green fluorescent protein Dronpa[15] benefits from an appropriate characteristic time close to the minute associated to its thermal return after photoconversion, which makes it appropriate for repeated use without generating a too demanding lower limit of the reliably measurable light intensity. However, the proposed methodology will be relevant as well for the other RSFPs.

In aqueous solution at neutral pH, **Dronpa-2** exhibits a broad absorption band spanning the 420-520 nm range. Under blue light around 480 nm, **Dronpa-2** photoswitches from a bright to a dark state (negative photochromism). In contrast, it photoswitches from a dark to a bright state under violet light (around 405 nm). The quantitative analysis of the kinetics of **Dronpa-2** photoswitching has been thoroughly investigated by Le Saux, Jullien, et al.[7,11] The time evolution of its fluorescence emission upon constant illumination at 480 nm and 405 nm obeys a simple two-state kinetic model at time scales longer than a few ms, which enables to measure light intensity up to 10 $E.m^{-2}.s^{-1}$ (3×10^{6} $W.m^{-2}$).[7]

Preparation of the Dronpa-2 samples Three **Dronpa-2**-based actinometers are proposed for measuring light intensity in the 440-500 nm wavelength range: a **Dronpa-2** solution, **Dronpa-2**-labeled *Escherichia coli* cells, and fixed U-2 OS eucaryotic cells **Dronpa-2**-labeled at their nucleus.

The production of those actinometers is reported in the Methods in the Main Text.

The concentration of the solutions of **Dronpa-2** purified protein, i.e. **Dronpa-2** solution, and **Dronpa-2** labeled bacteria were fixed as follows. The absorption spectrum of the original samples were recorded over the 200 nm – 800 nm wavelength range. The original solutions were subsequently diluted in DPBS 1× pH 7.4 buffer (2.7 mM KCl, 138 mM NaCl, 1.5 mM KH_2PO_4, 8.1 mM Na_2HPO_4) such that their absorbance at 488 nm was in the 0.15 range.

The **Dronpa-2** solution and **Dronpa-2**-labeled *Escherichia coli* cells can be kept under the protection of ambient light (container covered with an Aluminium foil) at - 20 ^{o}C for up to two weeks. The fixed U-2 OS eucaryotic cells **Dronpa-2**-labeled at their nucleus remain functional for several weeks when kept at +4°C.

Absorption and emission spectra of Dronpa-2 At neutral pH, the **Dronpa-2** solution exhibits a significant light absorption between 440 nm and 500 nm and emits fluorescence peaking at 512 nm (see Figure S29).

Determination of the cross sections of photoconversion of Dronpa-2 in bacteria at 480 and 405 nm We already evidenced that the **Dronpa-2** photoconversion can be reliably modeled as a two-state exchange up to a threshold of light intensity in the 10 $E.m^{-2}.s^{-1}$ with values of the cross sections of photoconversion of **Dronpa-2** in aqueous solution at pH 7.4, which are respectively equal to 198 and 415 $m^2.mol^{-1}$ at 480 and 405 nm.[7] We also analyzed the **Dronpa-2** photoconversion in fixed eucaryotic cells **Dronpa-2**-labeled at the nucleus and did not notice any significant departure of the photoconversion kinetics with respect to the solution (see Supplementary Table 6 in the Supporting Information of the reference[11]). In this subsection, we measured the values of the corresponding cross sections of photoconversion of **Dronpa-2** in **Dronpa-2** labeled bacteria.

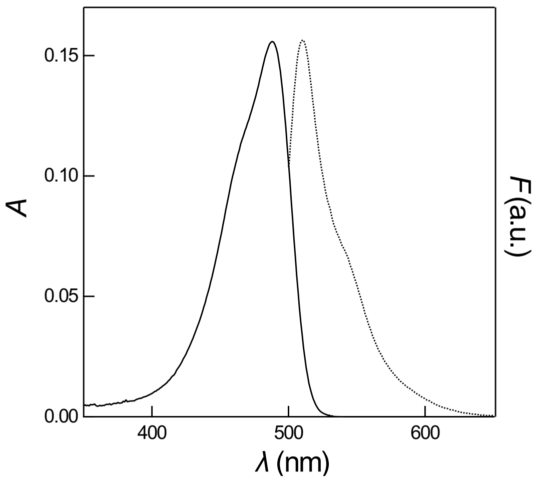

Figure S29: Absorption (solid line) and emission (dotted line; $\lambda_{\text{exc}} = 480$ nm) spectra of 2.1 μM **Dronpa-2** solution in DPBS $1\times$ pH 7.4 buffer recorded in a 1×1 cm^2 quartz cuvette. $T = 293$ K.

Figures S30a,c display the time dependence of the fluorescence emission of the suspension of **Dronpa-2** labeled bacteria in DPBS $1\times$ pH 7.4 buffer contained in a 3 mm light path quartz cuvette upon irradiation at various combinations of light intensities, which have been calibrated from analyzing the time evolution of the fluorescence signal from the **Dronpa-2** pH 7.4 aqueous solution upon illumination at 480 or 405 nm.[7,11] Figure S30a displays the time decay of the fluorescence emission of the suspension of **Dronpa-2** labeled bacteria upon illumination at several light intensities at 480 nm under constant light intensity at 405 nm whereas Figure S30c displays the time rise of the fluorescence emission of the suspension of 480 nm-phoswitched **Dronpa-2** labeled bacteria upon illumination at several light intensities at 405 nm under constant light intensity at 480 nm. Then we applied the monoexponential fitting function given in Eq.(S1) to the temporal evolutions of the normalized fluorescence at 515 nm in order to retrieve the characteristic times of the photoconversion τ under the various conditions of illumination. As displayed in Figures S30b,d the inverses of the characteristic times of the photoconversion linearly depend on the light intensity. This behavior demonstrates that the rate limiting step of the **Dronpa-2** photoconversion in **Dronpa-2** labeled bacteria is photochemical at least up to 2 10^{-3} E.m^{-2}.s^{-1} light intensity. Eq.(S24) was used to retrieve $\sigma(480) = 251 \pm 7$ m^2.mol^{-1} and $\sigma(405) = 370 \pm 2$ m^2.mol^{-1} from the observed slopes.

Comparison of the cross sections of photoconversion of Dronpa-2 at 480 nm in solution and in a polyacrylamide gel In order to account for the impact of molecular diffusion on the determination of the light intensity in the focal plane of the epifluorescence microscope, we compared the kinetics of the photoconversion of **Dronpa-2** on a selected homogeneous region of interest (100×100 pixels; see Figure S31a) in solution and in a polyacrylamide gel (prepared by adding 20 μL of **Dronpa-2** stock solution to 20 μL of 40% acrylamide/bis-acrylamide (37.5:1 ratio, Merck), 1 μL of 10% N,N,N',N'-Tetramethylethylenediamine (TEMED) in water and 1 μL of 10 % ammonium persulfate in water. The blue light was turned on and the decays of the fluorescence were collected on the camera at 3 Hz (see Figure S31b). The

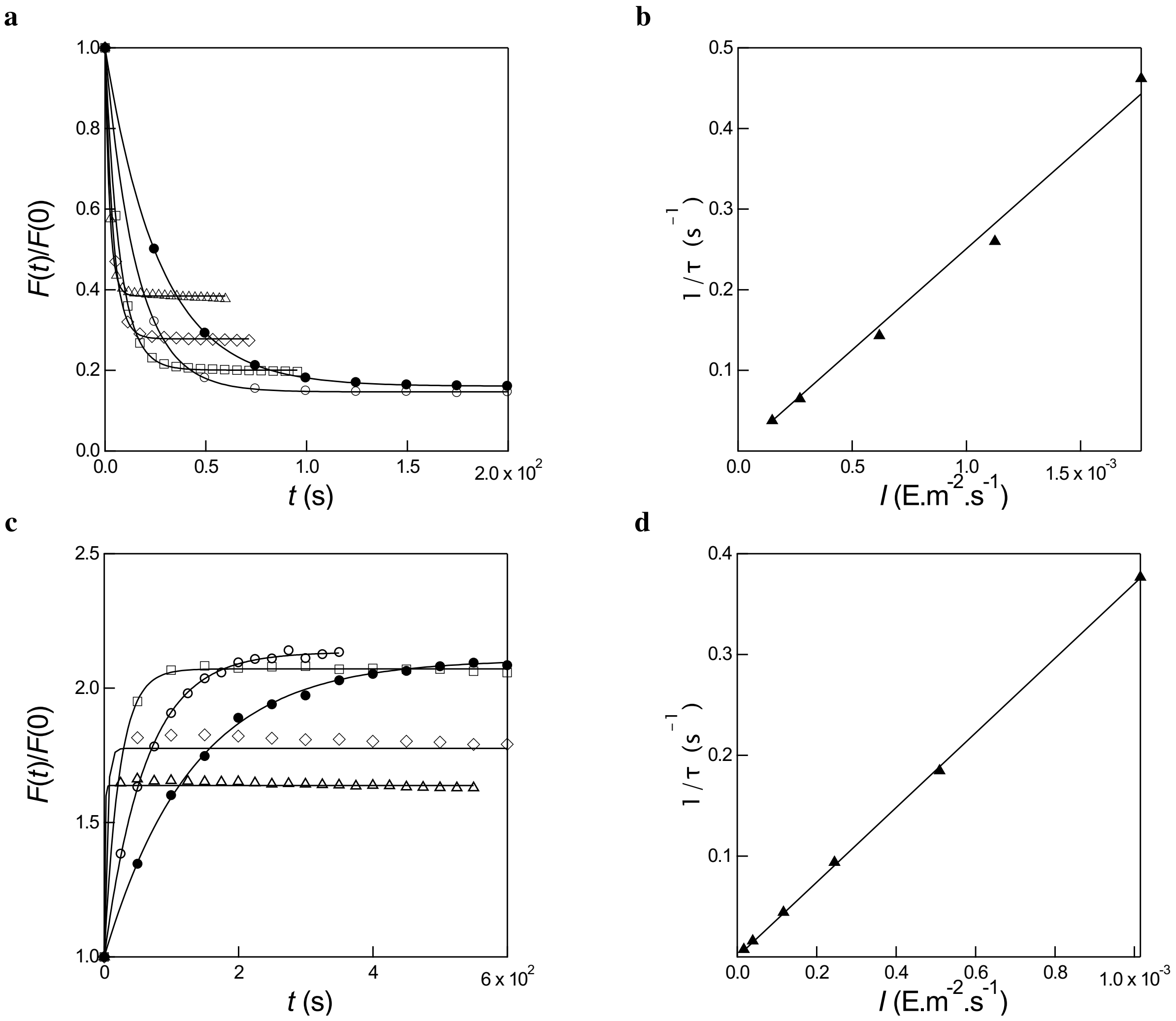

Figure S30: *Determination of the cross section of photoconversion of* ***Dronpa-2*** *in* ***Dronpa-2*** *labeled bacteria at 480 and 405 nm.* **a**: Time evolution of the normalized fluorescence emission at 515 nm from **Dronpa-2** labeled bacteria in DPBS $1\times$ pH 7.4 buffer in 3 mm optical path cuvette upon irradiation at various constant light intensities at 480 nm (in 10^{-4} E.m^{-2}.s^{-1}; disks: 1.5; circles: 2.7; squares: 6.2; diamonds: 11.2; triangles: 17.7) and constant light intensity at 405 nm equal to 3.9 10^{-5} E.m^{-2}.s^{-1}. Markers: Experimental data; Solid lines: Monoexponential fit with Eq.(S1); **b**: Dependence of the inverse of the characteristic time τ retrieved in **a** from the monoexponential fit on the light intensity. Markers: Experimental data; Solid line: Linear fit. The extracted slope is 251 ± 7 m^2.mol^{-1}; **c**: Time evolution of the normalized fluorescence emission at 515 nm from **Dronpa-2** labeled bacteria in 3 mm optical path cuvette upon irradiation at various constant light intensities at 405 nm (in 10^{-5} E.m^{-2}.s^{-1}; disks: 1.7; circles: 3.9; squares: 11.6; diamonds: 24.5; triangles: 51.0) and constant light intensity at 480 nm equal to 6.2 10^{-4} E.m^{-2}.s^{-1}. Markers: Experimental data; Solid lines: Monoexponential fit with Eq.(S1); **d**: Dependence of the inverse of the characteristic time τ retrieved in **c** from the monoexponential fit on the light intensity. Markers: Experimental data; Solid line: Linear fit. The extracted slope is 370 ± 2 m^2.mol^{-1}.

retrieved characteristic times τ decays were found similar in the solution ($\tau = 0.91$ s) and in the gel ($\tau = 1.03$ s) so as to support that the cross sections of photoconversion of **Dronpa-2** in solution and in a polyacrylamide gel are essentially identical under these experimental conditions.

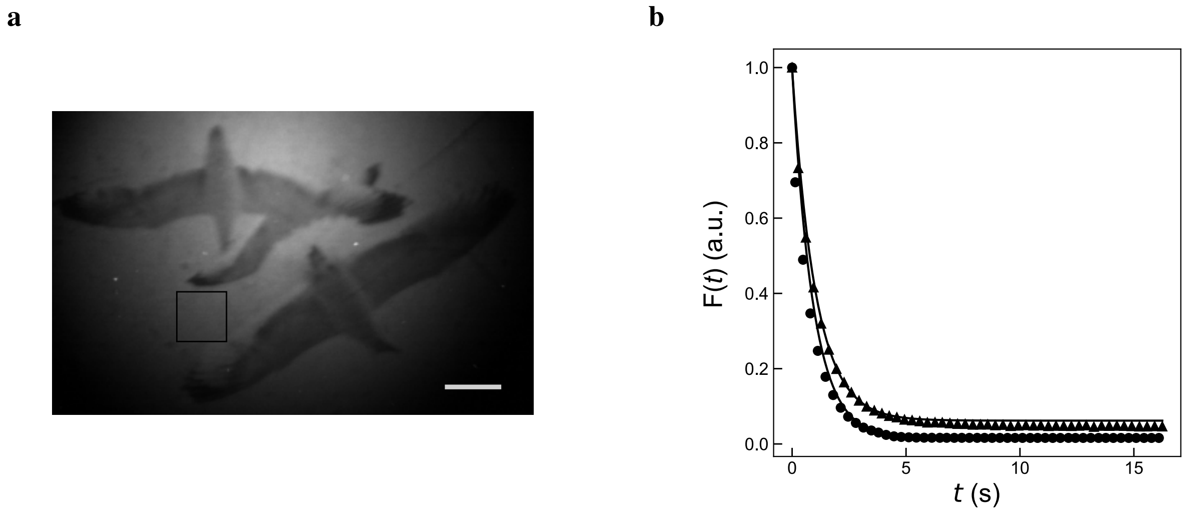

Figure S31: *Comparison of the fluorescence decay associated to the photoconversion of* ***Dronpa-2*** *in solution and in a polyacrylamide gel.* **a**: 100×100 pixels region of interest selected for analysis in the image generated in the polyacrylamide gel (scale bar: 100 μm); **b**: Averaged decays of the fluorescence signal over the selected area. Experimental data: disk (solution) triangles (gel); monoexponential fits: solid lines (solution: $\tau = 0.91$ s, polyacrylamide gel: $\tau = 1.03$ s).

Dependence of the cross sections of the Dronpa-2 photoconversion on the excitation wavelength Then we studied the dependence of the cross sections of photoconversion of **Dronpa-2** in **Dronpa-2** solution and in **Dronpa-2** labeled bacteria on the excitation wavelength.

Figures S32a,b display the time dependence of the fluorescence emission of a 8.8 μM **Dronpa-2** solution (Figure S32a) and of **Dronpa-2** labeled bacteria (Figure S32b) in DPBS $1\times$ pH 7.4 buffer upon irradiation at constant light intensity at 445, 480, and 500 nm in a 3 mm optical path quartz cuvette. Then we applied the monoexponential fitting function given in Eq.(S1) to the temporal rises of the normalized fluorescence at 515 nm in order to retrieve the rate constants of the photoconversion upon exciting at various wavelengths. Equipped with the rate constants of the photoconversion and the light intensities (retrieved from the light powers) measured at all the excitation wavelengths, we used Eq.(S23) to extract the dependence of the cross sections of photoconversion of **Dronpa-2** in the **Dronpa-2** solution and in **Dronpa-2** labeled bacteria on the excitation wavelength upon fixing their values to 198 and 251 $m^2.mol^{-1}$ at 480 nm respectively. The results are displayed in Table S5. By further using the dependence of the molar absorption coefficient of **Dronpa-2** in in DPBS $1\times$ pH 7.4 buffer on the wavelength, we extracted the quantum yield associated to **Dronpa-2** photoswitching at 445, 480, and 500 nm in **Dronpa-2** solution and in **Dronpa-2** labeled bacteria respectively. As displayed in Table S5, the extracted values are all in the 1–2×10^{-2} range and do not considerably depend on the excitation wavelength.

Estimate of the achievable measurement uncertainty on light intensity I Considering the uncertainty in the parameters (σ and τ), we estimate that the achievable measurement uncertainty on light intensity I is 20% by using **Dronpa-2**.

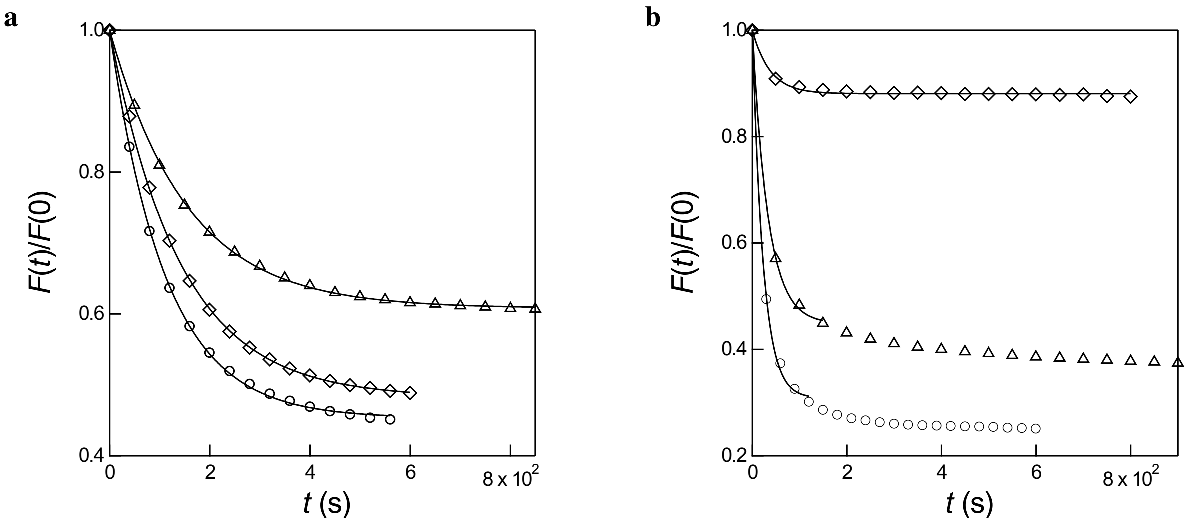

Figure S32: *Dependence of the cross sections of photoconversion of* ***Dronpa-2*** *on the excitation wavelength from analyzing the decay of the* ***Dronpa-2*** *fluorescence under illumination.* Time dependence of the normalized fluorescence emission at 515 nm of a 8.8 μM **Dronpa-2** solution (**a**) and **Dronpa-2** labeled bacteria (**b**) in DPBS 1$\times$ pH 7.4 buffer in 3 mm optical path cuvette upon illumination at constant light intensity at 445 (triangles), 480 nm (circles), and 500 (diamonds) nm. Markers: Experimental data; solid lines: Monoexponential fit with Eq.(S1). $T = 293$ K.

Table S5: Photoconversion parameters of **Dronpa-2** in **Dronpa-2** solution and in **Dronpa-2** labeled bacteria in DPBS 1$\times$ pH 7.4 buffer. The cross sections of photoconversion, $\sigma_{\rm sol}(\lambda_{\rm exc})$ and $\sigma_{\rm bac}(\lambda_{\rm exc})$, have been extracted from analyzing the decay of the fluorescence emission at 515 nm of a 8.8 μM **Dronpa-2** solution and **Dronpa-2** labeled bacteria respectively, under monochromatic illumination at 445, 480, and 500 nm upon adopting the value at 480 nm (which was measured in subsection 7.4.2) and under fixing the rate constant associated to thermal return k_{21}^{Δ} (3.2 10^{-3} s^{-1} in the **Dronpa-2** solution and in the **Dronpa-2** labeled bacteria respectively). The molar absorption coefficients $\varepsilon(\lambda_{\rm exc})$ were extracted from the **Dronpa-2** absorption spectrum in **Dronpa-2** solution in DPBS 1$\times$ pH 7.4 buffer by using the Beer-Lambert law. The quantum yields of **Dronpa-2** photoconversion in **Dronpa-2** solution and of **Dronpa-2** labeled bacteria, $\varphi_{\rm sol}$ and $\varphi_{\rm bac}$ respectively, were evaluated from the cross sections of photoconversion with Eq.(S23). $[I(\lambda_{\rm exc})]$ indicates the range of reliably measurable light intensity associated to the actinometer photoconversion at $\lambda_{\rm exc}$.

$\lambda_{\rm exc}$ (nm)	$10^4\varepsilon_{\rm sol}(\lambda_{\rm exc})$ ($\pm$0.1; M^{-1}cm^{-1})	$\sigma_{\rm sol}(\lambda_{\rm exc})$ ($\pm$10%; m^{2}mol^{-1})	$10^2\varphi_{\rm sol}$ ($\pm$0.2)	$\sigma_{\rm bac}(\lambda_{\rm exc})$ ($\pm$10%; m^{2}mol^{-1})	$10^2\varphi_{\rm bac}$ ($\pm$0.2)	$[I(\lambda_{\rm exc})]^a$ (E.m^{-2}.s^{-1} (W.m^2))
445	3.8	140	1.6	192	2.2	[3 10^{-4}–18] ([80–4.8 10^6])
480	7.0	198	1.2	251	1.6	[2 10^{-4}–10] ([50–2.5 10^6])
500	5.3	128	1.0	151	1.3	[3 10^{-4}–13] ([72–1.0 10^6])

^{a} The lower range of $I(\lambda_{\rm exc})$ has been computed from Eq.(S60) upon using the values of k_{21}^{Δ} and $\sigma_{\rm sol}(\lambda_{\rm exc})$. The upper range of $I(\lambda_{\rm exc})$ has been retrieved from the value of $I_1^{\rm c}(480\ {\rm nm})$ given in Supplementary Table 3 of the reference[7] by using $I_{\rm sup}(\lambda_{\rm exc}) = I_1^{\rm c}(480\ {\rm nm}) \times \frac{\varepsilon(480\ {\rm nm})}{\varepsilon(\lambda_{\rm exc})}$.

7.4.3 DASA

Photochromic dyes provide attractive reusable actinometers since they reversibly change of color upon photoreaction.[16] Yet, only a few of them absorb up to the red wavelength range. Donor-acceptor Stenhouse dyes reversibly photoswitch between an open conjugated state absorbing above 500 nm, and a cyclic non-conjugated state that absorbs below 300 nm.[17] We recently introduced such a photochromic dye – subsequently denoted **DASA**, which has been thoroughly investigated by Jullien, Adelizzi et al.[5] In acetonitrile, it exhibits a broad absorption band spanning the 530-670 nm range, which is

associated to an open form. It encounters a reversible two-step light-triggered conversion under illumination at 595 nm: A Z/E photoisomerization is followed by a thermally-driven cyclization resulting in the cyclic colorless form.

Preparation of the DASA solution The synthesis of the sodium 4-(4-((2Z,4E)-2-hydroxy-5-(indolin-1-yl)penta-2,4-dien-1-ylidene)-3-methyl-5-oxo-4,5-dihydro-1H-pyra-zol-1-yl)benzenesulfonate (**DASA**) actinometer is reported in the Methods in the Main Text. The preparation of the **DASA** solution is performed in two steps. A first 0.2 mM mother **DASA** solution is prepared in spectroscopy grade DMSO. It can be kept under the protection of ambient light (container covered with an Aluminium foil) at 20 °C for up to a week. The mother solution is then diluted in spectroscopy grade acetonitrile to yield a final 40 μM **DASA** in a 1:50 (v/v) (or 20 μM **DASA** in a 1:100 (v/v)) mixture of spectroscopy grade DMSO and acetonitrile. This solution has to be freshly prepared and equilibrated for 2 h before immediate use.

Absorption and emission spectra of DASA In acetonitrile, the **DASA** open state absorbs light between 530 and 670 nm and emits fluorescence emission, which peaks at 650 nm and extends up to 675 nm (see Figure S33).

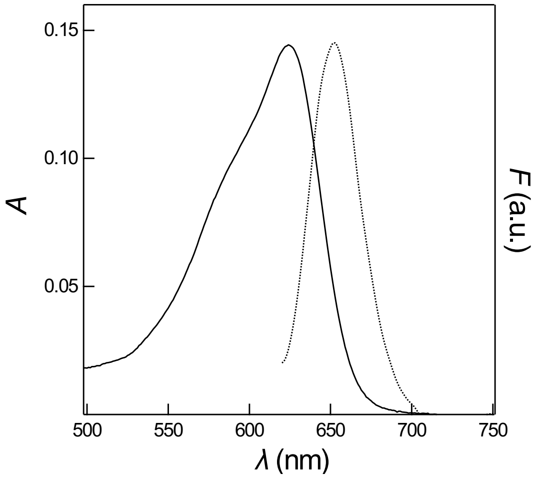

Figure S33: Absorption (solid line) and emission (dotted line; $\lambda_{exc} = 600$ nm) spectra of 30 μM **DASA** solution in a 1:50 (v/v) mixture of spectroscopy grade DMSO and acetonitrile recorded in a 3 mm quartz cuvette. $T = 293$ K.

Determination of the quantum yield of DASA photoconversion in acetonitrile at 632 nm We evaluated the quantum yield of **DASA** photoconversion in acetonitrile at 632 nm and examined whether there is a possible upper range of light intensity beyond which the photochemical step is not anymore rate-limiting in the **DASA** light-driven conversion.

Figure S34a displays the time evolution of the fluorescence emission of 40 μM **DASA** in a 1:50 (v/v) mixture of spectroscopy grade DMSO and acetonitrile contained in a 3 mm optical path cuvette upon illumination at various light intensities at 632 nm measured with a powermeter. The monoexponential fitting function given in Eq.(S1) was applied to the temporal decays of the normalized fluorescence emission at 675 nm in order to retrieve the characteristic times of the photoconversion τ upon exciting at various light intensities. As displayed in Figure S34b, the inverse of the characteristic

times of the photoconversion linearly depends on the light intensity. This behavior demonstrates that the rate limiting step of **DASA** photoconversion in acetonitrile is photochemical at least up to 6 10^{-4} E.m^{-2}.s^{-1} light intensity. Eq.(S24) was used to retrieve $\sigma = 1135 \pm 50$ m^2.mol^{-1} from the slope.

This experiment was reproduced on a microscopy setup with MPPC detection of the emitted fluorescence. 40 and 100 μM **DASA** in a 1:35 (v/v) mixture of spectroscopy grade DMSO and acetonitrile contained in a 200 μm-high glass slide chamber were exposed to illumination at various light intensities at 630 ± 7 nm (measured with a powermeter) and their emission was collected at 690 ± 8 nm. As displayed in Figure S34c, very similar results have been obtained with σ values ranging from 1128 ± 40 to 1136 ± 50 m^2.mol^{-1}.

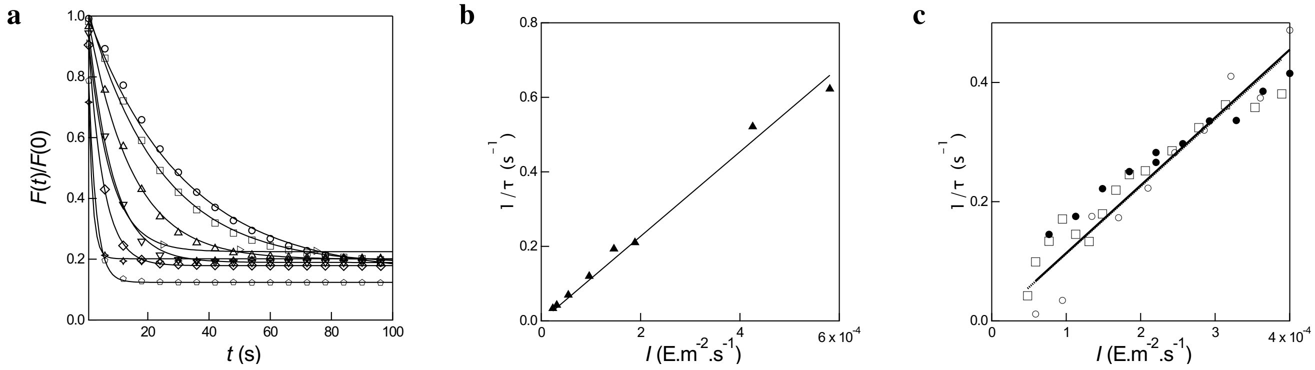

Figure S34: *Determination of the quantum yield of photoconversion of sodium 4-(4-((2Z,4E)-2-hydroxy-5-(indolin-1-yl)penta-2,4-dien-1-ylidene)-3-methyl-5-oxo-4,5-dihydro-1H-pyra-zol-1-yl)benzenesulfonate (**DASA**).* **a**: Time evolution of the normalized fluorescence emission at 675 nm from a 40 μM **DASA** solution in a 1:50 (v/v) mixture of spectroscopy grade DMSO and acetonitrile contained in a 3 mm optical path cuvette upon illumination at various light intensities at 632 nm (in $10^{-5}\times$ E.m^{-2}.s^{-1}: 2.3, 3.1, 5.5, 9.7, 15, 19, 43, and 58). Markers: Experimental data; Solid lines: Monoexponential fit with Eq.(S1); **b**: Dependence of the inverse of the characteristic time τ retrieved in **a** from the monoexponential fit on the light intensity. Markers: Experimental data; Solid line: Linear fit. The extracted slope is 1135 m^2.mol^{-1} **c**: Dependence of the inverse of the characteristic time τ retrieved in the experiments performed on **DASA** solutions in a 1:35 (v/v) mixture of spectroscopy grade DMSO and acetonitrile contained in a 200 μm-high glass slide chamber upon illumination at various light intensities with a microscopy setup equipped with MPPC detection of the emitted fluorescence. Excitation wavelength: 630 ± 7 nm; emission wavelength: 690 ± 8 nm. Markers: The disks and the circles are associated to two series of experiments on 40 μM **DASA** solution and the squares have been recorded on a 100 μM **DASA** solution. The solid and dotted lines result from the monoexponential fit on the light intensity of the data gathered on 40 and 100 μM **DASA** solutions respectively. The extracted slopes are 1135 ± 50 and 1128 ± 40 m^2.mol^{-1} respectively.

Dependence of the quantum yield of the DASA photoconversion on the excitation wavelength Then we studied the dependence of the quantum yield of the **DASA** photoconversion on the excitation wavelength. Two different protocols have been implemented.

Decay of the DASA absorbance under illumination Figure S35a displays a representative time dependence of the absorption spectrum of 40 μM **DASA** in a 1:50 (v/v) mixture of spectroscopy grade DMSO and acetonitrile upon irradiation at constant light intensity at 632 nm. This experiment has been reproduced upon exciting at various wavelengths

while recording the light power transmitted through the 3 mm light path quartz cuvette. Then we applied the monoexponential fitting function given in Eq.(S1) to the temporal decays of the normalized absorbance at 632 nm in order to retrieve the rate constants of the photoconversion upon exciting at various wavelengths (Figure S35b). Equipped with the rate constants of the photoconversion and the light intensities (retrieved from measuring the light powers with a powermeter) measured at all the excitation wavelengths, we used Eq.(S23) to extract the dependence of the cross section of the **DASA** photoconversion on the excitation wavelength upon fixing its value to 1135 $m^2.mol^{-1}$ at 632 nm. The results are displayed in Table S6. The quantum yield of the **DASA** photoconversion is demonstrated to be essentially constant over the whole range of investigated wavelengths.

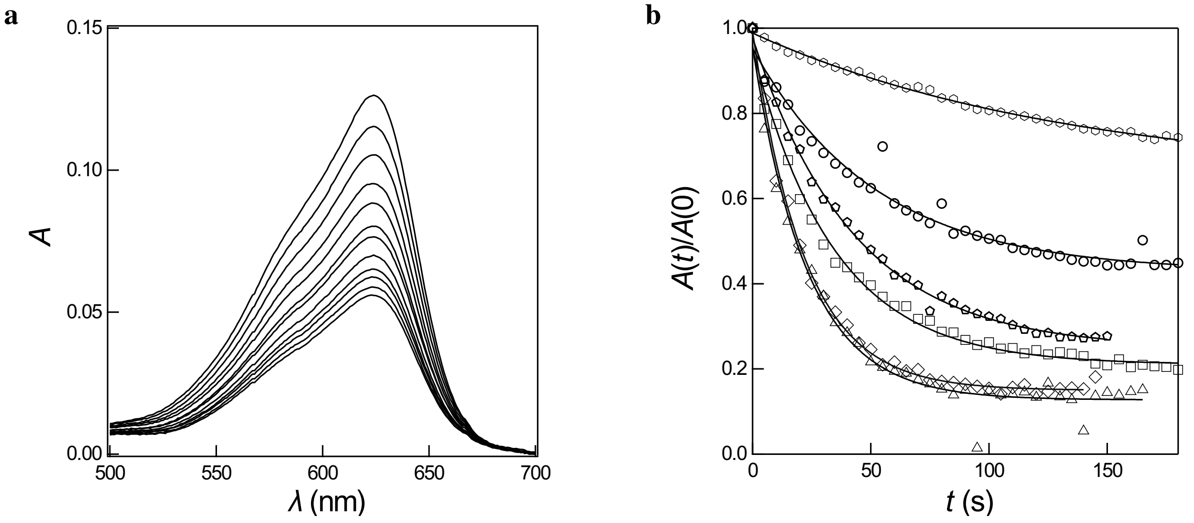

Figure S35: *Dependence of the cross section of the **DASA** photoconversion on the excitation wavelength from analyzing the decay of the **DASA** absorbance under illumination.* **a**: Time dependence of the absorption spectrum of 40 μM **DASA** in a 1:50 (v/v) mixture of spectroscopy grade DMSO and acetonitrile in a 3 mm light path quartz cuvette upon irradiation at constant light intensity at 632 nm in 5 s increments from the initial time; **b**: Time dependence of the normalized absorbance at 632 nm of 40 μM **DASA** solution in a 1:50 (v/v) mixture of spectroscopy grade DMSO and acetonitrile upon illumination at constant light intensity at 530 (circles), 560 nm (squares), 600 (triangles), 632 (diamonds), 650 (pentagons), and 670 (hexagons) nm. Markers: Experimental data; Solid lines: Monoexponential fit with Eq.(S1). T = 293 K.

Decay of the DASA fluorescence under illumination Figure S36 displays the time dependence of the fluorescence emission of 20 μM **DASA** in a 1:100 (v/v) mixture of spectroscopy grade DMSO and acetonitrile upon irradiation at constant light intensity at 530, 560, 600, 632, 650, and 670 nm, which have been measured from recording the light power transmitted through the 3 mm light path quartz cuvette. Then we applied the monoexponential fitting function given in Eq.(S1) to the temporal decays of the normalized fluorescence in order to retrieve the rate constants of the photoconversion upon exciting at various wavelengths. Equipped with the rate constants of the photoconversion and the light intensities (retrieved from the light powers) measured at all the excitation wavelengths, we used Eq.(S23) to extract the dependence of the cross section of the **DASA** photoconversion on the excitation wavelength upon fixing the value of the cross section of the **DASA** photoconversion to 1135 $m^2.mol^{-1}$ at 632 nm. The results are displayed in Table

S6. Again, the quantum yield of the **DASA** photoconversion is essentially constant over the whole range of investigated wavelengths. Hence, the value of the cross section for the **DASA** photoconversion can be reliably computed from the dependence of its molar absorption coefficient on the wavelength given in Eq.(S23).

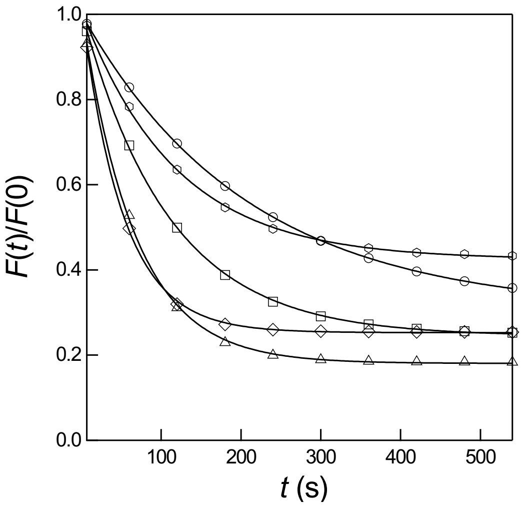

Figure S36: *Dependence of the cross section of the* ***DASA*** *photoconversion on the excitation wavelength from analyzing the drop of endogeneous fluorescence under illumination.* Time dependence of the normalized fluorescence emission of a 20 μM **DASA** solution in a 1:100 (v/v) mixture of spectroscopy grade DMSO and acetonitrile in a 3 mm light path quartz cuvette upon illumination at constant light intensity at 530 (circles), 560 nm (squares), 600 (triangles), 632 (diamonds), and 650 (hexagons) nm. Emission wavelength: 650 nm for illumination at 530, 560, 600, and 632 nm; 670 nm for 650 nm excitation; 685 nm for excitation at 670 nm. Markers: Experimental data; solid lines: Monoexponential fit with Eq.(S1). $T = 293$ K.

Estimate of the achievable measurement uncertainty on light intensity I Considering the uncertainty in the parameters (σ and τ), we estimate that the achievable measurement uncertainty on light intensity I is 20% by using **DASA**.

7.4.4 Photosynthetic apparatus (PA)

The preceding chemical actinometers provide accurate values of light intensity. However, they are effective in a rather narrow range of wavelengths, which necessitates to use several actinometers to measure light intensity over a wide range of wavelengths. We have been interested to exploit the photosynthetic apparatus as an intrinsically fluorescent actinometer, which can provide two to three orders of magnitude of light intensity over the whole 400–650 nm range.

In oxygenic photosynthetic organisms, sunlight is collected with an efficient antenna absorbing light in the whole visible wavelength range.[18] The absorbed energy is conveyed to the photosystem II and photosystem I, where it drives a charge separation accompanied by water splitting leading to oxygen evolution, followed by assimilation of carbon dioxide to produce sugars. However, a small part of the absorbed energy (a few percent) is released as fluorescence emission (mostly by chlorophylls, Chl) spanning the 650–800 nm range with an emission maximum around 680 nm at room temperature. When dark-adapted photosynthetic samples are exposed to continuous constant light, the Chl a fluorescence intensity shows characteristic changes called fluorescence transient or OJIP rise. Primarily reporting on the

Table S6: Photoconversion parameters of sodium 4-(4-((2Z,4E)-2-hydroxy-5-(indolin-1-yl)penta-2,4-dien-1-ylidene)-3-methyl-5-oxo-4,5-dihydro-1H-pyra-zol-1-yl)benzenesulfonate (**DASA**) in mixtures of spectroscopy grade DMSO and acetonitrile. The quantum yields of photoconversion, φ_A and φ_F have been respectively extracted from analyzing the decay of the absorbance at 632 nm of a 40 μM **DASA** 1:50 (v/v) DMSO:acetonitrile solution, and the decay of the fluorescence emission of a 20 μM **DASA** in 1:100 (v/v) DMSO:acetonitrile solution under monochromatic illumination at 530, 560, 600, 632, 650, and 670 nm as representatives of 530 nm – 670 nm range. φ is the mean value of φ_A and φ_F. The molar absorption coefficients $\varepsilon(\lambda_{\rm exc})$ were extracted from the **DASA** absorption spectrum by fixing $\varepsilon(632\ \rm nm) = 87 \times 10^3$ $\rm M^{-1}cm^{-1}$.[5] The cross section $\sigma(\lambda_{\rm exc})$ was evaluated with Eq.(S23). $[I(\lambda_{\rm exc})]$ indicates the range of reliably measurable light intensity associated to the actinometer photoconversion at $\lambda_{\rm exc}$.

$\lambda_{\rm exc}$ (nm)	$10^4 \times \varepsilon(\lambda_{\rm exc})$ (±0.1; $\rm M^{-1}cm^{-1}$)	φ_F (±0.01)	φ_A (±0.01)	φ (±0.01)	$\sigma(\lambda_{\rm exc})$ ($\pm10\%$; $\rm m^2mol^{-1}$)	$[I(\lambda_{\rm exc})]^a$ ($\rm E.m^{-2}.s^{-1}$ ($\rm W.m^2$))
530	1.8	0.05	0.07	0.06	255	[8-290] 10^{-5}([18-660])
560	3.5	0.05	0.08	0.07	530	[4-150] 10^{-5}([9-320])
600	7.2	0.05	0.06	0.05	885	[2-72] 10^{-5}([4-140])
632	8.7	0.06	0.06	0.06	1135	[2-60] 10^{-5}([4-110])
650	3.8	0.06	0.07	0.07	575	[3-140] 10^{-5}([6-260])
670	0.6	$-^b$	0.14^c	0.14^c	210^c	–

^{a} The lower range of $I(\lambda_{\rm exc})$ has been computed from Eq.(S60) upon using the values of k_{21}^{Δ} from reference[5] and $\sigma_{\rm sol}(\lambda_{\rm exc})$. The upper range of $I(\lambda_{\rm exc})$ has been retrieved from using $I_{\rm sup}(\lambda_{\rm exc}) = I_{\rm sup}(\lambda_{\rm exc,ref}) \times \frac{\varepsilon(\lambda_{\rm exc,ref})}{\varepsilon(\lambda_{\rm exc})}$ where $\lambda_{\rm exc,ref}$ designates the wavelength at which the extraction of the cross section of photoconversion has been performed ($\lambda_{\rm exc,ref} = 632$ nm for **DASA**).
^{b} Too slow a relaxation to be reliably measured.
^{c} The slow relaxation induced a higher error on the extraction of φ_A (and subsequently of φ and $\sigma(670\ \rm nm)$).

successive reduction of the electron acceptors of the photosynthetic electron transport chain, the chlorophyll fluorescence intensity rises in less than 1 s from a minimum level (the O level) to a maximum level (P). Depending on the light level and the experimental conditions, the curve displays one, two or three intermediate steps identified as local maxima labeled K, J and I, and subsequently decays over a few minutes.[19] The first step systematically reflects the light-limited reaction (reduction of the electron acceptor Qa). This so-called photochemical phase, linearly depends on the intensity of the exciting light.[20,21] Moreover, its value does not significantly depend on the nature of the photosynthetic organism,[6] so as to facilitating getting samples for light measurement.

Conditioning of the PA-containing samples The production of the **PA**-containing samples is reported in the Methods in the Main Text. All the samples have been submitted to darkness for 15 min before starting the illumination experiments.

Fluorescence excitation and emission spectra of PA Photosynthetic organisms collect sunlight over the whole visible wavelength range by means of an antenna integrating multiple chromophores. The associated energy is subsequently funneled to the reaction center, where the charge separation governing photosynthesis occurs. Considering that fluorescence emission is a side-product of the light-driven process of charge separation driving the initial step of **PA** fluorescence rise under constant illumination, we recorded the fluorescence excitation spectrum of a culture of *Chlamydomonas reinhardtii (CC_124)* in exponential growth phase (Figure S37). It exhibits strong values in the purple-blue and red wavelength ranges and a minimum at green wavelengths.

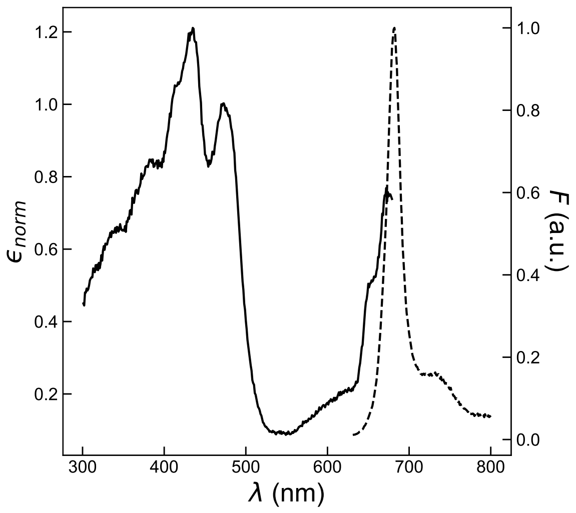

Figure S37: *Fluorescence excitation and emission spectra of **PA***. Normalized fluorescence excitation $\epsilon_{\rm norm}$ (solid line; $\lambda_{\rm em} = 690 \pm 2$ nm, normalized at 470 nm) and emission $F_{\rm norm}$ (dotted line; $\lambda_{\rm exc} = 470 \pm 2$ nm) spectra of a culture of *Chlamydomonas reinhardtii (CC_124)* in exponential growth phase. $T = 293$ K. The excitation spectrum has been normalized at 470 nm.

Determination of the cross section of PA photoconversion at 470 nm We then evaluated the cross section of **PA** photoconversion associated to the initial step of **PA** fluorescence rise from dark acclimated cells of *Chlamydomonas reinhardtii (CC_124)* (in exponential phase in minimal media) upon applying a 0.3–1 s-long[24] pulse of constant light at 470±10 nm. Figure S38a displays representative time evolutions of the normalized fluorescence emission at 470±10 nm from the dark acclimated cells observed with the photodetector under the epifluorescence microscope.[25]

After setting time zero to fluorescence crossing a threshold above zero corresponding to the light turning on and logarithmically subsampling the data to allow us for faster fitting, the fit of the time evolutions of the normalized fluorescence emission was performed stepwise:

- In a first step, we followed the analysis reported in reference[6] and applied an unsupervised fit with Eq.(S84)

$$F(t) = F(0) + A_{\rm OJ}\left(1 - e^{-t/\tau_{\rm OJ}}\right)^{s_{\rm OJ}} + A_{\rm JI}\left(1 - e^{-t/\tau_{\rm JI}}\right)^{s_{\rm JI}} + A_{\rm IP}\left(1 - e^{-t/\tau_{\rm IP}}\right)^{s_{\rm IP}} \quad \text{(S84)}$$

  in order to retrieve a first estimate of the value of the characteristic time $\tau_{\rm OJ}$ associated to the initial step of **PA** fluorescence rise ;

- In a second step, we restricted the time window to [0;3$\tau_{\rm OJ}$] and applied the fit given in Eq.(S85) to the time evolution of the fluorescence emission

$$F(t) = F(0) + A\left(1 - e^{-t/\tau}\right)^{s} \quad \text{(S85)}$$

[24]Depending on the light level.

[25]Recording started 10 ms before the LED was turned on. The rise-time of the LED is always faster than 1 μs while the dynamics of the initial step of **PA** fluorescence rise is in the tenths of microseconds range. Therefore the rise time of the LED does not interfere with the recording of the fluorescence rise dynamics.

upon fixing $s = 1.24^6$ in order to retrieve a second estimate of the value of the characteristic time τ associated to the intial rise of the **PA** fluorescence;

- In the last step, we restricted the time window to [0;5τ], applied the fit given in Eq.(S85) to the time evolution of the fluorescence emission upon adopting the values of parameters extracted during the second step as starting values, and retrieved the final value of the characteristic time τ associated to the initial step of **PA** fluorescence rise .

This fitting protocol was found relevant as displayed in Figures S38a,b. Moreover it was typically found accurate above 50 μE.m^{-2}.s^{-1} under blue illumination.

As displayed in Figure S38c, the inverse of the characteristic time of the photoconversion associated to the initial step of **PA** fluorescence rise linearly depends on the light intensity. This behavior demonstrates that the process driving the initial step of **PA** fluorescence rise evolution is photochemical at least up to 10^{-2} E.m^{-2}.s^{-1} light intensity. It also enabled us to retrieve (1.8 $\pm$ 0.1)$\times 10^6$ m^2.mol^{-1} as the cross section of **PA** photoconversion associated to the initial step of **PA** fluorescence rise from the slope.

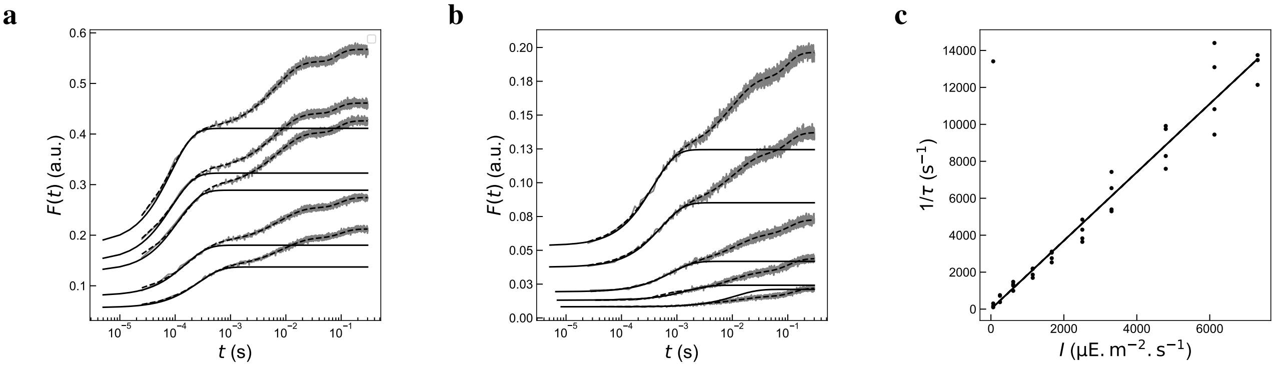

Figure S38: *Determination of the cross section associated with the initial step of **PA** fluorescence rise from dark acclimated microalgae (Chlamydomonas reinhardtii (CC_124) in exponential phase in minimal media) cells upon constant illumination at 470$\pm$10 nm.* **a,b**: Time evolution of the **PA** fluorescence emission under constant illumination at 470 nm in the epifluorescence microscope at various light intensities (previously calibrated with **Dronpa-2**) in the field of view. **a**: 7300, 6100, 4800, 3300, 2500 μE.m^{-2}.s^{-1}; **b**: 1700, 600, 250, 60 μE.m^{-2}.s^{-1}. A moving average was applied on the experimental data (grey markers) before subsampling with window sizes of 50 and 10 for the fits given in Eq.(S84) (dashed lines) and (S85) (solid lines) respectively to retrieve the characteristic time τ(μs)= 74, 76, 100, 134, 232, 327, 455, 676, 1320, 11000 associated to the initial step of **PA** fluorescence rise ; **c**: Light intensity-dependence of the inverse of the characteristic time τ associated to the initial step of **PA** fluorescence rise , which was retrieved from the monoexponential fit on the light intensity in the range 60–7300 μE.m^{-2}.s^{-1} by using the 470$\pm$10 nm LED of the epifluorescence microscope. Markers: experimental data, solid line: linear fit (slope $\sigma(470) = (1.8 \pm 0.1)\times 10^6$ m^2.mol^{-1}).

Dependence of the cross section of the PA photoconversion on the excitation wavelength The dependence of the cross section of the **PA** photoconversion on the excitation wavelength has been further investigated with the epifluorescence setup at two other wavelengths from two different LEDs (6 repeats: 3 with the strain CC_124 and 3 with the strain $WT4A$):

- A purple LED emitting at 405±7 nm. It was calibrated with a **Dronpa-2** solution as an actinometer[7] and used in the range 250–4000 μE.m^{-2}.s^{-1} (80–1200 W.m^{-2});

- A red-orange LED emitting at 650 nm. It was calibrated with the powermeter and used in the range 200–6500 μE.m^{-2}.s^{-1} (40–1200 W.m^{-2});

with collection of emitted fluorescence at 690 nm.

From the dependence of the inverse of the characteristic time on the light intensity (data not shown), we obtained (2.0± 0.06)×10^6 m^2.mol^{-1} and (1.0± 0.02)×10^6 m^2.mol^{-1} associated to the purple and and red-orange lights respectively.

Upon fixing the value of the cross section of the **PA** photoconversion to 2.0×10^6 m^2.mol^{-1} at 470 nm as a reference, we could scale the fluorescence excitation spectrum displayed in Figure S37 in order to generate the scaled fluorescence excitation spectrum $\sigma_{\rm mic}(\lambda)$ (see Figure S39). Figure S39 further displays the set of cross sections that have been directly measured in the preceding experiments. The fair observed agreement between the prediction from the scaled fluorescence excitation spectrum and the experimental values suggests the former to be relevant for retrieving the cross section of the **PA** photoconversion in the [400 nm; 650 nm] wavelength range.

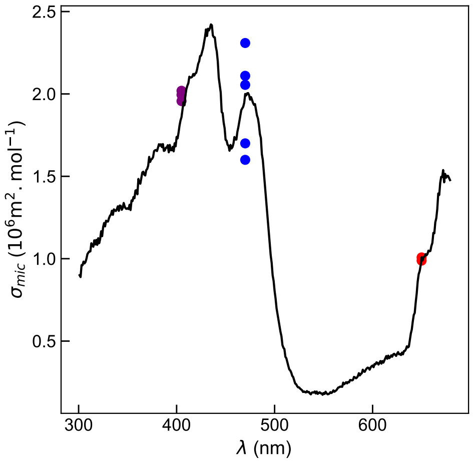

Figure S39: *Fluorescence excitation spectrum* $\sigma_{\rm mic}(\lambda_{\rm exc})$ *of* ***PA*** *in Chlamydomonas reinhardtii (CC_124)* ($\lambda_{\rm em}$ = 690 nm). Scaling reference: $\sigma(470)$ = 2.0 ×10^6 m^2.mol^{-1}. The color markers represent the $\sigma(\lambda_{exc})$ measured experimentally using three LEDs.

Table S7 sums up the relevant parameters which have to be used for measuring the light intensity at various wavelengths with the **PA** actinometer.

Estimate of the achievable measurement uncertainty on light intensity I Considering the uncertainty in the parameters (σ and τ) and the specific nature of the fitting function applied to the time evolution of the fluorescence signal, we estimate that the achievable measurement uncertainty on light intensity I is 70% by using **PA**.

Table S7: *Photoconversion parameters associated to the initial step of **PA** fluorescence rise* . The normalized excitation coefficient $\epsilon_{\mathrm{norm}}(\lambda_{\mathrm{exc}})$ was extracted from the **PA** fluorescence excitation spectrum displayed in Figure S37. The cross sections $\sigma(\lambda_{\mathrm{exc}})$ and $\sigma_{\mathrm{mic}}(\lambda_{\mathrm{exc}})$ have respectively been measured (see Figure S39) and computed at 405 and 650 nm from using the excitation spectra of the dark acclimated *Chlamydomonas reinhardtii (CC_124)* cells (in exponential phase in minimal media) upon fixing the value of the cross section of the **PA** photoconversion to 2.0 $\times 10^6$ $m^2.mol^{-1}$ at 470 nm. $I_{\mathrm{sup}}(\lambda_{\mathrm{exc}})$ indicates the upper light intensity tested and relevant for a reliable measurement.

λ_{exc} (nm)	$\epsilon_{\mathrm{norm}}(\lambda_{\mathrm{exc}})$	$\sigma(\lambda_{\mathrm{exc}})$ ($10^6 m^2 mol^{-1}$)	$\sigma_{\mathrm{mic}}(\lambda_{\mathrm{exc}})$ ($10^6 m^2 mol^{-1}$)	$I_{\mathrm{sup}}(\lambda_{\mathrm{exc}})$ ($E.m^{-2}.s^{-1}$ ($W.m^2$))
405	1.0 ± 0.05	2.0 ± 0.06	2.0 ± 0.4^a	$10^{-2}(3000)^b$
470	1.0 ± 0.05	2.0 ± 0.4	2.0 ± 0.4^a	$10^{-2}(2600)^b$
650	0.55 ± 0.05	1.0 ± 0.02	1.1 ± 0.4^a	$10^{-2}(1900)^b$

[a]Estimated from 6 repeats at 470 nm on two different *Chlamydomonas reinhardtii* strains.
[b]Extracted from the value of $I_{\mathrm{sup}}(\lambda_{\mathrm{exc}})$ by using $I_{\mathrm{sup}}(\lambda_{\mathrm{exc}}) = I_{\mathrm{sup}}(\lambda_{\mathrm{exc,ref}}) \times \frac{\epsilon(\lambda_{\mathrm{exc,ref}})}{\epsilon(\lambda_{\mathrm{exc}})}$ where $\lambda_{\mathrm{exc,ref}}$ designates the wavelength at which the extraction of the cross section of photoconversion has been performed ($\lambda_{\mathrm{exc,ref}} = 470$ nm for **PA**).

7.5 Combination of a non-fluorescent actinometer with a fluorescent reporter

7.5.1 α-(4-Diethylamino)phenyl)-N-phenylnitrone (Nit)

This actinometer has been thoroughly investigated.[10,22] It has been shown to irreversibly convert into N-(p-dimethylamino-phenyl)formanilide in ethanol under illumination in the UV A wavelength range.

Preparation of the Nit solutions The synthesis of α-(4-diethylamino)phenyl)-N-phenylnitrone (**Nit**) actinometer is reported in the Methods in the Main Text. Stock solutions of α-(4-diethylamino)phenyl)-N-phenylnitrone (3.0 mg/ml; 12.5 mM) and Rhodamine B (2.9 mg/mL; 6 mM) were produced in spectrograde ethanol. They can be kept under the protection of ambient light (container covered with an Aluminium foil) at -20 ^{o}C for up to two weeks. They were subsequently diluted in spectrograde ethanol to produce the final solutions upon taking care to exhibit an absorbance at the absorption maximum lower or equal to 0.15.

Absorption and emission spectra of Nit In ethanol, the **Nit** open state absorbs light between 320 and 430 nm. Rhodamine B emits fluorescence emission, which peaks at 574 nm and extends from 550 up to 650 nm (see Figure S40).

Determination of the quantum yield of Nit photoconversion in ethanol at 405 nm The value of the quantum yield of the **Nit** photoconversion has been already measured to be 0.16 at 365 nm.[22] In this manuscript, we independently evaluated this value at 405 nm. We also examined whether there is a possible upper range of light intensity beyond which the photochemical step is not anymore rate-limiting in the light-driven **Nit** conversion into N-(p-dimethylaminophenyl)formanilide in ethanol.

Figure S41a displays the time dependence of the fluorescence emission of 14 μM **Nit** and 1.0 μM **RhB** in spectroscopy grade ethanol upon irradiation at various light intensities at 405 nm, which have been measured from analyzing the time

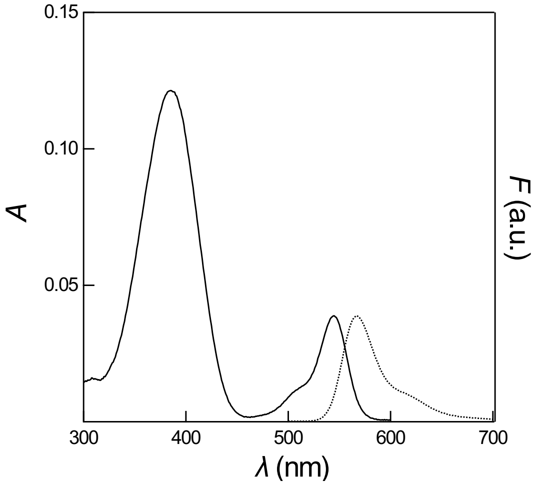

Figure S40: Absorption (solid line) and emission (dotted line; $\lambda_{\mathrm{exc}} = 384$ nm) spectra of 14 μM **Nit** and 1 μM **RhB** solution in EtOH recorded in a 1×1 cm^2 quartz cuvette. $T = 293$ K.

recovery of the fluorescence signal from 488 nm-photoswitched **Dronpa-2** upon illumination at 405 nm.[7,11] Then we applied the monoexponential fitting function given in Eq.(S1) to the temporal rises of the normalized fluorescence at 574 nm in order to retrieve the characteristic times of the photoconversion τ upon exciting at various light intensities. As displayed in Figure S41b, the inverse of the characteristic times of the photoconversion linearly depends on the light intensity. This behavior demonstrates that the rate limiting step of **Nit** photoconversion in ethanol is photochemical at least up to 7 10^{-4} E.m^{-2}.s^{-1} light intensity. Eq.(S23) was used to retrieve $\sigma = 1005 \pm 30$ m^2.mol^{-1} from the slope. Equipped with the latter value of the cross section for the photoconversion and the value of the molar absorption coefficient of **Nit** at 405 nm (3.1 10^4 M^{-1}.cm^{-1} = 3.1 10^3 m^2.mol^{-1}), we used Eq.(S23) to extract $\varphi_F = 0.14 \pm 0.02$ as the value of the quantum yield of the **Nit** photoconversion at 405 nm.

Dependence of the quantum yield of the Nit photoconversion on the excitation wavelength Then we studied the dependence of the quantum yield of the **Nit** photoconversion on the excitation wavelength. Two different protocols have been implemented.

Decay of the Nit absorbance under illumination Figure S42a displays a representative time dependence of the absorption spectrum of 5.8 μM **Nit** in spectroscopy grade ethanol upon irradiation at constant light intensity at 365 nm. This experiment has been reproduced upon exciting at various wavelengths while recording the light power transmitted through the 1 cm light path quartz cuvette. Then we applied the monoexponential fitting function given in Eq.(S86)

$$\frac{A(\lambda_{\mathrm{exc}},t)}{A(\lambda_{\mathrm{exc}},0)} = \frac{A(\lambda_{\mathrm{exc}},\infty)}{A(\lambda_{\mathrm{exc}},0)} + \left[1 - \frac{A(\lambda_{\mathrm{exc}},\infty)}{A(\lambda_{\mathrm{exc}},0)}\right] \exp(-t/\tau) \tag{S86}$$

to the temporal decays of the normalized absorbance at 383 nm in order to retrieve the rate constants of the photoconversion upon exciting at various wavelengths (Figure S42b). Equipped with the rate constants of the photoconversion and the light intensities (retrieved from the light powers) measured at all the excitation wavelengths, and the dependence of the

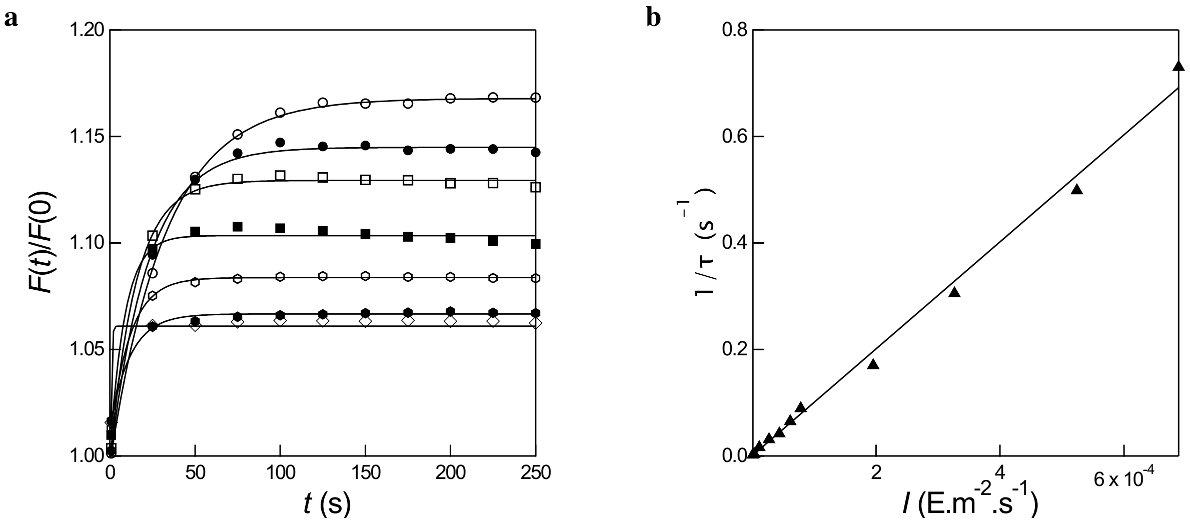

Figure S41: *Determination of the cross section for the photoconversion of α-(4-diethylamino)phenyl)-N-phenylnitrone (**Nit**) into N-(p-dimethylaminophenyl)formanilide in ethanol at 405 nm.* **a**: Time evolution of the normalized fluorescence emission at 574 nm from a 14 μM **Nit** and 1.0 μM **RhB** in spectroscopy grade ethanol contained in a 1×1 cm^2 quartz cuvette upon irradiation at constant light intensity at 405 nm at various light intensities (in 10^{-5} E.m^{-2}.s^{-1}): 2.7 (circles), 4.4 (disks), 6.2 (empty squares), 7.9 (filled squares), 33 (empty hexagons), 52 (diamonds), and 69 (filled hexagons). Markers: Experimental data; Solid lines: Monoexponential fit with Eq.(S1); **b**: Dependence of the inverse of the characteristic time τ retrieved in **a** from the monoexponential fit on the light intensity. Markers: Experimental data; Solid line: Linear fit. The extracted slope is 1005 ± 30 m^2.mol^{-1}.

molar absorption coefficient of **Nit** on the wavelength, we used Eq.(S23) to extract the dependence of the quantum yield of the **Nit** photoconversion on the excitation wavelength upon fixing the value of the quantum yield of the **Nit** photoconversion to 0.14 at 405 nm. The results are displayed in Table S8. The 0.15 value measured at 365 nm is in fair agreement with the 0.16 reported value at the same wavelength.[22] Moreover, the quantum yield of the **Nit** photoconversion is essentially constant over the whole range of investigated wavelengths.

Rise of the RhB fluorescence under illumination Figure S43 displays the time dependence of the fluorescence emission of 5.8 μM **Nit** and 1.0 μM **RhB** in spectroscopy grade ethanol upon irradiation at constant light intensity at 365, 380, 400, and 420 nm, which have been measured from recording the light power transmitted through the 1 cm light path quartz cuvette. Then we applied the monoexponential fitting function given in Eq.(S1) to the temporal rises of the normalized fluorescence at 574 nm in order to retrieve the rate constants of the photoconversion upon exciting at various wavelengths (Figure S43). Equipped with the rate constants of the photoconversion and the light intensities (retrieved from the light powers) measured at all the excitation wavelengths, and the dependence of the molar absorption coefficient of **Nit** on the wavelength, we used Eq.(S23) to extract the dependence of the quantum yield of the **Nit** photoconversion on the excitation wavelength upon fixing the value of the quantum yield of the **Nit** photoconversion to 0.14 at 405 nm. The results are displayed in Table S8. The 0.19 value measured at 365 nm is in fair agreement with the 0.16 reported value at the same wavelength.[22] Moreover, the quantum yield of the **Nit** photoconversion is again essentially constant over the whole range of investigated wavelengths.

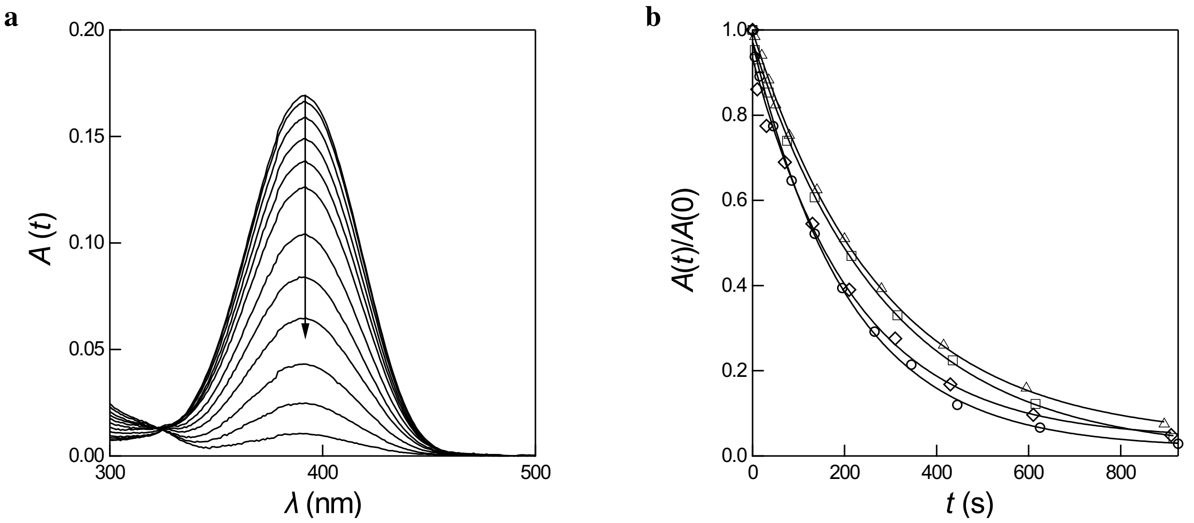

Figure S42: *Dependence of the quantum yield of the **Nit** photoconversion on the excitation wavelength from analyzing the decay of the **Nit** absorbance under illumination.* **a**: Time dependence of the absorption spectrum of 5.8 μM **Nit** in spectroscopy grade ethanol through the 1 cm light path quartz cuvette upon irradiation at constant light intensity at 365 nm. t(s): 0, 5, 20, 35, 50, 80, 140, 200, 280, 415, 595, and 895; **b**: Time dependence of the normalized absorbance at 383 nm of 5.8 μM α-(4-Diethylamino)phenyl)-N-phenylnitrone (**Nit**) in spectroscopy grade ethanol upon illumination at constant light intensity at 365 (diamonds), 380 nm (disks), 400 (diamonds) and 430 (squares) nm. Markers: Experimental data; solid lines: Monoexponential fit with Eq.(S86). $T = 293$ K

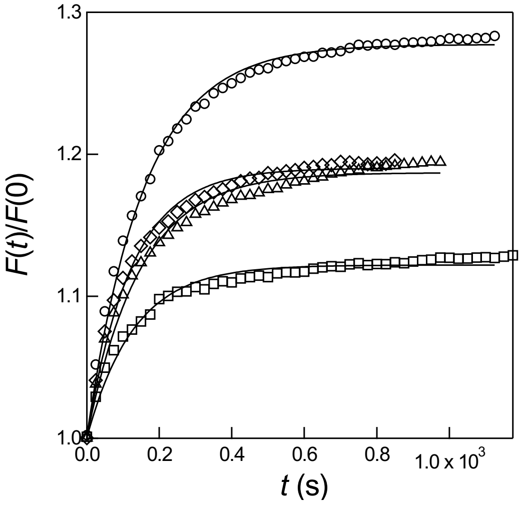

Figure S43: *Dependence of the quantum yield of the **Nit** photoconversion on the excitation wavelength from analyzing the rise of the **RhB** fluorescence under illumination.* Time dependence of the normalized fluorescence emission at 574 nm of a 5.8 μM **Nit** and 1μM **RhB** solution in spectroscopy grade ethanol through the 1 cm light path quartz cuvette upon illumination at constant light intensity at 365 (triangles), 380 nm (circles), 400 (diamonds) and 420 (squares) nm. Markers: Experimental data; solid lines: Monoexponential fit with Eq.(S86). $T = 293$ K.

Estimate of the achievable measurement uncertainty on light intensity I Considering the uncertainty in the parameters (σ and τ), we estimate that the achievable measurement uncertainty on light intensity I is 20% by using **Nit**.

Table S8: Photoconversion parameters of α-(4-diethylamino)phenyl)-N-phenylnitrone (**Nit**) in ethanol. The quantum yields of photoconversion, φ_A and φ_F have been respectively extracted from analyzing the decay of the absorbance at 383 nm of a 5.8 μM **Nit** solution in ethanol, and the rise of the fluorescence emission at 574 nm of a 5.8 μM **Nit** and 1μM **RhB** solution in ethanol under monochromatic illumination at 365, 380, 400, and 420 nm as representatives of 320 nm – 430 nm range. φ is the mean value of φ_A and φ_F. The molar absorption coefficients $\varepsilon(\lambda_{\rm exc})$ were extracted from the **Nit** absorption spectrum by using the Beer-Lambert law. The cross section $\sigma(\lambda_{\rm exc})$ was evaluated with Eq.(S23). $I_{\rm sup}(\lambda_{\rm exc})$ indicates the upper light intensity tested and relevant for a reliable measurement .

$\lambda_{\rm exc}$ (nm)	$10^4 \times \varepsilon(\lambda_{\rm exc})$ ($\pm$0.1; M^{-1}cm^{-1})	φ_F ($\pm$0.02)	φ_A ($\pm$0.02)	φ ($\pm$0.02)	$\sigma(\lambda_{\rm exc})$ ($\pm$10%; m^{2}mol^{-1})	$I_{\rm sup}(\lambda_{\rm exc})$ (E.m^{-2}.s^{-1}(W.m^{-2}))
365	2.2	0.24	0.15	0.19	960	11 10^{-4}(360)^a
380	3.4	0.13	0.18	0.15	1200	7.2 10^{-4}(230)^a
400	3.5	0.14	0.14	0.14	1100	7.0 10^{-4}(200)^a
420	2.0	0.20	0.20	0.20	850	12 10^{-4}(340)^a

^{a} Extracted from the value of $I_{\rm sup}(\lambda_{\rm exc})$ by using $I_{\rm sup}(\lambda_{\rm exc}) = I_{\rm sup}(\lambda_{\rm exc,ref}) \times \frac{\varepsilon(\lambda_{\rm exc,ref})}{\varepsilon(\lambda_{\rm exc})}$ where $\lambda_{\rm exc,ref}$ designates the wavelength at which the extraction of the cross section of photoconversion has been performed ($\lambda_{\rm exc,ref} = 405$ nm for **Nit**).

7.6 DDAO for transferring information on light intensity from a wavelength to another

One current operation on an optical setup is to transfer available information on light intensity from a wavelength to another. This is especially attractive in the orange and red wavelength range, where fluorescent actinometers are scarce and often exhibit a poor quantum yield of fluorescence. **DDAO** [7-hydroxy-9H-(1,3-dichloro-9,9-dimethylacridin-2-one)][23,24] is here an attractive fluorophore for transferring information on light intensity from a wavelength to another along the principle exposed in the Main Text. It is commercially available and exhibits a broad absorption band essentially covering the whole visible wavelength range after ionization of its phenol group obtained at pH$\geq$6.[24]

7.6.1 Preparation of the DDAO solutions

In a first step, we produced a 52 mM **DDAO** solution in DMSO by dissolving 0.8 mg of DDAO in 55 μg of spectrograde DMSO.[24] This mother solution was then diluted in an aqueous HEPES pH 7.9 buffer (100 mM NaCl, 5 mM NaOH, 10 mM HEPES) to reach 9 μM (see Figure S44).

7.6.2 Absorption and fluorescence emission spectra of DDAO

DDAO exhibits a broad absorption band between 450 and 650 nm and strongly emits fluorescence between 640 and 700 nm in HEPES pH 7.4 buffer. Table S9 reports on the dependence of the molar absorption coefficient of **DDAO** on the wavelength.

7.6.3 Validation of DDAO as light intensity-transferring fluorophore

We first checked that the quantum yield of fluorescence of **DDAO** did not depend on the excitation wavelength in its absorption band in the visible wavelength range as evidenced by the similarity of its absorption and normalized fluorescence excitation spectra (see Figure S2b). Hence, **DDAO** was shown to obey the Kasha's rule, which enables us to exploit

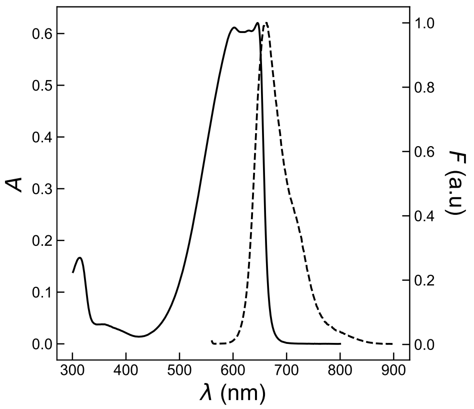

Figure S44: Absorption (solid line) and emission (dotted line; $\lambda_{\mathrm{exc}} = 600$ nm) spectra of 9 μM **DDAO** in an aqueous pH 7.9 HEPES buffer (100 mM NaCl, 5 mM NaOH, 10 mM HEPES) recorded in a 1×1 cm^2 quartz cuvette. $T = 293$ K.

Table S9: Dependence of the molar absorption coefficient $\varepsilon(\lambda_{\mathrm{exc}})$ of **DDAO** on the wavelength. Solvent: Aqueous HEPES pH 7.9 buffer (100 mM NaCl, 5 mM NaOH, 10 mM HEPES).

λ_{exc} (nm)	$10^{-4} \times \varepsilon(\lambda_{\mathrm{exc}})$ ($M^{-1}cm^{-1}$)
450	0.09 ± 0.01
470	0.18 ± 0.02
490	0.34 ± 0.04
510	0.60 ± 0.08
530	0.98 ± 0.13
550	1.44 ± 0.19
570	1.89 ± 0.24
590	2.24 ± 0.29
610	2.31 ± 0.30
630	2.31 ± 0.30
650	2.27 ± 0.30
670	0.21 ± 0.03

Eq.(S70) to transfer information on light intensity from one wavelength to another.

Then two series of experiments have been further performed in different regimes of light intensity to validate the relevance of **DDAO** as light intensity-transferring fluorophore.

Regime of low light intensity This series of experiments has been performed on the epifluorescence microscope by measuring the dependence of the **DDAO** fluorescence collected by the camera with a 775/140 band-pass filter on the light intensity from LEDs at 405±7 nm, 470±10 nm, 550±6 nm, and 630±9 nm evaluated by the current applied to feed the LEDs using the LED driver (DC4104, Thorlabs, NJ).

We first used the LED emitting at 405±7 nm (Figure S45a). For each applied voltage, we

- linked the **DDAO** fluorescence intensity to the light intensity, which was measured with the **Dronpa-2** actinometer;
- further measured the light intensity with the powermeter by using the protocol reported in subsection 7.3.1.

Then we turned to the LEDs emitting at 470±10 nm (Figure S45b), 550±6 nm (Figure S45c), and 630±9 nm (Figure S45d). For each applied voltage, we

- measured the **DDAO** fluorescence intensity and used Eq.(S70) to retrieve the light intensity;
- further measured the light intensity with the powermeter and an actinometer (**Dronpa-2** at 470±10 nm and **DASA** at 630±9 nm).

From examining Figures S45a–d, we concluded that:

- the light intensity linearly depends on the applied voltage for all the investigated LEDs;
- the **DDAO** fluorescence intensity provides consistent values of the light intensity with the measurements made with the fluorescent actinometers and the powermeter thereby validating its relevance.

Regime of high light intensity In a second series of experiments, we established the dependence of the fluorescence intensity at 650 nm from a 10 nM **DDAO** solution in 50 mM CHES buffer pH 9.3 on the light intensity of the focused 488 nm laser of our photoswitchometer,[7] which was measured with the powermeter.

In a first step, we measured the beam waist of the focused 488 nm laser by Fluorescence Correlation Spectroscopy (FCS). The time dependence of the autocorrelation function extracted from a 3.4 nM Fluorescein solution in 50 mM CHES buffer pH 9.3 is displayed in Figure S46a. It was fitted with the theoretical expression of the autocorrelation function involving 2D diffusion and the formation of the triplet state given in Eq.(S87) in which τ_D, τ_T, and A_T designate the diffusion time through the beam waist, the relaxation time of the fluorophore triplet state, and an amplitude fitting parameter.

$$G(\tau) = G(0) \times \left(1 + \frac{\tau}{\tau_D}\right)^{-1} \times \left[1 + A_T \exp\left(-\frac{\tau}{\tau_T}\right)\right] \tag{S87}$$

By using $D_{\mathrm{Fluo}} = 4.25\ 10^{-10}$ m^2.s^{-1} for the value of the diffusion coefficient of Fluorescein in water at 298 K,[25] we extracted the lateral extension of the beam waist $\omega_{\mathrm{xy}} = 2\left(D_{\mathrm{Fluo}}\tau_D\right)^{0.5} = 0.26\ \mu$m from the $\tau_D = 40\ \mu$s value retrieved from the fit. Equipped with the ω_{xy} value, we recorded the time dependence of the autocorrelation function extracted from a 10 nM DDAO solution in 50 mM CHES buffer pH 9.3 (see Figure S46b). No significant formation of a triplet state could be observed and we fitted experimental data with Eq.(S88).

$$G(\tau) = G(0) \times \left(1 + \frac{\tau}{\tau_D}\right)^{-1} \tag{S88}$$

From the retrieved diffusion time $\tau_D = 29\ \mu$s value and the knowledge of $\omega_{\mathrm{xy}} = 0.26\ \mu$m, we extracted $D_{\mathrm{DDAO}} = 5.8$ 10^{-10} m^2.s^{-1} for the value of the diffusion coefficient of **DDAO** in water at 293 K.

Equipped with the surface of the laser beam at the beam waist, we could investigate the dependence of the **DDAO** fluorescence signal on the light intensity of the laser measured with the powermeter. As displayed in Figure S47, the

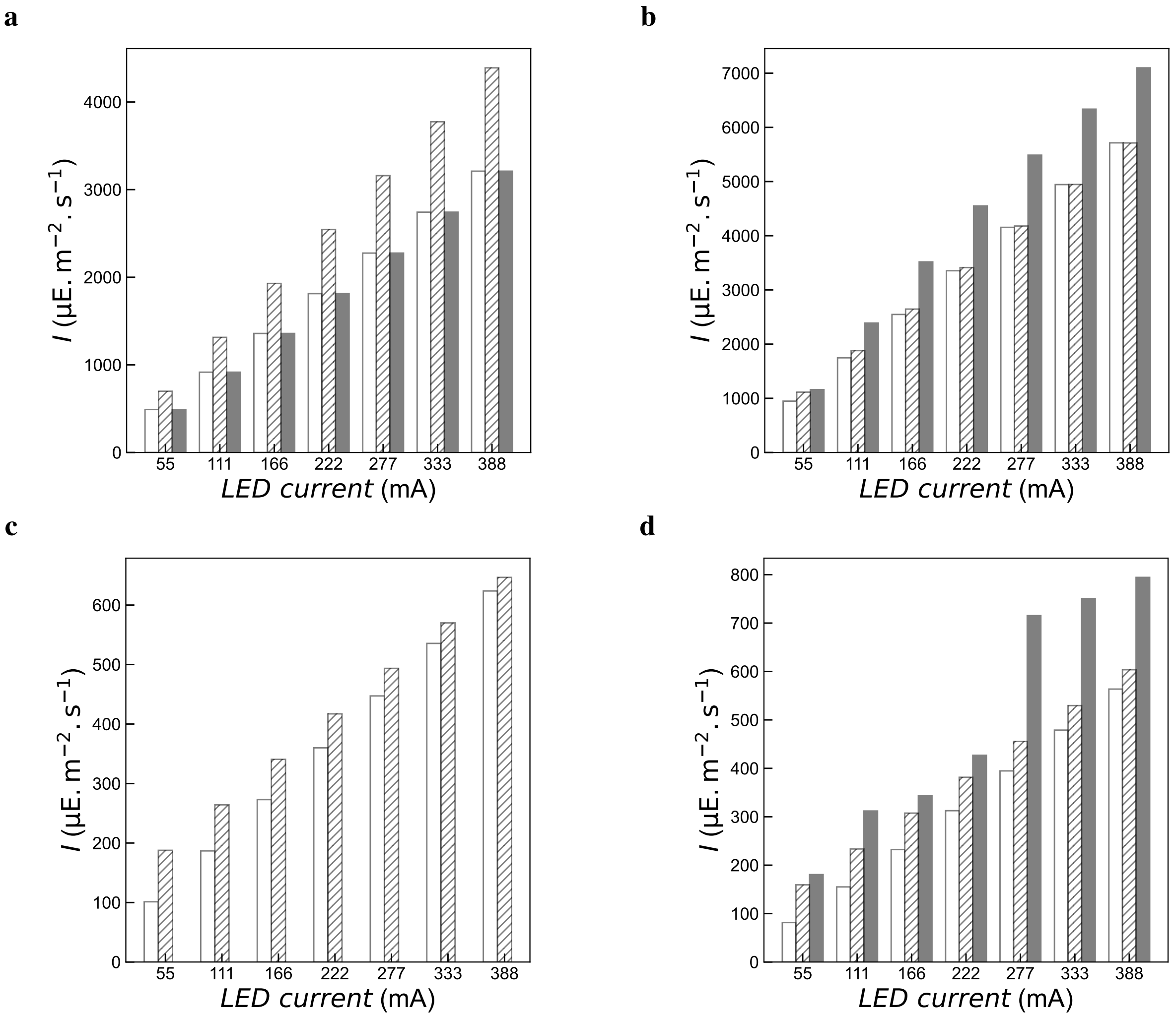

Figure S45: *Validation of* ***DDAO*** *as light intensity-transferring fluorophore in a regime of low light intensity*. Dependence of the light intensity from LEDs at 405±7 nm (**a**), 470±10 nm (**b**), 550±6 nm (**c**), and 630±9 nm (**d**) on the voltage applied to feed the LEDs as retrieved from using the **DDAO** fluorescence intensity (**a**–**d**, white bar), actinometers (**Dronpa-2** in **a** and **b**, **DASA** in **d**, black bar), and the powermeter (**a**–**d**, striped bar), using the protocol described in subsection 7.3.1. $T = 293$ K.

observed dependence is linear up to 1500 kW.cm^{-2} (6 10^4 E.m^{-2}.s^{-1}) and then saturates. Such a threshold is higher than the maximal light flux generated on a regular confocal microscope.

As a conclusion, **DDAO** is validated as light intensity-transferring fluorophore for most current microscopy setups with one-photon excitation.

Estimate of the achievable measurement uncertainty on light intensity I Considering the uncertainty in the parameters (σ and τ) exploited to acquire light intensity at a reference wavelength by using a fluorescent actinometer, we estimate that the achievable measurement uncertainty on light intensity I is 20% by using **DDAO**.

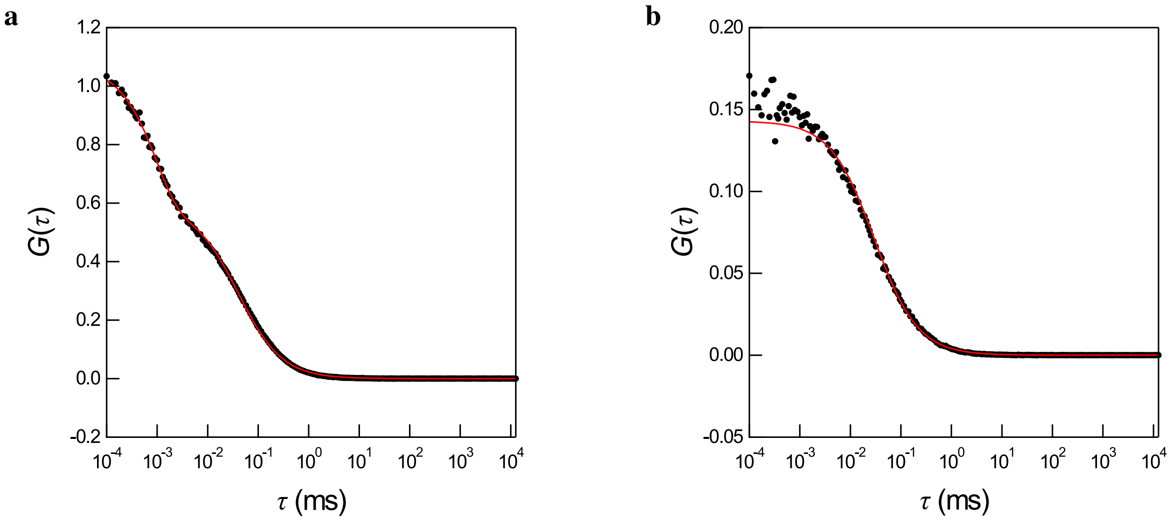

Figure S46: *FCS autocorrelation curves recorded at 293 K from 3.4 nM Fluorescein (**a**) and 10 nM **DDAO** (**b**).* Markers: Experimental data; Lines: Fits according to Eqs.(S87,S88) for Fluorescein and **DDAO** respectively, which yield $G(0) = 0.59$, $\tau_D = 40$ μs, $\tau_T = 1.0$ μs, and $A_T = 0.8$ for Fluorescein, and $G(0) = 0.14$ and $\tau_D = 29$ μs for **DDAO**. Solvent: 50 mM CHES buffer pH 9.3. $T = 293$ K.

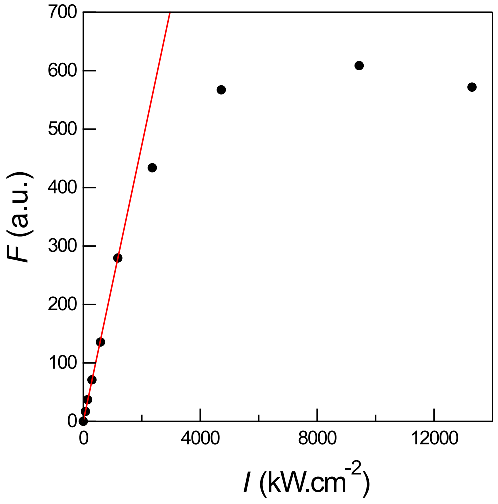

Figure S47: *Validation of **DDAO** as light intensity-transferring fluorophore in a regime of high light intensity*. Dependence of the fluorescence intensity at 650 nm from a 10 nM **DDAO** solution in 50 mM CHES buffer pH 9.3 on the light intensity of the 488 nm laser, which was measured with the powermeter. Markers: Experimental data; solid line: Linear fit. $T = 293$ K.

8 Supplementary Note 8: Validation of the extraction of the light intensity in fluorescence imaging

8.1 Fluorescence macroimager

The first protocol for light intensity measurement was validated in wide-field imaging by applying it in the case of a macroscope setup described in subsection 7.3.2 whose illumination comes in from an angle, causing an intensity gradient

at the sample, and comparing the obtained results to ones obtained through an optical simulation combined with a power meter reading.

A model of the illumination system was created and run using the optical design software package Optic Studio 18.9 (Zemax LLC, Kirkland, WA, US). The file corresponding to this is provided in Figure S48. A 3D view of the model within the software is shown at the top of Figure S48, while a CAD model, created in Rhinocerous 3D, is provided at the bottom of the same figure – to provide information on the relative spacing between the components. The corresponding Optic Studio (or Zemax) file is additionally provided (Macroscope/MACRO_Zemax.zmx). The optical components modelled are detailed in the subsection 7.3.2. In this case however, an excitation filter was not implemented, and instead the source wavelength was set to the appropriate wavelength of 470 nm. The LED was modelled using the "Source Radial" object, whereby the radial intensity profile was entered according to data provided by the manufacturer. An aperture was placed at the position of the achromatic doublets in order to prevent stray light, passing over the lenses, from reaching the detector placed at the sample plane. Its aperture was set to the diameter of the lenses. The sample plane was set at the appropriate angle according to the angle of illumination axis relative to the imaging axis.

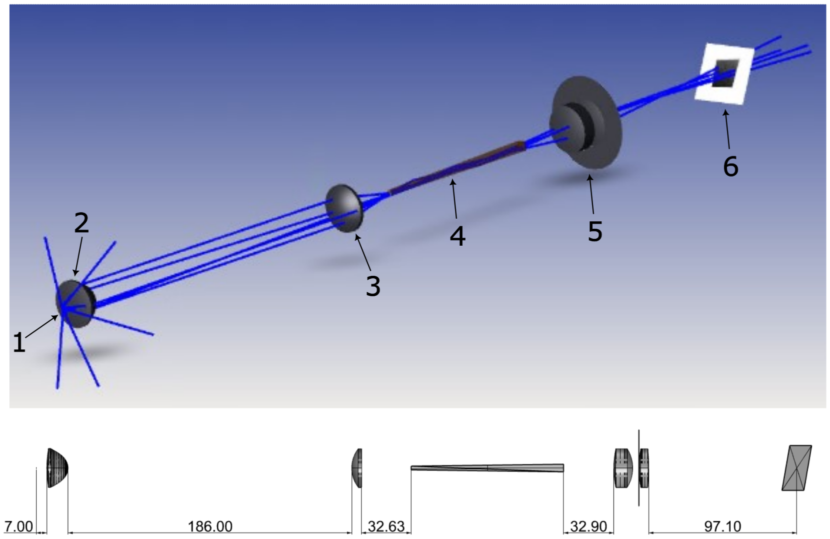

Figure S48: *3D view of the optical model of the fluorescence macroimager within the Optic Studio 18.9 software*. 1 – LED, 2 – Condenser, 3 – Plano-Convex Lens, 4 – Lightpipe, 5 – Matched Achromatic Doublet Pair and Aperture, 6 – Sample.

The simulation was run with 50,000,000 rays with the settings "Use Polarization" and "Split NSC Rays" selected. Following this, the incoherent irradiance data, provided (Macroscope/MACRO_SimulationData.npy), was saved for further processing. A jupyter python notebook, containing the code which carries out the calculations described in the remainder of this section, is provided (Macroscope/MACRO_Code.ipynb). The simulation provided the irradiance information in units of W.cm^{-2}. This was converted to units of μE.m^{-2}.s^{-1}, and the resulting image scaled and cropped such that the image was of the same size, and the relevant features in the same location, compared with the images obtained in the physical experiment. The power value attributed to the LED in the simulation was set to be a reasonable, but not the

exact, value. As such, the homogeneity information is accurate, but the overall power still required scaling. In order to do so, the power values from the simulation were integrated over the area of the illumination, to first obtain the overall power (P_{Total}):

$$P_{\mathrm{Total}} = \sum_{1}^{x}\sum_{1}^{y} I(x,y) \times A_{\mathrm{pixel}} \tag{S89}$$

where $I(x,y)$ is the intensity within a pixel with the image coordinates (x,y), and A_{pixel} the area that one pixel corresponds to at the sample plane. The scale factor between the overall power calculated from the simulation, and that obtained using a powermeter according to section 7.2.3 with a squared sensor (S170C, Thorlabs Inc., Newton, NJ, US) with a neutral density filter (to expand the sensor's maximum), was determined and applied to the intensity values of the simulated intensity image. This operation provided a corresponding simulated intensity image, which could be directly compared with the experimentally obtained intensity image.

As a way to compare the results, the angle of the light gradient direction was determined for both the simulated and experimentally obtained image. In order to do so, data were extracted along a straight line within the illuminated area, and a line fit made on the intensity data along such line, providing gradient information. This was carried out repeatedly on the image at different rotational positions. The rotation angle corresponding to the highest gradient corresponded to the direction of the gradient. The angle between the directions found for the computed and experimental gradients is $3.2°$, which accounts for a fair agreement between the data and the simulation.

8.2 Confocal microscopes

8.2.1 Theory

The measurement of light intensity in confocal microscopy exhibits several differences with respect to the measurement in wide-field microscopy:

- Illumination is not homogeneous over the field of view as in epifluorescence microscopy. Introducing the cartesian coordinates (x,y,z), we consider that illumination results from a focused laser beam assumed to adopt a radially and axially Gaussian intensity profile given in Eq.(S90)

$$I(x,y,z) = I_0 \exp\left[-2\left(\frac{x^2+y^2}{w_0^2}\right)\right]\exp\left[-2\left(\frac{z^2}{z_0^2}\right)\right] \tag{S90}$$

  where I_0, w_0, and z_0 designate the light intensity at coordinates $(0,0,0)$, the waist radius in the focal plane, and the radial resolution z_0 respectively (see Figure S49).[26]

- In order to build an image, the focus of the laser scans the sample according to a chosen pixel size Δy, as displayed in Figure S50.

[26]As a consequence, Eq. S90 is valid only for lenses of relatively low NA (which have a cylindrical illumination profile).

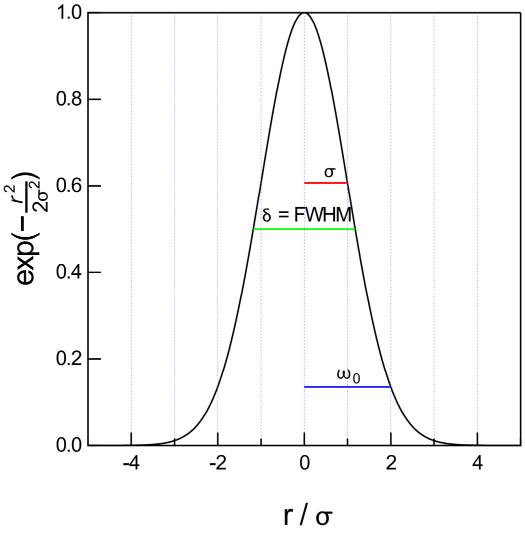

Figure S49: *Cross-section of the gaussian beam for* $z = z_0$. $\omega_0 = 2\sigma$ and I_0 corresponds to the maximum.

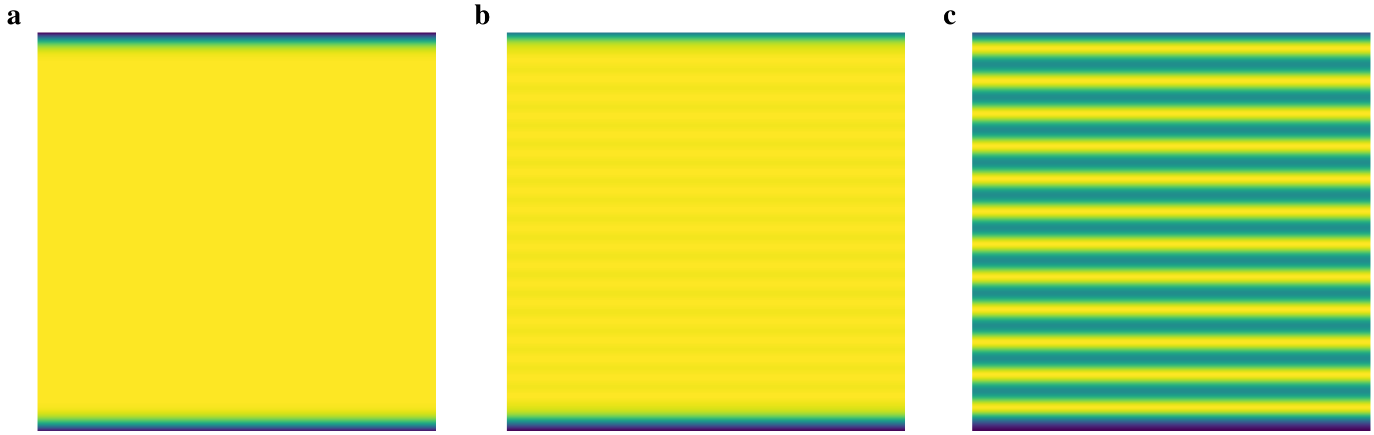

Figure S50: *Simulation of the integrated light path of the laser for different values of the pixel size* Δy. The simulations were performed by convolving a Gaussian kernel representing the beam of the laser with horizontal lines representing the path of the laser. $\omega_0 = 0.3$ and $\Delta y = 0.08$, 0.29 and 0.5 in **a**, **b** and **c** respectively.

In relation with estimating the light intensity in confocal microscopy, we are interested in the time evolution of the overall fluorescence signal arising from the molecules of fluorescent actinometer, which are assumed to be spatially fixed. The concentration of fluorescent actinometer $C(x, y, z, t)$ is submitted to Eq.(S91)

$$\frac{\partial C\left(x,y,z,t\right)}{\partial t} = -\sigma I\left(x\left(t\right), y\left(t\right), z\left(t\right)\right) C\left(x,y,z,t\right) \tag{S91}$$

by assuming the photoconversion to be irreversible.[27]

Photoconversion along a line Following Braeckmans et al,[26,27] we first consider the laser beam to scan along a single line expressed by $y = Y$ with a constant scanning speed v and take the x-axis to be parallel with the scanning direction.

[27]When photoconversion is reversible, Eq.(S91) reports on the distance of the concentration $C\left(x,y,z,t\right)$ to its steady-state.

We first estimate the overall fluorescence signal arising from the molecules of fluorescent actinometer when the scan of the line segment has been completed.

Due to scanning, the x-coordinate of the light intensity profile is a function of time: $I\left(x - x\left(t\right), y - Y, z\right)$. We consider scanning to start at $x = -\ell/2$ and end up at $x = \ell/2$ with $x\left(t\right) = vt - \ell/2$ and T to be the time it takes for the scanning beam to cross the line with length ℓ at constant scanning speed $v = \ell/T$. We denote $C_0\left(x, y, z\right)$ the concentration of fluorescent actinometer before starting to record the first frame. Then the fluorescence signal $F_{1,1}\left(x, y, z\right)$ at position (x, y, z) after time T is given in Eq.(S92)

$$F_{1,1}\left(x, y, z, T\right) = C_0\left(x, y, z\right) e^{\left[-\sigma \int_0^T L(x(t),Y,z) I(x-x(t),y-Y,z)dt\right]} \times \int_0^T L\left(x\left(t\right), Y, z\right) I\left(x - x\left(t\right), y - Y, z\right) dt \quad \text{(S92)}$$

where $L\left(x\left(t\right), Y, z\right)$ is the function that describes the modulation of the light intensity and has value 0 when the laser is switched off and 1 when the laser is turned on.

Expressing t in terms of $x\left(t\right)$ and renaming $x\left(t\right)$ as x', Eq.(S92) yields Eq.(S93)

$$F_{1,1}\left(x, y, z, T\right) \;=\; C_0\left(x, y, z\right) e^{\left[-\frac{\sigma}{v} \int_{-\ell/2}^{\ell/2} I(x-x',y-Y,z)dx'\right]} \times \int_{-\ell/2}^{\ell/2} I\left(x - x', y - Y, z\right) dx' \quad \text{(S93)}$$

which yields Eq.(S94)

$$\begin{aligned} F_{1,1}\left(x, y, z, T\right) = C_0\left(x, y, z\right) e^{-\sqrt{\frac{\pi}{8}}\frac{\sigma w_0}{v} I_0 e^{-2\frac{(y-Y)^2}{w_0^2}} e^{-2\frac{z^2}{z_0^2}} \left\{\mathrm{erf}\left[\frac{\sqrt{2}}{w_0}\left(x+\frac{\ell}{2}\right)\right] - \mathrm{erf}\left[\frac{\sqrt{2}}{w_0}\left(x-\frac{\ell}{2}\right)\right]\right\}} \\ \times \sqrt{\frac{\pi}{8}} \frac{w_0}{v} I_0 e^{-2\frac{(y-Y)^2}{w_0^2}} e^{-2\frac{z^2}{z_0^2}} \left\{\mathrm{erf}\left[\frac{\sqrt{2}}{w_0}\left(x + \frac{\ell}{2}\right)\right] - \mathrm{erf}\left[\frac{\sqrt{2}}{w_0}\left(x - \frac{\ell}{2}\right)\right]\right\} \end{aligned} \quad \text{(S94)}$$

after the variable change $X = \frac{\sqrt{2}}{w_0}\left(x - x'\right)$.

Provided that $\ell \gg w_0$ and $-\frac{\ell}{2} \ll x \ll \frac{\ell}{2}$, Eq.(S94) yields Eq.(S95)

$$F_{1,1}\left(x, y, z, T\right) = C_0\left(x, y, z\right) e^{-\sqrt{\frac{\pi}{2}}\frac{\sigma w_0}{v} I_0 e^{-2\frac{(y-Y)^2}{w_0^2}} e^{-2\frac{z^2}{z_0^2}}} \times \sqrt{\frac{\pi}{2}} \frac{w_0}{v} I_0 e^{-2\frac{(y-Y)^2}{w_0^2}} e^{-2\frac{z^2}{z_0^2}} \quad \text{(S95)}$$

Photoconversion of a square A 2D square geometry is subsequently photoconverted by scanning N line segments at regular interline spacing Δy. Following Braeckmans et al,[26,27] we consider the case associated to $\Delta y \leq w_0$. In such a situation, the molecules of fluorescent actinometer experience photoconversion during several scans and the fluorescence signal $F_{1,N}\left(x, y, z, T\right)$ obtained after scanning N lines is computed by integrating $F_{1,1}\left(x, y, z, T\right)$ over y from $y = -\ell/2$ to $y = \ell/2$. Hence, Eq.(S95) first yields Eq.(S96)

$$F_{1,N}\left(x, y, z, T\right) = C_0\left(x, y, z\right) e^{-\sqrt{\frac{\pi}{2}}\frac{\sigma w_0}{v\Delta y} I_0 e^{-2\frac{z^2}{z_0^2}} \int_{-\ell/2}^{\ell/2} e^{-2\frac{\left(y-y'\right)^2}{w_0^2}} dy'} \times \sqrt{\frac{\pi}{2}} \frac{w_0}{v\Delta y} I_0 e^{-2\frac{z^2}{z_0^2}} \int_{-\ell/2}^{\ell/2} e^{-2\frac{\left(y-y'\right)^2}{w_0^2}} dy', \quad \text{(S96)}$$

which gives Eq.(S97)

$$\begin{aligned} F_{1,N}\left(x, y, z, T\right) = C_0\left(x, y, z\right) e^{-\sqrt{\frac{\pi}{2}}\frac{\sigma w_0}{v\Delta y} I_0 e^{-2\frac{z^2}{z_0^2}} \int_{\frac{\sqrt{2}}{w_0}\left(y-\frac{\ell}{2}\right)}^{\frac{\sqrt{2}}{w_0}\left(y+\frac{\ell}{2}\right)} \frac{w_0}{\sqrt{2}} e^{-X^2} dX} \\ \times \sqrt{\frac{\pi}{2}} \frac{w_0}{v\Delta y} I_0 e^{-2\frac{z^2}{z_0^2}} \int_{\frac{\sqrt{2}}{w_0}\left(y-\frac{\ell}{2}\right)}^{\frac{\sqrt{2}}{w_0}\left(y+\frac{\ell}{2}\right)} \frac{w_0}{\sqrt{2}} e^{-X^2} dX \end{aligned} \quad \text{(S97)}$$

after the variable change $X = \frac{\sqrt{2}}{w_0}(y - y')$. Eq.(S97) eventually yields Eq.(S98)

$$F_{1,N}(x, y, z, T) = C_0(x, y, z)\, e^{-\sigma \frac{\pi w_0^2}{2v\Delta y} I_0 e^{-2\frac{z^2}{z_0^2}}} \times \frac{\pi}{2} \frac{w_0^2}{v\Delta y} I_0 e^{-2\frac{z^2}{z_0^2}} \tag{S98}$$

upon considering that $\ell \gg w_0$. In the limit of recovering the fluorescence signal from the focal plane only, one finally obtains Eq.(S99)

$$F_{1,N}(x, y, 0, T) = C_0(x, y, 0)\, e^{-\sigma \frac{\pi w_0^2}{2\Delta y^2} I_0 d} \times \frac{\pi w_0^2}{2\Delta y^2} I_0 d \tag{S99}$$

upon introducing the dwell time $d = \frac{\Delta y}{v}$.

Interestingly, $C_0(x, y, 0)\, e^{-\sigma \frac{\pi w_0^2}{2\Delta y^2} I_0 d}$ provides the final distribution of the concentration of the fluorescent actinometer after scanning N line segments to build the first frame, which acts as the initial distribution to build the second frame. Then, one reproduces the calculation above and it writes Eq.(S100)

$$F_{2,N}(x, y, 0, T) = C_1(x, y, 0)\, e^{-\sigma \frac{\pi w_0^2}{2\Delta y^2} I_0 d} = C_0(x, y, 0)\, e^{-\sigma \frac{\pi w_0^2}{2\Delta y^2} I_0 2d} \times \frac{\pi w_0^2}{2\Delta y^2} I_0 d \tag{S100}$$

After acquiring n frames, one eventually obtains Eq.(S101)

$$F_{n,N}(x, y, 0, T) = C_0(x, y, 0)\, e^{-\sigma \frac{\pi w_0^2}{2\Delta y^2} I_0 n d} \times \frac{\pi w_0^2}{2\Delta y^2} I_0 d = C_0(x, y, 0)\, I d e^{-\frac{nd}{\tau}} \tag{S101}$$

with

$$I = \frac{\pi w_0^2}{2\Delta y^2} I_0 \tag{S102}$$

and

$$\frac{1}{\tau} = \sigma I = \sigma \frac{\pi w_0^2}{2\Delta y^2} I_0, \tag{S103}$$

which designate the integral light intensity at the laser focus divided by the pixel surface, and the inverse of the characteristic time associated to the photoconversion of the fluorescent actinometer at light intensity I.

Hence, the extraction of the characteristic time τ from the decay of the fluorescence signal of the fluorescent actinometer on the number of frames multiplied by the dwell time (which amounts to a time t) provides an average of the light intensity I experienced by the sample during image acquisition.

8.2.2 Confocal microscopy equipped with a continuous laser

Measurement of the radial waist of the laser beam To validate the relevance of the evaluation of the light intensity by using the **Dronpa-2** actinometer, we first implemented Raster image correlation spectroscopy (RICS) on a suspension of Fluorescein-labeled 0.04 μm beads (FluoSpheres F8795, Thermo Fischer Scientific, MA) for determining the radial waist of the laser beam of the confocal microscope ω_0.[28,29]

In a first step, we measured the hydrodynamic radius of the fluorescent beads by FCS. We measured the beam waist of the focused 488 nm laser by recording the time dependence of the autocorrelation function extracted from a 10 nM Fluorescein solution in 50 mM CHES pH 9.3 (Figure S51a). It was fitted with the theoretical expression of the autocorrelation function involving 2D diffusion and the formation of the triplet state given in Eq. (S87). By using $D_{\mathrm{Fluo}} = 4.25\ 10^{-10}$ m^2.s^{-1} for the value of the diffusion coefficient of Fluorescein in water at 298 K,[25] we extracted the lateral extension of the beam waist $\omega_0 = 2\left(D_{\mathrm{Fluo}}\tau_D\right)^{0.5} = 0.28$ μm from the $\tau_D = 45$ μs value retrieved from the fit. We recorded the time dependence of the autocorrelation function extracted from a suspension of Fluorescein-labeled beads in 50 mM CHES pH 9.3 (see Figure S51b) and we fitted experimental data with Eq.(S88). From the retrieved diffusion time $\tau_D = 4.4$ ms value and the knowledge of $\omega_0 = 0.28$ μm, we extracted $D_{\mathrm{beads}} = 4.25\ 10^{-12}$ m^2.s^{-1} for the value of the diffusion coefficient of the Fluorescein-labeled beads in water at 293 K.

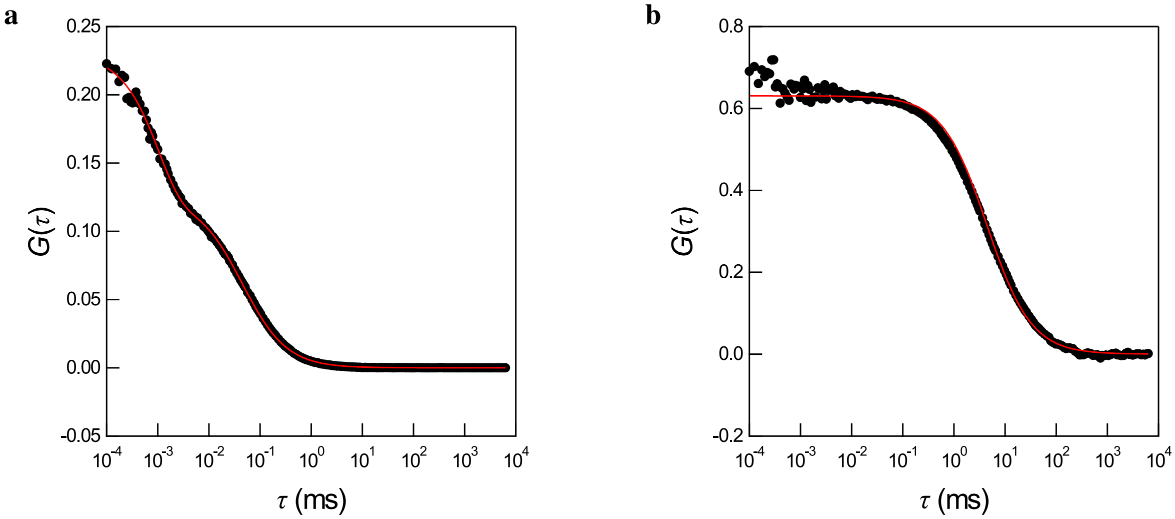

Figure S51: *FCS autocorrelation curves recorded at 293 K from 10 nM Fluorescein (**a**) and 3 nM Fluorospheres beads (**b**).* Markers: Experimental data; Lines: Fits according to Eqs.(S87,S88) for Fluorescein and Fluorescein-labeled beads respectively, which yield $G(0) = 0.12$, $\tau_D = 44$ μs, $\tau_T = 1.0$ μs, and $A_T = 0.9$ for Fluorescein, and $G(0) = 0.63$ and $\tau_D = 4.4$ ms for the Fluorescein-labeled beads. Solvent: 50 mM CHES buffer pH 9.3. $T = 293$ K.

Equipped with the diffusion coefficient of the Fluorescein-labeled beads, a series of 30 images of their suspension at 3 nM concentration in 50 mM CHES pH 9.3 sandwiched between two glass-slides with a 80 μm-thick spacer was acquired over a 10.6×10.6 μm^2 square in the raster scanning mode (Objective 20$\times$, NA 0.5; 256×256 pixel^2; pixel size: 0.042 μm; dwell time τ_{dt}: 8.275 μs) with the focused 488 nm laser of the confocal microscope (5% light power associated to 1.76 μW measured in the focal plane of the objective with the powermeter). The data have been processed with the Globals software for Images SimFCS, which is kindly available from https://www.lfd.uci.edu/globals/ (Figure S52). Hence, we retrieved $\omega_0 = 0.44$ μm from analyzing the whole set of data.

Measurement of the light intensity with the fluorescent actinometer The theoretical analysis reported in subsection 8.2.1 established the dependence of the characteristic time τ associated to the photoconversion of the fluorescent acti-

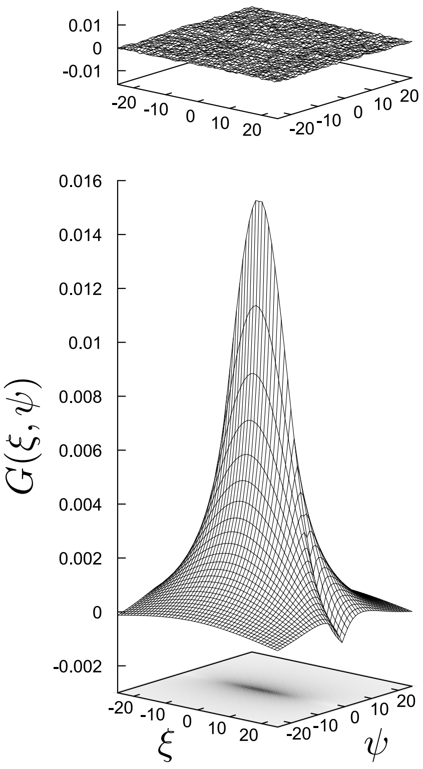

Figure S52: *Fit and difference of the correlation of the image of Fluorescein-labeled beads in water at 293 K obtained at 488 nm with the confocal microscope equipped with the continuous laser.* In the fit plotted in three dimensions, the height of the plot corresponds to the magnitude of the function (color code). The horizontal axis is the horizontal correlation shift, ξ. The vertical axis is the vertical correlation shift, ψ. The image correlation was calculated at a region of interest of 128 $\times$ 128 pixel^2 placed in the center of the original series of images and the correlation function was calculated for half the size of the region of interest in the original image (64$\times$64 pixels^2).

nometer on the light intensity I experienced by the sample during image acquisition and the tabulated cross section σ of the actinometer photoconversion. In a first step, we validated the relevance of the corresponding Eq.(S103) by plotting the dependence of $1/\tau$ on the square of the pixel size Δy^2.

As shown in Figure S53, τ linearly depends on the square of the pixel size Δy^2 up to ω_0^2 as anticipated from Eq.(S103). Hence, the measurement of the characteristic time τ together with the tabulated cross section σ of the actinometer photoconversion make possible to extract I experienced by the sample during image acquisition, and subsequently $I_0 = \frac{2\Delta y^2}{\sigma \pi w_0^2 \tau}$ from the knowledge of w_0 and Δy.

Measurement of the light intensity with the powermeter At the same time, a powermeter spatially integrates light intensity over all its detecting element and delivers $\frac{1}{2} I_0 \pi w_0^2$ (obtained by 2D integration of the Gaussian beam). With the present confocal microscope, scanning is performed at constant speed along the x-axis and light application effectively occurs only during a fraction γ of the period of the sinusoidal motion of the focal point.[28] Hence, the $power$ experienced

[28] To evaluate the fraction γ, the sensitive element of a fast photodiode (PD10A, Thorlabs, NJ) was placed at the focal plane of the imaging objective during acquisition of a series of 30 images. The output signal from the photodiode was digitized by a 300 MHz oscilloscope (RTB2004, Rhode and Schwarz, Munich, Germany) and we measured $\gamma = 0.4$, indicating that the laser excitation is turned off when the beam returns to the initial x position between line scans to reduce photo-damage to the sample.

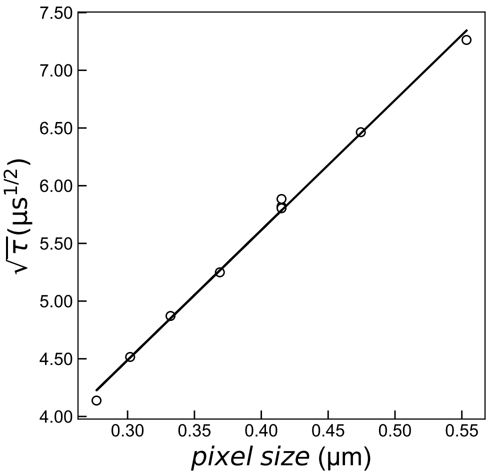

Figure S53: *Dependence of the square root of the characteristic time τ retrieved from the decay of the fluorescence signal of **Dronpa-2** on the pixel size Δy.* The dependence has been established by averaging the fluorescence signal over segmented 256×256 images of **Dronpa-2**-labeled fixed U-2 OS cells scanned with the confocal microscope equipped with a continuous laser with 8.24 μs dwell time and 10 % laser power at 488 nm.

by the sensor during scanning is $P = \frac{\gamma}{2} I_0 \pi w_0^2$.

Equipped with the value of w_0 measured with Raster image correlation spectroscopy and the γ parameter, it subsequently becomes possible to retrieve the $I_0 = \frac{2}{\gamma \pi w_0^2} P$ value from the light power measured with the powermeter.

Conclusion We used the preceding results to evaluate the consistency of the measurement of the light intensity delivered by the focused laser beam at the focal plane of the confocal microscope at $\lambda = 488$ nm as retrieved with the **Dronpa-2** actinometer and with the powermeter:

- Exploiting the **Dronpa-2** actinometer in the nucleus of fixed cells, we extracted $\tau = 19$ μs for the characteristic time associated to the photoconversion at 10% light power measured in the scanning mode at 1.7 μs dwell time over a 128×128 pixels^2 image with 0.415 μm pixel size and $\omega_0 = 0.44$ μm. Using $\sigma = 198$ m^2.mol^{-1}, we retrieved $I_0 = 150 \pm 30$ E.m^{-2}.s^{-1};

- Exploiting the associated measurement of the powermeter in the focal plane of the objective (3.65 μW; 1.45 10^{-11} E.s^{-1}), the value of w_0 measured with Raster image correlation spectroscopy ($\omega_0 = 0.44$ μm), and the $\gamma = 0.4$ parameter, we extracted $I_0 = 120 \pm 25$ E.m^{-2}.s^{-1}.

The fair agreement between both derivations underlines the relevance of the fluorescent actinometers to estimate the light intensity in confocal microscopy.

8.2.3 Confocal microscopy equipped with a pulsed laser

Measurement of the radial waist of the laser beam

Measurement from Raster image correlation spectroscopy (RICS) To measure the radial waist of the laser beam, we first proceeded as reported above with the confocal microscope equipped with a continuous laser. We applied Raster image correlation spectroscopy (RICS) on a suspension of Fluorescein-labeled 0.04 μm beads (FluoSpheres F8795, Thermo Fischer Scientific, MA) for determining the radial waist of the laser beam of the confocal microscope ω_0.[28,29]

Equipped with the $D_{\mathrm{beads}} = 4.25\ 10^{-12}$ m^2.s^{-1} diffusion coefficient of the Fluorescein-labeled beads in water at 293 K, we acquired a series of 50 images of their suspension at 3 nM concentration in 50 mM CHES pH 9.3 sandwiched between two glass-slides with a 120 μm-thick spacer over a 6.05×6.05 μm^2 square in the raster scanning mode (Objective HC PL APO CS2 40×, NA 1.1; 256 × 256 pixel^2; pixel size: 0.024 μm; dwell time τ_{dt}: 4.9 μs) with the focused 488 nm laser of the confocal microscope. The data have been then processed with the Globals software for Images SimFCS, which is kindly available from https://www.lfd.uci.edu/globals/ (Figure S54). Hence, we retrieved $\omega_0 = 0.32$ μm from analyzing the whole set of data.

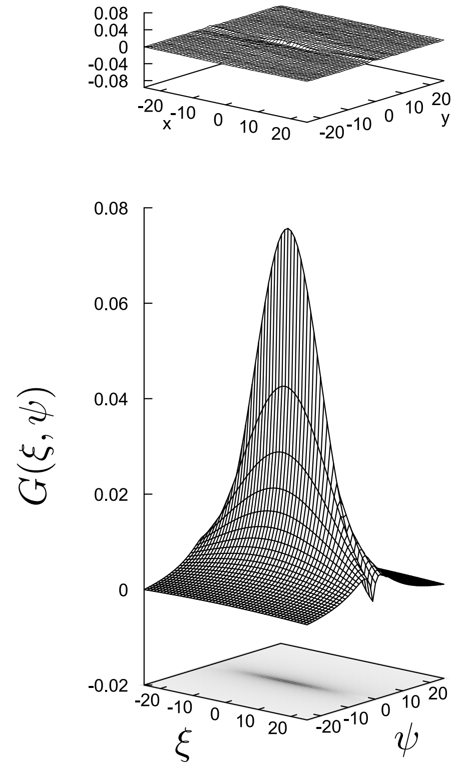

Figure S54: *Fit and difference of the correlation of the image of Fluorescein-labeled beads in water at 293 K obtained at 488 nm with the confocal microscope equipped with the pulsed laser.* In the fit plotted in three dimensions, the height of the plot corresponds to the magnitude of the function (color code). The horizontal axis is the horizontal correlation shift, ξ. The vertical axis is the vertical correlation shift, ψ. The image correlation was calculated at a region of interest of 128 × 128 pixel^2 placed at the center of the original series of images and the correlation function was half the size of the original image, 64×64 pixels^2.

Measurement from collecting the point spread function We also characterized the beam waist of the pulsed laser at 488 nm on the confocal microscope by recording a z-stack of (x,y) images of a 100 μm Green-Yellow-labeled bead (Fluospheres, ThermoFischer).[30] We both processed the images in 2D (by Gaussian fit of the image of the z-stack associated to the highest average value; Figure S55) and 3D (by using the software MetroloJ[31]) and extracted $\omega_0 = 0.28$ μm and $\omega_0 = 0.26$ μm respectively.

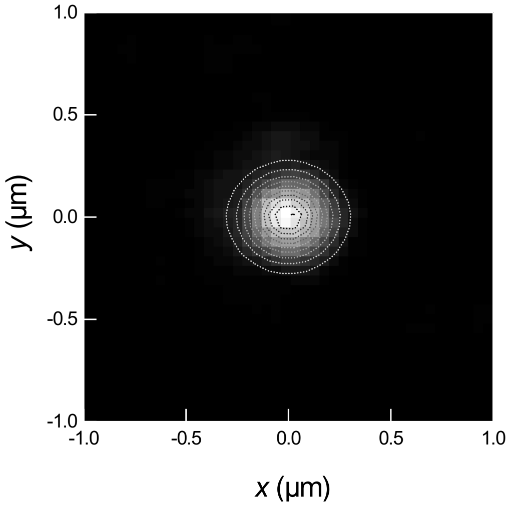

Figure S55: *Two-dimensional curve fitting of a 100 nm Green-Yellow-labeled polystyrene bead imaged with the confocal microscope equipped with the 488 nm pulsed laser.* Dotted contour lines from white to black correspond to increase in intensity by steps of 10% of the fitted maximum value.

Measurement of the light intensity with the fluorescent actinometer The principle of the validation reported above for the confocal microscope equipped with the continuous laser remains valid. Thus, we again evaluated the relevance of Eq.(S103) by plotting the dependence of $\sqrt{\tau}$ on the pixel size Δy.

As shown in Figure S56, τ linearly depends on the square of the pixel size Δy^2 up to ω_0^2 as anticipated from Eq.(S103). Hence, the measurement of τ together with the tabulated cross section σ of the actinometer photoconversion make possible to extract I experienced by the sample during image acquisition, and subsequently $I_0 = \frac{2\Delta y^2}{\sigma \pi w_0^2 \tau}$ from the knowledge of w_0 and Δy.

At that step, it is important to notice that the tabulated cross section σ of the **Dronpa-2** photoconversion reported in Table 1 of the Main Text refers to photoconversions, which have been investigated in a regime of light intensities not covering the ones presently reached with the pulsed laser at 488 nm.

Measurement of the light intensity with the powermeter The expressions derived above for the measurement of the light intensity with the powermeter are still applicable. Equipped with the value of w_0 measured with Raster image correlation spectroscopy and the γ parameter (equal to $\gamma = 1$ in the exploited bleaching mode), the I_0 value retrieved from the light power P measured with the powermeter is equal to $I_0 = \frac{2}{\gamma \pi w_0^2} P$.

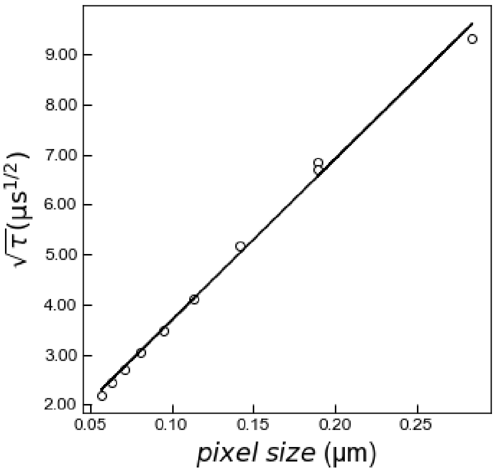

Figure S56: *Dependence of the square root of the characteristic time τ retrieved from the decay of the fluorescence signal of* ***Dronpa-2*** *on the pixel size Δy.* The dependence has been established by averaging the fluorescence signal over a segmented 512×512 images of **Dronpa-2**-labeled fixed U-2 OS cells scanned with the confocal microscope equipped with a pulsed laser with 1.2 μs dwell time and 0.2 % laser power at 488 nm.

Conclusion We used the preceding results to compare the measurements of the light intensity delivered by the focused laser beam at the focal plane of the confocal microscope at $\lambda = 488$ nm as retrieved with the **Dronpa-2** actinometer and with the powermeter from exploiting the value of the beam waist:

- Exploiting the **Dronpa-2** actinometer in the nucleus of fixed cells, we extracted $\tau = 1.2$ μs for the characteristic time associated to the photoconversion at 2% light power measured in the scanning mode at 1.2 μs dwell time over a 512×512 pixels^2 image with 0.114 μm pixel size and $\omega_0 = 0.3$ μm. Using $\sigma = 198$ m^2.mol^{-1}, we retrieved $I_0 = 390 \pm 80$ E.m^{-2}.s^{-1};
- Exploiting the associated measurement of the powermeter in the focal plane of the objective (17.3 μW; 6.9 10^{-11} E.s^{-1}), the value of w_0 measured with Raster image correlation spectroscopy ($\omega_0 = 0.3$ μm), and the $\gamma = 1$ parameter, we extracted $I_0 = 490 \pm 100$ E.m^{-2}.s^{-1}.

The fair agreement between both derivations suggests that using $\sigma = 198$ m^2.mol^{-1} is relevant for the measurement of the light intensity delivered by the focused laser beam at the focal plane of the confocal microscope.

9 Supplementary Note 9: Measurement of light intensity from a LED array

Here, we present a case demonstrating a limitation of the first protocol for measuring light intensity: retrieving 3D intensity distribution information in a non-homogeneous light intensity profile. In order to evaluate and demonstrate this limitation, an experiment was conducted whereby our protocol was applied to the situation of a Petri dish, containing a solution of the **Dronpa-2** fluorescent actinometer, being illuminated by a surrounding radial array of LEDs and being imaged from above; and the results compared to those obtained through an optical simulation.

9.1 Experimental Setup

An image and CAD rendered views of the setup, with the Petri dish and **Dronpa-2** solution sectioned, created in Rhinoceros 3D (Robert McNeel & Associates, Seattle, WA, US), of the setup are provided in Figure S57. The setup consists of an LED strip light (ZFS-85000HD-B, JKL Components Corporation, Los Angeles, CA, US), which contains 460 nm LEDs (L1SP-RYL0002800000, Lumileds, San Jose, CA, USA), stuck to the inside of a large 100 mm diameter plastic Petri dish (Corning Incorporated, Corning, NY, US), and a small 35 mm diameter plastic petri dish (Thermo Fisher Scientific, Waltham, MA, US), containing 1.6 mL of 4 μM **Dronpa-2** solution, placed inside it at its center. In order to have the solution at approximately the same height as the LEDs, a base was printed in black PLA/PHA plastic (ColorFabb B.V, Belfeld, Netherlands) on the Ultimaker 2+ Connect 3D printer (Ultimaker B.V., Utrecht, Netherlands), for the small Petri dish to sit on. In order to simplify the situation, and therefore the simulation, the bottom of the large Petri dish was covered with paper painted with highly absorbent black paint (Musou Black, Koyo Orient Japan Co., Ltd., Saitama, Japan) in order to eliminate any reflections, which could reach the **Dronpa-2** solution, meaning it could be ignored in the simulation (Figure S57). The CAD images do not show the large Petri dish for this reason. In Figure S57, the CAD models show the **Dronpa-2** solution in place, with a meniscus shape, based on the meniscus shape of water in a Petri dish as seen in reference.[32] A scale bar is provided for the final CAD model, which is a 2D frontal sectioned view, allowing the size and position of the different components to be recovered.

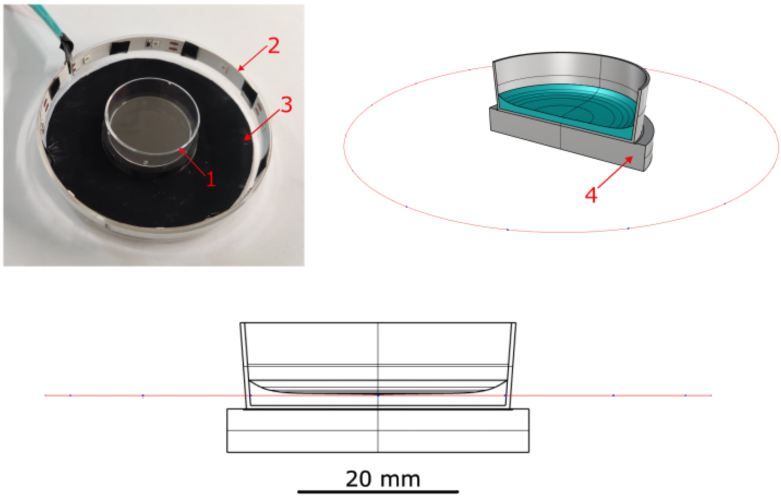

Figure S57: *Photo (top left), CAD perspective sectioned view (top right) and CAD front sectioned view (bottom) of the Petri dish LED array setup.* 1 – Small Petri Dish (where the **Dronpa-2** solution is held), 2 – LED Array (460 nm), 3 – Large Petri Dish with Blackened Bottom, 4 – 3D Printed Base. The large Petri dish is omitted in the CAD views since it is not implemented in the simulation. The 20 mm scale bar applies only to the bottom image. In the CAD drawings, the LEDs are shown in blue.

The setup was imaged from above using a greyscale global-shutter camera (UI-3060CP-M-GL, IDS Imaging Development Systems GmbH, Obersulm, Germany), with a Nikon camera objective (AF Nikkor 50 mm f/1.8D, Nikon, Tokyo,

Japan), and with an emission filter (550 nm CWL, 100 nm FWHM; 33-331, Edmund Optics Inc., Barrington, NJ, US), placed after the objective, to pass as much of only the fluorescence light as possible. In relation to the electronics, the LED was connected to an LED driver (LEDD1B, Thorlabs Inc., Newton, NJ, US) to power it, and the camera to a custom-built PC running Windows 10 (Microsoft Corp., Redmond, WA, US) to accept images from it. The cameras frames were triggered using an Elegoo Uno R3 board (Elegoo, Shenzhen, China). The experiment involved acquiring images, at a frequency of 6 Hz, for 70 s, while, at a time point shortly after the camera was started, the LED array was driven at the maximum current possible with the LED driver used. In this case, only 8 of the LEDs, of equal spacing, were used. In order to make this possible, black tape was placed over the relevant LEDs to block the light emitted from them. The data corresponding to the video and final intensity image output are provided (LED Array/LEDArray_ExperimentVideo.tiff).

9.2 Simulation Setup and Protocol

A model of the system was created and run using the optical design software package Optic Studio 18.9 (Zemax LLC, Kirkland, WA, US). The file corresponding to this is provided (LED Array/LEDArray_Simulation.zmx). A 3D view of the model within the software is shown at the top of Figure S58. The LEDs were modelled using the "Source Radial" object, whereby the radial intensity profile was entered according to data provided by the manufacturer. The Petri dish was modeled as a volume object with a refractive index of 1.59, corresponding to that of polycarbonate, with no absorption. The **Dronpa-2** solution was modelled as a volume object with the material "WATER" selected. The CAD files for these two objects have been provided (LED Array/Liquid.stp, LED Array/PetriDish.stp). The software does not contain the transmission data of the **Dronpa-2** solution, and so it was entered manually as 71 % for 30 mm of optical path distance. In order to be able to measure the profile of absorbed light flux within the solution, a detector volume, 20 $\times$ 20 $\times$ 3.228 mm, containing 200 $\times$ 200 $\times$ 20 voxels, was set up fully covering the solution. The simulation was run with 1,000,000 rays with the settings "Use Polarization" and "Split NSC Rays" selected. Following this, the absorbed light flux for each slice, provided (LED Array/LEDArray_Intensity2DSLices.npy), was saved for further processing. A jupyter python notebook, containing the code which carries out the calculations described in the remainder of this section, is provided (LED Array/LEDArry_Code.ipynb).

9.3 Results and Discussion

9.3.1 Simulated 3D-distribution of absorbed light flux

The 2D image slices obtained from the simulation are provided in Figure S59. It can be seen that the light intensity distribution varies along the z-axis, with a bright central region being apparent in the lower slices and not the top. It is likely that the cause is related to reflection phenomena occurring at the bottom of the dish.

9.3.2 Simulated 2D-maps of the characteristic times of Dronpa-2 photoconversion and light intensity

Theoretical derivation The conversion of the simulated steady 3D-spatial distribution of light intensity $I(r,z)$ into a 2D map of characteristic time $\tau(r,z)$ is required to compare the simulation with the experimental observation.

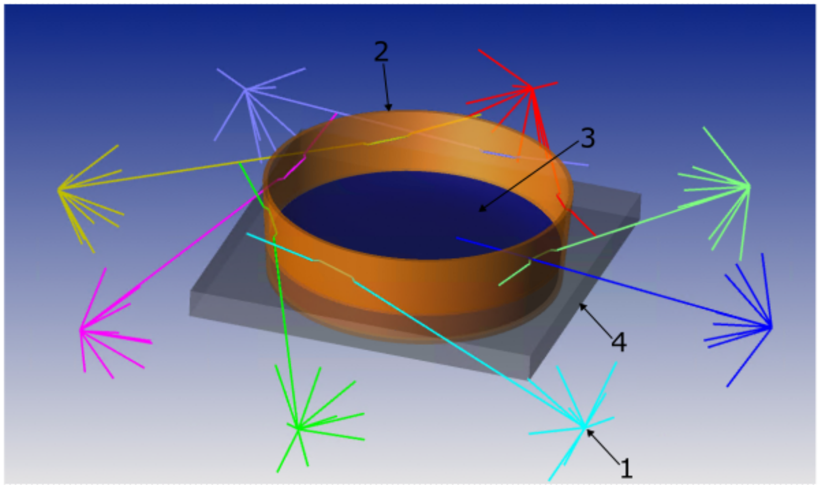

Figure S58: *3D view of the optical model of the Petri dish LED array setup within the Optic Studio 18.9 software*. 1 – LED, 2 – 35 mm Petri Dish, 3 – **Dronpa-2** solution, 4 – Detector Volume.

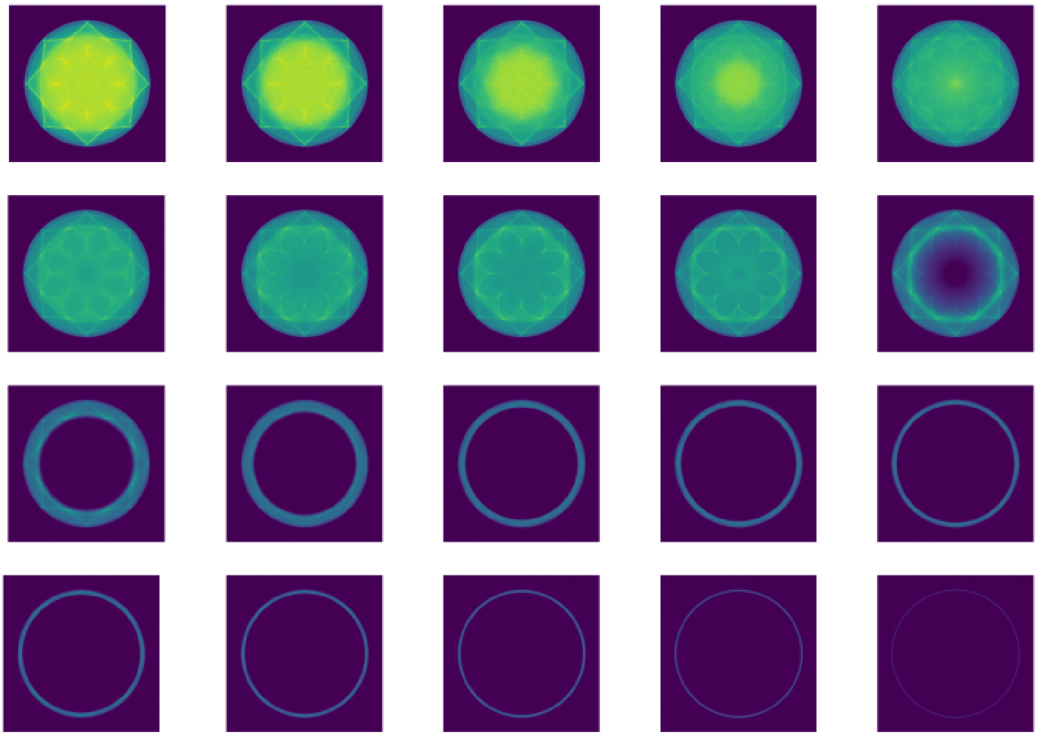

Figure S59: *2D images of the absorbed light flux at each of the 20 z-axis locations within the detection volume*. The first image corresponds to the **Dronpa-2** solution space adjacent to the bottom of the petri dish, and the following images, going left to right, and down, in a raster fashion, correspond to image slices progressively closer to the fluid surface. Since this work did not consider the absolute light intensity, but rather the distribution, a color scale is omitted.

The **Dronpa-2** solution contained in the Petri dish is divided in voxels, which are assumed to contain **Dronpa-2** at identical concentration. Furthermore, we consider large enough voxels to neglect diffusion effects involving transfer of the bright and dark states of **Dronpa-2** between voxels experiencing distinct light intensities. Under such conditions, the

conversion is performed as follows:

- In each voxel i, the time dependence of the fluorescence signal from **Dronpa-2** $F_i(r, z, t)$ obeys:

$$F_i(r, z, t) = F_i(r, z, \infty) + a_i(r, z) I_i(r, z) \exp[-\sigma I_i(r, z) t] \tag{S104}$$

  where $F_i(r, z, \infty)$ designates the final value of the fluorescence signal;

- One calculates the sums of the time dependences of the fluorescence signal from **Dronpa-2** along the optical axis $F(r, t)$

$$F(r, t) = \sum_{i=1}^{i=n} F_i(r, z, \infty) + \sum_{i=1}^{i=n} a_i(r, z) I_i(r, z) \exp[-\sigma I_i(r, z) t] \tag{S105}$$

  where the voxels along the optical axis are numbered from 1 to n;

- We assume $a_i(r, z) = a$ to be independent on r and z in the considered regime of light intensity. Then the sums of the time dependences of the fluorescence signal from **Dronpa-2** along the optical axis becomes

$$F(r, t) = \sum_{i=1}^{i=n} F_i(r, z, \infty) + a \sum_{i=1}^{i=n} I_i(r, z) \exp[-\sigma I_i(r, z) t] \tag{S106}$$

- Although multiexponentially decreasing, $F(r, t)$ is fitted with a monoexponential fitting function

$$F(r, t) = F(r, \infty) + b(r) \exp[-\sigma \langle I(r) \rangle t] \tag{S107}$$

  which provides the 2D-map of light intensity $\langle I(r) \rangle$.

- The 2D-map of $\tau(r)$ is eventually extracted as

$$\tau(r) = \frac{1}{\sigma \langle I(r) \rangle}. \tag{S108}$$

Results and discussion Maps of the characteristic times τ of **Dronpa-2** photoconversion, and light intensity I, for both the experimental and simulation cases, are provided in Figure S60. It can be seen visually that, although not an exact match, the results of both cases fairly agree with each other. More precisely, the eight-fold symmetry of the distribution is conveniently accounted for on both maps. One possible explanation for the small discrepancy arising from the weaker flower motif observed in the experimental maps is that the surface roughness, which could have impacted upon the reflections taking place, was not modelled for the simulation. Another possible factor causing differences could have been some inaccuracy in positioning between the plane of the LED array and the fluid. Despite the slight differences, this experiment demonstrates that the technique is able to extract information, closely matching that from a simulation. However, recovering information on light intensity in fully 3D cannot be retrieved from the integral image of light intensity displayed in Figure S60, which is a limit of this protocol.

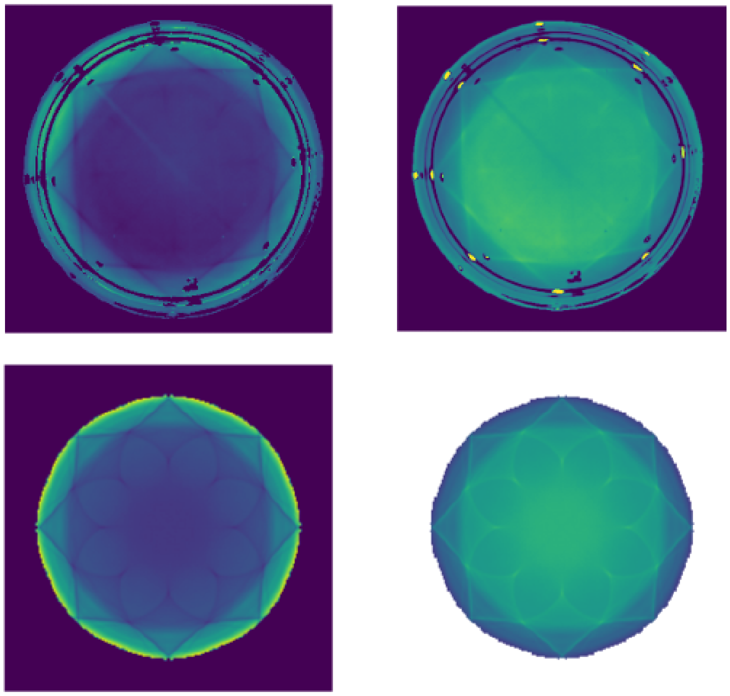

Figure S60: *τ (left) and light intensity (right) maps of the experimental (top) and simulation (bottom) situation.* Since this work did not consider the absolute light intensity, but rather the distribution, a color scale is omitted.

References

[1] Stéfan van der Walt, Johannes L. Schönberger, Juan Nunez-Iglesias, François Boulogne, Joshua D. Warner, Neil Yager, Emmanuelle Gouillart, Tony Yu, and the scikit-image contributors. scikit-image: image processing in Python. *PeerJ*, 2:e453, 6 2014.

[2] Matthew Newville, Till Stensitzki, Daniel B. Allen, and Antonino Ingargiola. LMFIT: Non-Linear Least-Square Minimization and Curve-Fitting for Python, September 2014.

[3] N. Gagey, P. Neveu, C. Benbrahim, B. Goetz, I. Aujard, J.-B. Baudin, and L. Jullien. Two-photon uncaging with fluorescence reporting: evaluation of the o-hydroxycinnamic platform. *Journal of the American Chemical Society*, 129(32):9986–9998, 2007.

[4] P. West and G. Davis. The synthesis of diarylnitrones. *The Journal of Organic Chemistry*, 54(21):5176–5180, 1989.

[5] Y. Shpinov, A. Schlichter, P. Pelupessy, T. Le Saux, L. Jullien, and B. Adelizzi. Unexpected acid-triggered formation of reversibly photoswitchable stenhouse salts from donor-acceptor stenhouse adducts. *Chemistry–A European Journal*, 2022.

[6] D. Joly and R. Carpentier. Sigmoidal reduction kinetics of the photosystem ii acceptor side in intact photosynthetic materials during fluorescence induction. *Photochemical & Photobiological Sciences*, 8(2):167–173, 2009.

[7] R. Chouket, A. Pellissier-Tanon, A. Lahlou, R. Zhang, D. Kim, M.-A. Plamont, M. Zhang, X. Zhang, P. Xu, N. Desprat, D. Bourgeois, A. Espagne, A. Lemarchand, T. Le Saux, and L. Jullien. Extra kinetic dimensions for label discrimination. *Nature communications*, 13(1):1–8, 2022.

[8] N. Kiskin and D. Ogden. Two-photon excitation and photolysis by pulsed laser illumination modelled by spatially non-uniform reactions with simultaneous diffusion. *Eur. Biophys. J.*, 30:571–587, 2002.

[9] N. Kiskin, R. Chillingworth, J. McCray, D. Piston, and D. Ogden. The efficiency of two-photon photolysis of a "caged" fluorophore, o-1-(2-nitrophenyl)ethylpyranine, in relation to photodamage of synaptic terminals. *Eur. Biophys. J.*, 30:588–604, 2002.

[10] P. Wang, L. Jullien, B. Valeur, J.-S. Filhol, J. Canceill, and J.-M. Lehn. Multichromophoric cyclodextrins. 5. antenna-induced unimolecular photoreactions. photoisomerization of a nitrone. *New journal of chemistry*, 20(7-8):895–907, 1996.

[11] J. Quérard, R. Zhang, Z. Kelemen, M.-A. Plamont, X. Xie, R. Chouket, I. Roemgens, Y. Korepina, S. Albright, E. Ipendey, M. Volovitch, H. L. Sladitschek, P. Neveu, L. Gissot, A. Gautier, J.-D. Faure, V. Croquette, T. Le Saux, and L. Jullien. Resonant out-of-phase fluorescence microscopy and remote imaging overcome spectral limitations. *Nat. Comm.*, 8:969, 2017.

[12] N. Gagey, P. Neveu, and L. Jullien. Reporting two-photon uncaging with the efficient 3,5-dibromo-2,4-dihydroxycinnamic caging group. *Angew. Chem. Intl. Ed.*, 46:2467–2469, 2007.

[13] D. Bourgeois and V. Adam. Reversible photoswitching in fluorescent proteins: a mechanistic view. *IUBMB life*, 64(6):482–491, 2012.

[14] A. Stiel, S. Trowitzsch, G. Weber, M. Andresen, C. Eggeling, S. Hell, S. Jakobs, and M. Wahl. 1.8 å bright-state structure of the reversibly switchable fluorescent protein Dronpa guides the generation of fast switching variants. *Biochem. J.*, 402:35–42, 2007.

[15] R. Ando, H. Mizuno, and A. Miyawaki. Regulated fast nucleocytoplasmic shuttling observed by reversible protein highlighting. *Science*, 306:1370–1373, 2004.

[16] H. Dürr and H. Bouas-Laurent. *Photochromism: molecules and systems, Elsevier*. Elsevier, 2003.

[17] M. Lerch, W. Szymanski, and B. Feringa. The (photo)chemistry of stenhouse photoswitches: guiding principles and system design. *Chem. Soc. Rev.*, 47:1910–1937, 2018.

[18] T. Mirkovic, E. Ostroumov, J. Anna, R. Van Grondelle, and G. Scholes. Light absorption and energy transfer in the antenna complexes of photosynthetic organisms. *Chemical reviews*, 117(2):249–293, 2017.

[19] A. Stirbet and Govindjee. On the relation between the kautsky effect (chlorophyll a fluorescence induction) and photosystem ii: basics and applications of the ojip fluorescence transient. *Journal of Photochemistry and Photobiology B: Biology*, 104(1-2):236–257, 2011.

[20] R. Delosme. Etude de l'induction de fluorescence des algues vertes et des chloroplastes au début d'une illumination intense. *Biochimica et Biophysica Acta (BBA)-Bioenergetics*, 143(1):108–128, 1967.

[21] R. Strasserf and A. Srivastava. Polyphasic chlorophyll a fluorescence transient in plants and cyanobacteria. *Photochemistry and photobiology*, 61(1):32–42, 1995.

[22] K. Koyano, H. Suzuki, Y. Mori, and I. Tanaka. Quantum yield of photo-rearrangement of nitrones. *Bulletin of the Chemical Society of Japan*, 43(11):3582–3587, 1970.

[23] P. Corey, R. Trimmer, and W. Biddlecom. A new chromogenic β-galactosidase substrate: 7-β-d-galactopyranosyloxy-9, 9-dimethyl-9h-acridin-2-one. *Angewandte Chemie International Edition in English*, 30(12):1646–1648, 1991.

[24] D. Warther, F. Bolze, J. Léonard, S. Gug, A. Specht, D. Puliti, X.-H. Sun, P. Kessler, Y. Lutz, J.-L. Vonesch, B. Winsor, J.-F. Nicoud, and M. Goeldner. Live-cell one-and two-photon uncaging of a far-red emitting acridinone fluorophore. *Journal of the American Chemical Society*, 132(8):2585–2590, 2010.

[25] C. Culbertson, S. Jacobson, and J. Ramsey. Diffusion coefficient measurements in microfluidic devices. *Talanta*, 56(2):365–373, 2002.

[26] K. Braeckmans, L. Peeters, N. Sanders, S. De Smedt, and J. Demeester. Three-dimensional fluorescence recovery after photobleaching with the confocal scanning laser microscope. *Biophysical journal*, 85(4):2240–2252, 2003.

[27] K. Braeckmans. Photobleaching with the confocal laser scanning microscope for mobility measurements and the encoding of microbeads. *Thesis submitted in fulfillment of the requirements for the degree of Doctor in Pharmaceutical Sciences, Ghent University*, 2004.

[28] M. Digman, C. Brown, P. Sengupta, P. Wiseman, A. Horwitz, and E. Gratton. Measuring fast dynamics in solutions and cells with a laser scanning microscope. *Biophysical journal*, 89(2):1317–1327, 2005.

[29] C. Brown, R. Dalal, B. Hebert, M. Digman, A. Horwitz, and E. Gratton. Raster image correlation spectroscopy (rics) for measuring fast protein dynamics and concentrations with a commercial laser scanning confocal microscope. *Journal of microscopy*, 229(1):78–91, 2008.

[30] R. Cole, T. Jinadasa, and C. Brown. Measuring and interpreting point spread functions to determine confocal microscope resolution and ensure quality control. *Nature protocols*, 6(12):1929–1941, 2011.

[31] C. Matthews and F. Cordeliéres. Metroloj: an imagej plugin to help monitor microscopes' health. In *ImageJ User & Developper Conference*, 2010.

[32] A. Paffi, M. Liberti, F. Apollonio, A. Sheppard, and Q. Balzano. In vitro exposure: Linear and non-linear thermodynamic events in petri dishes. *Bioelectromagnetics*, 36(7):527–537, 2015.
